# The Genome Sequence of the Fungal Pathogen *Fusarium virguliforme* That Causes Sudden Death Syndrome in Soybean

**DOI:** 10.1371/journal.pone.0081832

**Published:** 2014-01-14

**Authors:** Subodh K. Srivastava, Xiaoqiu Huang, Hargeet K. Brar, Ahmad M. Fakhoury, Burton H. Bluhm, Madan K. Bhattacharyya

**Affiliations:** 1 Department of Agronomy, Iowa State University, Ames, Iowa, United States of America; 2 Department of Computer Science, Iowa State University, Ames, Iowa, United States of America; 3 Department of Plant, Soil Science, and Agricultural Systems, Southern Illinois University, Carbondale, Illinois, United States of America; 4 Department of Plant Pathology, University of Arkansas, Fayetteville, Arkansas, United States of America; Soonchunhyang University, Republic of Korea

## Abstract

*Fusarium virguliforme* causes sudden death syndrome (SDS) of soybean, a disease of serious concern throughout most of the soybean producing regions of the world. Despite the global importance, little is known about the pathogenesis mechanisms of *F. virguliforme*. Thus, we applied Next-Generation DNA Sequencing to reveal the draft *F. virguliforme* genome sequence and identified putative pathogenicity genes to facilitate discovering the mechanisms used by the pathogen to cause this disease.

**Methodology/Principal Findings:**

We have generated the draft genome sequence of *F. virguliforme* by conducting whole-genome shotgun sequencing on a 454 GS-FLX Titanium sequencer. Initially, single-end reads of a 400-bp shotgun library were assembled using the PCAP program. Paired end sequences from 3 and 20 Kb DNA fragments and approximately 100 Kb inserts of 1,400 BAC clones were used to generate the assembled genome. The assembled genome sequence was 51 Mb. The N50 scaffold number was 11 with an N50 Scaffold length of 1,263 Kb. The AUGUSTUS gene prediction program predicted 14,845 putative genes, which were annotated with Pfam and GO databases. Gene distributions were uniform in all but one of the major scaffolds. Phylogenic analyses revealed that *F. virguliforme* was closely related to the pea pathogen, *Nectria haematococca*. Of the 14,845 *F. virguliforme* genes, 11,043 were conserved among five *Fusarium* species: *F. virguliforme*, *F. graminearum*, *F. verticillioides*, *F. oxysporum* and *N. haematococca*; and 1,332 *F. virguliforme-*specific genes, which may include pathogenicity genes. Additionally, searches for candidate *F. virguliforme* pathogenicity genes using gene sequences of the pathogen-host interaction database identified 358 genes.

**Conclusions:**

The *F. virguliforme* genome sequence and putative pathogenicity genes presented here will facilitate identification of pathogenicity mechanisms involved in SDS development. Together, these resources will expedite our efforts towards discovering pathogenicity mechanisms in *F. virguliforme.* This will ultimately lead to improvement of SDS resistance in soybean.

## Introduction

Crop plants encounter diverse fungal pathogens that cause a wide array of diseases and severely reduce yields. The fungal genus *Fusarium* is comprised of highly destructive pathogens that reduce crop productivity, contaminate harvested grains with mycotoxins, and in severe instances, cause crop failures that result in famines. Thus, effective management practices to control *Fusarium* pathogens are urgently needed to feed the world's rapidly growing human population. Biotechnological approaches based on knowledge gained from studying plant-fungal interactions hold significant promise to provide novel disease control strategies [1]. To facilitate genetic studies of the interactions between *Fusarium* fungi and crop species, genome sequences of four *Fusarium* species are currently available, *viz.*, *Fusarium oxysporum*, *F. graminearum*, *F. verticillioides*, and *Nectria haematococca* (*F. solani*) [2], [3].

*F. virguliforme* is a serious, yet comparatively understudied, fungal pathogen that causes sudden death syndrome (SDS) in soybean. The pathogen causes root necrosis and rot, as well as vascular discoloration of roots and stems. Root infection is often accompanied by foliar symptoms (foliar SDS), characterized initially by interveinal chlorosis followed by necrosis, and in severe cases, flower and pod abscission [4]. Interestingly, *F. virguliforme* has never been isolated from symptomatic foliar tissues, which strongly suggests that foliar symptoms result from translocated toxins produced in infected roots [5]. Severe yield losses are commonly associated with expression of foliar SDS symptoms. The major toxin that causes foliar SDS is a small acidic protein [6]–[8]. The toxin requires light to initiate foliar SDS symptoms [6], [9].

SDS was first detected in Arkansas in 1971, and has now spread to all soybean growing areas of the United States [6]. The disease is caused by two morphologically and phylogenetically distinct species within the *Fusarium solani* species complex, *F. tucamaniae* and *F. virguliforme* [10]. Populations of *F. tucamaniae*, which causes SDS in Argentina and Brazil, possess two mating types and thus possibly undergo sexual reproduction, whereas a sexual reproductive stage is most likely absent among populations of *F. virguliforme* [10], [11].

In this study, we sequenced the genome of the *F. virguliforme* Mont-1 strain by applying a shotgun sequencing approach. As expected, the *F. virguliforme* genome revealed high sequence identity to the genome sequences of previously sequenced *Fusarium* species [12]. To facilitate identification of candidate genes involved in SDS development, we searched the *F. virguliforme* genome with the pathogen-host interactions (PHI) sequence database (http://www.phi-base.org/) composed of experimentally verified pathogenicity, virulence and effector proteins from bacteria, fungi and oomycete pathogens that infect a wide range of hosts [13]. This approach identified 358 candidate pathogenicity genes in the *F. virguliforme* genome. In a parallel approach, we annotated 1,332 *F. virguliforme*-specific genes to identify candidate pathogenicity genes. The *F. virguliforme* genome sequence is available through the NCBI database DDBJ/EMBL/GenBank under the project ID (PID) 63281 and accession AEYB01000000 and can be viewed through a GMOD Generic Genome Browser (GBrowse) available at http://fvgbrowse.agron.iastate.edu.

The *F. virguliforme* genome sequence and putative pathogenicity genes presented here will facilitate the identification of pathogenicity mechanisms involved in SDS development and ultimately lead to a better management of SDS in soybean.

## Materials and Methods

### *F. virguliforme* isolate and DNA isolation

The sequenced *F. virguliforme* Mont-1 isolate, virulent to the soybean cultivar Essex, was produced from a single conidium. The genomic DNA was isolated from germinating conidia with a published genomic DNA isolation protocol [14]

### Genome sequencing and assembly

*F. virguliforme* Mont-1 DNA was sequenced in a 454 GS-FLX Titanium sequencing platform by SeqWright Inc. (Houston, TX). Three types of sequencing runs were conducted: (i) shotgun sequencing of ∼400 bp DNA fragments, (ii) sequencing of ∼3 kb paired-ends, and (iii) sequencing of ∼20 kb paired-ends. In collaboration with Lucigen, Inc. (Middleton, WI), a BAC library carrying approximately 100 Kb sheared DNA fragments [15] was constructed and both ends of 1,402 BAC clones were sequenced. Considering the use of both Sanger's dideoxy- and 454-sequence data in this study, we applied PCAP assembler software that can assemble both kinds of data [16]. Transcripts of *F. virguliforme* germinating conidia and mycelia were sequenced in an Illumina/Solexa genome Analyzer II (GAII) at the Iowa State University DNA Facility.

### Comparison of PCAP and Newbler assemblies

Newbler is a genome assembly program for 454 data. To compare the effectiveness of PCAP in assembling 454 data, we assembled libraries of 454-single reads and −3 kb paired-end reads using Newbler. The consensus sequences of each assembly derived either by PCAP or Newbler were assessed by mapping the assembled sequences of Illumina paired-end reads of the *F. virguliforme* Clinton-1B isolate onto the PCAP- and Newbler-derived assemblies using the Bowtie2 program [17] and calling single-nucleotide polymorphisms (SNPs) with SAMtools [18]. SNP rates for both assemblies were calculated.

### Gene prediction and annotation

The genes of the assembled *F. virguliforme* genome were predicted with the AUGUSTUS gene prediction program with options set for (i) *F. graminearum*, (ii) coding sequence and (iii) GFF [19]. The predicted genes were used as a reference set for mapping RNA sequences (http://fvgbrowse.agron.iastate.edu/) using the Bowtie program [20].

The preliminary annotation of the *F. virguliforme* genome was conducted using Pfam domain search and information was incorporated at the http://fvgbrowse.agron.iastate.edu/ genome browser. The Pfam database models were downloaded (http://Pfam.sanger.ac.uk/). The genome was annotated by conducting hmmscan search (HMMER 3.0, http://hmmer.org/). The BLAST2GO analysis was conducted at the http://www.blast2go.org/start_Blast2GO. The predicted *F. virguliforme* coding sequences were searched for identical sequences by conducting BLASTX search. A cut-off, E≤10^−10^ was used for BLASTX and annotation.

### Syntenic analysis between *Nectria haematococca* and *Fusarium virguliforme*

The homologous regions between *N. haematococca* chromosomes and *F. virguliforme* genomes were identified using MAUVE software [21]. Homologous co-ordinates were identified using NUCMER software [22] and matching *F. virguliforme* scaffolds with *N. haematococca* chromosomes was identified and visualized using MAUVE program. The chromosomes graph was visualized for locally collinear blocks (LCBs) with weight close to 5000 [21].

### Comparison and visualization of Pfam

The comparison of *F. virguliforme* (Scaffold 1) with the genome sequences of four *Fusarium* spp. was conducted using MUMmer genome comparison tool, and dot-pot was created using Mummer plot [22]. We used PROmer program [22], which aligns translated nucleotide sequences (six possible reading frames) with a filter of 100 nts to remove the noise. The visualization of Pfam annotation (heat map) was conducted using the multi-experiment visualization (MeV) tool [23]. We normalized the number of Pfam domain hits in an organism by dividing Pfam domain hits with total number of genes of that organism and then corrected with the respective SDs for visualization of the significant variation of a particular domain among species.

### Phylogenetic analysis of *F. virguliforme*

*F. virguliforme* and four *Fusarium* genomes along with *M. grisea*, *N. crassa*, *A. nidulans*, *R. oryzae*, *P. blakesleeanus*, *U. maydis* and the two oomycetes, *P. infestans* and *P. sojae*, were considered for phylogenetic analysis, which was conducted using 10 highly conserved (E≤10^−30^) single copy proteins; viz., g242, g255, g270, g326, g330, g364, g375, g380, g422 and g426 (http://fvgbrowse.agron.iastate.edu/). A maximum-likelihood tree was generated using the PHYML tool [24] (WAG model) with 1,000 bootstraps.

### Unique gene analysis

BLAST program (BLASTP) with a significant cut-off level, E≤10^−9^, was used to identify proteins common to all five *Fusarium* species or proteins that are unique to individual *Fusarium* species. Unique proteins (N) were calculated as N = X-Y-Z; where, X is the total number of proteins in a particular organism, Y is the proteins common to all *Fusarium* species, and Z is the number of proteins common to at least two *Fusarium* species.

### Analyses of the *F. virguliforme* genome for pathogenicity proteins

The *F. virguliforme* proteins were interrogated with the pathogenicity proteins of the pathogen-host interaction (PHI) database to identify candidate *F. virguliforme* pathogenicity proteins. PHI protein sequences were downloaded and interrogated with the predicted *F. virguliforme* proteins (BLAST locally with a cutoff, E≤10^−9^) to identify candidate *F. virguliforme* pathogenicity proteins. The selected sequences were reanalyzed to eliminate any false positives.

### Analyses of the *F. virguliforme* genome for secretory proteins

To identify the putative *F. virguliforme* secretory proteins, we analyzed the genome using SignalP program. The SignalP consists of two different predictors based on neural network and hidden Markov model algorithms Method. We used hidden Markov model algorithms and >0.9 probability value to identify the probable candidate secretory proteins.

## Results and Discussion

The *F. virguliforme* Mont-1 isolate used in this study was propagated from a single conidium and was confirmed to be virulent on soybean. Whole-genome shotgun sequencing was conducted on a *454*-GS-FLX Titanium platform. Initially, single-end reads of a 400-bp shotgun library were assembled using the PCAP genome assembly program [16]. The consensus genome sequence was determined from raw data with an average of 20-fold genome coverage. To facilitate assembly of the single read sequences into larger contigs, sequences of paired ends of approximately 3 and 20 Kb DNA molecules were obtained through sequencing on a 454 GS-FLX Titanium sequencer. In addition to paired-end sequences of random DNA fragments, sequences of both ends of inserts from 1,400 BAC clones with an average size 100 kb were obtained to support the assembly of shotgun sequences. The assembled genome sequence is 51 Mb (Table 1) with an N50 scaffold number of 11, an N50 scaffold length of 1,263 Kb and an N50 contig length of 73 Kb. The 1,386 scaffolds, which include 23 major scaffolds (0.5 to 5 Mb) and 1,363 (1 to 499 Kb) minor scaffolds, represent the entire 51 Mb genome sequence. The 51 Mb *F. virguliforme* genome sequence obtained in this study was comparable in size to the sequenced genomes of *F. graminearum* (36 Mb), *F. verticillioides* (42 Mb), *Nectria haematococca* (54 Mb) and *F. oxysporum* (60 Mb).

**Table 1:** General assembly statistics of the draft *Fusarium virguliforme* genome.

Number of contigs	3,098
Average length of contigs	16,284 bases
Total length of contigs	50,448,805 bases
Contig size N50	72,747 bases
N50 number of contigs	200
Number of scaffolds	1,386
Average length of scaffolds	36,757 bases
Total length of scaffolds	50,994,238 bases
Scaffold size N50	1,263,176 bases
N50 number of scaffolds	11
Total coverage	20×
Predicted genes	14,845

### Comparison of Newbler and PCAP assemblies

In assembling the genome sequence, we applied PCAP assembler software because this program can assemble sequences generated by both Sanger's dideoxy and pyrosequencing technologies, applied in this study. PCAP was originally developed for Sanger's dideoxy ABI 3730 reads. For assembling 454 sequence data, Newbler program was developed. Each assembly has its unique features. For example, PCAP produces support information from each type of read pairs for each region of every scaffold. The support information is useful in estimating the likelihood that a particular region of a scaffold is accurate. In addition, PCAP produces candidate SNPs with their consensus alignment columns and locations in scaffolds.

We compared the quality of the assembly obtained by PCAP with that of the assembly generated by Newbler by computing the SNP rates of these assemblies with that generated for the Illumina paired-end reads of the *F. virguliforme* Clinton-1B isolate. The SNP rate of the Newbler assembly with Clinton-1B reads was 1 SNP in 10,000 bp, whereas that for the PCAP assembly with Clinton-1B reads was 1.6 SNP in 10,000 bp. The low SNP rates suggest that both assemblies are equally effective in assembling 454 sequence data. The Newbler assembly is slightly more accurate in generating consensus sequences probably because it uses more trace information than PCAP, which uses only bases and their quality values.

### *F. virguliforme* is close relative of the pea pathogen, *N. haematococca*

**Figure 1:**
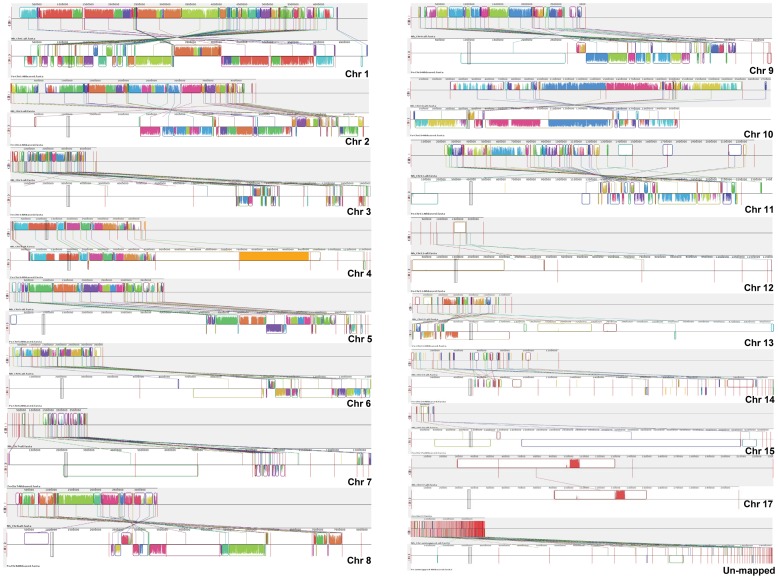
Synteny of *Fusarium virguliforme* Mont1 sequences to the *Nectria haematococca* chromosomal sequences. The colored blocks show the alignment of *F. virguliforme* sequences to the sequences of the *N. haematococca* chromosomes. Blocks below the central line indicate the regions that align in the reverse complement orientation.

To determine the evolutionary relationship of *F. virguliforme* with *F. graminearum*, *F. verticillioides*, *N. haematococca* and *F. oxysporum*, the largest scaffold (scaffold 1; 5.05 Mb) of the *F. virguliforme* genome was aligned with the genome sequences of these four *Fusarium* spp. *F. virguliforme* scaffold 1 showed different levels of conservation with the genomes of *F. verticillioides* (scaffold 3.1), *F. oxysporum* (scaffolds 2.1), *F. graminearum* (scaffold 3.1) and *N. haematococca* (scaffold Sca.1). The highest synteny was observed between *F. virguliforme* and *N. haematococca* (Figure S1) suggesting that from the evolutionary point of view, *F. virguliforme* is closet to *N. haematococca*. We, therefore, further investigated the local collinear blocks between the *F. virguliforme* Mont1 genome and *N. haematococca* chromosomes. Since the *F. virguliforme* sequenced genome has not been assigned to chromosomes, we identified the *N. haematococca* chromosome-specific *F. virguliforme* scaffolds using the NUCMER program [20]. The *N. haematococca* chromosome-specific *F. virguliforme* scaffolds were visualized using the MAUVE program [19]. Large *F. virguliforme* scaffolds, bigger than 5 kb, were mapped to the *N. haematococca* chromosomes and regions showing synteny are listed in Table S1 and Figure 1.

The contiguously colored regions are local collinear blocks (LCBs); i.e., regions without rearrangement of homologous backbone sequence [19]. LCBs below a genome's central line are in the reverse complement orientation relative to the reference genome. The highest number of LCBs was found in the N. haematococca chromosome 3 (56 LCBs) followed by chromosome 1 (48 LCBs). We did not identify any *F. virguliforme* scaffolds specific to *N. haematococca* chromosome 16.

The lack of greater levels of synteny throughout the genome may be due to rearrangement following separation of the two species from a common progenitor species. This led to development of mosaic patterns, unique to each species with least conservation between *Fusarium* species that are distantly related. Additionally, the high level of genomic variation likely stemmed in part from evolution of repeat sequences, low complexity sequences, or repeat-induced point mutation (RIP; [3]). These mechanisms have been reported to be the cause of genetic variations and source of genomic instability in other fungi [23]. The proportion of repeat sequences was determined by comparing the assembled *F. virguliforme* genome sequence to itself using the DDS2 program [25]. It is estimated that about 18% of the *F. virguliforme* genome is composed of repeat sequences, most of which contain low GC contents.

### Gene content and organization of genes in the *F. virguliforme* genome

To predict the number of genes in the *F. virguliforme* genome we analyzed the genome using the AUGUSTUS gene prediction program by setting the species option to *F. graminearum* species [19]. It is predicted that the genome contains 14,845 genes. This number is very close to the predicted gene numbers for the genomes of *F. graminearum* (13,332), *F. verticillioides* (14,179), *F. oxysporum* f. sp. *lycopersici* (17,735) , and *N. haematococca* (15,707). The average G + C content of the *F. virguliforme* coding regions was 49%. Transcripts from germinating conidia and mycelia of *F. virguliforme* were sequenced on an Illumina Genome Analyzer [26] and were aligned to the CDS sequences of the predicted genes for evidence of expression by using Bowtie program [20]. Of 14,845 predicted genes, 13,375 (90%) were expressed in germinating conidia, 13,281 (89%) in mycelia; and 14,070 (95%) showed transcripts at least in either or both conidia and mycelia.

**Figure 2:**
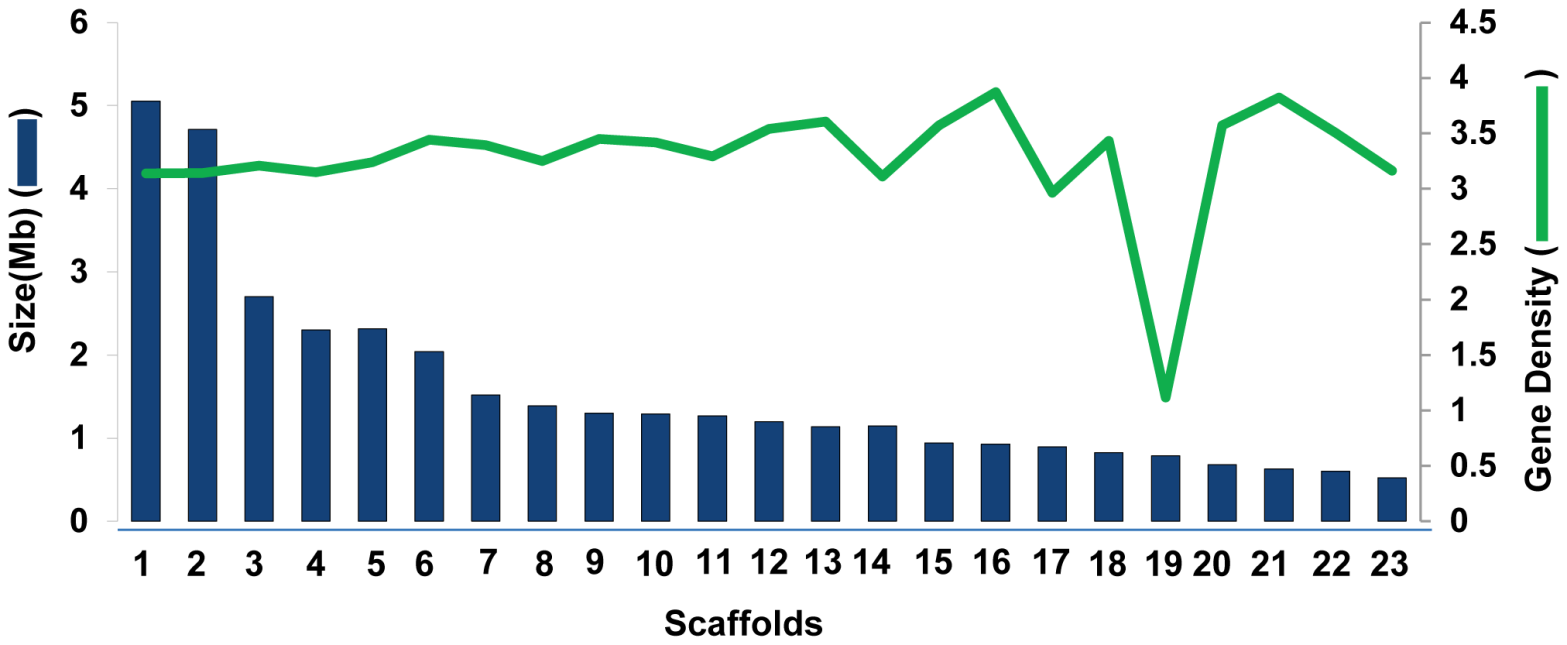
Non-uniform gene density in the *F. virguliforme* genome. Gene density of over 23 major scaffolds of the *F. virguliforme* genome is presented. Size of individual scaffold is presented in blue histograms covering 36.2 Mb (scale on the left) of the total 51 Mb genome. Gene density (number of genes/10 Kb) is presented with the green line (scale of the right).

Overall, gene density was lower in the *F. virguliforme* genome compared to other *Fusarium* species (Table S2). Gene density was ∼3 genes/10 Kb throughout most of the *F. virguliforme* genome except in Scaffold 19, which contains three-fold fewer genes (∼1 gene/10 Kb) (Figure 2). The G + C content of the coding regions of the Scaffold 19 is only 28% (Table S2), as compared to approximately 50% in the other scaffolds. A low G + C content in Scaffold 19 is due to accumulation of repeat sequences and could be the reason for a lower gene density in this genomic region. The G + C content is generally uniform among genes within a species [27], [28] and varies slightly among the *Fusarium* genomes (Table S3). The uniqueness of Scaffold 19 for gene density may also suggest a possible horizontal transfer of this genomic region from another species. Further studies will be required to determine if this is the case.

### Annotation of the *F. virguliforme* genes

**Figure 3:**
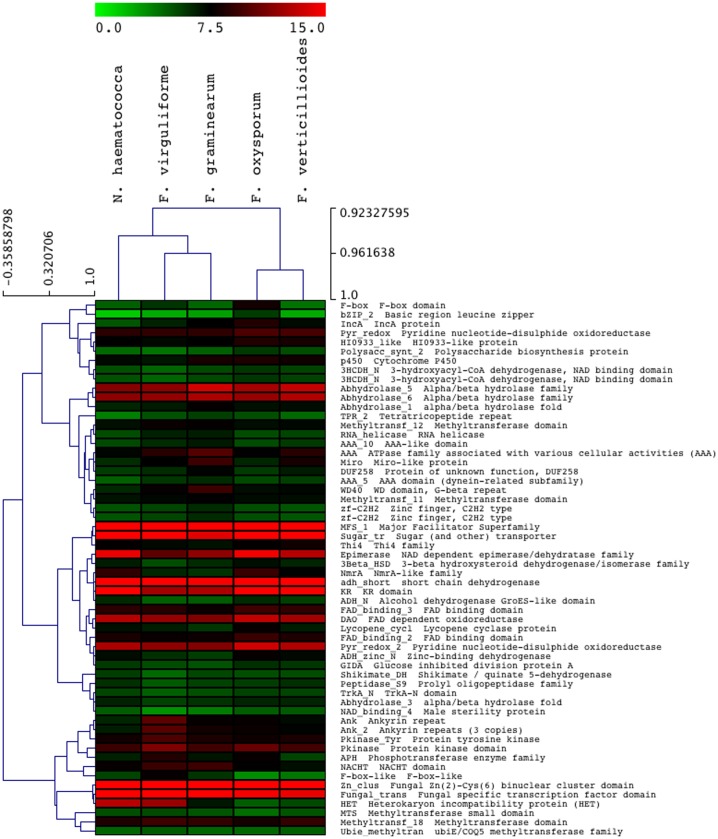
Heat-map depicting the Pfam domains in all *Fusarium* species. *F. virguliforme* genome is rich in Pkinase_Tyr (Protein tyrosine kinase), Ank (Ankyrin repeat), and HET (Heterokaryon incompatibility proteins) as compared to the other *Fusarium* genomes.

Predicted *F. virguliforme* genes were annotated by using Pfam [29] and BLAST2GO annotation protocols [30]. Annotations based on the Pfam database (e≤10^−9^) assigned functions to 78% of the predicted *F. virguliforme* proteins. The distribution of predicted proteins among functional classes was similar to that of *F. graminearum* , and was dominated by the following categories: MFS 1 (Major Facilitator Superfamily) (523), Zn_clus Fungal Zn(2)-Cys(6) binuclear cluster domain (396), Fungal trans (Fungal specific transcription factor domain) (311) and Sugar tr (Sugar and other transporters) domains (266) (Figures 3, S2; Table S4). The Pfam annotations of *Fusarium* relatives were analyzed with the multi-experiment viewer tool; and the annotation heat-map was generated using all Pfam domains (up to 100 hits). The standard deviation of genome Pfam domain was generated and used to divide with the number of Pfam hits in each respective genome to normalize the data (Figure 3; Table S5). This analysis revealed that many abundant domains were present in comparable proportions among the *Fusarium* genomes; e.g., Major Facilitator Superfamily (MFS 1), Sugar (and other) transporter (Sugar_tr), KR domain (KR), NAD dependent epimerase/dehydratase family (Epimerase), and short chain dehydrogenase (adh_short). On the other hand, domains such as protein tyrosine kinase (Pkinase_Tyr), ankyrin repeat (Ank), and heterokaryon incompatibility protein (HET) domains were significantly higher in number in the *F. virguliforme* genome [31], [32]. These protein domains have been reported to be involved in pathogenicity and could be important in SDS development. GO annotation of the 14,845 genes were conducted using BLASTX program (e≤10^−10^). A functional annotation was assigned to 14,810 (99.76%) genes and 7,954 (53.58%) of these were grouped into 16 functional categories (Figure S2). The majority of the genes were classified into metabolic processes followed by cellular processes, suggesting that most of the genes are required for basal metabolism and housekeeping functions. The Pfam annotated and un-annotated genes are presented in Gbrowse (http://fvgbrowse.agron.iastate.edu). The minimum length of predicted *F. virguliforme* coding sequences was 201 nucleotides and the average length was 1,482 nucleotides.

### *F. virguliforme* proteins conserved across species

To determine the number of *F. virguliforme* genes that are broadly conserved across diverse taxa, we conducted BLASTP analyses across the genomes of 25 organisms (Table S6) [33] (e≤10^−9^) using Standalone BLAST (version 2.2.15) search tools (http://BLAST.ncbi.nlm.nih.gov/) and results are presented in Figure S3 and Table S7. The most closely related organisms included *N. haematococca*, *F. oxysporum*, *F. graminearum*, *F. verticillioides*, *Neurospora crassa*, *Aspergillus nidulans*, *Ustilago maydis*, *Phycomyces blakesleeanus*, *Rhizopus oryzae*, and *Saccharomyces cerevisiae.* More distantly related organisms included *Danio rerio*, *Glycine max*, *Arabidopsis thaliana*, *Homo sapiens*, *Phytophthora sojae*, *Phytophthora infestans*, *Dictyostelium discoideum*, *Zea mays*, *Oryza sativa ssp. japonica*, *Drosophila melanogaster*, *Caenorhabditis elegans*, *Rhizobium leguminosarum*, *Pseudomonas syringae*, *Agrobacterium tumefaciens* and *Escherichia coli*. More than 80% of the *F. virguliforme* proteins shared high identity with proteins of the closely related *Fusarium* species *viz*. *N. haematococca, F. oxysporum, F. graminearum and F. verticillioides*. Among the organisms investigated, 2,076 of the *F. virguliforme* proteins showed identity to corresponding proteins of the distant species. For example, a large proportion of *E. coli* (42%) proteins are conserved in *F. virguliforme* (Table S7).

We searched for conserved genes (E≤10^−9^) via BLASTP in a step-by-step fashion as described below. First, we identified 13,068 *N. haematococca* proteins that showed identity (E≤10^−9^) to *F. virguliforme* proteins. Of these 13,068 *N. haematococca* proteins, 11,878 showed identity to *F. oxysporum* proteins. We used these 11,878 proteins in the next step of the search and so on (Figure S4; Table S8). We identified 762 *F. virguliforme* proteins (5.13%) that are conserved in all organisms including *E. coli*. The set of 762 *F. virguliforme* proteins (Table S9) conserved in all 26 organisms was classified by molecular function into 18 groups (Figure S5). As expected, many of the conserved proteins regulate housekeeping functions such as metabolic, cellular and developmental processes in all organisms.

### Identification of genes unique to *F. virguliforme*

In order to identify the genes unique to *F. virguliforme*, we compared the genome sequences of *F. virguliforme* and four closely related *Fusarium* species (Table S8) by conducting BLASTP analyses (E≤10^−9^) [33]. We identified 11,043 genes that were common to all five *Fusarium* species (Figure S6). Of the 14,845 *F. virguliforme* genes, 1,332 were unique to *F. virguliforme*. Further investigation of these 1,332 genes revealed that most were novel; only 98 of the 1,332 unique *F. virguliforme* genes showed similarities to known genes (Table S10). Based on GO annotations (Blast2GO; biological process), the 98 unique *F. virguliforme* genes were classified into 19 groups (Figure S7). Potential pathogenesis-related genes in this group included a polyketide synthase, protein serine threonine kinase and carbonic anhydrase, all of which could play important roles in initiating SDS in soybean (Table S10) [34]–[36].

### Phylogenic analysis of *F. virguliforme*

**Figure 4:**
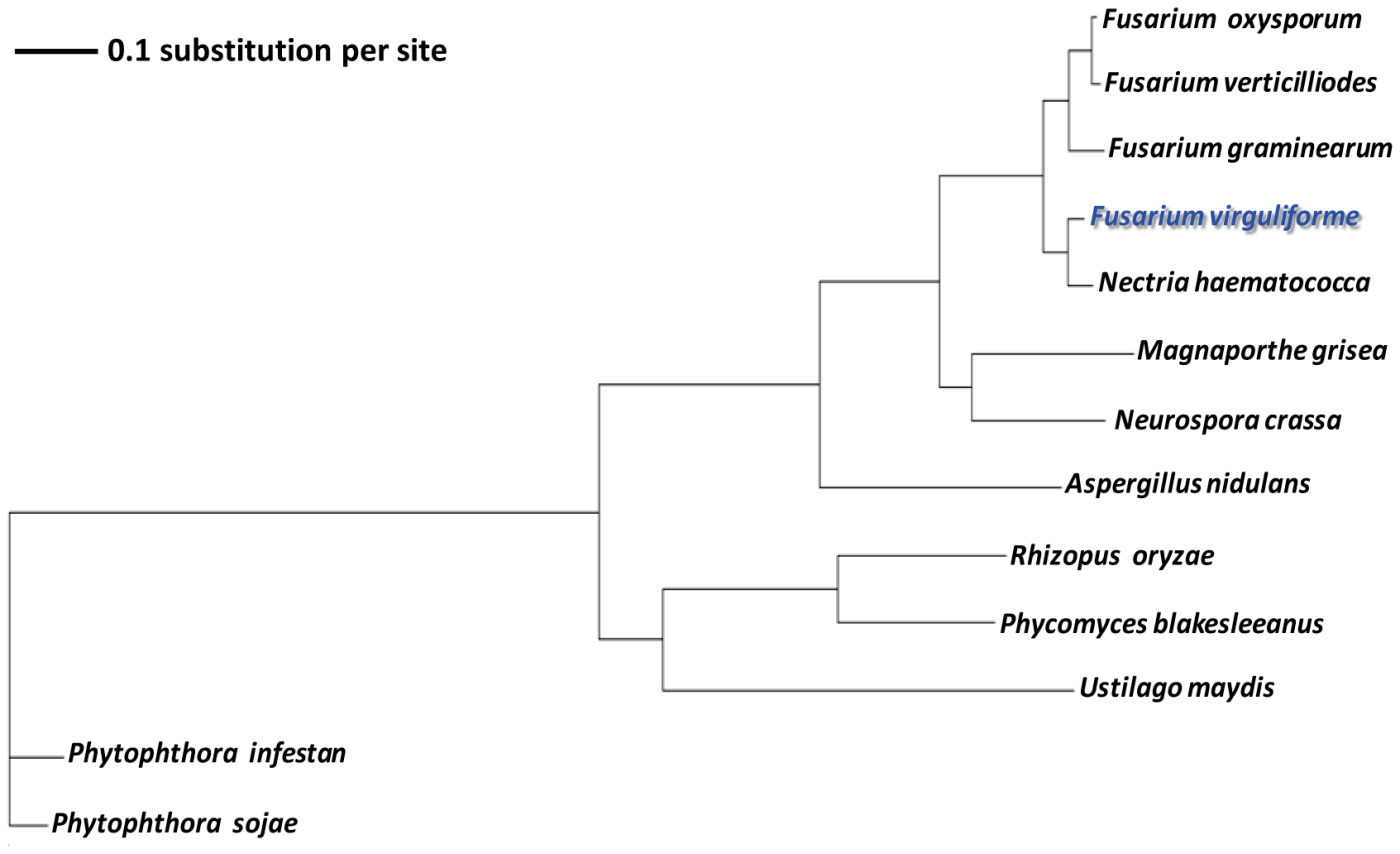
Phylogenetic tree showing the relationship of *F. virguliforme* with other *Fusarium* species. The tree was constructed using 10 randomly selected single copy orthologous genes using PHYML program (WAG model of evolution) with 1,000 bootstraps.

A phylogenic analysis was conducted to determine the relatedness of *F. virguliforme* to other *Fusarium* species. Ten orthologous, single copy genes were selected arbitrarily to construct a phylogenetic tree of five *Fusarium* species, *M. grisea* [37], *N. crassa* [38], *A. nidulans* [39], *R. oryzae* [40], *P. blakesleeanus* (http://genome.jgi-psf.org/Phybl1/Phybl1.home.html) *and U. maydis* (http://www.broadinstitute.org/). The oomycete pathogens, *P. sojae* (soybean pathogen) [41] and *P. infestans* (potato pathogen) [42], were included as taxonomic out-groups [24]. The five *Fusarium* species grouped together in one clade suggesting their origin from a single progenitor species. As expected, the oomycete pathogens grouped into a separate, more distant clade. The *Fusarium* clade was closest to the rice blast pathogen, *M. grisea* (Figure 4). Within this clade, *F. virguliforme* formed a sub-clade with the pea pathogen *N. haematococca*, which suggests that *N. haematococca* is the closest relative of *F. virguliforme* among the sequenced *Fusarium* species. This result supports our observations from the synteny study presented in Figure 1.

### Identification of candidate pathogenicity proteins

Two approaches were applied to identify candidate pathogenicity proteins. Firstly, the *F. virguliforme* genome sequence was interrogated with sequences of the pathogen-host interaction database (PHI database; http://www.phi-base.org/), consisted of experimentally verified pathogenicity, virulence, and effector proteins from bacteria, fungi and oomycetes that infect plants, humans, animals, insects, fishes and fungi. Of the 1,100 proteins in the PHI database, 786 pathogenicity genes are from fungi, 27 from oomycetes, 137 from bacteria and the rest are effector proteins [13]. The *F. virguliforme* genome was searched with the PHI protein database (E≤10^−9^) to identify possible pathogenicity genes. The 358 *F. virguliforme* proteins showing high sequence identity to members of the PHI protein database (Table S11) were classified into 21 groups based on GO annotation (biological process/level 2) (Figure S8). A substantial percent of the 358 genes were involved in metabolic processes; many were predicted to be involved in the biosynthesis of secondary metabolites. We identified five polyketide synthase genes that may be involved in the biosynthesis of non-proteinacious toxin (Table S11). We identified three pectate lyases, which could be involved in root necrosis or root rot. Another candidate pathogenicity protein (*Fv2806*) showed identity to the *Pseudomonas syringae* type III effector HopI1 protein (AAL84247.1). This list of pathogenicity genes laid the foundation for dissecting the pathogenicity mechanisms through functional analyses of these genes.

Some of the pathogenicity proteins are excreted to the extracellular space. These proteins carry signal sequences for excretion. In the second approach, we applied the SignalP program [43] to identify proteins containing signal peptides. Among the 14,845 predicted *F. virguliforme* proteins, use of the hmm model with the cut off 0.9 hmm score identified 1,155 putative secretory proteins (Table S12). These proteins were annotated using Blast2go and classified into eight groups (Figure S9). A large number of these proteins contain catalytic and binding activity sites. Some of these proteins could be important pathogenicity factors for SDS development in soybean.

## Conclusions

Here, we present the genome sequence of *F. virguliforme*, an important soybean pathogen that causes losses estimated to be over $0.1 billion annually in the United States [44]. Although SDS is characterized by distinctive foliar symptoms, the pathogen is exclusively found in roots of diseased plants. One or more fungal toxins have long been suspected to induce foliar SDS symptoms, although the current understanding of symptom development is fragmentary. In order to identify candidate pathogenicity genes, we interrogated the *F. virguliforme* genome sequence with the pathogen-host interactions sequence database and identified 358 candidate pathogenicity genes (Table S11). These include five polyketide synthases, which may be involved in the synthesis of polyketide toxins [45]. We also identified three pectate lyases that may be involved in cell wall degradation in root tissues to cause root necrosis or rotting. Additionally, we identified a candidate pathogenicity protein that showed high similarity to a bacterial effector protein (AAL84247.1). Among the identified 1,332 unique *F. virguliforme* genes, only 98 showed similarity to previously isolated genes. One of the 98 genes encodes a polyketide synthase, which may be involved in toxin biosynthesis [35].

Comparisons of the *F. virguliforme* genome with that of four *Fusarium* pathogens revealed new information about the relatedness of the five species and fundamental genomic similarities shared by these pathogenic species. Among the *Fusarium* species studied, the pea pathogen *N. haematococca* (*F. solani*) is the closest relative of *F. virguliforme*. The genome size of *F. virguliforme* is comparable to that of the previously sequenced *Fusarium* species. We observed that the G + C content in the gene-poor regions of the *F. virguliforme* genome was reduced approximately to half of the average G + C content of the genome. Furthermore, we identified a set of 762 *F. virguliforme* proteins (Table S9) that are conserved across a set of 26 organisms including *F. virguliforme*. The 762 conserved proteins, as expected, primarily regulate metabolic and cellular functions (Figure S5).

In summary, through this investigation, we have assembled the *F. virguliforme* genome sequence by conducting shotgun 454-sequencing and identified a set of candidate pathogenicity genes for discovering the pathogenicity mechanisms used by this serious soybean pathogen to cause SDS. Genome sequence reported here would become important public resource to a broad community of researchers engaged in developing tools to manage SDS, one of the most devastating diseases affecting global soybean production.

## Supporting Information

Figure S1 **Dot-plot analyses of** ***F. virguliforme*** **with four** ***Fusarium*** **spp.** The alignments are between *Fusarium virguliforme* Scaffold 1 (5.05 Mb) and genome sequences of the *Fusarium* spp. A) *F. virguliforme* with *F. graminearum* (8.93 Mb); B) *F. virguliforme* with *F. verticilliodes* (4.62 Mb); C) *F. virguliforme* with *F. oxysporum* (4.35 Mb); D) *F. virguliforme* with *N. haematococca* (4.93 Mb).
(PPT)

Figure S2 **Classification of the** ***F. virguliforme*** **genes based on GO annotation.** Biological process was considered in classifying the genes.
(PPT)

Figure S3 **Extent of similarity of** ***F. virguliforme*** **genes with that of the selected organisms.** The blue line indicates the percentage *F. virguliforme* genes that are similar (E≤9) to selected organisms. Brown line represents the proportion of genes in an individual that showed similarity (E≤9) to *F. virguliforme* genes.
(PPT)

Figure S4 **Conserved** ***F. virguliforme*** **proteins across a wide range of species.** Organisms investigated were *F. virguliforme (Fv), N. haematococca (Nh), F. oxysporum (Fo), F. graminearum (Fg), F. verticillioides (Fvt), N. crassa (Nc), A. nidulans (An), U. maydis (Um), P. blakesleeanus (Pb), R. oryzae (Ro), S. cerevisiae (Sc), D. rerio (Dr), D. melanogaster (Dm), G. max (Gm), A. thaliana (At), H. sapiens (Hs), P. sojae (Ps), P. infestans (Pi), R. leguminosarum (Rl), O. sativa ssp. japonica (Osj), D. discoideum (Dd), Z. mays (Zm), P. syringae (Ps), A. tumefaciens (Atu), E. coli (Ec), C. elegans (Ce).* Conserved *F. virguliforme* gene numbers in a species are shown in parenthesis.
(PPT)

Figure S5 **GO annotation of 762** ***F. virguliforme*** **proteins that are conserved across 25 diverse organisms.** A large number of the conserved proteins control metabolic, cellular and developmental processes.
(PPT)

Figure S6 **Unique** ***F. virguliforme genes*** **.** There are 11,403 genes that are common to all five *Fusarium* species and 1,332 genes unique to *F. virguliforme* at E≤10^−9^. The numbers in the light blue circle are the number of genes of a species that showed similarity (E≤10^−9^) to genes of at least another species.
(PPT)

Figure S7 **GO annotation of 98 unique** ***F. virguliforme*** **genes based on biological process.**
(PPT)

Figure S8 **GO annotation of 358 candidate pathogenicity proteins based on biological process.**
(PPT)

Figure S9 **GO annotation of 1,155 putative secretory proteins based on biological process.**
(PPT)

Table S1 **Alignments of coordinates between** ***N. haematococca*** **and** ***F. virguliforme*** **Mont1 genomes.**
(DOC)

Table S2 **Gene density and GC contents across** ***Fusarium species.***
(DOC)

Table S3 **Variation in gene density and G + C contents of coding sequences observed among 23 major scaffolds of the** ***F. virguliforme*** **genome.**
(DOC)

Table S4 **Pfam functional domain groups^1^.**
(DOC)

Table S5 **List of the normalized^1^ pfam domains of** ***Fusarium*** **species.**
(DOC)

Table S6 **Organisms and data sources used in genome analyses.**
(DOC)

Table S7 ***F. virguliforme*** **(** ***Fv*** **) genes thaxft showed similarity to genes of other organisms.**
(DOC)

Table S8 **Conserved** ***F. virguliforme*** **genes among selected organisms.**
(DOC)

Table S9 **The 762** ***F. virguliforme*** **genes that are conserved among 25 diverse organisms.**
(DOC)

Table S10 **GO annotation of 98 of the 1,332 unique** ***F. virguliforme*** **genes.**
(DOC)

Table S11 **Identification of candidate 358 pathogenicity genes through interrogation of the** ***F. virguliforme*** **genome with the PHI database.**
(DOC)

Table S12 **Candidate** ***F. virguliforme*** **secretory proteins.**
(DOC)
