# Supplementary material for: The Genome Sequence of the Fungal Pathogen Fusarium virguliforme That Causes Sudden Death Syndrome in Soybean
Source: PLoS One. 2014 Jan 14;9(1):e81832. doi: 10.1371/journal.pone.0081832 (PMC3891557; doi:10.1371/journal.pone.0081832)
Supplement: Figure S1 — Dot-plot analyses of F. virguliforme with four Fusarium spp. The alignments are between Fusarium virguliforme Scaffold 1 (5.05 Mb) and genome sequences of the Fusarium spp. A) F. virguliforme with F. graminearum (8.93 Mb); B) F. virguliforme with F. verticilliodes (4.62 Mb); C) F. virguliforme with F. oxysporum (4.35 Mb); D) F. virguliforme with N. haematococca (4.93 Mb). (PPT) [file pone.0081832.s001.ppt]

## Slide 1
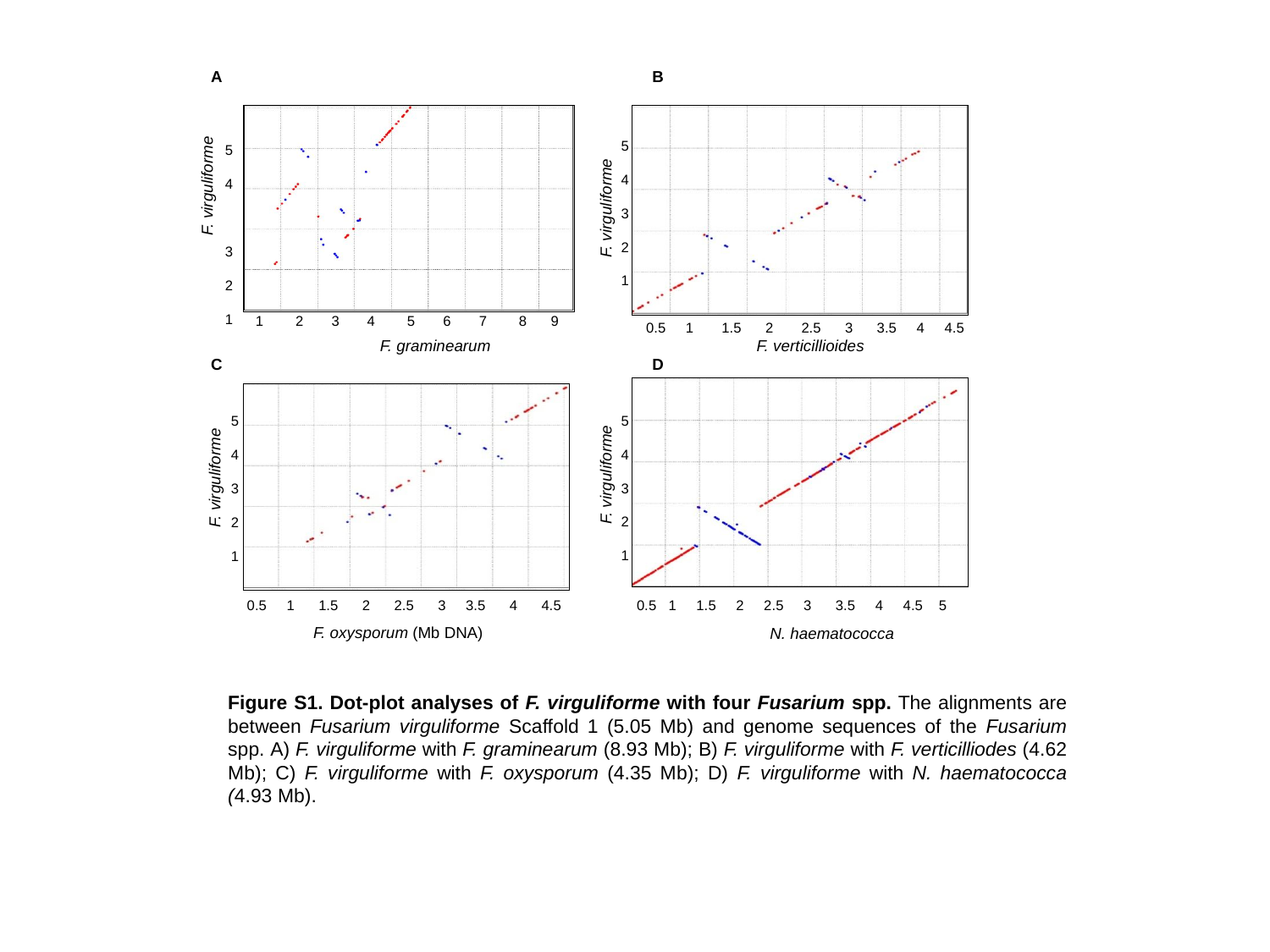

A
B
5
4
3
2
1
5
4
3
2
1
F. virguliforme
F. virguliforme
1 2 3 4 5 6 7 8 9
 0.5 1 1.5 2 2.5 3 3.5 4 4.5
F. graminearum
F. verticillioides
C
D
5
4
3
2
1
5
4
3
2
1
F. virguliforme
F. virguliforme
 0.5 1 1.5 2 2.5 3 3.5 4 4.5
 0.5 1 1.5 2 2.5 3 3.5 4 4.5 5
F. oxysporum (Mb DNA)
N. haematococca
Figure S1. Dot-plot analyses of F. virguliforme with four Fusarium spp. The alignments are between Fusarium virguliforme Scaffold 1 (5.05 Mb) and genome sequences of the Fusarium spp. A) F. virguliforme with F. graminearum (8.93 Mb); B) F. virguliforme with F. verticilliodes (4.62 Mb); C) F. virguliforme with F. oxysporum (4.35 Mb); D) F. virguliforme with N. haematococca (4.93 Mb).
