# Supplementary material for: The Genome Sequence of the Fungal Pathogen Fusarium virguliforme That Causes Sudden Death Syndrome in Soybean
Source: PLoS One. 2014 Jan 14;9(1):e81832. doi: 10.1371/journal.pone.0081832 (PMC3891557; doi:10.1371/journal.pone.0081832)
Supplement: Figure S2 — Classification of the F. virguliforme genes based on GO annotation. Biological process was considered in classifying the genes. (PPT) [file pone.0081832.s002.ppt]

## Slide 1
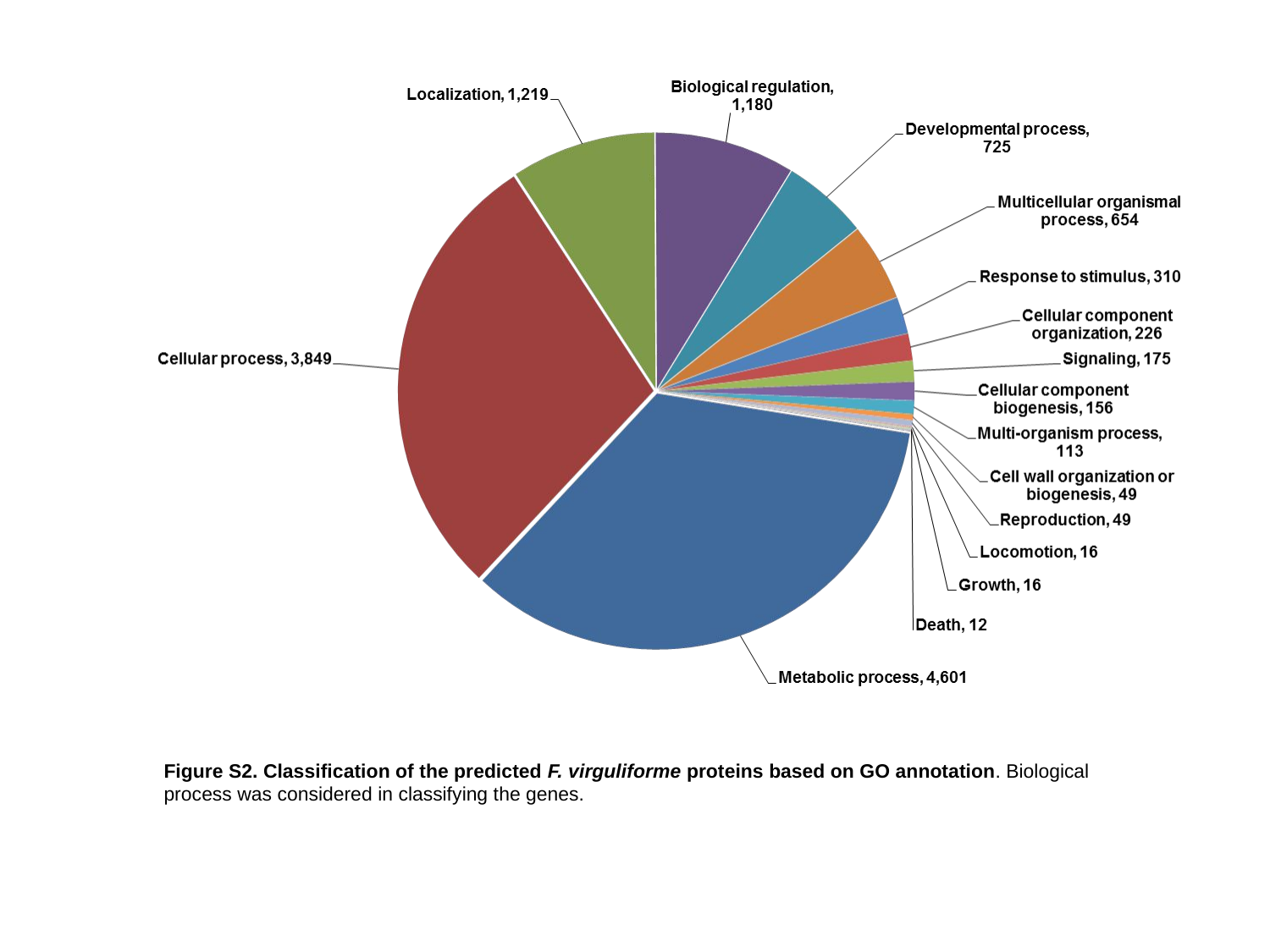

Figure S2. Classification of the predicted F. virguliforme proteins based on GO annotation. Biological process was considered in classifying the genes.
