# Supplementary material for: The Genome Sequence of the Fungal Pathogen Fusarium virguliforme That Causes Sudden Death Syndrome in Soybean
Source: PLoS One. 2014 Jan 14;9(1):e81832. doi: 10.1371/journal.pone.0081832 (PMC3891557; doi:10.1371/journal.pone.0081832)
Supplement: Figure S3 — Extent of similarity of F. virguliforme genes with that of the selected organisms. The blue line indicates the percentage F. virguliforme genes that are similar (E≤9) to selected organisms. Brown line represents the proportion of genes in an individual that showed similarity (E≤9) to F. virguliforme genes. (PPT) [file pone.0081832.s003.ppt]

## Slide 1
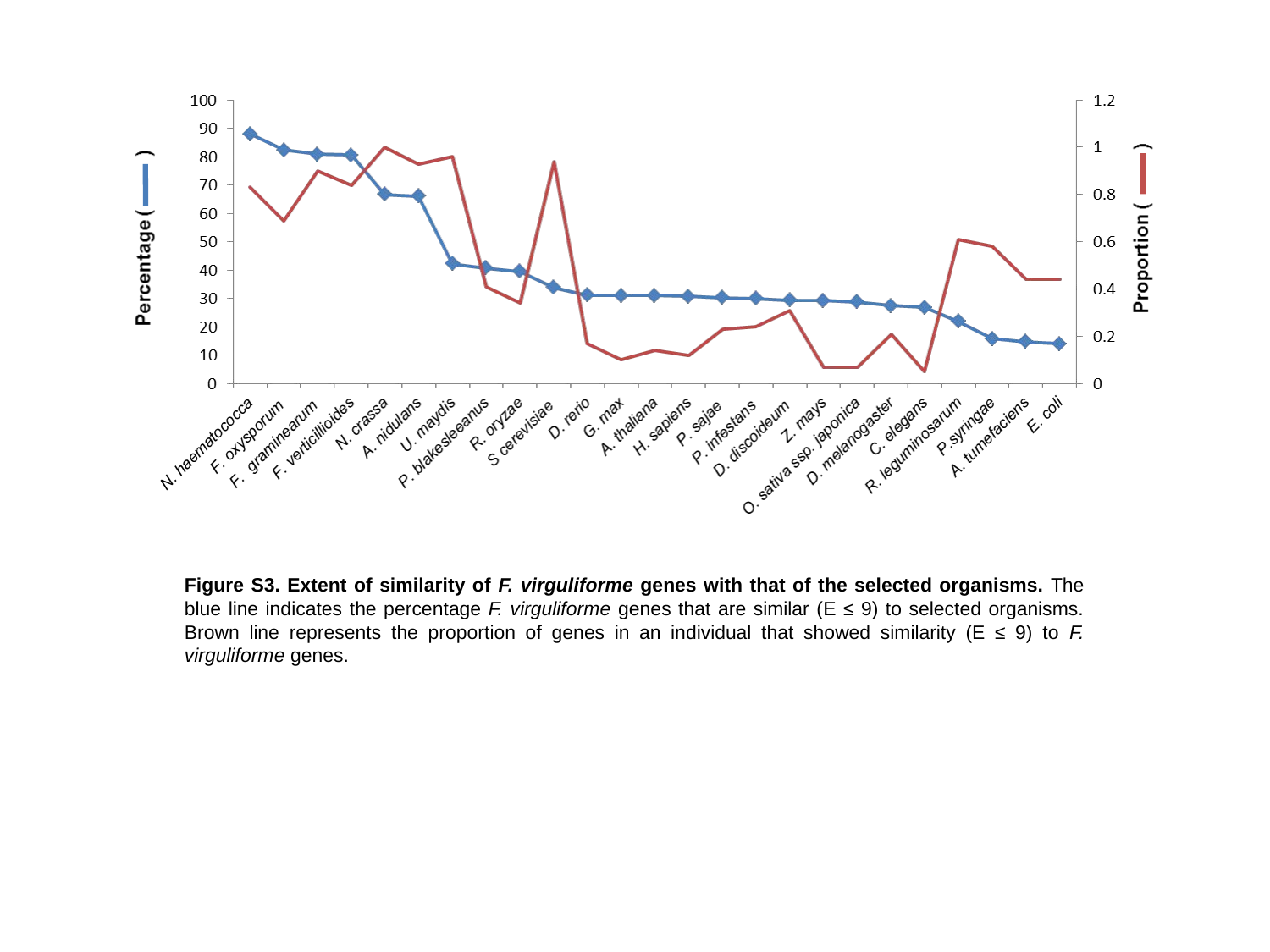

Figure S3. Extent of similarity of F. virguliforme genes with that of the selected organisms. The blue line indicates the percentage F. virguliforme genes that are similar (E ≤ 9) to selected organisms. Brown line represents the proportion of genes in an individual that showed similarity (E ≤ 9) to F. virguliforme genes.
