# Supplementary material for: The Genome Sequence of the Fungal Pathogen Fusarium virguliforme That Causes Sudden Death Syndrome in Soybean
Source: PLoS One. 2014 Jan 14;9(1):e81832. doi: 10.1371/journal.pone.0081832 (PMC3891557; doi:10.1371/journal.pone.0081832)
Supplement: Figure S4 — Conserved F. virguliforme proteins across a wide range of species. Organisms investigated were F. virguliforme (Fv), N. haematococca (Nh), F. oxysporum (Fo), F. graminearum (Fg), F. verticillioides (Fvt), N. crassa (Nc), A. nidulans (An), U. maydis (Um), P. blakesleeanus (Pb), R. oryzae (Ro), S. cerevisiae (Sc), D. rerio (Dr), D. melanogaster (Dm), G. max (Gm), A. thaliana (At), H. sapiens (Hs), P. sojae (Ps), P. infestans (Pi), R. leguminosarum (Rl), O. sativa ssp. japonica (Osj), D. discoideum (Dd), Z. mays (Zm), P. syringae (Ps), A. tumefaciens (Atu), E. coli (Ec), C. elegans (Ce). Conserved F. virguliforme gene numbers in a species are shown in parenthesis. (PPT) [file pone.0081832.s004.ppt]

## Slide 1
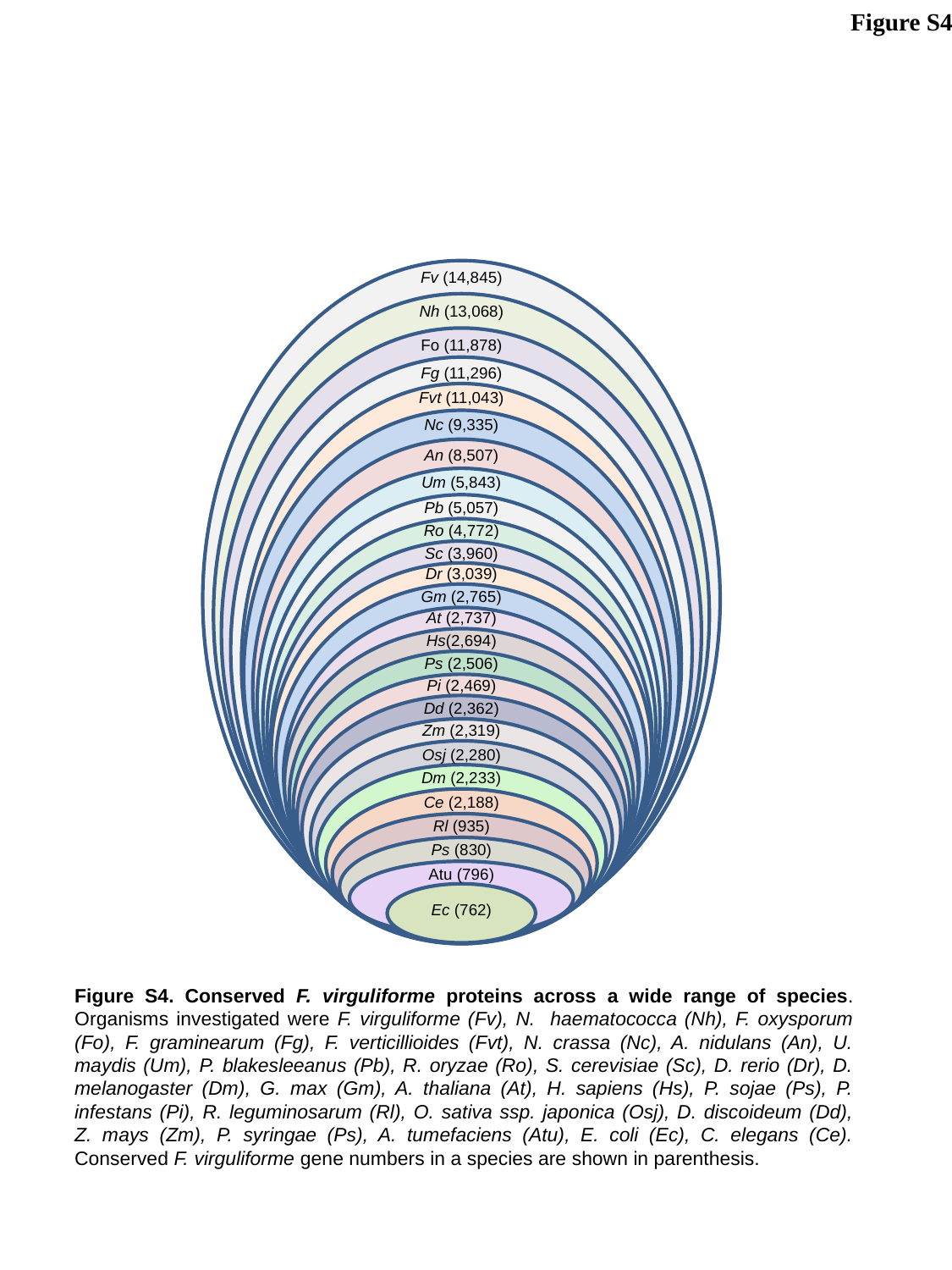

Figure S4
Fv (14,845)
Nh (13,068)
Fo (11,878)
Fg (11,296)
Fvt (11,043)
Nc (9,335)
An (8,507)
Um (5,843)
Pb (5,057)
Ro (4,772)
Sc (3,960)
Dr (3,039)
Gm (2,765)
At (2,737)
Hs(2,694)
Ps (2,506)
Pi (2,469)
Dd (2,362)
Zm (2,319)
Osj (2,280)
Dm (2,233)
Ce (2,188)
Rl (935)
Ps (830)
Atu (796)
Ec (762)
Figure S4. Conserved F. virguliforme proteins across a wide range of species. Organisms investigated were F. virguliforme (Fv), N. haematococca (Nh), F. oxysporum (Fo), F. graminearum (Fg), F. verticillioides (Fvt), N. crassa (Nc), A. nidulans (An), U. maydis (Um), P. blakesleeanus (Pb), R. oryzae (Ro), S. cerevisiae (Sc), D. rerio (Dr), D. melanogaster (Dm), G. max (Gm), A. thaliana (At), H. sapiens (Hs), P. sojae (Ps), P. infestans (Pi), R. leguminosarum (Rl), O. sativa ssp. japonica (Osj), D. discoideum (Dd), Z. mays (Zm), P. syringae (Ps), A. tumefaciens (Atu), E. coli (Ec), C. elegans (Ce). Conserved F. virguliforme gene numbers in a species are shown in parenthesis.
