# Supplementary material for: The Genome Sequence of the Fungal Pathogen Fusarium virguliforme That Causes Sudden Death Syndrome in Soybean
Source: PLoS One. 2014 Jan 14;9(1):e81832. doi: 10.1371/journal.pone.0081832 (PMC3891557; doi:10.1371/journal.pone.0081832)
Supplement: Figure S5 — GO annotation of 762 F. virguliforme proteins that are conserved across 25 diverse organisms. A large number of the conserved proteins control metabolic, cellular and developmental processes. (PPT) [file pone.0081832.s005.ppt]

## Slide 1
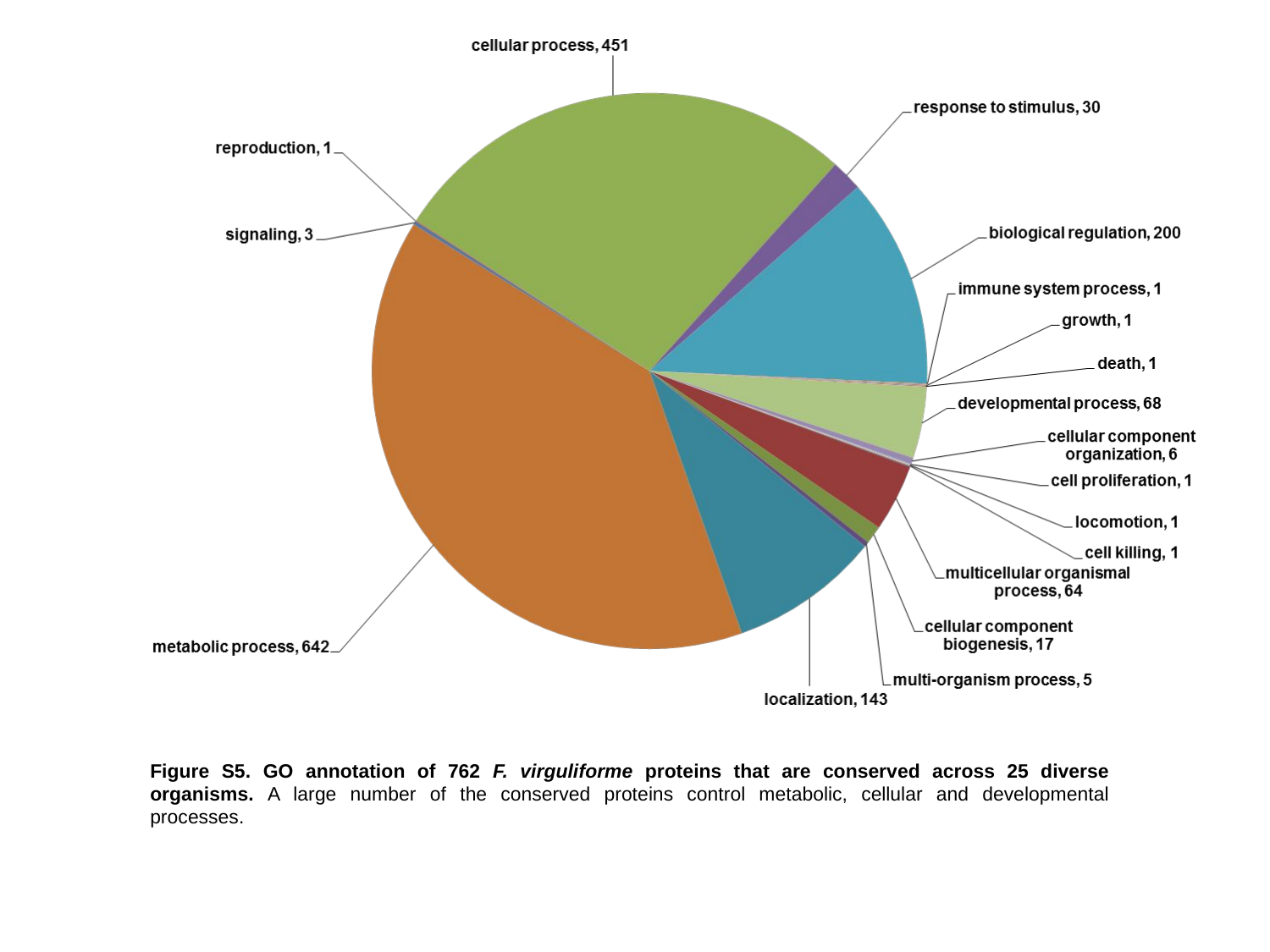

Figure S5. GO annotation of 762 F. virguliforme proteins that are conserved across 25 diverse organisms. A large number of the conserved proteins control metabolic, cellular and developmental processes.
