# Supplementary material for: The Genome Sequence of the Fungal Pathogen Fusarium virguliforme That Causes Sudden Death Syndrome in Soybean
Source: PLoS One. 2014 Jan 14;9(1):e81832. doi: 10.1371/journal.pone.0081832 (PMC3891557; doi:10.1371/journal.pone.0081832)
Supplement: Figure S6 — Unique F. virguliforme genes . There are 11,403 genes that are common to all five Fusarium species and 1,332 genes unique to F. virguliforme at E≤10−9. The numbers in the light blue circle are the number of genes of a species that showed similarity (E≤10−9) to genes of at least another species. (PPT) [file pone.0081832.s006.ppt]

## Slide 1
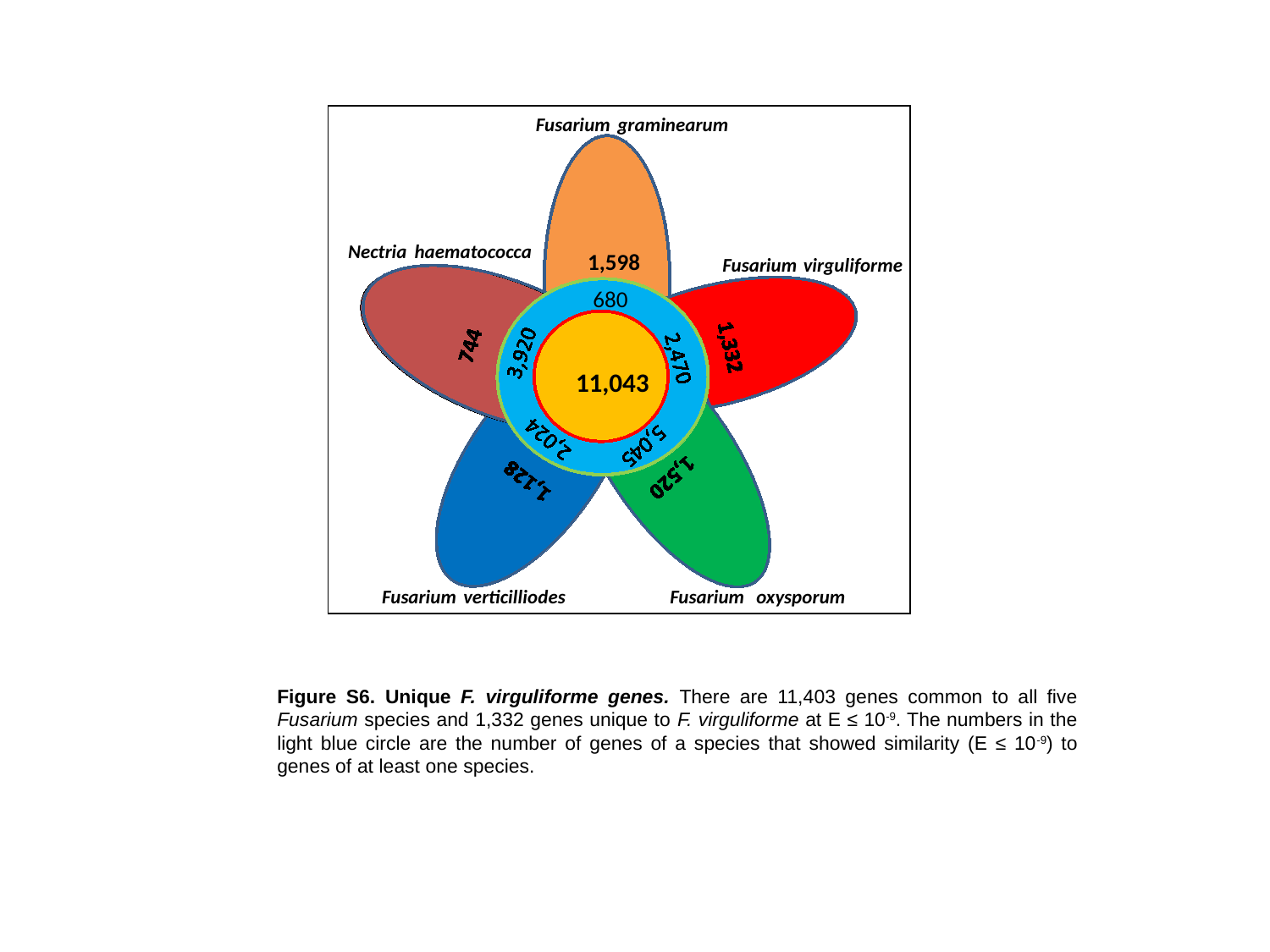

Fusarium
graminearum
Nectria
haematococca
1,598
Fusarium
virguliforme
680
11,043
Fusarium
verticilliodes
Fusarium
oxysporum
Figure S6. Unique F. virguliforme genes. There are 11,403 genes common to all five Fusarium species and 1,332 genes unique to F. virguliforme at E ≤ 10-9. The numbers in the light blue circle are the number of genes of a species that showed similarity (E ≤ 10-9) to genes of at least one species.
