# Supplementary figures and images for: The Genome Sequence of the Fungal Pathogen Fusarium virguliforme That Causes Sudden Death Syndrome in Soybean
Source: PLoS One. 2014 Jan 14;9(1):e81832. doi: 10.1371/journal.pone.0081832 (PMC3891557; doi:10.1371/journal.pone.0081832)

## Slide 1
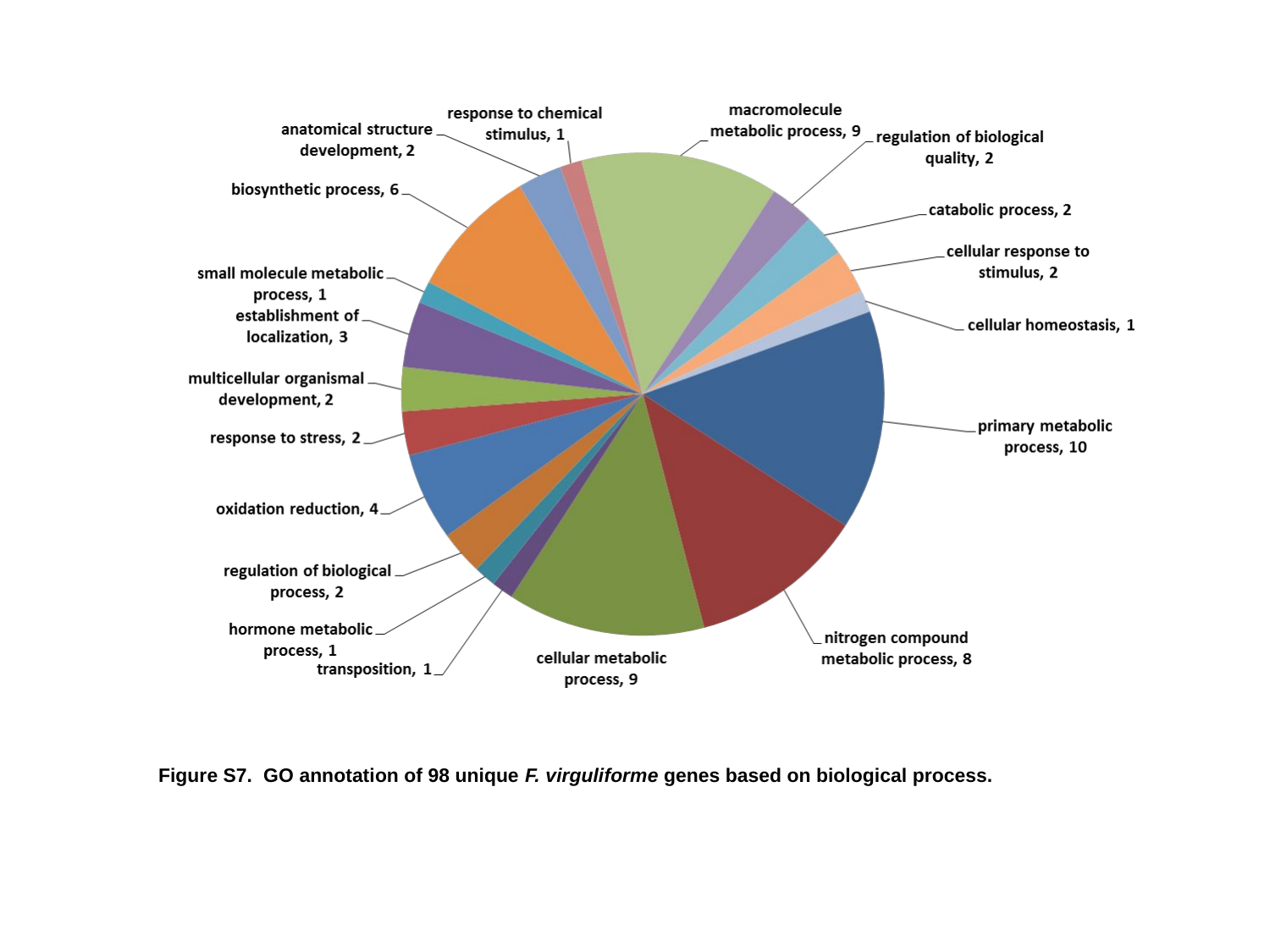

Figure S7. GO annotation of 98 unique F. virguliforme genes based on biological process.

Supplement: Figure S7 — GO annotation of 98 unique F. virguliforme genes based on biological process. (PPT) [file pone.0081832.s007.ppt]

## Slide 1
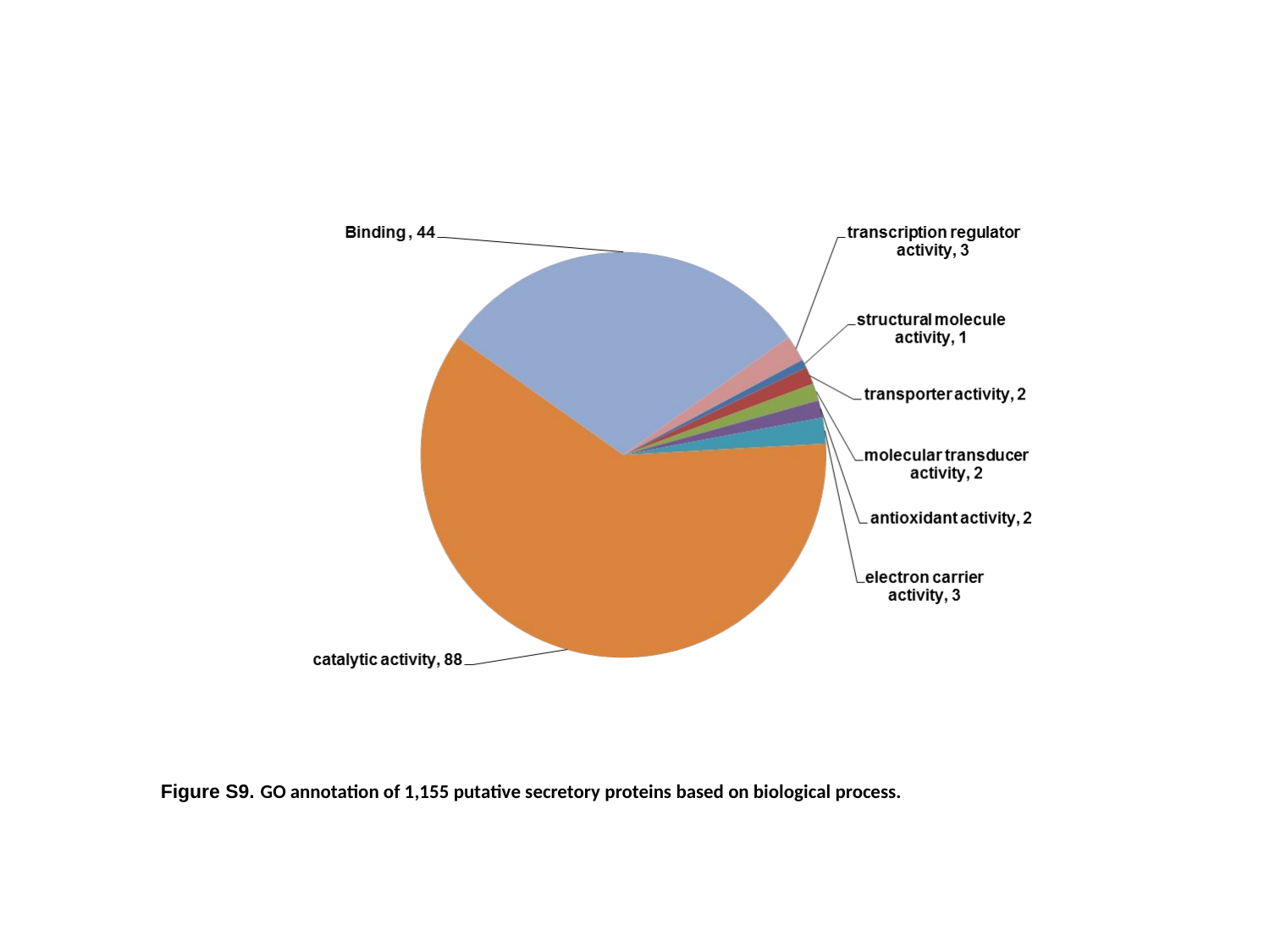

Figure S9. GO annotation of 1,155 putative secretory proteins based on biological process.

Supplement: Figure S9 — GO annotation of 1,155 putative secretory proteins based on biological process. (PPT) [file pone.0081832.s009.ppt]
