# Supplementary material for: The Genome Sequence of the Fungal Pathogen Fusarium virguliforme That Causes Sudden Death Syndrome in Soybean
Source: PLoS One. 2014 Jan 14;9(1):e81832. doi: 10.1371/journal.pone.0081832 (PMC3891557; doi:10.1371/journal.pone.0081832)
Supplement: Table S1 — Alignments of coordinates between N. haematococca and F. virguliforme Mont1 genomes. (DOC) [file pone.0081832.s010.doc]

**Table S1.** Alignments of coordinates between *N. haematococca*  and *F. virguliforme* Mont1 genomes.

| **Reference length** | | **Query length** | | **Reference difference** | | **Query difference** | **% Iden- tity** | ***N. haematococca*  Scafolds** | ***F. virguliforme* Scafolds** |
| --- | --- | --- | --- | --- | --- | --- | --- | --- | --- |
| **Start [S1]** | **End [E1]** | **Start [S2]** | **End [E2]** | | **[LEN 1]** | [LEN 2] |  |  |  |
| 13677 | 15914 | 5014496 | 5012263 | | 2238 | 2234 | 89.14 | sca_1_chr1_3_0 | Scaffold0 |
| 26098 | 29212 | 5001936 | 4998791 | | 3115 | 3146 | 91.77 | sca_1_chr1_3_0 | Scaffold0 |
| 96877 | 99823 | 4931341 | 4928374 | | 2947 | 2968 | 90.8 | sca_1_chr1_3_0 | Scaffold0 |
| 108785 | 111429 | 4919566 | 4916975 | | 2645 | 2592 | 90.02 | sca_1_chr1_3_0 | Scaffold0 |
| 112311 | 115375 | 4916131 | 4913029 | | 3065 | 3103 | 88.95 | sca_1_chr1_3_0 | Scaffold0 |
| 124367 | 128024 | 4904094 | 4900414 | | 3658 | 3681 | 88.3 | sca_1_chr1_3_0 | Scaffold0 |
| 180665 | 182736 | 4848189 | 4846099 | | 2072 | 2091 | 86.63 | sca_1_chr1_3_0 | Scaffold0 |
| 196713 | 199264 | 4832434 | 4829865 | | 2552 | 2570 | 87.34 | sca_1_chr1_3_0 | Scaffold0 |
| 200845 | 203635 | 4828195 | 4825382 | | 2791 | 2814 | 90.5 | sca_1_chr1_3_0 | Scaffold0 |
| 231856 | 238687 | 4797340 | 4790499 | | 6832 | 6842 | 89.29 | sca_1_chr1_3_0 | Scaffold0 |
| 242091 | 245159 | 4787312 | 4784264 | | 3069 | 3049 | 87.66 | sca_1_chr1_3_0 | Scaffold0 |
| 245366 | 248908 | 4784057 | 4780494 | | 3543 | 3564 | 85.35 | sca_1_chr1_3_0 | Scaffold0 |
| 249383 | 249692 | 4779987 | 4779665 | | 310 | 323 | 95.36 | sca_1_chr1_3_0 | Scaffold0 |
| 250104 | 251662 | 4779271 | 4777726 | | 1559 | 1546 | 82.65 | sca_1_chr1_3_0 | Scaffold0 |
| 256389 | 258633 | 4773218 | 4770959 | | 2245 | 2260 | 88.39 | sca_1_chr1_3_0 | Scaffold0 |
| 259493 | 262363 | 4770125 | 4767212 | | 2871 | 2914 | 89.02 | sca_1_chr1_3_0 | Scaffold0 |
| 264362 | 266216 | 4765186 | 4763336 | | 1855 | 1851 | 87.45 | sca_1_chr1_3_0 | Scaffold0 |
| 268502 | 268758 | 4761081 | 4760815 | | 257 | 267 | 87.36 | sca_1_chr1_3_0 | Scaffold0 |
| 300862 | 305782 | 4729908 | 4725024 | | 4921 | 4885 | 89.32 | sca_1_chr1_3_0 | Scaffold0 |
| 361952 | 363691 | 4664679 | 4662936 | | 1740 | 1744 | 89.88 | sca_1_chr1_3_0 | Scaffold0 |
| 426707 | 427223 | 4599239 | 4598745 | | 517 | 495 | 85.28 | sca_1_chr1_3_0 | Scaffold0 |
| 431651 | 432102 | 4594516 | 4594046 | | 452 | 471 | 88.87 | sca_1_chr1_3_0 | Scaffold0 |
| 434229 | 436091 | 4591896 | 4590024 | | 1863 | 1873 | 92.55 | sca_1_chr1_3_0 | Scaffold0 |
| 444024 | 447211 | 4581987 | 4578809 | | 3188 | 3179 | 88.82 | sca_1_chr1_3_0 | Scaffold0 |
| 456351 | 457664 | 4570020 | 4568711 | | 1314 | 1310 | 95.36 | sca_1_chr1_3_0 | Scaffold0 |
| 473193 | 474248 | 4552761 | 4551699 | | 1056 | 1063 | 92 | sca_1_chr1_3_0 | Scaffold0 |
| 514825 | 515140 | 4511052 | 4510729 | | 316 | 324 | 91.16 | sca_1_chr1_3_0 | Scaffold0 |
| 525244 | 532622 | 4500791 | 4493414 | | 7379 | 7378 | 89.73 | sca_1_chr1_3_0 | Scaffold0 |
| 541076 | 541348 | 4484939 | 4484670 | | 273 | 270 | 92.34 | sca_1_chr1_3_0 | Scaffold0 |
| 551421 | 552180 | 4475080 | 4474313 | | 760 | 768 | 84.59 | sca_1_chr1_3_0 | Scaffold0 |
| 600644 | 611005 | 4430049 | 4419679 | | 10362 | 10371 | 88.6 | sca_1_chr1_3_0 | Scaffold0 |
| 628995 | 633823 | 4401688 | 4396831 | | 4829 | 4858 | 90.46 | sca_1_chr1_3_0 | Scaffold0 |
| 656659 | 657453 | 4372062 | 4371280 | | 795 | 783 | 92.03 | sca_1_chr1_3_0 | Scaffold0 |
| 658018 | 664725 | 4370789 | 4364100 | | 6708 | 6690 | 89.26 | sca_1_chr1_3_0 | Scaffold0 |
| 699708 | 701564 | 4329111 | 4327165 | | 1857 | 1947 | 89.51 | sca_1_chr1_3_0 | Scaffold0 |
| 703194 | 704805 | 4325538 | 4323857 | | 1612 | 1682 | 85.56 | sca_1_chr1_3_0 | Scaffold0 |
| 751852 | 753404 | 4279665 | 4278079 | | 1553 | 1587 | 86.1 | sca_1_chr1_3_0 | Scaffold0 |
| 785446 | 789922 | 4247140 | 4242647 | | 4477 | 4494 | 87.99 | sca_1_chr1_3_0 | Scaffold0 |
| 823644 | 828531 | 4205357 | 4200482 | | 4888 | 4876 | 87.36 | sca_1_chr1_3_0 | Scaffold0 |
| 829716 | 830570 | 4199300 | 4198449 | | 855 | 852 | 92.07 | sca_1_chr1_3_0 | Scaffold0 |
| 859460 | 861081 | 4169724 | 4168140 | | 1622 | 1585 | 86.62 | sca_1_chr1_3_0 | Scaffold0 |
| 883134 | 884615 | 4146176 | 4144690 | | 1482 | 1487 | 88.02 | sca_1_chr1_3_0 | Scaffold0 |
| 901712 | 906159 | 4125438 | 4120999 | | 4448 | 4440 | 86.1 | sca_1_chr1_3_0 | Scaffold0 |
| 974895 | 977009 | 3136108 | 3138246 | | 2115 | 2139 | 91.13 | sca_1_chr1_3_0 | Scaffold0 |
| 979923 | 981784 | 3141142 | 3143001 | | 1862 | 1860 | 91.21 | sca_1_chr1_3_0 | Scaffold0 |
| 1015923 | 1018121 | 3181667 | 3179446 | | 2199 | 2222 | 90.77 | sca_1_chr1_3_0 | Scaffold0 |
| 1018610 | 1021311 | 3179041 | 3176357 | | 2702 | 2685 | 90.32 | sca_1_chr1_3_0 | Scaffold0 |
| 1048495 | 1054150 | 3211279 | 3216965 | | 5656 | 5687 | 87.42 | sca_1_chr1_3_0 | Scaffold0 |
| 1102569 | 1104496 | 3265198 | 3267127 | | 1928 | 1930 | 86.41 | sca_1_chr1_3_0 | Scaffold0 |
| 1106886 | 1109663 | 3269569 | 3272338 | | 2778 | 2770 | 85.98 | sca_1_chr1_3_0 | Scaffold0 |
| 1123049 | 1125009 | 3286185 | 3288136 | | 1961 | 1952 | 89.11 | sca_1_chr1_3_0 | Scaffold0 |
| 1170252 | 1177147 | 3332952 | 3339792 | | 6896 | 6841 | 88.44 | sca_1_chr1_3_0 | Scaffold0 |
| 1237069 | 1237332 | 3399139 | 3399403 | | 264 | 265 | 95.85 | sca_1_chr1_3_0 | Scaffold0 |
| 1251855 | 1256162 | 3414031 | 3418370 | | 4308 | 4340 | 90.21 | sca_1_chr1_3_0 | Scaffold0 |
| 1256974 | 1257304 | 3419381 | 3419707 | | 331 | 327 | 88.17 | sca_1_chr1_3_0 | Scaffold0 |
| 1257412 | 1258777 | 3420007 | 3421332 | | 1366 | 1326 | 90 | sca_1_chr1_3_0 | Scaffold0 |
| 1268334 | 1271589 | 3430583 | 3433842 | | 3256 | 3260 | 89.21 | sca_1_chr1_3_0 | Scaffold0 |
| 1354669 | 1359478 | 3517865 | 3522647 | | 4810 | 4783 | 88 | sca_1_chr1_3_0 | Scaffold0 |
| 1459915 | 1463374 | 3625078 | 3628524 | | 3460 | 3447 | 90.62 | sca_1_chr1_3_0 | Scaffold0 |
| 1507915 | 1509071 | 3680278 | 3681431 | | 1157 | 1154 | 89.93 | sca_1_chr1_3_0 | Scaffold0 |
| 1517750 | 1518047 | 3688388 | 3688678 | | 298 | 291 | 89.93 | sca_1_chr1_3_0 | Scaffold0 |
| 1519455 | 1519575 | 3690106 | 3690226 | | 121 | 121 | 99.17 | sca_1_chr1_3_0 | Scaffold0 |
| 1522085 | 1524864 | 3692776 | 3695561 | | 2780 | 2786 | 85.25 | sca_1_chr1_3_0 | Scaffold0 |
| 1537809 | 1537952 | 3706012 | 3706157 | | 144 | 146 | 96.58 | sca_1_chr1_3_0 | Scaffold0 |
| 1621084 | 1624598 | 3785213 | 3788714 | | 3515 | 3502 | 90.59 | sca_1_chr1_3_0 | Scaffold0 |
| 1641328 | 1645184 | 3805039 | 3808914 | | 3857 | 3876 | 88.13 | sca_1_chr1_3_0 | Scaffold0 |
| 1709759 | 1711616 | 3872188 | 3874081 | | 1858 | 1894 | 89.84 | sca_1_chr1_3_0 | Scaffold0 |
| 1722029 | 1724150 | 3884299 | 3886433 | | 2122 | 2135 | 88.01 | sca_1_chr1_3_0 | Scaffold0 |
| 1791131 | 1793650 | 3957724 | 3960251 | | 2520 | 2528 | 92.54 | sca_1_chr1_3_0 | Scaffold0 |
| 1844441 | 1845853 | 4011073 | 4012453 | | 1413 | 1381 | 84.95 | sca_1_chr1_3_0 | Scaffold0 |
| 1860079 | 1860416 | 4026740 | 4027081 | | 338 | 342 | 94.44 | sca_1_chr1_3_0 | Scaffold0 |
| 1875595 | 1875712 | 4041114 | 4041231 | | 118 | 118 | 99.15 | sca_1_chr1_3_0 | Scaffold0 |
| 1875604 | 1875712 | 273400 | 273291 | | 109 | 110 | 99.09 | sca_1_chr1_3_0 | Scaffold20 |
| 1891145 | 1893191 | 3129751 | 3127706 | | 2047 | 2046 | 88.35 | sca_1_chr1_3_0 | Scaffold0 |
| 1904977 | 1908749 | 3115373 | 3111654 | | 3773 | 3720 | 89.51 | sca_1_chr1_3_0 | Scaffold0 |
| 1921495 | 1922025 | 3098923 | 3098410 | | 531 | 514 | 83.36 | sca_1_chr1_3_0 | Scaffold0 |
| 1921758 | 1922025 | 149257 | 149518 | | 268 | 262 | 91.04 | sca_1_chr1_3_0 | Scaffold20 |
| 1925928 | 1928514 | 3094074 | 3091489 | | 2587 | 2586 | 86.41 | sca_1_chr1_3_0 | Scaffold0 |
| 1983105 | 1984315 | 3036824 | 3035591 | | 1211 | 1234 | 86.56 | sca_1_chr1_3_0 | Scaffold0 |
| 2000557 | 2004496 | 3019250 | 3015308 | | 3940 | 3943 | 87.3 | sca_1_chr1_3_0 | Scaffold0 |
| 2006297 | 2006840 | 3013567 | 3013012 | | 544 | 556 | 89.45 | sca_1_chr1_3_0 | Scaffold0 |
| 2026910 | 2028370 | 2992986 | 2991539 | | 1461 | 1448 | 87.68 | sca_1_chr1_3_0 | Scaffold0 |
| 2040176 | 2040899 | 2983319 | 2982596 | | 724 | 724 | 93.09 | sca_1_chr1_3_0 | Scaffold0 |
| 2055458 | 2057202 | 2968150 | 2966402 | | 1745 | 1749 | 93.88 | sca_1_chr1_3_0 | Scaffold0 |
| 2134629 | 2136337 | 2884435 | 2882723 | | 1709 | 1713 | 87.63 | sca_1_chr1_3_0 | Scaffold0 |
| 2152259 | 2152636 | 2866843 | 2866445 | | 378 | 399 | 89.78 | sca_1_chr1_3_0 | Scaffold0 |
| 2157971 | 2158320 | 2861386 | 2861039 | | 350 | 348 | 92.88 | sca_1_chr1_3_0 | Scaffold0 |
| 2161629 | 2162093 | 2857881 | 2857397 | | 465 | 485 | 89.28 | sca_1_chr1_3_0 | Scaffold0 |
| 2182767 | 2184437 | 2837047 | 2835397 | | 1671 | 1651 | 86.41 | sca_1_chr1_3_0 | Scaffold0 |
| 2210915 | 2214900 | 2809365 | 2805370 | | 3986 | 3996 | 88.97 | sca_1_chr1_3_0 | Scaffold0 |
| 2217570 | 2219133 | 2802659 | 2801108 | | 1564 | 1552 | 89.49 | sca_1_chr1_3_0 | Scaffold0 |
| 2267326 | 2268060 | 2753678 | 2752951 | | 735 | 728 | 85.58 | sca_1_chr1_3_0 | Scaffold0 |
| 2275105 | 2279226 | 2745953 | 2741814 | | 4122 | 4140 | 88.3 | sca_1_chr1_3_0 | Scaffold0 |
| 2292796 | 2297030 | 2728093 | 2723891 | | 4235 | 4203 | 89.27 | sca_1_chr1_3_0 | Scaffold0 |
| 2304311 | 2308223 | 2717798 | 2713860 | | 3913 | 3939 | 87.79 | sca_1_chr1_3_0 | Scaffold0 |
| 2403746 | 2406946 | 2628779 | 2625539 | | 3201 | 3241 | 88.02 | sca_1_chr1_3_0 | Scaffold0 |
| 2412842 | 2413380 | 2619868 | 2619315 | | 539 | 554 | 86.37 | sca_1_chr1_3_0 | Scaffold0 |
| 2465162 | 2466678 | 2568390 | 2566882 | | 1517 | 1509 | 90.86 | sca_1_chr1_3_0 | Scaffold0 |
| 2466870 | 2468820 | 2566679 | 2564724 | | 1951 | 1956 | 89.18 | sca_1_chr1_3_0 | Scaffold0 |
| 2534559 | 2535857 | 2499687 | 2498369 | | 1299 | 1319 | 87.22 | sca_1_chr1_3_0 | Scaffold0 |
| 2542123 | 2551081 | 2492084 | 2483137 | | 8959 | 8948 | 88.88 | sca_1_chr1_3_0 | Scaffold0 |
| 2562881 | 2565823 | 2471539 | 2468598 | | 2943 | 2942 | 89.02 | sca_1_chr1_3_0 | Scaffold0 |
| 2624632 | 2626070 | 2417396 | 2418834 | | 1439 | 1439 | 95.07 | sca_1_chr1_3_0 | Scaffold0 |
| 2650265 | 2651495 | 2388186 | 2386971 | | 1231 | 1216 | 87.34 | sca_1_chr1_3_0 | Scaffold0 |
| 2653410 | 2655921 | 2385001 | 2382501 | | 2512 | 2501 | 87.03 | sca_1_chr1_3_0 | Scaffold0 |
| 2700628 | 2703676 | 2338238 | 2335193 | | 3049 | 3046 | 90.54 | sca_1_chr1_3_0 | Scaffold0 |
| 2723155 | 2725541 | 2315576 | 2313208 | | 2387 | 2369 | 91.56 | sca_1_chr1_3_0 | Scaffold0 |
| 2732756 | 2734106 | 2306108 | 2304781 | | 1351 | 1328 | 91.18 | sca_1_chr1_3_0 | Scaffold0 |
| 2743034 | 2748071 | 2295980 | 2290939 | | 5038 | 5042 | 87.06 | sca_1_chr1_3_0 | Scaffold0 |
| 2750023 | 2755065 | 2288822 | 2283784 | | 5043 | 5039 | 87.1 | sca_1_chr1_3_0 | Scaffold0 |
| 2789301 | 2789751 | 2250226 | 2249780 | | 451 | 447 | 84.5 | sca_1_chr1_3_0 | Scaffold0 |
| 2816767 | 2818754 | 2238423 | 2240401 | | 1988 | 1979 | 91.02 | sca_1_chr1_3_0 | Scaffold0 |
| 2849615 | 2849823 | 2182433 | 2182642 | | 209 | 210 | 99.05 | sca_1_chr1_3_0 | Scaffold0 |
| 2974247 | 2975697 | 2068752 | 2070208 | | 1451 | 1457 | 90.68 | sca_1_chr1_3_0 | Scaffold0 |
| 2990361 | 2992310 | 2044480 | 2042531 | | 1950 | 1950 | 87.88 | sca_1_chr1_3_0 | Scaffold0 |
| 3001349 | 3002243 | 2032827 | 2031931 | | 895 | 897 | 91.15 | sca_1_chr1_3_0 | Scaffold0 |
| 3015611 | 3019363 | 2018897 | 2015131 | | 3753 | 3767 | 87.34 | sca_1_chr1_3_0 | Scaffold0 |
| 3109493 | 3112468 | 1899766 | 1902713 | | 2976 | 2948 | 88.46 | sca_1_chr1_3_0 | Scaffold0 |
| 3129291 | 3131950 | 1919074 | 1921734 | | 2660 | 2661 | 89.5 | sca_1_chr1_3_0 | Scaffold0 |
| 3212603 | 3217787 | 1837379 | 1832182 | | 5185 | 5198 | 87.87 | sca_1_chr1_3_0 | Scaffold0 |
| 3286534 | 3287206 | 1764448 | 1763768 | | 673 | 681 | 85.39 | sca_1_chr1_3_0 | Scaffold0 |
| 3363750 | 3369797 | 1625485 | 1631480 | | 6048 | 5996 | 86.5 | sca_1_chr1_3_0 | Scaffold0 |
| 3457955 | 3460056 | 1581461 | 1579380 | | 2102 | 2082 | 89.01 | sca_1_chr1_3_0 | Scaffold0 |
| 3460184 | 3462291 | 1579252 | 1577136 | | 2108 | 2117 | 90.67 | sca_1_chr1_3_0 | Scaffold0 |
| 3521091 | 3524382 | 1520208 | 1516939 | | 3292 | 3270 | 88.89 | sca_1_chr1_3_0 | Scaffold0 |
| 3533291 | 3536652 | 1508858 | 1505416 | | 3362 | 3443 | 89.16 | sca_1_chr1_3_0 | Scaffold0 |
| 3562153 | 3565202 | 1479822 | 1476791 | | 3050 | 3032 | 87.01 | sca_1_chr1_3_0 | Scaffold0 |
| 3574859 | 3575834 | 1467482 | 1466550 | | 976 | 933 | 87.11 | sca_1_chr1_3_0 | Scaffold0 |
| 3595679 | 3596145 | 1446888 | 1446425 | | 467 | 464 | 91.91 | sca_1_chr1_3_0 | Scaffold0 |
| 3737512 | 3741011 | 1312562 | 1309063 | | 3500 | 3500 | 88.82 | sca_1_chr1_3_0 | Scaffold0 |
| 3791115 | 3791857 | 1258021 | 1258742 | | 743 | 722 | 89.01 | sca_1_chr1_3_0 | Scaffold0 |
| 3850073 | 3855411 | 1207831 | 1202491 | | 5339 | 5341 | 86.77 | sca_1_chr1_3_0 | Scaffold0 |
| 3980437 | 3980599 | 1081450 | 1081297 | | 163 | 154 | 93.25 | sca_1_chr1_3_0 | Scaffold0 |
| 4117661 | 4118391 | 952820 | 952098 | | 731 | 723 | 87.63 | sca_1_chr1_3_0 | Scaffold0 |
| 4119432 | 4122215 | 951077 | 948307 | | 2784 | 2771 | 86.75 | sca_1_chr1_3_0 | Scaffold0 |
| 4253587 | 4254088 | 809524 | 809026 | | 502 | 499 | 90.62 | sca_1_chr1_3_0 | Scaffold0 |
| 4356420 | 4361115 | 695286 | 690576 | | 4696 | 4711 | 86.82 | sca_1_chr1_3_0 | Scaffold0 |
| 4368225 | 4369419 | 683398 | 682279 | | 1195 | 1120 | 85.81 | sca_1_chr1_3_0 | Scaffold0 |
| 4706187 | 4710787 | 388581 | 383920 | | 4601 | 4662 | 87.8 | sca_1_chr1_3_0 | Scaffold0 |
| 4720975 | 4724270 | 373455 | 370145 | | 3296 | 3311 | 88.85 | sca_1_chr1_3_0 | Scaffold0 |
|  |  |  |  | |  |  |  |  |  |
| 102819 | 105539 | 631334 | 634084 | | 2721 | 2751 | 88.76 | sca_10_chr8_2_0 | Scaffold19 |
| 222391 | 226033 | 1021206 | 1017554 | | 3643 | 3653 | 89.19 | sca_10_chr8_2_0 | Scaffold13 |
| 278191 | 281514 | 966625 | 963281 | | 3324 | 3345 | 87.76 | sca_10_chr8_2_0 | Scaffold13 |
| 294459 | 298139 | 950171 | 946429 | | 3681 | 3743 | 88.55 | sca_10_chr8_2_0 | Scaffold13 |
| 307699 | 309817 | 936947 | 934824 | | 2119 | 2124 | 87.05 | sca_10_chr8_2_0 | Scaffold13 |
| 334381 | 334775 | 910641 | 910244 | | 395 | 398 | 85.37 | sca_10_chr8_2_0 | Scaffold13 |
| 355235 | 364449 | 890169 | 880931 | | 9215 | 9239 | 89.83 | sca_10_chr8_2_0 | Scaffold13 |
| 414808 | 417089 | 842150 | 839899 | | 2282 | 2252 | 93.3 | sca_10_chr8_2_0 | Scaffold13 |
| 417513 | 419835 | 839479 | 837164 | | 2323 | 2316 | 88.2 | sca_10_chr8_2_0 | Scaffold13 |
| 445070 | 446090 | 810988 | 809963 | | 1021 | 1026 | 89.42 | sca_10_chr8_2_0 | Scaffold13 |
| 448013 | 449933 | 807972 | 806060 | | 1921 | 1913 | 90.6 | sca_10_chr8_2_0 | Scaffold13 |
| 501614 | 504020 | 753544 | 751160 | | 2407 | 2385 | 87.94 | sca_10_chr8_2_0 | Scaffold13 |
| 541855 | 541981 | 111425 | 111551 | | 127 | 127 | 99.21 | sca_10_chr8_2_0 | Scaffold38 |
| 541858 | 541985 | 1794498 | 1794625 | | 128 | 128 | 99.22 | sca_10_chr8_2_0 | Scaffold3 |
| 541859 | 541980 | 500367 | 500488 | | 122 | 122 | 100 | sca_10_chr8_2_0 | Scaffold15 |
| 592685 | 594399 | 667690 | 665974 | | 1715 | 1717 | 87.99 | sca_10_chr8_2_0 | Scaffold13 |
| 613853 | 618786 | 646705 | 641783 | | 4934 | 4923 | 86.62 | sca_10_chr8_2_0 | Scaffold13 |
| 631612 | 633471 | 629115 | 627262 | | 1860 | 1854 | 89.52 | sca_10_chr8_2_0 | Scaffold13 |
| 648367 | 649792 | 612501 | 611129 | | 1426 | 1373 | 87.39 | sca_10_chr8_2_0 | Scaffold13 |
| 686810 | 688928 | 572468 | 570338 | | 2119 | 2131 | 90.76 | sca_10_chr8_2_0 | Scaffold13 |
| 718967 | 719461 | 540027 | 539523 | | 495 | 505 | 91.05 | sca_10_chr8_2_0 | Scaffold13 |
| 808272 | 810530 | 451696 | 449435 | | 2259 | 2262 | 89.68 | sca_10_chr8_2_0 | Scaffold13 |
| 830692 | 832190 | 429543 | 428041 | | 1499 | 1503 | 89.85 | sca_10_chr8_2_0 | Scaffold13 |
| 832402 | 834284 | 427868 | 425943 | | 1883 | 1926 | 85.01 | sca_10_chr8_2_0 | Scaffold13 |
| 836473 | 840281 | 423940 | 420118 | | 3809 | 3823 | 87.11 | sca_10_chr8_2_0 | Scaffold13 |
| 842962 | 843860 | 417494 | 416623 | | 899 | 872 | 83.53 | sca_10_chr8_2_0 | Scaffold13 |
| 848993 | 849907 | 411472 | 410562 | | 915 | 911 | 89.47 | sca_10_chr8_2_0 | Scaffold13 |
| 855162 | 856226 | 405334 | 404282 | | 1065 | 1053 | 89.05 | sca_10_chr8_2_0 | Scaffold13 |
| 871788 | 875395 | 390542 | 386902 | | 3608 | 3641 | 89.6 | sca_10_chr8_2_0 | Scaffold13 |
| 878028 | 880988 | 384366 | 381413 | | 2961 | 2954 | 89.28 | sca_10_chr8_2_0 | Scaffold13 |
| 980056 | 988864 | 278873 | 270023 | | 8809 | 8851 | 87.96 | sca_10_chr8_2_0 | Scaffold13 |
| 1012398 | 1016306 | 247157 | 243247 | | 3909 | 3911 | 91.66 | sca_10_chr8_2_0 | Scaffold13 |
| 1051226 | 1051973 | 208901 | 208154 | | 748 | 748 | 96.66 | sca_10_chr8_2_0 | Scaffold13 |
| 1059475 | 1062491 | 192983 | 189919 | | 3017 | 3065 | 91.09 | sca_10_chr8_2_0 | Scaffold13 |
| 1064114 | 1065192 | 188332 | 187229 | | 1079 | 1104 | 90.67 | sca_10_chr8_2_0 | Scaffold13 |
| 1073213 | 1074342 | 183385 | 182260 | | 1130 | 1126 | 88.33 | sca_10_chr8_2_0 | Scaffold13 |
| 1080195 | 1085388 | 176500 | 171341 | | 5194 | 5160 | 87.77 | sca_10_chr8_2_0 | Scaffold13 |
| 1093522 | 1096476 | 163450 | 160485 | | 2955 | 2966 | 88.56 | sca_10_chr8_2_0 | Scaffold13 |
| 1116950 | 1120367 | 140080 | 136724 | | 3418 | 3357 | 88.45 | sca_10_chr8_2_0 | Scaffold13 |
| 1130850 | 1132160 | 126487 | 125149 | | 1311 | 1339 | 89.72 | sca_10_chr8_2_0 | Scaffold13 |
| 1168981 | 1173092 | 88412 | 84309 | | 4112 | 4104 | 92.12 | sca_10_chr8_2_0 | Scaffold13 |
| 1173235 | 1174584 | 84114 | 82767 | | 1350 | 1348 | 91.07 | sca_10_chr8_2_0 | Scaffold13 |
| 1177649 | 1182900 | 79734 | 74490 | | 5252 | 5245 | 89.91 | sca_10_chr8_2_0 | Scaffold13 |
| 1197850 | 1198784 | 60096 | 59167 | | 935 | 930 | 93.16 | sca_10_chr8_2_0 | Scaffold13 |
| 1241424 | 1245597 | 24020 | 19853 | | 4174 | 4168 | 89.26 | sca_10_chr8_2_0 | Scaffold13 |
|  |  |  |  | |  |  |  |  |  |
| 30830 | 32548 | 19202 | 17479 | | 1719 | 1724 | 96.3 | sca_101_unmapped | Scaffold72 |
| 32627 | 33393 | 17275 | 16502 | | 767 | 774 | 96.51 | sca_101_unmapped | Scaffold72 |
| 1 | 110 | 153947 | 153838 | | 110 | 110 | 100 | sca_102_unmapped | Scaffold26 |
| 1 | 110 | 181564 | 181673 | | 110 | 110 | 100 | sca_102_unmapped | Scaffold44 |
| 1 | 120 | 44549 | 44429 | | 120 | 121 | 98.35 | sca_102_unmapped | Scaffold77 |
| 1 | 110 | 38914 | 38805 | | 110 | 110 | 100 | sca_102_unmapped | Scaffold82 |
| 11727 | 11917 | 136782 | 136588 | | 191 | 195 | 96.92 | sca_103_unmapped | Scaffold41 |
| 12035 | 15118 | 2242 | 5328 | | 3084 | 3087 | 95.21 | sca_103_unmapped | Scaffold104 |
| 9328 | 12132 | 47841 | 50635 | | 2805 | 2795 | 90.15 | sca_104_unmapped | Scaffold52 |
|  |  |  |  | |  |  |  |  |  |
| 17230 | 18638 | 1267260 | 1265847 | | 1409 | 1414 | 90.41 | sca_11_chr8_3_0 | Scaffold9 |
| 30281 | 32422 | 1254283 | 1252220 | | 2142 | 2064 | 88.27 | sca_11_chr8_3_0 | Scaffold9 |
| 93801 | 97368 | 1174601 | 1171041 | | 3568 | 3561 | 93.42 | sca_11_chr8_3_0 | Scaffold9 |
| 111156 | 112128 | 1158767 | 1157793 | | 973 | 975 | 95.9 | sca_11_chr8_3_0 | Scaffold9 |
| 112788 | 114020 | 1156992 | 1155758 | | 1233 | 1235 | 92.83 | sca_11_chr8_3_0 | Scaffold9 |
| 118576 | 122171 | 1150982 | 1147384 | | 3596 | 3599 | 89.54 | sca_11_chr8_3_0 | Scaffold9 |
| 149062 | 151898 | 1119754 | 1116907 | | 2837 | 2848 | 84.65 | sca_11_chr8_3_0 | Scaffold9 |
| 155502 | 157516 | 1113285 | 1111274 | | 2015 | 2012 | 85.76 | sca_11_chr8_3_0 | Scaffold9 |
| 162147 | 167632 | 1106616 | 1101159 | | 5486 | 5458 | 87.3 | sca_11_chr8_3_0 | Scaffold9 |
| 218844 | 222654 | 1050110 | 1046322 | | 3811 | 3789 | 87.2 | sca_11_chr8_3_0 | Scaffold9 |
| 275132 | 275642 | 994548 | 994029 | | 511 | 520 | 91.43 | sca_11_chr8_3_0 | Scaffold9 |
| 296497 | 298418 | 974426 | 972487 | | 1922 | 1940 | 86.94 | sca_11_chr8_3_0 | Scaffold9 |
| 303936 | 305882 | 966996 | 965061 | | 1947 | 1936 | 88.44 | sca_11_chr8_3_0 | Scaffold9 |
| 404519 | 408535 | 758049 | 753951 | | 4017 | 4099 | 86.46 | sca_11_chr8_3_0 | Scaffold9 |
| 419514 | 421755 | 743024 | 740779 | | 2242 | 2246 | 87.62 | sca_11_chr8_3_0 | Scaffold9 |
| 454691 | 456840 | 701798 | 699674 | | 2150 | 2125 | 91.58 | sca_11_chr8_3_0 | Scaffold9 |
| 465534 | 465660 | 1343419 | 1343545 | | 127 | 127 | 100 | sca_11_chr8_3_0 | Scaffold3 |
| 543103 | 544913 | 642455 | 640634 | | 1811 | 1822 | 83.9 | sca_11_chr8_3_0 | Scaffold9 |
| 557257 | 559232 | 629372 | 627412 | | 1976 | 1961 | 85.51 | sca_11_chr8_3_0 | Scaffold9 |
| 560671 | 563778 | 625981 | 622850 | | 3108 | 3132 | 86.35 | sca_11_chr8_3_0 | Scaffold9 |
| 573175 | 577308 | 616672 | 612531 | | 4134 | 4142 | 91.14 | sca_11_chr8_3_0 | Scaffold9 |
| 754835 | 756306 | 329501 | 330991 | | 1472 | 1491 | 91.23 | sca_11_chr8_3_0 | Scaffold9 |
| 798517 | 799508 | 372552 | 373534 | | 992 | 983 | 87.26 | sca_11_chr8_3_0 | Scaffold9 |
| 822374 | 823034 | 396412 | 397077 | | 661 | 666 | 89.64 | sca_11_chr8_3_0 | Scaffold9 |
| 830797 | 831190 | 406568 | 406969 | | 394 | 402 | 86.27 | sca_11_chr8_3_0 | Scaffold9 |
| 847014 | 848443 | 422767 | 424213 | | 1430 | 1447 | 89.48 | sca_11_chr8_3_0 | Scaffold9 |
| 854990 | 856634 | 430531 | 432172 | | 1645 | 1642 | 92.08 | sca_11_chr8_3_0 | Scaffold9 |
| 1152332 | 1154722 | 116106 | 113725 | | 2391 | 2382 | 87.04 | sca_11_chr8_3_0 | Scaffold9 |
|  |  |  |  | |  |  |  |  |  |
| 33092 | 39028 | 3587 | 9506 | | 5937 | 5920 | 95.9 | sca_112_unmapped | Scaffold18 |
| 39742 | 42666 | 40607 | 37672 | | 2925 | 2936 | 92.58 | sca_112_unmapped | Scaffold60 |
| 6129 | 10954 | 14606 | 9798 | | 4826 | 4809 | 96.69 | sca_114_unmapped | Scaffold137 |
| 12960 | 19051 | 50586 | 44510 | | 6092 | 6077 | 95.62 | sca_114_unmapped | Scaffold63 |
| 7982 | 13551 | 52268 | 46723 | | 5570 | 5546 | 91.37 | sca_117_unmapped | Scaffold57 |
| 14012 | 18071 | 46205 | 42148 | | 4060 | 4058 | 92.03 | sca_117_unmapped | Scaffold57 |
| 8484 | 11852 | 75847 | 72412 | | 3369 | 3436 | 92.54 | sca_118_unmapped | Scaffold28 |
| 26014 | 26532 | 119984 | 120502 | | 519 | 519 | 94.99 | sca_118_unmapped | Scaffold54 |
| 1 | 948 | 15356 | 16301 | | 948 | 946 | 83.95 | sca_119_unmapped | Scaffold18 |
|  |  |  |  | |  |  |  |  |  |
| 175401 | 176799 | 83505 | 84909 | | 1399 | 1405 | 90.91 | sca_12_chr5_2_0 | Scaffold2 |
| 186072 | 186485 | 94172 | 94594 | | 414 | 423 | 92.25 | sca_12_chr5_2_0 | Scaffold2 |
| 248912 | 249881 | 154583 | 155513 | | 970 | 931 | 88.08 | sca_12_chr5_2_0 | Scaffold2 |
| 250002 | 251045 | 155621 | 156631 | | 1044 | 1011 | 92.18 | sca_12_chr5_2_0 | Scaffold2 |
| 251805 | 253756 | 157334 | 159288 | | 1952 | 1955 | 92.88 | sca_12_chr5_2_0 | Scaffold2 |
| 377318 | 377589 | 283047 | 283316 | | 272 | 270 | 85.45 | sca_12_chr5_2_0 | Scaffold2 |
| 407852 | 412041 | 314961 | 319127 | | 4190 | 4167 | 89.55 | sca_12_chr5_2_0 | Scaffold2 |
| 429080 | 429858 | 335720 | 336500 | | 779 | 781 | 92.34 | sca_12_chr5_2_0 | Scaffold2 |
| 432346 | 433817 | 339150 | 340566 | | 1472 | 1417 | 86.77 | sca_12_chr5_2_0 | Scaffold2 |
| 437509 | 440105 | 344540 | 347056 | | 2597 | 2517 | 86.03 | sca_12_chr5_2_0 | Scaffold2 |
| 447200 | 448379 | 353875 | 355061 | | 1180 | 1187 | 90.1 | sca_12_chr5_2_0 | Scaffold2 |
| 523758 | 528529 | 415624 | 420336 | | 4772 | 4713 | 89.78 | sca_12_chr5_2_0 | Scaffold2 |
| 538559 | 540474 | 429936 | 431841 | | 1916 | 1906 | 90.86 | sca_12_chr5_2_0 | Scaffold2 |
| 551126 | 551855 | 441805 | 442529 | | 730 | 725 | 87.88 | sca_12_chr5_2_0 | Scaffold2 |
| 555608 | 558754 | 446163 | 449281 | | 3147 | 3119 | 87.2 | sca_12_chr5_2_0 | Scaffold2 |
| 569025 | 570656 | 459335 | 460914 | | 1632 | 1580 | 88.9 | sca_12_chr5_2_0 | Scaffold2 |
| 621149 | 622987 | 512636 | 514510 | | 1839 | 1875 | 88.02 | sca_12_chr5_2_0 | Scaffold2 |
| 639340 | 640484 | 530060 | 531265 | | 1145 | 1206 | 84.65 | sca_12_chr5_2_0 | Scaffold2 |
| 647850 | 649393 | 538462 | 540008 | | 1544 | 1547 | 88.08 | sca_12_chr5_2_0 | Scaffold2 |
| 694218 | 694979 | 584201 | 584964 | | 762 | 764 | 90.32 | sca_12_chr5_2_0 | Scaffold2 |
| 695867 | 699982 | 585838 | 589970 | | 4116 | 4133 | 86.54 | sca_12_chr5_2_0 | Scaffold2 |
| 720372 | 726280 | 608785 | 614680 | | 5909 | 5896 | 87.58 | sca_12_chr5_2_0 | Scaffold2 |
| 732768 | 734636 | 621037 | 622903 | | 1869 | 1867 | 95.57 | sca_12_chr5_2_0 | Scaffold2 |
| 734838 | 735914 | 623084 | 624173 | | 1077 | 1090 | 92.79 | sca_12_chr5_2_0 | Scaffold2 |
| 782232 | 782944 | 669401 | 670116 | | 713 | 716 | 88.2 | sca_12_chr5_2_0 | Scaffold2 |
| 833769 | 834341 | 719403 | 719975 | | 573 | 573 | 93.48 | sca_12_chr5_2_0 | Scaffold2 |
| 842506 | 842707 | 727880 | 728092 | | 202 | 213 | 90.19 | sca_12_chr5_2_0 | Scaffold2 |
| 861767 | 862267 | 746689 | 747190 | | 501 | 502 | 93.43 | sca_12_chr5_2_0 | Scaffold2 |
| 948928 | 949056 | 4753450 | 4753322 | | 129 | 129 | 100 | sca_12_chr5_2_0 | Scaffold0 |
| 965669 | 966062 | 859010 | 859404 | | 394 | 395 | 91.96 | sca_12_chr5_2_0 | Scaffold2 |
| 1120437 | 1124598 | 1008702 | 1012828 | | 4162 | 4127 | 86.75 | sca_12_chr5_2_0 | Scaffold2 |
| 1139896 | 1142098 | 1028183 | 1030402 | | 2203 | 2220 | 90.24 | sca_12_chr5_2_0 | Scaffold2 |
| 1145211 | 1148793 | 1033730 | 1037319 | | 3583 | 3590 | 87.62 | sca_12_chr5_2_0 | Scaffold2 |
|  |  |  |  | |  |  |  |  |  |
| 15515 | 23297 | 1105382 | 1097632 | | 7783 | 7751 | 97.52 | sca_122_unmapped | Scaffold13 |
| 25509 | 26682 | 1097632 | 1096464 | | 1174 | 1169 | 98.64 | sca_122_unmapped | Scaffold13 |
| 29450 | 29981 | 1096464 | 1095932 | | 532 | 533 | 98.69 | sca_122_unmapped | Scaffold13 |
| 14348 | 14580 | 51608 | 51840 | | 233 | 233 | 95.28 | sca_124_unmapped | Scaffold53 |
| 22959 | 24187 | 127310 | 126083 | | 1229 | 1228 | 83.86 | sca_127_unmapped | Scaffold34 |
| 15432 | 16715 | 5 | 1292 | | 1284 | 1288 | 82.54 | sca_130_unmapped | Scaffold85 |
| 6342 | 9111 | 16146 | 18902 | | 2770 | 2757 | 94.31 | sca_137_unmapped | Scaffold60 |
| 17226 | 20635 | 108011 | 111421 | | 3410 | 3411 | 95.19 | sca_137_unmapped | Scaffold50 |
|  |  |  |  | |  |  |  |  |  |
| 8386 | 14306 | 979506 | 973567 | | 5921 | 5940 | 86.72 | sca_14_chr10_3_0 | Scaffold5 |
| 16422 | 16744 | 971473 | 971139 | | 323 | 335 | 94.63 | sca_14_chr10_3_0 | Scaffold5 |
| 20685 | 20888 | 967012 | 966810 | | 204 | 203 | 94.15 | sca_14_chr10_3_0 | Scaffold5 |
| 36653 | 39189 | 950753 | 948220 | | 2537 | 2534 | 89.99 | sca_14_chr10_3_0 | Scaffold5 |
| 80803 | 82742 | 910419 | 908479 | | 1940 | 1941 | 90 | sca_14_chr10_3_0 | Scaffold5 |
| 159773 | 161270 | 832937 | 831442 | | 1498 | 1496 | 88.97 | sca_14_chr10_3_0 | Scaffold5 |
| 251119 | 254910 | 726688 | 722895 | | 3792 | 3794 | 91.21 | sca_14_chr10_3_0 | Scaffold5 |
| 530706 | 533750 | 453806 | 456808 | | 3045 | 3003 | 92.37 | sca_14_chr10_3_0 | Scaffold5 |
| 557010 | 563286 | 477845 | 484118 | | 6277 | 6274 | 88.76 | sca_14_chr10_3_0 | Scaffold5 |
| 563653 | 563755 | 435862 | 435760 | | 103 | 103 | 100 | sca_14_chr10_3_0 | Scaffold5 |
| 594034 | 594194 | 411941 | 411784 | | 161 | 158 | 97.52 | sca_14_chr10_3_0 | Scaffold5 |
| 683770 | 687499 | 319092 | 315362 | | 3730 | 3731 | 89.25 | sca_14_chr10_3_0 | Scaffold5 |
| 845706 | 847722 | 158575 | 156539 | | 2017 | 2037 | 91.97 | sca_14_chr10_3_0 | Scaffold5 |
|  |  |  |  | |  |  |  |  |  |
| 6909 | 9609 | 25197 | 22497 | | 2701 | 2701 | 96.05 | sca_148_unmapped | Scaffold106 |
| 9609 | 12028 | 22153 | 19748 | | 2420 | 2406 | 96.69 | sca_148_unmapped | Scaffold106 |
| 12201 | 14719 | 19614 | 17086 | | 2519 | 2529 | 95.61 | sca_148_unmapped | Scaffold106 |
| 15646 | 20881 | 15347 | 10245 | | 5236 | 5103 | 93.84 | sca_148_unmapped | Scaffold106 |
| 1 | 278 | 284 | 8 | | 278 | 277 | 93.91 | sca_161_unmapped | Scaffold24 |
| 1 | 621 | 4046 | 4666 | | 621 | 621 | 87.78 | sca_161_unmapped | Scaffold289 |
| 11422 | 14135 | 14156 | 11450 | | 2714 | 2707 | 97.86 | sca_161_unmapped | Scaffold100 |
| 13589 | 14199 | 4897 | 5500 | | 611 | 604 | 93.78 | sca_161_unmapped | Scaffold56 |
| 14135 | 14997 | 11293 | 10434 | | 863 | 860 | 96.76 | sca_161_unmapped | Scaffold100 |
| 11247 | 16797 | 125981 | 120412 | | 5551 | 5570 | 94.76 | sca_164_unmapped | Scaffold41 |
|  |  |  |  | |  |  |  |  |  |
| 318294 | 320322 | 69110 | 71160 | | 2029 | 2051 | 88.67 | sca_17_chr8_1_0 | Scaffold19 |
| 399347 | 400208 | 128527 | 129388 | | 862 | 862 | 91.53 | sca_17_chr8_1_0 | Scaffold19 |
| 449291 | 451276 | 175604 | 177550 | | 1986 | 1947 | 85.93 | sca_17_chr8_1_0 | Scaffold19 |
| 550192 | 552640 | 266663 | 269119 | | 2449 | 2457 | 92.22 | sca_17_chr8_1_0 | Scaffold19 |
| 568841 | 572161 | 284744 | 288145 | | 3321 | 3402 | 86.72 | sca_17_chr8_1_0 | Scaffold19 |
| 793834 | 793962 | 324962 | 325090 | | 129 | 129 | 97.69 | sca_17_chr8_1_0 | Scaffold11 |
| 793861 | 793962 | 119468 | 119569 | | 102 | 102 | 100 | sca_17_chr8_1_0 | Scaffold25 |
| 806260 | 811256 | 465515 | 470503 | | 4997 | 4989 | 89.96 | sca_17_chr8_1_0 | Scaffold19 |
|  |  |  |  | |  |  |  |  |  |
| 13154 | 14489 | 85715 | 84378 | | 1336 | 1338 | 91.56 | sca_171_unmapped | Scaffold58 |
| 2811 | 2915 | 153759 | 153863 | | 105 | 105 | 99.05 | sca_173_unmapped | Scaffold48 |
| 1 | 976 | 51907 | 52886 | | 976 | 980 | 96.43 | sca_174_unmapped | Scaffold22 |
| 5157 | 5425 | 2346 | 2613 | | 269 | 268 | 97.77 | sca_175_unmapped | Scaffold127 |
| 338 | 1427 | 179682 | 180770 | | 1090 | 1089 | 83.56 | sca_179_unmapped | Scaffold26 |
| 4837 | 5943 | 2620 | 1514 | | 1107 | 1107 | 87.08 | sca_179_unmapped | Scaffold106 |
|  |  |  |  | |  |  |  |  |  |
| 30703 | 32852 | 365757 | 367899 | | 2150 | 2143 | 89.02 | sca_18_chr2_1_0 | Scaffold24 |
| 63976 | 66199 | 399442 | 401656 | | 2224 | 2215 | 89.59 | sca_18_chr2_1_0 | Scaffold24 |
| 394893 | 395833 | 100054 | 99114 | | 941 | 941 | 93.3 | sca_18_chr2_1_0 | Scaffold24 |
| 718734 | 719916 | 1205256 | 1204099 | | 1183 | 1158 | 84.7 | sca_18_chr2_1_0 | Scaffold8 |
| 722602 | 724008 | 1201418 | 1199988 | | 1407 | 1431 | 92.05 | sca_18_chr2_1_0 | Scaffold8 |
| 1051492 | 1052958 | 940575 | 939126 | | 1467 | 1450 | 91.01 | sca_18_chr2_1_0 | Scaffold8 |
| 1073721 | 1078476 | 918952 | 914243 | | 4756 | 4710 | 88.77 | sca_18_chr2_1_0 | Scaffold8 |
| 1110650 | 1113120 | 881218 | 878735 | | 2471 | 2484 | 88.79 | sca_18_chr2_1_0 | Scaffold8 |
| 1175376 | 1177875 | 814143 | 811654 | | 2500 | 2490 | 89.84 | sca_18_chr2_1_0 | Scaffold8 |
| 1339304 | 1341034 | 653789 | 652068 | | 1731 | 1722 | 90.87 | sca_18_chr2_1_0 | Scaffold8 |
| 1361194 | 1365233 | 632804 | 628734 | | 4040 | 4071 | 88 | sca_18_chr2_1_0 | Scaffold8 |
| 1409996 | 1413281 | 584352 | 581080 | | 3286 | 3273 | 86.3 | sca_18_chr2_1_0 | Scaffold8 |
| 1419208 | 1424007 | 575266 | 570467 | | 4800 | 4800 | 87.5 | sca_18_chr2_1_0 | Scaffold8 |
| 1448035 | 1452402 | 547393 | 543023 | | 4368 | 4371 | 88.77 | sca_18_chr2_1_0 | Scaffold8 |
| 1455038 | 1458871 | 540372 | 536519 | | 3834 | 3854 | 89.57 | sca_18_chr2_1_0 | Scaffold8 |
| 1461929 | 1465938 | 533469 | 529443 | | 4010 | 4027 | 91.87 | sca_18_chr2_1_0 | Scaffold8 |
| 1509033 | 1515600 | 489709 | 483082 | | 6568 | 6628 | 89.45 | sca_18_chr2_1_0 | Scaffold8 |
| 1522806 | 1524156 | 475795 | 474481 | | 1351 | 1315 | 85.55 | sca_18_chr2_1_0 | Scaffold8 |
| 1551069 | 1555290 | 447784 | 443571 | | 4222 | 4214 | 88.27 | sca_18_chr2_1_0 | Scaffold8 |
| 1559156 | 1561981 | 439731 | 436912 | | 2826 | 2820 | 92.79 | sca_18_chr2_1_0 | Scaffold8 |
| 1590891 | 1591342 | 408978 | 408523 | | 452 | 456 | 92.27 | sca_18_chr2_1_0 | Scaffold8 |
| 1593004 | 1593808 | 406738 | 405937 | | 805 | 802 | 91.78 | sca_18_chr2_1_0 | Scaffold8 |
| 1604400 | 1605958 | 395153 | 393586 | | 1559 | 1568 | 91.2 | sca_18_chr2_1_0 | Scaffold8 |
| 1614278 | 1616798 | 385369 | 382819 | | 2521 | 2551 | 86.33 | sca_18_chr2_1_0 | Scaffold8 |
| 1642523 | 1643789 | 358094 | 356776 | | 1267 | 1319 | 85.04 | sca_18_chr2_1_0 | Scaffold8 |
| 1657050 | 1658476 | 343607 | 342189 | | 1427 | 1419 | 89.36 | sca_18_chr2_1_0 | Scaffold8 |
| 1674983 | 1676166 | 325815 | 324635 | | 1184 | 1181 | 88.75 | sca_18_chr2_1_0 | Scaffold8 |
| 1678055 | 1678746 | 321844 | 321162 | | 692 | 683 | 87.43 | sca_18_chr2_1_0 | Scaffold8 |
| 1722188 | 1727651 | 278107 | 272645 | | 5464 | 5463 | 87.88 | sca_18_chr2_1_0 | Scaffold8 |
| 1734326 | 1736346 | 265859 | 263822 | | 2021 | 2038 | 89.64 | sca_18_chr2_1_0 | Scaffold8 |
| 1753645 | 1755119 | 246681 | 245211 | | 1475 | 1471 | 89.29 | sca_18_chr2_1_0 | Scaffold8 |
| 1762454 | 1762645 | 238184 | 237996 | | 192 | 189 | 92.71 | sca_18_chr2_1_0 | Scaffold8 |
| 1772717 | 1775682 | 228018 | 225039 | | 2966 | 2980 | 89.63 | sca_18_chr2_1_0 | Scaffold8 |
| 1832289 | 1835190 | 169428 | 166476 | | 2902 | 2953 | 91.78 | sca_18_chr2_1_0 | Scaffold8 |
| 1838652 | 1841556 | 163047 | 160092 | | 2905 | 2956 | 84.58 | sca_18_chr2_1_0 | Scaffold8 |
| 1848557 | 1850360 | 153500 | 151749 | | 1804 | 1752 | 88.05 | sca_18_chr2_1_0 | Scaffold8 |
| 1863683 | 1866886 | 140344 | 137101 | | 3204 | 3244 | 89.11 | sca_18_chr2_1_0 | Scaffold8 |
| 1870441 | 1875411 | 133468 | 128417 | | 4971 | 5052 | 88.19 | sca_18_chr2_1_0 | Scaffold8 |
| 1896603 | 1900030 | 107856 | 104398 | | 3428 | 3459 | 88.61 | sca_18_chr2_1_0 | Scaffold8 |
| 1903331 | 1904779 | 100943 | 99459 | | 1449 | 1485 | 89.06 | sca_18_chr2_1_0 | Scaffold8 |
| 1937434 | 1945543 | 67497 | 59352 | | 8110 | 8146 | 88.96 | sca_18_chr2_1_0 | Scaffold8 |
| 1949185 | 1951404 | 55690 | 53539 | | 2220 | 2152 | 89.76 | sca_18_chr2_1_0 | Scaffold8 |
| 1965463 | 1970390 | 40127 | 35233 | | 4928 | 4895 | 90.38 | sca_18_chr2_1_0 | Scaffold8 |
| 2005554 | 2007878 | 1366214 | 1363849 | | 2325 | 2366 | 89.38 | sca_18_chr2_1_0 | Scaffold7 |
| 2116432 | 2118351 | 1253890 | 1252000 | | 1920 | 1891 | 88.57 | sca_18_chr2_1_0 | Scaffold7 |
| 2251580 | 2254894 | 1118139 | 1114810 | | 3315 | 3330 | 87.46 | sca_18_chr2_1_0 | Scaffold7 |
| 2342555 | 2342762 | 1024066 | 1023860 | | 208 | 207 | 96.63 | sca_18_chr2_1_0 | Scaffold7 |
| 2352047 | 2353217 | 1014542 | 1013383 | | 1171 | 1160 | 87.94 | sca_18_chr2_1_0 | Scaffold7 |
| 2363006 | 2363154 | 1003909 | 1003760 | | 149 | 150 | 91.5 | sca_18_chr2_1_0 | Scaffold7 |
| 2363035 | 2363142 | 914610 | 914501 | | 108 | 110 | 98.18 | sca_18_chr2_1_0 | Scaffold7 |
| 2367594 | 2369370 | 999047 | 997288 | | 1777 | 1760 | 91.77 | sca_18_chr2_1_0 | Scaffold7 |
| 2454562 | 2457259 | 907396 | 904672 | | 2698 | 2725 | 89.37 | sca_18_chr2_1_0 | Scaffold7 |
| 2460497 | 2463421 | 901447 | 898531 | | 2925 | 2917 | 94.46 | sca_18_chr2_1_0 | Scaffold7 |
| 2483343 | 2488920 | 878726 | 873101 | | 5578 | 5626 | 88.46 | sca_18_chr2_1_0 | Scaffold7 |
| 2502778 | 2506154 | 862571 | 859218 | | 3377 | 3354 | 87.73 | sca_18_chr2_1_0 | Scaffold7 |
| 2511128 | 2515638 | 854631 | 850079 | | 4511 | 4553 | 87.72 | sca_18_chr2_1_0 | Scaffold7 |
| 2515858 | 2517422 | 849374 | 847805 | | 1565 | 1570 | 84.61 | sca_18_chr2_1_0 | Scaffold7 |
| 2526219 | 2529567 | 839047 | 835713 | | 3349 | 3335 | 88.58 | sca_18_chr2_1_0 | Scaffold7 |
| 2531673 | 2532727 | 833706 | 832662 | | 1055 | 1045 | 88.66 | sca_18_chr2_1_0 | Scaffold7 |
| 2568063 | 2570117 | 801965 | 799931 | | 2055 | 2035 | 89.45 | sca_18_chr2_1_0 | Scaffold7 |
| 2584555 | 2584756 | 785216 | 785011 | | 202 | 206 | 92.75 | sca_18_chr2_1_0 | Scaffold7 |
| 2623134 | 2625176 | 745860 | 743866 | | 2043 | 1995 | 86.09 | sca_18_chr2_1_0 | Scaffold7 |
| 2635249 | 2638754 | 733596 | 730079 | | 3506 | 3518 | 88.47 | sca_18_chr2_1_0 | Scaffold7 |
| 2645341 | 2647160 | 723149 | 721326 | | 1820 | 1824 | 87.47 | sca_18_chr2_1_0 | Scaffold7 |
| 2669849 | 2673392 | 698581 | 695041 | | 3544 | 3541 | 85.37 | sca_18_chr2_1_0 | Scaffold7 |
| 2739247 | 2739590 | 631791 | 631448 | | 344 | 344 | 91.27 | sca_18_chr2_1_0 | Scaffold7 |
| 2794520 | 2795502 | 588294 | 587309 | | 983 | 986 | 88.41 | sca_18_chr2_1_0 | Scaffold7 |
| 2800946 | 2801142 | 28940 | 29136 | | 197 | 197 | 98.98 | sca_18_chr2_1_0 | Scaffold58 |
| 2810269 | 2812008 | 578070 | 576335 | | 1740 | 1736 | 87.44 | sca_18_chr2_1_0 | Scaffold7 |
| 2866893 | 2868154 | 522827 | 521589 | | 1262 | 1239 | 87.86 | sca_18_chr2_1_0 | Scaffold7 |
| 2915486 | 2918069 | 481028 | 478469 | | 2584 | 2560 | 89.68 | sca_18_chr2_1_0 | Scaffold7 |
| 2938639 | 2941343 | 458586 | 455896 | | 2705 | 2691 | 88.12 | sca_18_chr2_1_0 | Scaffold7 |
| 2952459 | 2954373 | 439866 | 437950 | | 1915 | 1917 | 93.42 | sca_18_chr2_1_0 | Scaffold7 |
| 2963029 | 2966202 | 429400 | 426227 | | 3174 | 3174 | 87.12 | sca_18_chr2_1_0 | Scaffold7 |
| 3088971 | 3091978 | 299137 | 296073 | | 3008 | 3065 | 87.29 | sca_18_chr2_1_0 | Scaffold7 |
| 3092652 | 3096763 | 295435 | 291334 | | 4112 | 4102 | 90.46 | sca_18_chr2_1_0 | Scaffold7 |
| 3104875 | 3108670 | 283335 | 279575 | | 3796 | 3761 | 88.3 | sca_18_chr2_1_0 | Scaffold7 |
| 3138731 | 3140889 | 250459 | 248297 | | 2159 | 2163 | 91.13 | sca_18_chr2_1_0 | Scaffold7 |
| 3147954 | 3150538 | 240476 | 237866 | | 2585 | 2611 | 93.43 | sca_18_chr2_1_0 | Scaffold7 |
| 3170017 | 3173096 | 218403 | 215234 | | 3080 | 3170 | 87.17 | sca_18_chr2_1_0 | Scaffold7 |
| 3188640 | 3191258 | 199550 | 196855 | | 2619 | 2696 | 88.9 | sca_18_chr2_1_0 | Scaffold7 |
| 3201461 | 3203086 | 186662 | 185005 | | 1626 | 1658 | 87.23 | sca_18_chr2_1_0 | Scaffold7 |
| 3204701 | 3208314 | 183332 | 179700 | | 3614 | 3633 | 90.67 | sca_18_chr2_1_0 | Scaffold7 |
| 3288595 | 3291109 | 94047 | 91519 | | 2515 | 2529 | 91.1 | sca_18_chr2_1_0 | Scaffold7 |
| 3308173 | 3312374 | 74439 | 70156 | | 4202 | 4284 | 89.68 | sca_18_chr2_1_0 | Scaffold7 |
| 3317596 | 3317875 | 64500 | 64216 | | 280 | 285 | 94.08 | sca_18_chr2_1_0 | Scaffold7 |
| 3434629 | 3438473 | 50149 | 53996 | | 3845 | 3848 | 90.17 | sca_18_chr2_1_0 | Scaffold17 |
| 3438963 | 3440315 | 54433 | 55805 | | 1353 | 1373 | 88.56 | sca_18_chr2_1_0 | Scaffold17 |
| 3446687 | 3449371 | 62021 | 64712 | | 2685 | 2692 | 86.66 | sca_18_chr2_1_0 | Scaffold17 |
| 3480958 | 3483412 | 95372 | 97831 | | 2455 | 2460 | 87.5 | sca_18_chr2_1_0 | Scaffold17 |
| 3522171 | 3522704 | 134558 | 135079 | | 534 | 522 | 92.7 | sca_18_chr2_1_0 | Scaffold17 |
| 3586265 | 3587212 | 198420 | 199364 | | 948 | 945 | 84.67 | sca_18_chr2_1_0 | Scaffold17 |
| 3590709 | 3595158 | 202862 | 207304 | | 4450 | 4443 | 91.4 | sca_18_chr2_1_0 | Scaffold17 |
| 3603501 | 3610424 | 215386 | 222317 | | 6924 | 6932 | 89.7 | sca_18_chr2_1_0 | Scaffold17 |
| 3632093 | 3632219 | 213910 | 213784 | | 127 | 127 | 99.21 | sca_18_chr2_1_0 | Scaffold17 |
|  |  |  |  | |  |  |  |  |  |
| 2716 | 4086 | 818440 | 817090 | | 1371 | 1351 | 93.55 | sca_182_unmapped | Scaffold17 |
|  |  |  |  | |  |  |  |  |  |
| 130652 | 133829 | 4257506 | 4260698 | | 3178 | 3193 | 89.49 | sca_19_chr4_3_0 | Scaffold1 |
| 138330 | 143559 | 4265175 | 4270370 | | 5230 | 5196 | 89.06 | sca_19_chr4_3_0 | Scaffold1 |
| 202816 | 210905 | 4325547 | 4333594 | | 8090 | 8048 | 91.11 | sca_19_chr4_3_0 | Scaffold1 |
| 315590 | 315717 | 4426542 | 4426669 | | 128 | 128 | 100 | sca_19_chr4_3_0 | Scaffold1 |
| 315590 | 315719 | 429893 | 429764 | | 130 | 130 | 100 | sca_19_chr4_3_0 | Scaffold17 |
| 315590 | 315717 | 941112 | 941239 | | 128 | 128 | 100 | sca_19_chr4_3_0 | Scaffold2 |
| 315595 | 315719 | 1794501 | 1794625 | | 125 | 125 | 100 | sca_19_chr4_3_0 | Scaffold3 |
| 315607 | 315719 | 229000 | 229112 | | 113 | 113 | 100 | sca_19_chr4_3_0 | Scaffold16 |
| 467990 | 469608 | 4577419 | 4579029 | | 1619 | 1611 | 86.92 | sca_19_chr4_3_0 | Scaffold1 |
| 475184 | 475324 | 1792547 | 1792407 | | 141 | 141 | 97.87 | sca_19_chr4_3_0 | Scaffold2 |
| 647160 | 648193 | 2848 | 3884 | | 1034 | 1037 | 82.92 | sca_19_chr4_3_0 | Scaffold225 |
| 682428 | 684650 | 303057 | 305304 | | 2223 | 2248 | 89.43 | sca_19_chr4_3_0 | Scaffold16 |
|  |  |  |  | |  |  |  |  |  |
| 7432 | 8770 | 4403 | 3072 | | 1339 | 1332 | 95.38 | sca_191_unmapped | Scaffold47 |
| 6486 | 6672 | 60430 | 60244 | | 187 | 187 | 97.86 | sca_192_unmapped | Scaffold69 |
| 2045 | 2495 | 22917 | 22467 | | 451 | 451 | 95.34 | sca_198_unmapped | Scaffold115 |
| 8855 | 10475 | 12160 | 13779 | | 1621 | 1620 | 93.99 | sca_199_unmapped | Scaffold60 |
|  |  |  |  | |  |  |  |  |  |
| 266523 | 267791 | 486158 | 487430 | | 1269 | 1273 | 88 | sca_2_chr3_3_0 | Scaffold22 |
| 409824 | 412130 | 2222648 | 2224978 | | 2307 | 2331 | 88.22 | sca_2_chr3_3_0 | Scaffold4 |
| 519584 | 519719 | 4753309 | 4753446 | | 136 | 138 | 97.83 | sca_2_chr3_3_0 | Scaffold0 |
| 519590 | 519734 | 690552 | 690694 | | 145 | 143 | 97.24 | sca_2_chr3_3_0 | Scaffold9 |
| 519592 | 519717 | 675238 | 675363 | | 126 | 126 | 100 | sca_2_chr3_3_0 | Scaffold1 |
| 519592 | 519718 | 288113 | 287987 | | 127 | 127 | 99.21 | sca_2_chr3_3_0 | Scaffold17 |
| 519594 | 519728 | 1343544 | 1343411 | | 135 | 134 | 99.26 | sca_2_chr3_3_0 | Scaffold3 |
| 572540 | 573256 | 2078103 | 2077370 | | 717 | 734 | 90.22 | sca_2_chr3_3_0 | Scaffold4 |
| 574027 | 575239 | 2076617 | 2075359 | | 1213 | 1259 | 85.58 | sca_2_chr3_3_0 | Scaffold4 |
| 593869 | 596862 | 2056319 | 2053322 | | 2994 | 2998 | 86.8 | sca_2_chr3_3_0 | Scaffold4 |
| 704681 | 707298 | 1956383 | 1953746 | | 2618 | 2638 | 86.57 | sca_2_chr3_3_0 | Scaffold4 |
| 840375 | 842785 | 1671906 | 1674354 | | 2411 | 2449 | 87.63 | sca_2_chr3_3_0 | Scaffold4 |
| 844183 | 844523 | 1675606 | 1675936 | | 341 | 331 | 87.21 | sca_2_chr3_3_0 | Scaffold4 |
| 876474 | 878292 | 1707211 | 1709005 | | 1819 | 1795 | 93.53 | sca_2_chr3_3_0 | Scaffold4 |
| 890518 | 890634 | 1720594 | 1720710 | | 117 | 117 | 97.46 | sca_2_chr3_3_0 | Scaffold4 |
| 929506 | 933465 | 1759925 | 1763896 | | 3960 | 3972 | 88.68 | sca_2_chr3_3_0 | Scaffold4 |
| 976510 | 977938 | 1806374 | 1807789 | | 1429 | 1416 | 90.54 | sca_2_chr3_3_0 | Scaffold4 |
| 1010337 | 1011101 | 1839441 | 1840181 | | 765 | 741 | 84.53 | sca_2_chr3_3_0 | Scaffold4 |
| 1017244 | 1017802 | 1846024 | 1846600 | | 559 | 577 | 89.62 | sca_2_chr3_3_0 | Scaffold4 |
| 1017895 | 1021299 | 1846718 | 1850127 | | 3405 | 3410 | 89.64 | sca_2_chr3_3_0 | Scaffold4 |
| 1067386 | 1070329 | 1611111 | 1608173 | | 2944 | 2939 | 87.07 | sca_2_chr3_3_0 | Scaffold4 |
| 1123408 | 1126522 | 927929 | 931080 | | 3115 | 3152 | 87.44 | sca_2_chr3_3_0 | Scaffold4 |
| 1211992 | 1213813 | 1013584 | 1015412 | | 1822 | 1829 | 89.17 | sca_2_chr3_3_0 | Scaffold4 |
| 1226382 | 1230786 | 1028099 | 1032491 | | 4405 | 4393 | 87.94 | sca_2_chr3_3_0 | Scaffold4 |
| 1279657 | 1280028 | 1080028 | 1080397 | | 372 | 370 | 90.98 | sca_2_chr3_3_0 | Scaffold4 |
| 1292178 | 1296547 | 1092005 | 1096411 | | 4370 | 4407 | 91.45 | sca_2_chr3_3_0 | Scaffold4 |
| 1301314 | 1302438 | 1101157 | 1102289 | | 1125 | 1133 | 84.82 | sca_2_chr3_3_0 | Scaffold4 |
| 1304000 | 1305527 | 1103841 | 1105362 | | 1528 | 1522 | 91.69 | sca_2_chr3_3_0 | Scaffold4 |
| 1319568 | 1321824 | 1119199 | 1121410 | | 2257 | 2212 | 87.74 | sca_2_chr3_3_0 | Scaffold4 |
| 1321974 | 1323512 | 1121551 | 1123110 | | 1539 | 1560 | 87.96 | sca_2_chr3_3_0 | Scaffold4 |
| 1323739 | 1325276 | 1123324 | 1124871 | | 1538 | 1548 | 85.45 | sca_2_chr3_3_0 | Scaffold4 |
| 1329290 | 1330674 | 1128649 | 1130035 | | 1385 | 1387 | 86.86 | sca_2_chr3_3_0 | Scaffold4 |
| 1398737 | 1403279 | 1194454 | 1199013 | | 4543 | 4560 | 87.84 | sca_2_chr3_3_0 | Scaffold4 |
| 1428647 | 1428924 | 1226471 | 1226770 | | 278 | 300 | 90.67 | sca_2_chr3_3_0 | Scaffold4 |
| 1445453 | 1445877 | 1242854 | 1243275 | | 425 | 422 | 94.43 | sca_2_chr3_3_0 | Scaffold4 |
| 1446028 | 1447754 | 1243402 | 1245117 | | 1727 | 1716 | 88.42 | sca_2_chr3_3_0 | Scaffold4 |
| 1448506 | 1449689 | 1245812 | 1246979 | | 1184 | 1168 | 85.54 | sca_2_chr3_3_0 | Scaffold4 |
| 1451218 | 1454063 | 1248515 | 1251336 | | 2846 | 2822 | 89 | sca_2_chr3_3_0 | Scaffold4 |
| 1464270 | 1464672 | 1260802 | 1261204 | | 403 | 403 | 90.58 | sca_2_chr3_3_0 | Scaffold4 |
| 1465216 | 1466534 | 1261808 | 1263156 | | 1319 | 1349 | 89.27 | sca_2_chr3_3_0 | Scaffold4 |
| 1492086 | 1502202 | 1288048 | 1298111 | | 10117 | 10064 | 87.21 | sca_2_chr3_3_0 | Scaffold4 |
| 1548279 | 1549517 | 1351001 | 1349794 | | 1239 | 1208 | 88.08 | sca_2_chr3_3_0 | Scaffold4 |
| 1550856 | 1551844 | 1348530 | 1347544 | | 989 | 987 | 87.44 | sca_2_chr3_3_0 | Scaffold4 |
| 1553639 | 1555524 | 1345704 | 1343817 | | 1886 | 1888 | 88.48 | sca_2_chr3_3_0 | Scaffold4 |
| 1565817 | 1567563 | 1335075 | 1333356 | | 1747 | 1720 | 89.77 | sca_2_chr3_3_0 | Scaffold4 |
| 1571408 | 1571732 | 1329851 | 1329508 | | 325 | 344 | 86.44 | sca_2_chr3_3_0 | Scaffold4 |
| 1596389 | 1599568 | 1400550 | 1397377 | | 3180 | 3174 | 89.81 | sca_2_chr3_3_0 | Scaffold4 |
| 1737236 | 1741187 | 1516695 | 1520667 | | 3952 | 3973 | 90.08 | sca_2_chr3_3_0 | Scaffold4 |
| 1797339 | 1797789 | 1553604 | 1553146 | | 451 | 459 | 83.51 | sca_2_chr3_3_0 | Scaffold4 |
| 1862821 | 1862957 | 831860 | 831727 | | 137 | 134 | 94.93 | sca_2_chr3_3_0 | Scaffold4 |
| 1875012 | 1877047 | 819812 | 817787 | | 2036 | 2026 | 89.44 | sca_2_chr3_3_0 | Scaffold4 |
| 1900534 | 1902498 | 792776 | 790797 | | 1965 | 1980 | 88.58 | sca_2_chr3_3_0 | Scaffold4 |
| 1904364 | 1906101 | 789028 | 787277 | | 1738 | 1752 | 88.93 | sca_2_chr3_3_0 | Scaffold4 |
| 1928111 | 1929591 | 766747 | 765224 | | 1481 | 1524 | 87.66 | sca_2_chr3_3_0 | Scaffold4 |
| 2003950 | 2005357 | 691393 | 689984 | | 1408 | 1410 | 94.27 | sca_2_chr3_3_0 | Scaffold4 |
| 2066612 | 2067185 | 641092 | 640521 | | 574 | 572 | 88.74 | sca_2_chr3_3_0 | Scaffold4 |
| 2077659 | 2079072 | 629773 | 628357 | | 1414 | 1417 | 93.77 | sca_2_chr3_3_0 | Scaffold4 |
| 2094948 | 2098089 | 612567 | 609431 | | 3142 | 3137 | 87.95 | sca_2_chr3_3_0 | Scaffold4 |
| 2110474 | 2113481 | 597268 | 594272 | | 3008 | 2997 | 92.38 | sca_2_chr3_3_0 | Scaffold4 |
| 2202369 | 2202973 | 502921 | 502318 | | 605 | 604 | 88.94 | sca_2_chr3_3_0 | Scaffold4 |
| 2210283 | 2210640 | 495273 | 494915 | | 358 | 359 | 91.18 | sca_2_chr3_3_0 | Scaffold4 |
| 2260856 | 2261105 | 447574 | 447322 | | 250 | 253 | 86.49 | sca_2_chr3_3_0 | Scaffold4 |
| 2275257 | 2278275 | 433248 | 430190 | | 3019 | 3059 | 90.17 | sca_2_chr3_3_0 | Scaffold4 |
| 2292473 | 2296774 | 416354 | 412149 | | 4302 | 4206 | 88.4 | sca_2_chr3_3_0 | Scaffold4 |
| 2414528 | 2415203 | 255430 | 254761 | | 676 | 670 | 85.42 | sca_2_chr3_3_0 | Scaffold4 |
| 2428266 | 2430688 | 241934 | 239494 | | 2423 | 2441 | 93.8 | sca_2_chr3_3_0 | Scaffold4 |
| 2629559 | 2638745 | 119042 | 109781 | | 9187 | 9262 | 94.25 | sca_2_chr3_3_0 | Scaffold55 |
| 2792817 | 2794758 | 822182 | 824118 | | 1942 | 1937 | 92.61 | sca_2_chr3_3_0 | Scaffold16 |
| 2851219 | 2853574 | 726405 | 724061 | | 2356 | 2345 | 88.05 | sca_2_chr3_3_0 | Scaffold16 |
| 2874063 | 2877293 | 718843 | 715591 | | 3231 | 3253 | 91.25 | sca_2_chr3_3_0 | Scaffold16 |
| 2895675 | 2896657 | 694973 | 693989 | | 983 | 985 | 88.78 | sca_2_chr3_3_0 | Scaffold16 |
| 2931158 | 2932042 | 665183 | 664312 | | 885 | 872 | 87.18 | sca_2_chr3_3_0 | Scaffold16 |
| 2951580 | 2955383 | 645888 | 642077 | | 3804 | 3812 | 91.27 | sca_2_chr3_3_0 | Scaffold16 |
| 2979080 | 2981260 | 622349 | 620156 | | 2181 | 2194 | 90.42 | sca_2_chr3_3_0 | Scaffold16 |
| 3081185 | 3082057 | 544224 | 543351 | | 873 | 874 | 85.43 | sca_2_chr3_3_0 | Scaffold16 |
| 3109444 | 3110311 | 516828 | 515955 | | 868 | 874 | 88.35 | sca_2_chr3_3_0 | Scaffold16 |
| 3120668 | 3122403 | 505355 | 503624 | | 1736 | 1732 | 86.81 | sca_2_chr3_3_0 | Scaffold16 |
| 3129814 | 3130510 | 496032 | 495333 | | 697 | 700 | 88.47 | sca_2_chr3_3_0 | Scaffold16 |
| 3131937 | 3132142 | 493925 | 493711 | | 206 | 215 | 93.02 | sca_2_chr3_3_0 | Scaffold16 |
| 3133880 | 3135733 | 491851 | 490024 | | 1854 | 1828 | 87.51 | sca_2_chr3_3_0 | Scaffold16 |
| 3168274 | 3170027 | 459723 | 457971 | | 1754 | 1753 | 87.94 | sca_2_chr3_3_0 | Scaffold16 |
| 3226574 | 3226700 | 4753445 | 4753319 | | 127 | 127 | 100 | sca_2_chr3_3_0 | Scaffold0 |
| 3233969 | 3236079 | 400018 | 397930 | | 2111 | 2089 | 86.15 | sca_2_chr3_3_0 | Scaffold16 |
| 3248427 | 3250359 | 385994 | 384068 | | 1933 | 1927 | 86.11 | sca_2_chr3_3_0 | Scaffold16 |
|  |  |  |  | |  |  |  |  |  |
| 181210 | 183954 | 471823 | 469058 | | 2745 | 2766 | 88.98 | sca_20_chr6_4_0 | Scaffold12 |
| 306259 | 310729 | 355926 | 351452 | | 4471 | 4475 | 89 | sca_20_chr6_4_0 | Scaffold12 |
| 399031 | 402466 | 256063 | 252632 | | 3436 | 3432 | 93.98 | sca_20_chr6_4_0 | Scaffold12 |
| 424059 | 425623 | 231595 | 230014 | | 1565 | 1582 | 88.13 | sca_20_chr6_4_0 | Scaffold12 |
| 441940 | 443907 | 212548 | 210583 | | 1968 | 1966 | 91.21 | sca_20_chr6_4_0 | Scaffold12 |
| 452138 | 458125 | 202596 | 196622 | | 5988 | 5975 | 89.47 | sca_20_chr6_4_0 | Scaffold12 |
| 484313 | 490361 | 168711 | 162636 | | 6049 | 6076 | 90.48 | sca_20_chr6_4_0 | Scaffold12 |
| 524663 | 525159 | 130260 | 129761 | | 497 | 500 | 90.23 | sca_20_chr6_4_0 | Scaffold12 |
| 798812 | 799823 | 1394849 | 1393829 | | 1012 | 1021 | 83.61 | sca_20_chr6_4_0 | Scaffold6 |
| 862781 | 866165 | 1351275 | 1347912 | | 3385 | 3364 | 86.57 | sca_20_chr6_4_0 | Scaffold6 |
| 869700 | 871480 | 1344681 | 1342889 | | 1781 | 1793 | 90.19 | sca_20_chr6_4_0 | Scaffold6 |
| 913307 | 914638 | 1298742 | 1297446 | | 1332 | 1297 | 87.52 | sca_20_chr6_4_0 | Scaffold6 |
| 937647 | 938954 | 1274526 | 1273240 | | 1308 | 1287 | 84.97 | sca_20_chr6_4_0 | Scaffold6 |
| 1010174 | 1013384 | 1200740 | 1197520 | | 3211 | 3221 | 86.77 | sca_20_chr6_4_0 | Scaffold6 |
| 1045492 | 1049996 | 1130668 | 1135110 | | 4505 | 4443 | 90.04 | sca_20_chr6_4_0 | Scaffold6 |
| 1051268 | 1051736 | 1136308 | 1136762 | | 469 | 455 | 89.5 | sca_20_chr6_4_0 | Scaffold6 |
| 1054176 | 1056518 | 1139047 | 1141461 | | 2343 | 2415 | 86.68 | sca_20_chr6_4_0 | Scaffold6 |
| 1137910 | 1141047 | 1070246 | 1067121 | | 3138 | 3126 | 89.74 | sca_20_chr6_4_0 | Scaffold6 |
| 1151194 | 1151508 | 1057217 | 1056888 | | 315 | 330 | 89.76 | sca_20_chr6_4_0 | Scaffold6 |
| 1207843 | 1211508 | 1001783 | 998105 | | 3666 | 3679 | 92.61 | sca_20_chr6_4_0 | Scaffold6 |
| 1240194 | 1243343 | 970447 | 967300 | | 3150 | 3148 | 87.58 | sca_20_chr6_4_0 | Scaffold6 |
| 1260551 | 1262724 | 950455 | 948279 | | 2174 | 2177 | 92.33 | sca_20_chr6_4_0 | Scaffold6 |
| 1269835 | 1271532 | 940930 | 939199 | | 1698 | 1732 | 89.19 | sca_20_chr6_4_0 | Scaffold6 |
| 1276602 | 1278143 | 934064 | 932507 | | 1542 | 1558 | 91.33 | sca_20_chr6_4_0 | Scaffold6 |
| 1285833 | 1288012 | 925466 | 923292 | | 2180 | 2175 | 88.74 | sca_20_chr6_4_0 | Scaffold6 |
| 1306483 | 1307728 | 903281 | 902028 | | 1246 | 1254 | 89.77 | sca_20_chr6_4_0 | Scaffold6 |
| 1395704 | 1397490 | 819226 | 817441 | | 1787 | 1786 | 91.49 | sca_20_chr6_4_0 | Scaffold6 |
| 1413037 | 1413162 | 4753320 | 4753445 | | 126 | 126 | 100 | sca_20_chr6_4_0 | Scaffold0 |
| 1418498 | 1418831 | 794016 | 793686 | | 334 | 331 | 90.03 | sca_20_chr6_4_0 | Scaffold6 |
| 1445115 | 1445812 | 768204 | 767564 | | 698 | 641 | 83.74 | sca_20_chr6_4_0 | Scaffold6 |
| 1509618 | 1513893 | 706731 | 702447 | | 4276 | 4285 | 87.86 | sca_20_chr6_4_0 | Scaffold6 |
| 1544495 | 1547671 | 676340 | 673178 | | 3177 | 3163 | 92.15 | sca_20_chr6_4_0 | Scaffold6 |
| 1549002 | 1550664 | 671874 | 670202 | | 1663 | 1673 | 93.68 | sca_20_chr6_4_0 | Scaffold6 |
| 1561694 | 1565326 | 659378 | 655742 | | 3633 | 3637 | 90.52 | sca_20_chr6_4_0 | Scaffold6 |
| 1584162 | 1589312 | 636628 | 631440 | | 5151 | 5189 | 87.6 | sca_20_chr6_4_0 | Scaffold6 |
| 1718646 | 1719108 | 286762 | 286253 | | 463 | 510 | 84.71 | sca_20_chr6_4_0 | Scaffold6 |
| 1752041 | 1752273 | 306891 | 307135 | | 233 | 245 | 85.71 | sca_20_chr6_4_0 | Scaffold6 |
| 1873949 | 1878363 | 410590 | 415074 | | 4415 | 4485 | 88.5 | sca_20_chr6_4_0 | Scaffold6 |
| 1887578 | 1889700 | 423857 | 425978 | | 2123 | 2122 | 86.96 | sca_20_chr6_4_0 | Scaffold6 |
| 1956026 | 1958710 | 475424 | 478157 | | 2685 | 2734 | 88.99 | sca_20_chr6_4_0 | Scaffold6 |
| 1983903 | 1988879 | 502419 | 507349 | | 4977 | 4931 | 88.26 | sca_20_chr6_4_0 | Scaffold6 |
|  |  |  |  | |  |  |  |  |  |
| 182274 | 183422 | 555102 | 556235 | | 1149 | 1134 | 87.46 | sca_22_chr7_11_0 | Scaffold11 |
| 254878 | 255941 | 482264 | 481202 | | 1064 | 1063 | 91.35 | sca_22_chr7_11_0 | Scaffold11 |
| 281839 | 283046 | 456680 | 455491 | | 1208 | 1190 | 87.42 | sca_22_chr7_11_0 | Scaffold11 |
|  |  |  |  | |  |  |  |  |  |
| 6627 | 10650 | 40729 | 44744 | | 4024 | 4016 | 95.7 | sca_23_chr14_3_0 | Scaffold69 |
| 28309 | 30036 | 10923 | 9196 | | 1728 | 1728 | 96.06 | sca_23_chr14_3_0 | Scaffold71 |
| 30301 | 38325 | 8916 | 842 | | 8025 | 8075 | 94.51 | sca_23_chr14_3_0 | Scaffold71 |
| 63678 | 65333 | 25906 | 24245 | | 1656 | 1662 | 93.08 | sca_23_chr14_3_0 | Scaffold79 |
| 82894 | 83040 | 129472 | 129617 | | 147 | 146 | 97.96 | sca_23_chr14_3_0 | Scaffold41 |
| 161200 | 161777 | 128236 | 127659 | | 578 | 578 | 90.83 | sca_23_chr14_3_0 | Scaffold41 |
| 161200 | 161355 | 17961 | 18115 | | 156 | 155 | 98.08 | sca_23_chr14_3_0 | Scaffold54 |
| 161200 | 161777 | 79881 | 80453 | | 578 | 573 | 94.98 | sca_23_chr14_3_0 | Scaffold57 |
| 161930 | 162195 | 127659 | 127395 | | 266 | 265 | 98.5 | sca_23_chr14_3_0 | Scaffold41 |
| 166418 | 166572 | 162327 | 162480 | | 155 | 154 | 97.42 | sca_23_chr14_3_0 | Scaffold41 |
| 290613 | 290977 | 117640 | 118003 | | 365 | 364 | 93.97 | sca_23_chr14_3_0 | Scaffold28 |
| 291854 | 293697 | 159836 | 161711 | | 1844 | 1876 | 93.39 | sca_23_chr14_3_0 | Scaffold28 |
| 301250 | 303094 | 140907 | 142752 | | 1845 | 1846 | 92.47 | sca_23_chr14_3_0 | Scaffold28 |
| 310595 | 312255 | 145291 | 146950 | | 1661 | 1660 | 91.77 | sca_23_chr14_3_0 | Scaffold28 |
| 315443 | 320145 | 6612 | 1894 | | 4703 | 4719 | 93 | sca_23_chr14_3_0 | Scaffold202 |
| 316395 | 320930 | 151546 | 156111 | | 4536 | 4566 | 94.32 | sca_23_chr14_3_0 | Scaffold28 |
| 321714 | 322622 | 156442 | 157326 | | 909 | 885 | 91.68 | sca_23_chr14_3_0 | Scaffold28 |
| 330131 | 332167 | 163042 | 165079 | | 2037 | 2038 | 91.95 | sca_23_chr14_3_0 | Scaffold28 |
| 331444 | 332167 | 176186 | 175469 | | 724 | 718 | 93.39 | sca_23_chr14_3_0 | Scaffold28 |
| 334262 | 335463 | 175466 | 174256 | | 1202 | 1211 | 93.74 | sca_23_chr14_3_0 | Scaffold28 |
| 335474 | 336795 | 173795 | 172471 | | 1322 | 1325 | 94.58 | sca_23_chr14_3_0 | Scaffold28 |
| 336285 | 338144 | 166210 | 168079 | | 1860 | 1870 | 94.41 | sca_23_chr14_3_0 | Scaffold28 |
| 345504 | 348625 | 180364 | 183475 | | 3122 | 3112 | 92.61 | sca_23_chr14_3_0 | Scaffold28 |
| 350934 | 353508 | 185741 | 188305 | | 2575 | 2565 | 94.22 | sca_23_chr14_3_0 | Scaffold28 |
| 361299 | 365385 | 188303 | 192397 | | 4087 | 4095 | 92.31 | sca_23_chr14_3_0 | Scaffold28 |
| 366784 | 368730 | 193976 | 195944 | | 1947 | 1969 | 88.56 | sca_23_chr14_3_0 | Scaffold28 |
| 418073 | 420187 | 44711 | 46826 | | 2115 | 2116 | 92.17 | sca_23_chr14_3_0 | Scaffold28 |
| 11145 | 11331 | 60244 | 60430 | | 187 | 187 | 100 | sca_24_chr14_5_0 | Scaffold69 |
| 20103 | 20261 | 72503 | 72345 | | 159 | 159 | 95.6 | sca_24_chr14_5_0 | Scaffold67 |
| 21217 | 21824 | 15392 | 15979 | | 608 | 588 | 92.12 | sca_24_chr14_5_0 | Scaffold127 |
| 21343 | 22508 | 106623 | 107809 | | 1166 | 1187 | 95.79 | sca_24_chr14_5_0 | Scaffold54 |
| 21350 | 22167 | 51112 | 51929 | | 818 | 818 | 95.49 | sca_24_chr14_5_0 | Scaffold88 |
| 22123 | 22718 | 29068 | 29663 | | 596 | 596 | 98.66 | sca_24_chr14_5_0 | Scaffold88 |
| 24861 | 25173 | 44380 | 44692 | | 313 | 313 | 99.36 | sca_24_chr14_5_0 | Scaffold98 |
| 29323 | 29771 | 320118 | 320566 | | 449 | 449 | 96.44 | sca_24_chr14_5_0 | Scaffold33 |
| 30458 | 31246 | 109313 | 110099 | | 789 | 787 | 96.07 | sca_24_chr14_5_0 | Scaffold54 |
| 45904 | 48086 | 75313 | 73131 | | 2183 | 2183 | 89.24 | sca_24_chr14_5_0 | Scaffold75 |
| 62136 | 62987 | 154601 | 155454 | | 852 | 854 | 90.63 | sca_24_chr14_5_0 | Scaffold34 |
| 63037 | 64455 | 160048 | 161471 | | 1419 | 1424 | 91.92 | sca_24_chr14_5_0 | Scaffold34 |
| 71925 | 72856 | 13105 | 12173 | | 932 | 933 | 90.46 | sca_24_chr14_5_0 | Scaffold54 |
| 72857 | 73052 | 136782 | 136587 | | 196 | 196 | 98.47 | sca_24_chr14_5_0 | Scaffold41 |
| 72857 | 73050 | 4568 | 4375 | | 194 | 194 | 97.94 | sca_24_chr14_5_0 | Scaffold92 |
| 83524 | 84615 | 70633 | 71724 | | 1092 | 1092 | 92.67 | sca_24_chr14_5_0 | Scaffold75 |
| 92548 | 92928 | 416 | 33 | | 381 | 384 | 95.87 | sca_24_chr14_5_0 | Scaffold100 |
| 92559 | 92928 | 9693 | 10060 | | 370 | 368 | 97.3 | sca_24_chr14_5_0 | Scaffold47 |
| 92787 | 92928 | 10104 | 10245 | | 142 | 142 | 99.3 | sca_24_chr14_5_0 | Scaffold106 |
| 101024 | 101152 | 23543 | 23672 | | 129 | 130 | 97.69 | sca_24_chr14_5_0 | Scaffold100 |
| 127910 | 128156 | 91690 | 91459 | | 247 | 232 | 91.9 | sca_24_chr14_5_0 | Scaffold41 |
| 127910 | 128157 | 65882 | 65650 | | 248 | 233 | 91.53 | sca_24_chr14_5_0 | Scaffold50 |
| 142958 | 144376 | 161471 | 160048 | | 1419 | 1424 | 91.99 | sca_24_chr14_5_0 | Scaffold34 |
| 144426 | 145278 | 155454 | 154600 | | 853 | 855 | 90.06 | sca_24_chr14_5_0 | Scaffold34 |
| 159193 | 162551 | 20886 | 24244 | | 3359 | 3359 | 98.18 | sca_24_chr14_5_0 | Scaffold79 |
| 169614 | 169808 | 61657 | 61851 | | 195 | 195 | 96.41 | sca_24_chr14_5_0 | Scaffold46 |
| 169614 | 169744 | 92481 | 92611 | | 131 | 131 | 99.24 | sca_24_chr14_5_0 | Scaffold57 |
| 169615 | 169808 | 12390 | 12580 | | 194 | 191 | 95.88 | sca_24_chr14_5_0 | Scaffold88 |
| 177680 | 179062 | 2562 | 1165 | | 1383 | 1398 | 94.78 | sca_24_chr14_5_0 | Scaffold110 |
| 198321 | 199412 | 71724 | 70633 | | 1092 | 1092 | 94.6 | sca_24_chr14_5_0 | Scaffold75 |
| 242616 | 246016 | 18329 | 14922 | | 3401 | 3408 | 97.3 | sca_24_chr14_5_0 | Scaffold125 |
| 274654 | 277401 | 28675 | 25935 | | 2748 | 2741 | 95.56 | sca_24_chr14_5_0 | Scaffold58 |
| 319808 | 320418 | 15291 | 15901 | | 611 | 611 | 98.85 | sca_24_chr14_5_0 | Scaffold100 |
| 335060 | 336150 | 70633 | 71723 | | 1091 | 1091 | 97.07 | sca_24_chr14_5_0 | Scaffold75 |
| 344497 | 344665 | 28010 | 28178 | | 169 | 169 | 97.63 | sca_24_chr14_5_0 | Scaffold90 |
| 345862 | 346485 | 28178 | 28803 | | 624 | 626 | 97.45 | sca_24_chr14_5_0 | Scaffold90 |
| 346522 | 346748 | 29768 | 29542 | | 227 | 227 | 98.68 | sca_24_chr14_5_0 | Scaffold90 |
| 349009 | 353162 | 17293 | 13106 | | 4154 | 4188 | 92.57 | sca_24_chr14_5_0 | Scaffold54 |
| 391666 | 392232 | 15857 | 15291 | | 567 | 567 | 96.3 | sca_24_chr14_5_0 | Scaffold100 |
|  |  |  |  | |  |  |  |  |  |
| 63524 | 63907 | 32888 | 33271 | | 384 | 384 | 97.66 | sca_25_chr15_3_0 | Scaffold69 |
|  |  |  |  | |  |  |  |  |  |
| 30546 | 31500 | 404574 | 405521 | | 955 | 948 | 86.19 | sca_26_chr2_2_0 | Scaffold17 |
| 41898 | 45500 | 415637 | 419237 | | 3603 | 3601 | 88.14 | sca_26_chr2_2_0 | Scaffold17 |
| 56053 | 56182 | 1343545 | 1343416 | | 130 | 130 | 99.23 | sca_26_chr2_2_0 | Scaffold3 |
| 66477 | 71828 | 440467 | 445852 | | 5352 | 5386 | 90.7 | sca_26_chr2_2_0 | Scaffold17 |
| 72044 | 73252 | 446085 | 447271 | | 1209 | 1187 | 89.42 | sca_26_chr2_2_0 | Scaffold17 |
| 76448 | 77285 | 450348 | 451144 | | 838 | 797 | 88.21 | sca_26_chr2_2_0 | Scaffold17 |
| 147447 | 152609 | 515924 | 521110 | | 5163 | 5187 | 89.87 | sca_26_chr2_2_0 | Scaffold17 |
|  |  |  |  | |  |  |  |  |  |
| 1 | 438 | 26942 | 27376 | | 438 | 435 | 90.43 | sca_27_chr9_1_0 | Scaffold111 |
| 17 | 1290 | 126751 | 128021 | | 1274 | 1271 | 84.29 | sca_27_chr9_1_0 | Scaffold34 |
| 389474 | 390509 | 2021899 | 2020851 | | 1036 | 1049 | 87.62 | sca_27_chr9_1_0 | Scaffold3 |
| 420600 | 421558 | 1989136 | 1988181 | | 959 | 956 | 86.4 | sca_27_chr9_1_0 | Scaffold3 |
| 503922 | 507055 | 1913569 | 1910432 | | 3134 | 3138 | 89.6 | sca_27_chr9_1_0 | Scaffold3 |
| 511272 | 512490 | 1906278 | 1905057 | | 1219 | 1222 | 86.18 | sca_27_chr9_1_0 | Scaffold3 |
| 625777 | 625901 | 941241 | 941117 | | 125 | 125 | 100 | sca_27_chr9_1_0 | Scaffold2 |
|  |  |  |  | |  |  |  |  |  |
| 310401 | 313508 | 186292 | 183172 | | 3108 | 3121 | 86.34 | sca_28_chr13_5_0 | Scaffold1 |
| 330558 | 331885 | 168328 | 166994 | | 1328 | 1335 | 88.36 | sca_28_chr13_5_0 | Scaffold1 |
| 333954 | 338222 | 164943 | 160651 | | 4269 | 4293 | 89.55 | sca_28_chr13_5_0 | Scaffold1 |
| 339088 | 339238 | 159904 | 159760 | | 151 | 145 | 93.42 | sca_28_chr13_5_0 | Scaffold1 |
|  |  |  |  | |  |  |  |  |  |
| 21881 | 25482 | 10841 | 7254 | | 3602 | 3588 | 86.89 | sca_29_chr12_4_0 | Scaffold109 |
| 31967 | 32098 | 429763 | 429894 | | 132 | 132 | 100 | sca_29_chr12_4_0 | Scaffold17 |
| 31970 | 32098 | 4426669 | 4426541 | | 129 | 129 | 100 | sca_29_chr12_4_0 | Scaffold1 |
| 31970 | 32098 | 941239 | 941111 | | 129 | 129 | 100 | sca_29_chr12_4_0 | Scaffold2 |
| 440977 | 445132 | 13106 | 17295 | | 4156 | 4190 | 93.29 | sca_29_chr12_4_0 | Scaffold54 |
|  |  |  |  | |  |  |  |  |  |
| 7019 | 9535 | 778369 | 780862 | | 2517 | 2494 | 86.69 | sca_3_chr4_2_0 | Scaffold1 |
| 66175 | 70595 | 837510 | 841911 | | 4421 | 4402 | 89.75 | sca_3_chr4_2_0 | Scaffold1 |
| 71901 | 72884 | 843149 | 844127 | | 984 | 979 | 88.14 | sca_3_chr4_2_0 | Scaffold1 |
| 102885 | 105182 | 871770 | 874049 | | 2298 | 2280 | 89.71 | sca_3_chr4_2_0 | Scaffold1 |
| 106930 | 110977 | 875755 | 879871 | | 4048 | 4117 | 89.96 | sca_3_chr4_2_0 | Scaffold1 |
| 117197 | 120265 | 885410 | 888496 | | 3069 | 3087 | 88.2 | sca_3_chr4_2_0 | Scaffold1 |
| 227556 | 228822 | 991214 | 992509 | | 1267 | 1296 | 85.9 | sca_3_chr4_2_0 | Scaffold1 |
| 229209 | 230833 | 992876 | 994562 | | 1625 | 1687 | 86.26 | sca_3_chr4_2_0 | Scaffold1 |
| 246983 | 249707 | 1010845 | 1013588 | | 2725 | 2744 | 89.62 | sca_3_chr4_2_0 | Scaffold1 |
| 350951 | 354814 | 1114551 | 1118421 | | 3864 | 3871 | 88.22 | sca_3_chr4_2_0 | Scaffold1 |
| 373537 | 377552 | 1136963 | 1141066 | | 4016 | 4104 | 87.73 | sca_3_chr4_2_0 | Scaffold1 |
| 434579 | 438832 | 1195481 | 1199739 | | 4254 | 4259 | 89.57 | sca_3_chr4_2_0 | Scaffold1 |
| 440263 | 442161 | 1201223 | 1203123 | | 1899 | 1901 | 92.54 | sca_3_chr4_2_0 | Scaffold1 |
| 448665 | 453739 | 1209595 | 1214635 | | 5075 | 5041 | 88.65 | sca_3_chr4_2_0 | Scaffold1 |
| 454065 | 455864 | 1214928 | 1216706 | | 1800 | 1779 | 94.25 | sca_3_chr4_2_0 | Scaffold1 |
| 468504 | 471192 | 1229159 | 1231852 | | 2689 | 2694 | 91.02 | sca_3_chr4_2_0 | Scaffold1 |
| 473318 | 473778 | 1234121 | 1234571 | | 461 | 451 | 86.72 | sca_3_chr4_2_0 | Scaffold1 |
| 491553 | 491985 | 1252240 | 1252672 | | 433 | 433 | 93.79 | sca_3_chr4_2_0 | Scaffold1 |
| 496292 | 497419 | 1256750 | 1257847 | | 1128 | 1098 | 87.41 | sca_3_chr4_2_0 | Scaffold1 |
| 511638 | 513463 | 1271825 | 1273640 | | 1826 | 1816 | 88.66 | sca_3_chr4_2_0 | Scaffold1 |
| 515287 | 518656 | 1275460 | 1278861 | | 3370 | 3402 | 87.86 | sca_3_chr4_2_0 | Scaffold1 |
| 518613 | 518909 | 1279043 | 1279353 | | 297 | 311 | 88.05 | sca_3_chr4_2_0 | Scaffold1 |
| 549752 | 551864 | 1310041 | 1312180 | | 2113 | 2140 | 89.18 | sca_3_chr4_2_0 | Scaffold1 |
| 552696 | 553046 | 1312999 | 1313351 | | 351 | 353 | 86.81 | sca_3_chr4_2_0 | Scaffold1 |
| 584691 | 586839 | 1344303 | 1346426 | | 2149 | 2124 | 88.85 | sca_3_chr4_2_0 | Scaffold1 |
| 598255 | 603512 | 1356651 | 1361927 | | 5258 | 5277 | 89.58 | sca_3_chr4_2_0 | Scaffold1 |
| 610379 | 613334 | 1368893 | 1371831 | | 2956 | 2939 | 88.52 | sca_3_chr4_2_0 | Scaffold1 |
| 620798 | 621684 | 1379103 | 1379997 | | 887 | 895 | 91.64 | sca_3_chr4_2_0 | Scaffold1 |
| 643026 | 645597 | 1400717 | 1403338 | | 2572 | 2622 | 87.91 | sca_3_chr4_2_0 | Scaffold1 |
| 661779 | 664167 | 1419231 | 1421639 | | 2389 | 2409 | 87.16 | sca_3_chr4_2_0 | Scaffold1 |
| 685963 | 690181 | 1590123 | 1594322 | | 4219 | 4200 | 87.5 | sca_3_chr4_2_0 | Scaffold1 |
| 704432 | 706054 | 1608467 | 1610076 | | 1623 | 1610 | 89.21 | sca_3_chr4_2_0 | Scaffold1 |
| 739469 | 743230 | 1642117 | 1645880 | | 3762 | 3764 | 90.14 | sca_3_chr4_2_0 | Scaffold1 |
| 765734 | 767949 | 1668036 | 1670209 | | 2216 | 2174 | 86.75 | sca_3_chr4_2_0 | Scaffold1 |
| 794510 | 802004 | 1695683 | 1703189 | | 7495 | 7507 | 88.03 | sca_3_chr4_2_0 | Scaffold1 |
| 802860 | 803768 | 1703930 | 1704835 | | 909 | 906 | 86.17 | sca_3_chr4_2_0 | Scaffold1 |
| 814558 | 815557 | 1715489 | 1716546 | | 1000 | 1058 | 83.91 | sca_3_chr4_2_0 | Scaffold1 |
| 825155 | 826999 | 1725819 | 1727677 | | 1845 | 1859 | 89.48 | sca_3_chr4_2_0 | Scaffold1 |
| 876935 | 883389 | 1777231 | 1783704 | | 6455 | 6474 | 88.93 | sca_3_chr4_2_0 | Scaffold1 |
| 901599 | 902072 | 1800779 | 1801252 | | 474 | 474 | 87.37 | sca_3_chr4_2_0 | Scaffold1 |
| 913489 | 915522 | 1812812 | 1814859 | | 2034 | 2048 | 91.96 | sca_3_chr4_2_0 | Scaffold1 |
| 919512 | 921486 | 1818527 | 1820499 | | 1975 | 1973 | 91.92 | sca_3_chr4_2_0 | Scaffold1 |
| 945797 | 948063 | 1844352 | 1846627 | | 2267 | 2276 | 90.78 | sca_3_chr4_2_0 | Scaffold1 |
| 955351 | 958889 | 1853489 | 1857015 | | 3539 | 3527 | 88.35 | sca_3_chr4_2_0 | Scaffold1 |
| 974217 | 976961 | 1872020 | 1874791 | | 2745 | 2772 | 88.53 | sca_3_chr4_2_0 | Scaffold1 |
| 983860 | 987349 | 1881326 | 1884821 | | 3490 | 3496 | 89.63 | sca_3_chr4_2_0 | Scaffold1 |
| 1012469 | 1013278 | 1910749 | 1911558 | | 810 | 810 | 93.95 | sca_3_chr4_2_0 | Scaffold1 |
| 1059534 | 1063300 | 1961686 | 1965424 | | 3767 | 3739 | 89.09 | sca_3_chr4_2_0 | Scaffold1 |
| 1066998 | 1067909 | 1969136 | 1970065 | | 912 | 930 | 89.43 | sca_3_chr4_2_0 | Scaffold1 |
| 1071463 | 1076577 | 1973528 | 1978634 | | 5115 | 5107 | 87.34 | sca_3_chr4_2_0 | Scaffold1 |
| 1142414 | 1143146 | 2045242 | 2045979 | | 733 | 738 | 92.82 | sca_3_chr4_2_0 | Scaffold1 |
| 1235867 | 1237844 | 2134780 | 2136748 | | 1978 | 1969 | 85.02 | sca_3_chr4_2_0 | Scaffold1 |
| 1252386 | 1256030 | 2150585 | 2154245 | | 3645 | 3661 | 89.42 | sca_3_chr4_2_0 | Scaffold1 |
| 1290158 | 1292664 | 2187970 | 2190550 | | 2507 | 2581 | 88.43 | sca_3_chr4_2_0 | Scaffold1 |
| 1341332 | 1346000 | 2257132 | 2252379 | | 4669 | 4754 | 90.17 | sca_3_chr4_2_0 | Scaffold1 |
| 1359021 | 1360220 | 2270353 | 2271545 | | 1200 | 1193 | 89.98 | sca_3_chr4_2_0 | Scaffold1 |
| 1365989 | 1369666 | 2277136 | 2280745 | | 3678 | 3610 | 87.89 | sca_3_chr4_2_0 | Scaffold1 |
| 1405750 | 1408342 | 2315018 | 2317663 | | 2593 | 2646 | 88.48 | sca_3_chr4_2_0 | Scaffold1 |
| 1434811 | 1435778 | 2356366 | 2357351 | | 968 | 986 | 88.28 | sca_3_chr4_2_0 | Scaffold1 |
| 1437708 | 1440022 | 2359313 | 2361652 | | 2315 | 2340 | 88.5 | sca_3_chr4_2_0 | Scaffold1 |
| 1447461 | 1454051 | 2368973 | 2375520 | | 6591 | 6548 | 88.16 | sca_3_chr4_2_0 | Scaffold1 |
| 1455201 | 1461403 | 2376614 | 2382815 | | 6203 | 6202 | 89.41 | sca_3_chr4_2_0 | Scaffold1 |
| 1495043 | 1496045 | 2417307 | 2418318 | | 1003 | 1012 | 89.73 | sca_3_chr4_2_0 | Scaffold1 |
| 1587405 | 1588297 | 2542607 | 2541720 | | 893 | 888 | 88 | sca_3_chr4_2_0 | Scaffold1 |
| 1591761 | 1593844 | 2538249 | 2536119 | | 2084 | 2131 | 88.66 | sca_3_chr4_2_0 | Scaffold1 |
| 1650232 | 1650359 | 2570416 | 2570543 | | 128 | 128 | 98.44 | sca_3_chr4_2_0 | Scaffold1 |
| 1659802 | 1662034 | 2579930 | 2582204 | | 2233 | 2275 | 89.8 | sca_3_chr4_2_0 | Scaffold1 |
| 1663439 | 1667278 | 2583551 | 2587406 | | 3840 | 3856 | 89.77 | sca_3_chr4_2_0 | Scaffold1 |
| 1671094 | 1677016 | 2591526 | 2597545 | | 5923 | 6020 | 88.46 | sca_3_chr4_2_0 | Scaffold1 |
| 1677394 | 1679111 | 2597917 | 2599592 | | 1718 | 1676 | 84.87 | sca_3_chr4_2_0 | Scaffold1 |
| 1698854 | 1701570 | 2619016 | 2621720 | | 2717 | 2705 | 89.03 | sca_3_chr4_2_0 | Scaffold1 |
| 1706157 | 1709295 | 2626281 | 2629432 | | 3139 | 3152 | 89.85 | sca_3_chr4_2_0 | Scaffold1 |
| 1716858 | 1719161 | 2636756 | 2639014 | | 2304 | 2259 | 88.14 | sca_3_chr4_2_0 | Scaffold1 |
| 1786184 | 1789266 | 2706108 | 2709119 | | 3083 | 3012 | 89.53 | sca_3_chr4_2_0 | Scaffold1 |
| 1793924 | 1794632 | 2713757 | 2714448 | | 709 | 692 | 86.94 | sca_3_chr4_2_0 | Scaffold1 |
| 1862127 | 1862849 | 2780724 | 2781446 | | 723 | 723 | 94.88 | sca_3_chr4_2_0 | Scaffold1 |
| 1890483 | 1892226 | 2808090 | 2809841 | | 1744 | 1752 | 90.02 | sca_3_chr4_2_0 | Scaffold1 |
| 1946998 | 1948428 | 2864054 | 2865496 | | 1431 | 1443 | 88.14 | sca_3_chr4_2_0 | Scaffold1 |
| 1955908 | 1956053 | 2873026 | 2873171 | | 146 | 146 | 97.95 | sca_3_chr4_2_0 | Scaffold1 |
| 1957729 | 1962645 | 2874878 | 2879786 | | 4917 | 4909 | 86.97 | sca_3_chr4_2_0 | Scaffold1 |
| 1979946 | 1983178 | 2897001 | 2900229 | | 3233 | 3229 | 90.44 | sca_3_chr4_2_0 | Scaffold1 |
| 2004921 | 2009312 | 2921938 | 2926377 | | 4392 | 4440 | 89.1 | sca_3_chr4_2_0 | Scaffold1 |
| 2009482 | 2011681 | 2926522 | 2928713 | | 2200 | 2192 | 90.54 | sca_3_chr4_2_0 | Scaffold1 |
| 2019515 | 2021493 | 2935723 | 2937732 | | 1979 | 2010 | 90 | sca_3_chr4_2_0 | Scaffold1 |
| 2035731 | 2037499 | 2952036 | 2953791 | | 1769 | 1756 | 89.36 | sca_3_chr4_2_0 | Scaffold1 |
| 2037640 | 2037823 | 2953906 | 2954090 | | 184 | 185 | 94.15 | sca_3_chr4_2_0 | Scaffold1 |
| 2037945 | 2038161 | 2954208 | 2954419 | | 217 | 212 | 94.01 | sca_3_chr4_2_0 | Scaffold1 |
| 2059895 | 2060075 | 2975675 | 2975856 | | 181 | 182 | 96.7 | sca_3_chr4_2_0 | Scaffold1 |
| 2060179 | 2063187 | 2976291 | 2979302 | | 3009 | 3012 | 88.41 | sca_3_chr4_2_0 | Scaffold1 |
| 2134287 | 2138888 | 3036531 | 3041129 | | 4602 | 4599 | 91.98 | sca_3_chr4_2_0 | Scaffold1 |
| 2208443 | 2208889 | 3105908 | 3106345 | | 447 | 438 | 86.75 | sca_3_chr4_2_0 | Scaffold1 |
| 2227494 | 2227670 | 3124591 | 3124769 | | 177 | 179 | 96.67 | sca_3_chr4_2_0 | Scaffold1 |
| 2249979 | 2251982 | 3146699 | 3148697 | | 2004 | 1999 | 89.92 | sca_3_chr4_2_0 | Scaffold1 |
| 2302402 | 2306760 | 3198636 | 3202969 | | 4359 | 4334 | 89.23 | sca_3_chr4_2_0 | Scaffold1 |
| 2334204 | 2339477 | 3229562 | 3234872 | | 5274 | 5311 | 88.74 | sca_3_chr4_2_0 | Scaffold1 |
| 2349754 | 2350471 | 3244958 | 3245681 | | 718 | 724 | 89.24 | sca_3_chr4_2_0 | Scaffold1 |
| 2357983 | 2361978 | 3252958 | 3256939 | | 3996 | 3982 | 86.97 | sca_3_chr4_2_0 | Scaffold1 |
| 2363033 | 2363560 | 3257905 | 3258451 | | 528 | 547 | 90.96 | sca_3_chr4_2_0 | Scaffold1 |
| 2381499 | 2383257 | 3275899 | 3277668 | | 1759 | 1770 | 91.29 | sca_3_chr4_2_0 | Scaffold1 |
| 2386939 | 2387199 | 3281390 | 3281666 | | 261 | 277 | 89.21 | sca_3_chr4_2_0 | Scaffold1 |
| 2387464 | 2390950 | 3281847 | 3285336 | | 3487 | 3490 | 91.76 | sca_3_chr4_2_0 | Scaffold1 |
| 2415335 | 2416321 | 3306880 | 3307889 | | 987 | 1010 | 86.89 | sca_3_chr4_2_0 | Scaffold1 |
| 2440115 | 2440556 | 3331485 | 3331950 | | 442 | 466 | 91.42 | sca_3_chr4_2_0 | Scaffold1 |
| 2497755 | 2501383 | 3379123 | 3382765 | | 3629 | 3643 | 92.08 | sca_3_chr4_2_0 | Scaffold1 |
| 2502691 | 2503864 | 3384035 | 3385212 | | 1174 | 1178 | 85.68 | sca_3_chr4_2_0 | Scaffold1 |
| 2504229 | 2512357 | 3385575 | 3393674 | | 8129 | 8100 | 90.06 | sca_3_chr4_2_0 | Scaffold1 |
| 2557247 | 2558196 | 3434808 | 3435735 | | 950 | 928 | 90.19 | sca_3_chr4_2_0 | Scaffold1 |
| 2585869 | 2587973 | 3461279 | 3463404 | | 2105 | 2126 | 87.51 | sca_3_chr4_2_0 | Scaffold1 |
| 2588132 | 2590748 | 3463557 | 3466171 | | 2617 | 2615 | 91.8 | sca_3_chr4_2_0 | Scaffold1 |
| 2616990 | 2617815 | 3492735 | 3493533 | | 826 | 799 | 85.54 | sca_3_chr4_2_0 | Scaffold1 |
| 2727036 | 2727206 | 3600042 | 3600208 | | 171 | 167 | 94.19 | sca_3_chr4_2_0 | Scaffold1 |
| 2740960 | 2742227 | 3613783 | 3615054 | | 1268 | 1272 | 86.93 | sca_3_chr4_2_0 | Scaffold1 |
| 2745090 | 2748303 | 3617753 | 3620947 | | 3214 | 3195 | 92.33 | sca_3_chr4_2_0 | Scaffold1 |
| 2754231 | 2756633 | 3626780 | 3629176 | | 2403 | 2397 | 91.47 | sca_3_chr4_2_0 | Scaffold1 |
| 2817596 | 2822148 | 3696516 | 3701052 | | 4553 | 4537 | 87.55 | sca_3_chr4_2_0 | Scaffold1 |
| 2922489 | 2925579 | 3803022 | 3806047 | | 3091 | 3026 | 87.52 | sca_3_chr4_2_0 | Scaffold1 |
| 2931503 | 2932605 | 3812274 | 3813365 | | 1103 | 1092 | 89.5 | sca_3_chr4_2_0 | Scaffold1 |
| 2933862 | 2936359 | 3814503 | 3817006 | | 2498 | 2504 | 89.94 | sca_3_chr4_2_0 | Scaffold1 |
| 3039724 | 3042466 | 3919656 | 3922362 | | 2743 | 2707 | 90.28 | sca_3_chr4_2_0 | Scaffold1 |
| 3043279 | 3044046 | 3923189 | 3923966 | | 768 | 778 | 91.53 | sca_3_chr4_2_0 | Scaffold1 |
| 3045376 | 3046497 | 3925375 | 3926497 | | 1122 | 1123 | 89.55 | sca_3_chr4_2_0 | Scaffold1 |
| 3087099 | 3091018 | 3966114 | 3970054 | | 3920 | 3941 | 89.68 | sca_3_chr4_2_0 | Scaffold1 |
| 3093234 | 3093987 | 3972223 | 3972957 | | 754 | 735 | 85.42 | sca_3_chr4_2_0 | Scaffold1 |
| 3135913 | 3139975 | 4012231 | 4016330 | | 4063 | 4100 | 87.08 | sca_3_chr4_2_0 | Scaffold1 |
| 3188130 | 3190289 | 4064101 | 4066281 | | 2160 | 2181 | 86.31 | sca_3_chr4_2_0 | Scaffold1 |
|  |  |  |  | |  |  |  |  |  |
| 317365 | 322932 | 130474 | 135952 | | 5568 | 5479 | 92.22 | sca_32_chr11_3_0 | Scaffold41 |
| 115326 | 115451 | 394105 | 394230 | | 126 | 126 | 98.41 | sca_36_chr11_2_0 | Scaffold10 |
| 229308 | 229806 | 379005 | 378506 | | 499 | 500 | 88.05 | sca_36_chr11_2_0 | Scaffold15 |
|  |  |  |  | |  |  |  |  |  |
| 80891 | 82721 | 104079 | 102273 | | 1831 | 1807 | 87.29 | sca_37_chr6_2_0 | Scaffold27 |
| 261973 | 263762 | 1022038 | 1020262 | | 1790 | 1777 | 89.28 | sca_37_chr6_2_0 | Scaffold12 |
| 293681 | 294810 | 988966 | 987831 | | 1130 | 1136 | 88.7 | sca_37_chr6_2_0 | Scaffold12 |
| 331219 | 331337 | 310532 | 310650 | | 119 | 119 | 98.32 | sca_37_chr6_2_0 | Scaffold11 |
| 347397 | 347982 | 929063 | 928496 | | 586 | 568 | 87.31 | sca_37_chr6_2_0 | Scaffold12 |
| 377314 | 381738 | 900296 | 895881 | | 4425 | 4416 | 88.19 | sca_37_chr6_2_0 | Scaffold12 |
| 386222 | 387086 | 891148 | 890277 | | 865 | 872 | 85.84 | sca_37_chr6_2_0 | Scaffold12 |
|  |  |  |  | |  |  |  |  |  |
| 158755 | 158858 | 831724 | 831827 | | 104 | 104 | 99.04 | sca_41_chr12_2_0 | Scaffold4 |
|  |  |  |  | |  |  |  |  |  |
| 117995 | 125876 | 173341 | 165465 | | 7882 | 7877 | 89.83 | sca_44_chr3_2_0 | Scaffold22 |
| 133908 | 134689 | 157589 | 156810 | | 782 | 780 | 87.75 | sca_44_chr3_2_0 | Scaffold22 |
|  |  |  |  | |  |  |  |  |  |
| 85401 | 85630 | 121020 | 120791 | | 230 | 230 | 97.39 | sca_45_unmapped | Scaffold54 |
|  |  |  |  | |  |  |  |  |  |
| 36233 | 37572 | 1178503 | 1179842 | | 1340 | 1340 | 91.09 | sca_5_chr5_3_0 | Scaffold2 |
| 43320 | 45259 | 1185558 | 1187492 | | 1940 | 1935 | 93.25 | sca_5_chr5_3_0 | Scaffold2 |
| 49561 | 53602 | 1191800 | 1195822 | | 4042 | 4023 | 88.95 | sca_5_chr5_3_0 | Scaffold2 |
| 54300 | 55831 | 1196516 | 1198045 | | 1532 | 1530 | 92.87 | sca_5_chr5_3_0 | Scaffold2 |
| 56163 | 64719 | 1198395 | 1206961 | | 8557 | 8567 | 89.32 | sca_5_chr5_3_0 | Scaffold2 |
| 110362 | 110886 | 1251529 | 1252077 | | 525 | 549 | 86.43 | sca_5_chr5_3_0 | Scaffold2 |
| 122901 | 123725 | 1263980 | 1264791 | | 825 | 812 | 85.61 | sca_5_chr5_3_0 | Scaffold2 |
| 133772 | 137621 | 1274658 | 1278529 | | 3850 | 3872 | 87.38 | sca_5_chr5_3_0 | Scaffold2 |
| 171530 | 172778 | 1311931 | 1313146 | | 1249 | 1216 | 87.23 | sca_5_chr5_3_0 | Scaffold2 |
| 179722 | 181844 | 1334962 | 1337071 | | 2123 | 2110 | 87.66 | sca_5_chr5_3_0 | Scaffold2 |
| 202133 | 203623 | 1357495 | 1358996 | | 1491 | 1502 | 92.69 | sca_5_chr5_3_0 | Scaffold2 |
| 238692 | 243721 | 1393118 | 1398080 | | 5030 | 4963 | 84.73 | sca_5_chr5_3_0 | Scaffold2 |
| 300569 | 303801 | 1449511 | 1452715 | | 3233 | 3205 | 89.53 | sca_5_chr5_3_0 | Scaffold2 |
| 325669 | 327249 | 1473452 | 1475012 | | 1581 | 1561 | 87.06 | sca_5_chr5_3_0 | Scaffold2 |
| 334023 | 334779 | 1481790 | 1482577 | | 757 | 788 | 86.74 | sca_5_chr5_3_0 | Scaffold2 |
| 335540 | 341250 | 1483373 | 1489183 | | 5711 | 5811 | 90.65 | sca_5_chr5_3_0 | Scaffold2 |
| 366256 | 367847 | 1513291 | 1514890 | | 1592 | 1600 | 89.09 | sca_5_chr5_3_0 | Scaffold2 |
| 371147 | 373225 | 1517834 | 1519920 | | 2079 | 2087 | 86.51 | sca_5_chr5_3_0 | Scaffold2 |
| 383151 | 385216 | 1529326 | 1531376 | | 2066 | 2051 | 88.46 | sca_5_chr5_3_0 | Scaffold2 |
| 385376 | 388218 | 1531536 | 1534387 | | 2843 | 2852 | 87.1 | sca_5_chr5_3_0 | Scaffold2 |
| 398179 | 398372 | 2060061 | 2059868 | | 194 | 194 | 93.33 | sca_5_chr5_3_0 | Scaffold2 |
| 449369 | 449853 | 2013707 | 2013231 | | 485 | 477 | 91.04 | sca_5_chr5_3_0 | Scaffold2 |
| 473081 | 473980 | 1988503 | 1987607 | | 900 | 897 | 91.01 | sca_5_chr5_3_0 | Scaffold2 |
| 533927 | 534207 | 1932694 | 1932421 | | 281 | 274 | 90.14 | sca_5_chr5_3_0 | Scaffold2 |
| 549924 | 552417 | 1917032 | 1914512 | | 2494 | 2521 | 87.1 | sca_5_chr5_3_0 | Scaffold2 |
| 554725 | 559635 | 1912258 | 1907341 | | 4911 | 4918 | 87.92 | sca_5_chr5_3_0 | Scaffold2 |
| 576516 | 580774 | 1890690 | 1886386 | | 4259 | 4305 | 90.32 | sca_5_chr5_3_0 | Scaffold2 |
| 602994 | 605779 | 1864528 | 1861729 | | 2786 | 2800 | 89.78 | sca_5_chr5_3_0 | Scaffold2 |
| 608795 | 609047 | 1858952 | 1858698 | | 253 | 255 | 96.86 | sca_5_chr5_3_0 | Scaffold2 |
| 621530 | 624249 | 1846145 | 1843402 | | 2720 | 2744 | 91.49 | sca_5_chr5_3_0 | Scaffold2 |
| 638925 | 639717 | 1829157 | 1828369 | | 793 | 789 | 94.23 | sca_5_chr5_3_0 | Scaffold2 |
| 676604 | 676807 | 1792597 | 1792394 | | 204 | 204 | 92.65 | sca_5_chr5_3_0 | Scaffold2 |
| 682340 | 684932 | 1786770 | 1784177 | | 2593 | 2594 | 90.95 | sca_5_chr5_3_0 | Scaffold2 |
| 708489 | 709246 | 1761148 | 1760386 | | 758 | 763 | 88.41 | sca_5_chr5_3_0 | Scaffold2 |
| 724539 | 725497 | 1747352 | 1746399 | | 959 | 954 | 88.43 | sca_5_chr5_3_0 | Scaffold2 |
| 804716 | 809780 | 1668069 | 1662997 | | 5065 | 5073 | 89.92 | sca_5_chr5_3_0 | Scaffold2 |
| 824567 | 827954 | 1648519 | 1645141 | | 3388 | 3379 | 85.05 | sca_5_chr5_3_0 | Scaffold2 |
| 835425 | 836047 | 1637760 | 1637146 | | 623 | 615 | 88.19 | sca_5_chr5_3_0 | Scaffold2 |
| 863619 | 866784 | 1609760 | 1606624 | | 3166 | 3137 | 90.5 | sca_5_chr5_3_0 | Scaffold2 |
| 866819 | 867099 | 1606443 | 1606163 | | 281 | 281 | 98.22 | sca_5_chr5_3_0 | Scaffold2 |
| 953135 | 959099 | 2087207 | 2093189 | | 5965 | 5983 | 87.03 | sca_5_chr5_3_0 | Scaffold2 |
| 990367 | 990983 | 2131982 | 2132589 | | 617 | 608 | 91.16 | sca_5_chr5_3_0 | Scaffold2 |
| 1125292 | 1127366 | 2265782 | 2267846 | | 2075 | 2065 | 86.8 | sca_5_chr5_3_0 | Scaffold2 |
| 1159584 | 1161976 | 2299697 | 2302102 | | 2393 | 2406 | 84.97 | sca_5_chr5_3_0 | Scaffold2 |
| 1162299 | 1163937 | 2302458 | 2304126 | | 1639 | 1669 | 87.55 | sca_5_chr5_3_0 | Scaffold2 |
| 1164440 | 1164857 | 2304642 | 2305055 | | 418 | 414 | 90.05 | sca_5_chr5_3_0 | Scaffold2 |
| 1165451 | 1168791 | 2305597 | 2308843 | | 3341 | 3247 | 87.8 | sca_5_chr5_3_0 | Scaffold2 |
| 1168974 | 1171254 | 2309010 | 2311218 | | 2281 | 2209 | 86.61 | sca_5_chr5_3_0 | Scaffold2 |
| 1198071 | 1200052 | 2337051 | 2339009 | | 1982 | 1959 | 90.83 | sca_5_chr5_3_0 | Scaffold2 |
| 1218651 | 1218927 | 2360183 | 2360438 | | 277 | 256 | 88.89 | sca_5_chr5_3_0 | Scaffold2 |
| 1330065 | 1332352 | 2471757 | 2474026 | | 2288 | 2270 | 84.68 | sca_5_chr5_3_0 | Scaffold2 |
| 1365540 | 1366851 | 2505949 | 2507264 | | 1312 | 1316 | 91.6 | sca_5_chr5_3_0 | Scaffold2 |
| 1384601 | 1386874 | 2524376 | 2526664 | | 2274 | 2289 | 92.03 | sca_5_chr5_3_0 | Scaffold2 |
| 1395635 | 1400308 | 2535280 | 2539981 | | 4674 | 4702 | 89.12 | sca_5_chr5_3_0 | Scaffold2 |
| 1411474 | 1413361 | 2550800 | 2552654 | | 1888 | 1855 | 86.95 | sca_5_chr5_3_0 | Scaffold2 |
| 1425987 | 1427067 | 2565151 | 2566255 | | 1081 | 1105 | 87.15 | sca_5_chr5_3_0 | Scaffold2 |
| 1434388 | 1435905 | 2574103 | 2575600 | | 1518 | 1498 | 88.7 | sca_5_chr5_3_0 | Scaffold2 |
| 1450331 | 1452028 | 2589969 | 2591674 | | 1698 | 1706 | 86.15 | sca_5_chr5_3_0 | Scaffold2 |
| 1464576 | 1464961 | 2604033 | 2604422 | | 386 | 390 | 91.48 | sca_5_chr5_3_0 | Scaffold2 |
| 1518530 | 1520495 | 2657338 | 2659312 | | 1966 | 1975 | 86.86 | sca_5_chr5_3_0 | Scaffold2 |
| 1556057 | 1559232 | 35861 | 32672 | | 3176 | 3190 | 86.52 | sca_5_chr5_3_0 | Scaffold20 |
| 1571236 | 1572212 | 22827 | 21883 | | 977 | 945 | 86.02 | sca_5_chr5_3_0 | Scaffold20 |
| 1574729 | 1575099 | 19457 | 19088 | | 371 | 370 | 84.24 | sca_5_chr5_3_0 | Scaffold20 |
| 1699741 | 1700973 | 163571 | 164830 | | 1233 | 1260 | 93.74 | sca_5_chr5_3_0 | Scaffold20 |
| 1724026 | 1727812 | 187936 | 191743 | | 3787 | 3808 | 88.79 | sca_5_chr5_3_0 | Scaffold20 |
| 1760935 | 1762768 | 224927 | 226743 | | 1834 | 1817 | 88.94 | sca_5_chr5_3_0 | Scaffold20 |
| 1823277 | 1825598 | 288539 | 290870 | | 2322 | 2332 | 89.52 | sca_5_chr5_3_0 | Scaffold20 |
| 1850634 | 1855792 | 314639 | 319841 | | 5159 | 5203 | 87.67 | sca_5_chr5_3_0 | Scaffold20 |
| 1915569 | 1915723 | 373932 | 374087 | | 155 | 156 | 92.95 | sca_5_chr5_3_0 | Scaffold20 |
| 2003839 | 2008651 | 459676 | 464493 | | 4813 | 4818 | 89.52 | sca_5_chr5_3_0 | Scaffold20 |
| 2019635 | 2023272 | 474919 | 478517 | | 3638 | 3599 | 89.72 | sca_5_chr5_3_0 | Scaffold20 |
| 2023406 | 2024867 | 478619 | 480085 | | 1462 | 1467 | 88.12 | sca_5_chr5_3_0 | Scaffold20 |
| 2115247 | 2116003 | 46481 | 47237 | | 757 | 757 | 96.04 | sca_5_chr5_3_0 | Scaffold71 |
| 2120873 | 2121064 | 54316 | 54508 | | 192 | 193 | 95.85 | sca_5_chr5_3_0 | Scaffold58 |
| 2120873 | 2121064 | 35958 | 36149 | | 192 | 192 | 98.96 | sca_5_chr5_3_0 | Scaffold69 |
| 2161851 | 2162027 | 71981 | 71805 | | 177 | 177 | 96.05 | sca_5_chr5_3_0 | Scaffold28 |
| 2333852 | 2337158 | 204027 | 200722 | | 3307 | 3306 | 85.37 | sca_5_chr5_3_0 | Scaffold32 |
| 2479861 | 2485426 | 80995 | 86601 | | 5566 | 5607 | 89.13 | sca_5_chr5_3_0 | Scaffold32 |
|  |  |  |  | |  |  |  |  |  |
| 39333 | 42837 | 832725 | 829223 | | 3505 | 3503 | 88.05 | sca_53_chr6_3_0 | Scaffold12 |
| 43576 | 44498 | 828594 | 827659 | | 923 | 936 | 89.58 | sca_53_chr6_3_0 | Scaffold12 |
| 71663 | 72665 | 801390 | 800384 | | 1003 | 1007 | 91.86 | sca_53_chr6_3_0 | Scaffold12 |
| 96288 | 97679 | 778147 | 776747 | | 1392 | 1401 | 90.03 | sca_53_chr6_3_0 | Scaffold12 |
| 122056 | 124172 | 752224 | 750108 | | 2117 | 2117 | 91.35 | sca_53_chr6_3_0 | Scaffold12 |
| 147656 | 150814 | 720863 | 717711 | | 3159 | 3153 | 90.32 | sca_53_chr6_3_0 | Scaffold12 |
| 172911 | 173043 | 675370 | 675238 | | 133 | 133 | 100 | sca_53_chr6_3_0 | Scaffold1 |
| 179988 | 183964 | 689179 | 685169 | | 3977 | 4011 | 90.42 | sca_53_chr6_3_0 | Scaffold12 |
|  |  |  |  | |  |  |  |  |  |
| 38387 | 39074 | 201974 | 201285 | | 688 | 690 | 91.62 | sca_54_chr3_4_0 | Scaffold16 |
| 74831 | 79339 | 169433 | 164864 | | 4509 | 4570 | 87.48 | sca_54_chr3_4_0 | Scaffold16 |
| 80006 | 80774 | 164129 | 163384 | | 769 | 746 | 85.64 | sca_54_chr3_4_0 | Scaffold16 |
| 105341 | 106720 | 152641 | 153994 | | 1380 | 1354 | 87.32 | sca_54_chr3_4_0 | Scaffold16 |
| 120541 | 120848 | 125109 | 124806 | | 308 | 304 | 95.79 | sca_54_chr3_4_0 | Scaffold16 |
| 174473 | 176817 | 73068 | 70707 | | 2345 | 2362 | 88.82 | sca_54_chr3_4_0 | Scaffold16 |
|  |  |  |  | |  |  |  |  |  |
| 44517 | 45045 | 564268 | 563738 | | 529 | 531 | 94.35 | sca_55_chr13_4_0 | Scaffold1 |
| 51615 | 52019 | 557296 | 556895 | | 405 | 402 | 95.07 | sca_55_chr13_4_0 | Scaffold1 |
| 55166 | 55619 | 553757 | 553307 | | 454 | 451 | 94.97 | sca_55_chr13_4_0 | Scaffold1 |
| 55758 | 56046 | 553163 | 552875 | | 289 | 289 | 91.07 | sca_55_chr13_4_0 | Scaffold1 |
|  |  |  |  | |  |  |  |  |  |
| 13876 | 13979 | 831827 | 831724 | | 104 | 104 | 100 | sca_56_chr12_3_0 | Scaffold4 |
| 179243 | 179369 | 587186 | 587061 | | 127 | 126 | 99.21 | sca_56_chr12_3_0 | Scaffold16 |
|  |  |  |  | |  |  |  |  |  |
| 138393 | 138524 | 536921 | 537053 | | 132 | 133 | 99.25 | sca_58_chr7_9_0 | Scaffold16 |
| 138394 | 138518 | 4426671 | 4426547 | | 125 | 125 | 100 | sca_58_chr7_9_0 | Scaffold1 |
| 138394 | 138519 | 111555 | 111430 | | 126 | 126 | 100 | sca_58_chr7_9_0 | Scaffold38 |
| 138394 | 138519 | 50309 | 50184 | | 126 | 126 | 100 | sca_58_chr7_9_0 | Scaffold64 |
| 144774 | 144897 | 426334 | 426458 | | 124 | 125 | 96 | sca_58_chr7_9_0 | Scaffold5 |
|  |  |  |  | |  |  |  |  |  |
| 31662 | 31869 | 19193 | 18985 | | 208 | 209 | 94.26 | sca_59_chr4_1_0 | Scaffold100 |
| 31674 | 31865 | 29131 | 28940 | | 192 | 192 | 95.83 | sca_59_chr4_1_0 | Scaffold58 |
| 55424 | 58828 | 601680 | 605028 | | 3405 | 3349 | 89 | sca_59_chr4_1_0 | Scaffold1 |
| 61140 | 66968 | 607182 | 613016 | | 5829 | 5835 | 90.45 | sca_59_chr4_1_0 | Scaffold1 |
| 122892 | 124360 | 673636 | 675090 | | 1469 | 1455 | 85.41 | sca_59_chr4_1_0 | Scaffold1 |
| 129197 | 133555 | 681833 | 686141 | | 4359 | 4309 | 89.54 | sca_59_chr4_1_0 | Scaffold1 |
| 140091 | 143790 | 692822 | 696531 | | 3700 | 3710 | 91.11 | sca_59_chr4_1_0 | Scaffold1 |
|  |  |  |  | |  |  |  |  |  |
| 34679 | 36262 | 1765448 | 1763911 | | 1584 | 1538 | 92.26 | sca_6_chr9_2_0 | Scaffold3 |
| 37881 | 38469 | 1762370 | 1761762 | | 589 | 609 | 86.17 | sca_6_chr9_2_0 | Scaffold3 |
| 54407 | 61151 | 1746670 | 1740009 | | 6745 | 6662 | 90.06 | sca_6_chr9_2_0 | Scaffold3 |
| 65922 | 68093 | 1735241 | 1733063 | | 2172 | 2179 | 92.86 | sca_6_chr9_2_0 | Scaffold3 |
| 93896 | 97386 | 1697972 | 1694474 | | 3491 | 3499 | 89.79 | sca_6_chr9_2_0 | Scaffold3 |
| 135404 | 140365 | 1657771 | 1652794 | | 4962 | 4978 | 90.5 | sca_6_chr9_2_0 | Scaffold3 |
| 168470 | 172036 | 1630462 | 1626899 | | 3567 | 3564 | 86.74 | sca_6_chr9_2_0 | Scaffold3 |
| 176602 | 179675 | 1622308 | 1619273 | | 3074 | 3036 | 86.76 | sca_6_chr9_2_0 | Scaffold3 |
| 207262 | 209201 | 1591738 | 1589778 | | 1940 | 1961 | 87.26 | sca_6_chr9_2_0 | Scaffold3 |
| 244466 | 246616 | 1502175 | 1500037 | | 2151 | 2139 | 88.01 | sca_6_chr9_2_0 | Scaffold3 |
| 256600 | 262554 | 1490131 | 1484084 | | 5955 | 6048 | 88.57 | sca_6_chr9_2_0 | Scaffold3 |
| 264811 | 268836 | 1481850 | 1477844 | | 4026 | 4007 | 89.11 | sca_6_chr9_2_0 | Scaffold3 |
| 287227 | 287837 | 1447682 | 1448267 | | 611 | 586 | 85.81 | sca_6_chr9_2_0 | Scaffold3 |
| 330577 | 339720 | 1418904 | 1409825 | | 9144 | 9080 | 86.45 | sca_6_chr9_2_0 | Scaffold3 |
| 360709 | 366901 | 1389603 | 1383372 | | 6193 | 6232 | 90.28 | sca_6_chr9_2_0 | Scaffold3 |
| 368665 | 370220 | 1381609 | 1380036 | | 1556 | 1574 | 90.7 | sca_6_chr9_2_0 | Scaffold3 |
| 373437 | 374191 | 1376859 | 1376101 | | 755 | 759 | 88.27 | sca_6_chr9_2_0 | Scaffold3 |
| 414409 | 418502 | 1334786 | 1330675 | | 4094 | 4112 | 86.93 | sca_6_chr9_2_0 | Scaffold3 |
| 420810 | 421309 | 1328401 | 1327892 | | 500 | 510 | 87.67 | sca_6_chr9_2_0 | Scaffold3 |
| 423880 | 427300 | 1325319 | 1321894 | | 3421 | 3426 | 90 | sca_6_chr9_2_0 | Scaffold3 |
| 449501 | 452224 | 1299950 | 1297240 | | 2724 | 2711 | 91.22 | sca_6_chr9_2_0 | Scaffold3 |
| 489333 | 490858 | 1254202 | 1252676 | | 1526 | 1527 | 88.05 | sca_6_chr9_2_0 | Scaffold3 |
| 491930 | 495150 | 1251624 | 1248357 | | 3221 | 3268 | 87.49 | sca_6_chr9_2_0 | Scaffold3 |
| 513489 | 514668 | 1229324 | 1228138 | | 1180 | 1187 | 90.19 | sca_6_chr9_2_0 | Scaffold3 |
| 517619 | 519028 | 1225027 | 1223596 | | 1410 | 1432 | 90.24 | sca_6_chr9_2_0 | Scaffold3 |
| 523193 | 523439 | 1219481 | 1219245 | | 247 | 237 | 87.95 | sca_6_chr9_2_0 | Scaffold3 |
| 565784 | 568714 | 1168034 | 1170912 | | 2931 | 2879 | 88.76 | sca_6_chr9_2_0 | Scaffold3 |
| 572757 | 573067 | 1174879 | 1175199 | | 311 | 321 | 85.24 | sca_6_chr9_2_0 | Scaffold3 |
| 592222 | 593299 | 1154508 | 1153421 | | 1078 | 1088 | 89.09 | sca_6_chr9_2_0 | Scaffold3 |
| 625977 | 627695 | 1121732 | 1120023 | | 1719 | 1710 | 91.81 | sca_6_chr9_2_0 | Scaffold3 |
| 644794 | 645692 | 1102723 | 1101779 | | 899 | 945 | 90.92 | sca_6_chr9_2_0 | Scaffold3 |
| 646759 | 648180 | 1100767 | 1099340 | | 1422 | 1428 | 91.85 | sca_6_chr9_2_0 | Scaffold3 |
| 764585 | 769657 | 983374 | 978349 | | 5073 | 5026 | 89.76 | sca_6_chr9_2_0 | Scaffold3 |
| 770691 | 772919 | 977306 | 975092 | | 2229 | 2215 | 90.45 | sca_6_chr9_2_0 | Scaffold3 |
| 793978 | 796517 | 954616 | 952088 | | 2540 | 2529 | 92.49 | sca_6_chr9_2_0 | Scaffold3 |
| 852039 | 852391 | 896818 | 896479 | | 353 | 340 | 91.36 | sca_6_chr9_2_0 | Scaffold3 |
| 852504 | 853612 | 896381 | 895263 | | 1109 | 1119 | 87.3 | sca_6_chr9_2_0 | Scaffold3 |
| 876047 | 879092 | 873567 | 870555 | | 3046 | 3013 | 88.57 | sca_6_chr9_2_0 | Scaffold3 |
| 913818 | 914113 | 836371 | 836073 | | 296 | 299 | 87.83 | sca_6_chr9_2_0 | Scaffold3 |
| 940931 | 941710 | 808852 | 808051 | | 780 | 802 | 92.58 | sca_6_chr9_2_0 | Scaffold3 |
| 943844 | 944845 | 805738 | 804733 | | 1002 | 1006 | 90.66 | sca_6_chr9_2_0 | Scaffold3 |
| 946934 | 950131 | 802789 | 799636 | | 3198 | 3154 | 86.74 | sca_6_chr9_2_0 | Scaffold3 |
| 968720 | 971932 | 781213 | 777983 | | 3213 | 3231 | 91.66 | sca_6_chr9_2_0 | Scaffold3 |
| 982100 | 986304 | 769320 | 765087 | | 4205 | 4234 | 86.01 | sca_6_chr9_2_0 | Scaffold3 |
| 1082427 | 1082988 | 674289 | 673718 | | 562 | 572 | 90.5 | sca_6_chr9_2_0 | Scaffold3 |
| 1096226 | 1101230 | 660712 | 655690 | | 5005 | 5023 | 91.85 | sca_6_chr9_2_0 | Scaffold3 |
| 1101359 | 1101694 | 655582 | 655273 | | 336 | 310 | 89.64 | sca_6_chr9_2_0 | Scaffold3 |
| 1134354 | 1137667 | 622696 | 619396 | | 3314 | 3301 | 90.77 | sca_6_chr9_2_0 | Scaffold3 |
| 1176598 | 1176938 | 581468 | 581132 | | 341 | 337 | 91.23 | sca_6_chr9_2_0 | Scaffold3 |
| 1195450 | 1197512 | 562822 | 560775 | | 2063 | 2048 | 91.16 | sca_6_chr9_2_0 | Scaffold3 |
| 1329253 | 1332685 | 433261 | 429827 | | 3433 | 3435 | 88.88 | sca_6_chr9_2_0 | Scaffold3 |
| 1333858 | 1334805 | 428723 | 427780 | | 948 | 944 | 90.1 | sca_6_chr9_2_0 | Scaffold3 |
| 1456261 | 1458221 | 95278 | 93329 | | 1961 | 1950 | 87.42 | sca_6_chr9_2_0 | Scaffold21 |
| 1559139 | 1559943 | 221049 | 221860 | | 805 | 812 | 91.47 | sca_6_chr9_2_0 | Scaffold3 |
| 1600566 | 1601461 | 261932 | 262827 | | 896 | 896 | 92.88 | sca_6_chr9_2_0 | Scaffold3 |
| 1633276 | 1635658 | 293604 | 295957 | | 2383 | 2354 | 87.59 | sca_6_chr9_2_0 | Scaffold3 |
| 1650715 | 1654075 | 310432 | 313776 | | 3361 | 3345 | 88.84 | sca_6_chr9_2_0 | Scaffold3 |
| 1788630 | 1792702 | 206811 | 210787 | | 4073 | 3977 | 89.54 | sca_6_chr9_2_0 | Scaffold21 |
| 1802277 | 1807812 | 220052 | 225603 | | 5536 | 5552 | 88.99 | sca_6_chr9_2_0 | Scaffold21 |
| 1811795 | 1814792 | 229667 | 232678 | | 2998 | 3012 | 88.11 | sca_6_chr9_2_0 | Scaffold21 |
| 1842066 | 1843933 | 259336 | 261197 | | 1868 | 1862 | 91.03 | sca_6_chr9_2_0 | Scaffold21 |
| 1879496 | 1881160 | 288760 | 290390 | | 1665 | 1631 | 84.11 | sca_6_chr9_2_0 | Scaffold21 |
| 1934596 | 1935087 | 341923 | 342392 | | 492 | 470 | 83.56 | sca_6_chr9_2_0 | Scaffold21 |
| 1940048 | 1940778 | 347100 | 347812 | | 731 | 713 | 87.99 | sca_6_chr9_2_0 | Scaffold21 |
| 1950798 | 1952232 | 357477 | 358905 | | 1435 | 1429 | 87.79 | sca_6_chr9_2_0 | Scaffold21 |
| 1957267 | 1964149 | 363781 | 370616 | | 6883 | 6836 | 87.14 | sca_6_chr9_2_0 | Scaffold21 |
| 1989907 | 1990046 | 2629418 | 2629556 | | 140 | 139 | 95 | sca_6_chr9_2_0 | Scaffold2 |
| 2007009 | 2008233 | 445960 | 444732 | | 1225 | 1229 | 89.03 | sca_6_chr9_2_0 | Scaffold21 |
| 2228722 | 2232107 | 17969 | 14587 | | 3386 | 3383 | 97.46 | sca_6_chr9_2_0 | Scaffold115 |
| 2232342 | 2232482 | 78216 | 78356 | | 141 | 141 | 97.87 | sca_6_chr9_2_0 | Scaffold41 |
| 2233693 | 2234020 | 14993 | 15320 | | 328 | 328 | 99.39 | sca_6_chr9_2_0 | Scaffold71 |
|  |  |  |  | |  |  |  |  |  |
| 6317 | 7562 | 1150901 | 1149657 | | 1246 | 1245 | 82.48 | sca_60_chr3_6_0 | Scaffold11 |
|  |  |  |  | |  |  |  |  |  |
| 92108 | 92211 | 31290 | 31187 | | 104 | 104 | 100 | sca_61_chr5_1_0 | Scaffold61 |
|  |  |  |  | |  |  |  |  |  |
| 41014 | 41381 | 18329 | 18694 | | 368 | 366 | 95.38 | sca_62_chr14_1_0 | Scaffold125 |
| 41015 | 41801 | 87729 | 88497 | | 787 | 769 | 93.91 | sca_62_chr14_1_0 | Scaffold28 |
| 65964 | 66707 | 14663 | 15404 | | 744 | 742 | 97.04 | sca_62_chr14_1_0 | Scaffold88 |
| 66888 | 68296 | 1956 | 3364 | | 1409 | 1409 | 97.8 | sca_62_chr14_1_0 | Scaffold50 |
| 116003 | 116455 | 52891 | 52435 | | 453 | 457 | 96.5 | sca_62_chr14_1_0 | Scaffold22 |
| 116608 | 120949 | 112015 | 107675 | | 4342 | 4341 | 95.95 | sca_62_chr14_1_0 | Scaffold50 |
| 49621 | 50863 | 35318 | 34087 | | 1243 | 1232 | 93.81 | sca_63_chr14_2_0 | Scaffold71 |
| 50862 | 51790 | 14183 | 15110 | | 929 | 928 | 97.95 | sca_63_chr14_2_0 | Scaffold100 |
| 55665 | 58003 | 326888 | 324546 | | 2339 | 2343 | 96.67 | sca_63_chr14_2_0 | Scaffold33 |
| 140185 | 143030 | 30474 | 27625 | | 2846 | 2850 | 98 | sca_63_chr14_2_0 | Scaffold69 |
|  |  |  |  | |  |  |  |  |  |
| 27880 | 32033 | 17293 | 13106 | | 4154 | 4188 | 92.52 | sca_64_chr4_4_0 | Scaffold54 |
|  |  |  |  | |  |  |  |  |  |
| 31156 | 31275 | 675245 | 675364 | | 120 | 120 | 100 | sca_72_chr7_6_0 | Scaffold1 |
|  |  |  |  | |  |  |  |  |  |
| 63757 | 65686 | 74466 | 76390 | | 1930 | 1925 | 89.32 | sca_73_unmapped | Scaffold33 |
|  |  |  |  | |  |  |  |  |  |
| 46075 | 50911 | 130474 | 135298 | | 4837 | 4825 | 93.49 | sca_75_chr17_2_0 | Scaffold41 |
|  |  |  |  | |  |  |  |  |  |
| 64003 | 64130 | 24380 | 24253 | | 128 | 128 | 98.44 | sca_78_chr15_2_0 | Scaffold104 |
| 64011 | 64130 | 230739 | 230620 | | 120 | 120 | 100 | sca_78_chr15_2_0 | Scaffold0 |
| 64011 | 64134 | 233594 | 233717 | | 124 | 124 | 100 | sca_78_chr15_2_0 | Scaffold33 |
| 64011 | 64133 | 2297 | 2175 | | 123 | 123 | 99.19 | sca_78_chr15_2_0 | Scaffold77 |
|  |  |  |  | |  |  |  |  |  |
| 335237 | 336885 | 1060026 | 1058389 | | 1649 | 1638 | 88.02 | sca_8_chr1_1_0 | Scaffold10 |
| 360231 | 364006 | 1035542 | 1031788 | | 3776 | 3755 | 89.04 | sca_8_chr1_1_0 | Scaffold10 |
| 408094 | 408868 | 992386 | 991585 | | 775 | 802 | 85.84 | sca_8_chr1_1_0 | Scaffold10 |
| 451485 | 454398 | 950562 | 947670 | | 2914 | 2893 | 87.24 | sca_8_chr1_1_0 | Scaffold10 |
| 498442 | 501492 | 903868 | 900841 | | 3051 | 3028 | 89.03 | sca_8_chr1_1_0 | Scaffold10 |
| 557952 | 561871 | 844119 | 840216 | | 3920 | 3904 | 85.55 | sca_8_chr1_1_0 | Scaffold10 |
| 736373 | 738482 | 673029 | 670979 | | 2110 | 2051 | 88.37 | sca_8_chr1_1_0 | Scaffold10 |
| 748791 | 749116 | 660674 | 660346 | | 326 | 329 | 92.75 | sca_8_chr1_1_0 | Scaffold10 |
| 786072 | 791281 | 624045 | 618819 | | 5210 | 5227 | 87.26 | sca_8_chr1_1_0 | Scaffold10 |
| 803585 | 807456 | 606826 | 602952 | | 3872 | 3875 | 85.65 | sca_8_chr1_1_0 | Scaffold10 |
| 821876 | 822374 | 588625 | 588130 | | 499 | 496 | 89.2 | sca_8_chr1_1_0 | Scaffold10 |
| 881019 | 884799 | 520661 | 516881 | | 3781 | 3781 | 86.54 | sca_8_chr1_1_0 | Scaffold10 |
| 906615 | 910271 | 495174 | 491497 | | 3657 | 3678 | 88.16 | sca_8_chr1_1_0 | Scaffold10 |
| 934876 | 936929 | 467520 | 465496 | | 2054 | 2025 | 90.03 | sca_8_chr1_1_0 | Scaffold10 |
| 964985 | 969014 | 436942 | 432903 | | 4030 | 4040 | 87.9 | sca_8_chr1_1_0 | Scaffold10 |
| 972050 | 975830 | 429853 | 426098 | | 3781 | 3756 | 88.54 | sca_8_chr1_1_0 | Scaffold10 |
| 985423 | 987804 | 416476 | 414061 | | 2382 | 2416 | 88.02 | sca_8_chr1_1_0 | Scaffold10 |
| 988106 | 988396 | 413768 | 413476 | | 291 | 293 | 86.1 | sca_8_chr1_1_0 | Scaffold10 |
| 994999 | 995252 | 406460 | 406211 | | 254 | 250 | 96.06 | sca_8_chr1_1_0 | Scaffold10 |
| 1027697 | 1029082 | 372310 | 370977 | | 1386 | 1334 | 85.03 | sca_8_chr1_1_0 | Scaffold10 |
| 1131802 | 1134225 | 272759 | 270304 | | 2424 | 2456 | 88.48 | sca_8_chr1_1_0 | Scaffold10 |
| 1148473 | 1151497 | 256209 | 253227 | | 3025 | 2983 | 86.66 | sca_8_chr1_1_0 | Scaffold10 |
| 1161207 | 1169239 | 240182 | 232165 | | 8033 | 8018 | 91.26 | sca_8_chr1_1_0 | Scaffold10 |
| 1203815 | 1206323 | 198910 | 196404 | | 2509 | 2507 | 92.76 | sca_8_chr1_1_0 | Scaffold10 |
| 1221771 | 1229835 | 180917 | 172898 | | 8065 | 8020 | 87.99 | sca_8_chr1_1_0 | Scaffold10 |
| 1243785 | 1244213 | 161164 | 160731 | | 429 | 434 | 88.06 | sca_8_chr1_1_0 | Scaffold10 |
| 1262224 | 1266765 | 142831 | 138275 | | 4542 | 4557 | 90.06 | sca_8_chr1_1_0 | Scaffold10 |
| 1269653 | 1270402 | 135432 | 134671 | | 750 | 762 | 90.22 | sca_8_chr1_1_0 | Scaffold10 |
| 1276736 | 1281136 | 128129 | 123729 | | 4401 | 4401 | 90.62 | sca_8_chr1_1_0 | Scaffold10 |
| 1320272 | 1321935 | 85090 | 83430 | | 1664 | 1661 | 89.31 | sca_8_chr1_1_0 | Scaffold10 |
| 1331358 | 1335957 | 74153 | 69572 | | 4600 | 4582 | 91.42 | sca_8_chr1_1_0 | Scaffold10 |
|  |  |  |  | |  |  |  |  |  |
| 153039 | 158050 | 1927883 | 1922857 | | 5012 | 5027 | 88.15 | sca_82_chr10_2_0 | Scaffold5 |
| 404851 | 404965 | 1724720 | 1724834 | | 115 | 115 | 100 | sca_82_chr10_2_0 | Scaffold5 |
| 720949 | 721454 | 1488638 | 1488164 | | 506 | 475 | 82.57 | sca_82_chr10_2_0 | Scaffold5 |
| 773742 | 778124 | 1438227 | 1433843 | | 4383 | 4385 | 88.91 | sca_82_chr10_2_0 | Scaffold5 |
| 794180 | 796547 | 1418490 | 1416112 | | 2368 | 2379 | 88.26 | sca_82_chr10_2_0 | Scaffold5 |
| 796658 | 797609 | 1415928 | 1414993 | | 952 | 936 | 90.49 | sca_82_chr10_2_0 | Scaffold5 |
| 861701 | 861913 | 1355577 | 1355368 | | 213 | 210 | 94.37 | sca_82_chr10_2_0 | Scaffold5 |
| 875511 | 877374 | 1341806 | 1339945 | | 1864 | 1862 | 92.22 | sca_82_chr10_2_0 | Scaffold5 |
| 898049 | 914346 | 1320313 | 1304035 | | 16298 | 16279 | 91.41 | sca_82_chr10_2_0 | Scaffold5 |
| 924380 | 926159 | 1294591 | 1292786 | | 1780 | 1806 | 86.75 | sca_82_chr10_2_0 | Scaffold5 |
| 938671 | 940496 | 1280644 | 1278825 | | 1826 | 1820 | 88.15 | sca_82_chr10_2_0 | Scaffold5 |
| 946447 | 947428 | 1273030 | 1272063 | | 982 | 968 | 90.24 | sca_82_chr10_2_0 | Scaffold5 |
| 982307 | 984431 | 1241147 | 1239037 | | 2125 | 2111 | 84.88 | sca_82_chr10_2_0 | Scaffold5 |
| 1033906 | 1036326 | 1190829 | 1188437 | | 2421 | 2393 | 85.92 | sca_82_chr10_2_0 | Scaffold5 |
| 1039736 | 1042718 | 1185117 | 1182119 | | 2983 | 2999 | 84.05 | sca_82_chr10_2_0 | Scaffold5 |
| 1043076 | 1044658 | 1181772 | 1180164 | | 1583 | 1609 | 90.78 | sca_82_chr10_2_0 | Scaffold5 |
| 1045676 | 1050196 | 1179124 | 1174580 | | 4521 | 4545 | 86.84 | sca_82_chr10_2_0 | Scaffold5 |
| 1059130 | 1062644 | 1165539 | 1162018 | | 3515 | 3522 | 88.43 | sca_82_chr10_2_0 | Scaffold5 |
| 1063873 | 1066358 | 1160950 | 1158474 | | 2486 | 2477 | 91.52 | sca_82_chr10_2_0 | Scaffold5 |
| 1096161 | 1096415 | 1128751 | 1128496 | | 255 | 256 | 87.12 | sca_82_chr10_2_0 | Scaffold5 |
| 1102587 | 1103772 | 1122450 | 1121263 | | 1186 | 1188 | 88.76 | sca_82_chr10_2_0 | Scaffold5 |
|  |  |  |  | |  |  |  |  |  |
| 41391 | 44084 | 182639 | 179949 | | 2694 | 2691 | 96.15 | sca_83_unmapped | Scaffold41 |
| 41528 | 44086 | 91641 | 89083 | | 2559 | 2559 | 95.78 | sca_83_unmapped | Scaffold50 |
| 45837 | 50004 | 88292 | 84114 | | 4168 | 4179 | 94.03 | sca_83_unmapped | Scaffold50 |
| 50004 | 52811 | 84006 | 81199 | | 2808 | 2808 | 95.05 | sca_83_unmapped | Scaffold50 |
| 52812 | 54810 | 81048 | 79045 | | 1999 | 2004 | 95.66 | sca_83_unmapped | Scaffold50 |
| 54815 | 56715 | 78897 | 76983 | | 1901 | 1915 | 95.15 | sca_83_unmapped | Scaffold50 |
| 57933 | 58565 | 5668 | 6299 | | 633 | 632 | 91.47 | sca_83_unmapped | Scaffold92 |
| 12836 | 13030 | 136589 | 136783 | | 195 | 195 | 96.41 | sca_87_unmapped | Scaffold41 |
| 12836 | 13029 | 4375 | 4568 | | 194 | 194 | 96.39 | sca_87_unmapped | Scaffold92 |
| 56972 | 57130 | 251772 | 251614 | | 159 | 159 | 99.37 | sca_87_unmapped | Scaffold16 |
| 56972 | 57130 | 1523902 | 1523744 | | 159 | 159 | 99.37 | sca_87_unmapped | Scaffold3 |
| 56972 | 57130 | 233561 | 233720 | | 159 | 160 | 98.75 | sca_87_unmapped | Scaffold33 |
| 56972 | 57130 | 39501 | 39343 | | 159 | 159 | 99.37 | sca_87_unmapped | Scaffold82 |
| 56973 | 57137 | 24380 | 24217 | | 165 | 164 | 93.94 | sca_87_unmapped | Scaffold104 |
| 56974 | 57114 | 761600 | 761460 | | 141 | 141 | 100 | sca_87_unmapped | Scaffold1 |
| 56974 | 57130 | 1134912 | 1135068 | | 157 | 157 | 99.36 | sca_87_unmapped | Scaffold11 |
| 56974 | 57130 | 1091787 | 1091631 | | 157 | 157 | 99.36 | sca_87_unmapped | Scaffold2 |
| 56974 | 57130 | 362477 | 362633 | | 157 | 157 | 99.36 | sca_87_unmapped | Scaffold22 |
| 56974 | 57130 | 181452 | 181608 | | 157 | 157 | 99.36 | sca_87_unmapped | Scaffold44 |
| 56974 | 57130 | 16303 | 16459 | | 157 | 157 | 99.36 | sca_87_unmapped | Scaffold62 |
| 56987 | 57134 | 761599 | 761451 | | 148 | 149 | 98.66 | sca_87_unmapped | Scaffold1 |
| 16871 | 17062 | 192896 | 192705 | | 192 | 192 | 93.23 | sca_88_unmapped | Scaffold41 |
|  |  |  |  | |  |  |  |  |  |
| 269250 | 269364 | 229114 | 229000 | | 115 | 115 | 100 | sca_9_chr7_10_0 | Scaffold16 |
| 269250 | 269378 | 111558 | 111429 | | 129 | 130 | 99.23 | sca_9_chr7_10_0 | Scaffold38 |
| 269252 | 269379 | 1794625 | 1794498 | | 128 | 128 | 100 | sca_9_chr7_10_0 | Scaffold3 |
| 269257 | 269378 | 500488 | 500367 | | 122 | 122 | 100 | sca_9_chr7_10_0 | Scaffold15 |
| 517481 | 517610 | 287985 | 288114 | | 130 | 130 | 100 | sca_9_chr7_10_0 | Scaffold17 |
| 517484 | 517613 | 675363 | 675234 | | 130 | 130 | 99.23 | sca_9_chr7_10_0 | Scaffold1 |
| 590323 | 591900 | 168080 | 169688 | | 1578 | 1609 | 85.78 | sca_9_chr7_10_0 | Scaffold38 |
|  |  |  |  | |  |  |  |  |  |
| 34126 | 34319 | 46675 | 46481 | | 194 | 195 | 96.92 | sca_93_unmapped | Scaffold71 |
| 34130 | 34319 | 192707 | 192896 | | 190 | 190 | 97.89 | sca_93_unmapped | Scaffold41 |
| 34130 | 34319 | 54506 | 54316 | | 190 | 191 | 97.38 | sca_93_unmapped | Scaffold58 |
| 34130 | 34319 | 36147 | 35958 | | 190 | 190 | 99.47 | sca_93_unmapped | Scaffold69 |
| 34130 | 34321 | 42754 | 42564 | | 192 | 191 | 98.96 | sca_93_unmapped | Scaffold91 |
| 34131 | 34319 | 23543 | 23355 | | 189 | 189 | 99.47 | sca_93_unmapped | Scaffold100 |
| 34131 | 34319 | 1117 | 929 | | 189 | 189 | 99.47 | sca_93_unmapped | Scaffold50 |
| 45343 | 49156 | 30358 | 34180 | | 3814 | 3823 | 89.85 | sca_93_unmapped | Scaffold100 |
|  |  |  |  | |  |  |  |  |  |
| 8655 | 10861 | 81835 | 84041 | | 2207 | 2207 | 91.59 | sca_94_chr7_5_0 | Scaffold41 |
|  |  |  |  | |  |  |  |  |  |
| 8075 | 8554 | 89637 | 89169 | | 480 | 469 | 93.35 | sca_98_unmapped | Scaffold58 |
| 8816 | 12248 | 89164 | 85724 | | 3433 | 3441 | 94.03 | sca_98_unmapped | Scaffold58 |
| 37275 | 39216 | 60489 | 58557 | | 1942 | 1933 | 91.22 | sca_98_unmapped | Scaffold58 |
