# Supplementary material for: The Genome Sequence of the Fungal Pathogen Fusarium virguliforme That Causes Sudden Death Syndrome in Soybean
Source: PLoS One. 2014 Jan 14;9(1):e81832. doi: 10.1371/journal.pone.0081832 (PMC3891557; doi:10.1371/journal.pone.0081832)
Supplement: Table S2 — Gene density and GC contents across Fusarium species. (DOC) [file pone.0081832.s011.doc]

**Table S2.** Gene density and GC contents across *Fusarium species.*

| **Organisms** | **Size (Mb)** | **No. of genes** | **Gene No. /10 kb** | **^1^G + C (%)** |
| --- | --- | --- | --- | --- |
| ***Nectria haematococca*** | 49.6 | 15,707 | 3.17 | 50.73 |
| ***Fusarium oxysporum*** | 59.5 | 17,608 | 2.96 | 47.28 |
| ***Fusarium verticillioides*** | 40.5 | 14,195 | 3.50 | 48.62 |
| ***Fusarium graminearum*** | 35.3 | 13,321 | 3.77 | 48.04 |
| ***Fusarium virguliforme*** | 50.9 | 14,845 | 2.92 | 49.04 |

^1^G + C contents of the coding sequences.
