# Supplementary material for: The Genome Sequence of the Fungal Pathogen Fusarium virguliforme That Causes Sudden Death Syndrome in Soybean
Source: PLoS One. 2014 Jan 14;9(1):e81832. doi: 10.1371/journal.pone.0081832 (PMC3891557; doi:10.1371/journal.pone.0081832)
Supplement: Table S3 — Variation in gene density and G + C contents of coding sequences observed among 23 major scaffolds of the F. virguliforme genome. (DOC) [file pone.0081832.s012.doc]

**Table S3.** Variation in gene density and G + C contents of coding sequences observed among 23 major scaffolds of the *F. virguliforme* genome.

| **Scaffolds** | **Size (Mb)** | **Gene Number/10 kb** | **G + C (%)** |
| --- | --- | --- | --- |
| Scaffold1 | 5.05 | 3.14 | 53.45 |
| Scaffold2 | 4.71 | 3.14 | 53.81 |
| Scaffold3 | 2.70 | 3.21 | 54 |
| Scaffold4 | 2.30 | 3.15 | 52.11 |
| Scaffold5 | 2.32 | 3.24 | 54.84 |
| Scaffold6 | 2.04 | 3.44 | 53 |
| Scaffold7 | 1.52 | 3.39 | 53.69 |
| Scaffold8 | 1.39 | 3.25 | 55.2 |
| Scaffold9 | 1.30 | 3.45 | 55.23 |
| Scaffold10 | 1.29 | 3.42 | 53.67 |
| Scaffold11 | 1.27 | 3.29 | 54.01 |
| Scaffold12 | 1.20 | 3.54 | 50.49 |
| Scaffold13 | 1.14 | 3.61 | 54.36 |
| Scaffold14 | 1.15 | 3.11 | 53.51 |
| Scaffold15 | 0.94 | 3.57 | 51.55 |
| Scaffold16 | 0.93 | 3.87 | 52.79 |
| Scaffold17 | 0.89 | 2.96 | 50.23 |
| Scaffold18 | 0.82 | 3.43 | 52.29 |
| Scaffold19 | 0.79 | 1.12 | 28.37 |
| Scaffold20 | 0.68 | 3.57 | 53.37 |
| Scaffold21 | 0.63 | 3.82 | 53.86 |
| Scaffold22 | 0.60 | 3.51 | 51.81 |
| Scaffold23 | 0.52 | 3.16 | 46.63 |
