# Supplementary material for: The Genome Sequence of the Fungal Pathogen Fusarium virguliforme That Causes Sudden Death Syndrome in Soybean
Source: PLoS One. 2014 Jan 14;9(1):e81832. doi: 10.1371/journal.pone.0081832 (PMC3891557; doi:10.1371/journal.pone.0081832)
Supplement: Table S4 — Pfam functional domain groups1. (DOC) [file pone.0081832.s013.doc]

**Table S4.** Pfam functional domain groups^1^.

| **Number**  **of proteins** | **Pfam function domain group** |
| --- | --- |
| 523 | MFS_1 Major Facilitator Superfamily |
| 396 | Zn_clus Fungal Zn(2)-Cys(6) binuclear cluster domain |
| 311 | Fungal_trans Fungal specific transcription factor domain |
| 266 | Sugar_tr Sugar (and other) transporter |
| 266 | adh_short short chain dehydrogenase |
| 219 | KR KR domain |
| 215 | Pyr_redox_2 Pyridine nucleotide-disulphide oxidoreductase |
| 214 | HET Heterokaryon incompatibility protein (HET) |
| 211 | DAO FAD dependent oxidoreductase |
| 200 | Pkinase Protein kinase domain |
| 183 | Epimerase NAD dependent epimerase/dehydratase family |
| 182 | Ank Ankyrin repeat |
| 174 | Pkinase_Tyr Protein tyrosine kinase |
| 165 | NACHT NACHT domain |
| 159 | Pyr_redox Pyridine nucleotide-disulphide oxidoreductase |
| 155 | AAA ATPase family associated with various cellular activities (AAA) |
| 154 | FAD_binding_3 FAD binding domain |
| 152 | APH Phosphotransferase enzyme family |
| 139 | Methyltransf_12 Methyltransferase domain |
| 137 | WD40 WD domain, G-beta repeat |
| 137 | FAD_binding_2 FAD binding domain |
| 134 | Miro Miro-like protein |
| 130 | Methyltransf_11 Methyltransferase domain |
| 129 | Thi4 Thi4 family |
| 129 | HI0933_like HI0933-like protein |
| 122 | p450 Cytochrome P450 |
| 122 | Abhydrolase_1 alpha/beta hydrolase fold |
| 118 | Lycopene_cycl Lycopene cyclase protein |
| 116 | RNA_helicase RNA helicase |
| 116 | IncA IncA protein |
| 115 | NmrA NmrA-like family |
| 110 | DUF258 Protein of unknown function, DUF258 |
| 108 | ADH_zinc_N Zinc-binding dehydrogenase |
| 104 | F-box F-box domain |
| 104 | AAA_5 AAA domain (dynein-related subfamily) |
| 97 | zf-C2H2 Zinc finger, C2H2 type |
| 97 | Abhydrolase_3 alpha/beta hydrolase fold |
| 94 | MTS Methyltransferase small domain |
| 93 | GIDA Glucose inhibited division protein A |
| 93 | 3Beta_HSD 3-beta hydroxysteroid dehydrogenase/isomerase family |
| 91 | TPR_2 Tetratricopeptide repeat |
| 87 | Helicase_C Helicase conserved C-terminal domain |
| 86 | Ubie_methyltran ubiE/COQ5 methyltransferase family |
| 86 | Peptidase_S9 Prolyl oligopeptidase family |
| 84 | DEAD DEAD/DEAH box helicase |
| 83 | ADH_N Alcohol dehydrogenase GroES-like domain |
| 82 | AA_permease Amino acid permease |
| 81 | TRI12 Fungal trichothecene efflux pump (TRI12) |
| 80 | TrkA_N TrkA-N domain |
| 80 | NB-ARC NB-ARC domain |
| 80 | 3HCDH_N 3-hydroxyacyl-CoA dehydrogenase, NAD binding domain |
| 78 | Shikimate_DH Shikimate / quinate 5-dehydrogenase |
| 78 | Acetyltransf_1 Acetyltransferase (GNAT) family |
| 74 | Zeta_toxin Zeta toxin |
| 74 | RRM_1 RNA recognition motif. (a.k.a. RRM, RBD, or RNP domain) |
| 73 | Polysacc_synt_2 Polysaccharide biosynthesis protein |
| 73 | Kdo Lipopolysaccharide kinase (Kdo/WaaP) family |
| 72 | SMC_N RecF/RecN/SMC N terminal domain |
| 72 | ResIII Type III restriction enzyme, res subunit |
| 70 | TPR_1 Tetratricopeptide repeat |
| 69 | bZIP_1 bZIP transcription factor |
| 69 | ABC_tran ABC transporter |
| 67 | MobB Molybdopterin guanine dinucleotide synthesis protein B |
| 67 | Dynamin_N Dynamin family |
| 66 | zf-C3HC4 Zinc finger, C3HC4 type (RING finger) |
| 65 | GTP_EFTU Elongation factor Tu GTP binding domain |
| 65 | FtsJ FtsJ-like methyltransferase |
| 64 | Arch_ATPase Archaeal ATPase |
| 63 | PrmA Ribosomal protein L11 methyltransferase (PrmA) |
| 63 | CDC45 CDC45-like protein |
| 63 | 2-Hacid_dh_C D-isomer specific 2-hydroxyacid dehydrogenase, NAD binding domain |
| 61 | Methyltransf_16 Putative methyltransferase |
| 61 | AlaDh_PNT_C Alanine dehydrogenase/PNT, C-terminal domain |
| 59 | Amino_oxidase Flavin containing amine oxidoreductase |
| 57 | Nop14 Nop14-like family |
| 57 | NAD_binding_4 Male sterility protein |
| 57 | Methyltransf_4 Putative methyltransferase |
| 56 | Saccharop_dh Saccharopine dehydrogenase |
| 56 | IstB IstB-like ATP binding protein |
| 56 | cobW CobW/HypB/UreG, nucleotide-binding domain |
| 55 | GSPII_E Type II/IV secretion system protein |
| 55 | COesterase Carboxylesterase |
| 54 | Hydrolase haloacid dehalogenase-like hydrolase |
| 53 | zf-C2H2_jaz Zinc-finger double-stranded RNA-binding |
| 53 | ApbA Ketopantoate reductase PanE/ApbA |
| 52 | Trp_halogenase Tryptophan halogenase |
| 51 | Semialdhyde_dh Semialdehyde dehydrogenase, NAD binding domain |
| 51 | RuvB_N Holliday junction DNA helicase ruvB N-terminus |
| 51 | Ras Ras family |
| 51 | PGAP1 PGAP1-like protein |
| 51 | NAD_binding_2 NAD binding domain of 6-phosphogluconate dehydrogenase |
| 51 | FAD_binding_4 FAD binding domain |
| 51 | AMP-binding AMP-binding enzyme |
| 50 | HEAT HEAT repeat |

^1^Pfam functional domain groups containing fifty and more *F. virguliforme* proteins are listed.
