# Supplementary material for: The Genome Sequence of the Fungal Pathogen Fusarium virguliforme That Causes Sudden Death Syndrome in Soybean
Source: PLoS One. 2014 Jan 14;9(1):e81832. doi: 10.1371/journal.pone.0081832 (PMC3891557; doi:10.1371/journal.pone.0081832)
Supplement: Table S5 — List of the normalized1 pfam domains of Fusarium species. (DOC) [file pone.0081832.s014.doc]

**Table S5. List of the normalized^1^ pfam domains across *Fusarium* species.**

| **Protein Domain (Pfam)** | ***F. virguliforme*** | ***N. haematococca*** | ***F. oxysporum*** | ***F. graminearum*** | ***F. verticillioides*** |
| --- | --- | --- | --- | --- | --- |
| MFS_1 Major Facilitator Superfamily | 29.156 | 29.635 | 30.496 | 26.691 | 32.083 |
| Zn_clus Fungal Zn(2)-Cys(6) binuclear cluster domain | 22.289 | 23.539 | 15.774 | 19.573 | 15.514 |
| Fungal_trans Fungal specific transcription factor domain | 17.448 | 19.089 | 14.872 | 15.516 | 14.459 |
| adh_short short chain dehydrogenase | 14.972 | 17.888 | 18.378 | 14.876 | 17.252 |
| Sugar_tr Sugar (and other) transporter | 14.972 | 15.663 | 16.675 | 14.235 | 17.066 |
| KR KR domain | 12.326 | 15.797 | 16.224 | 12.598 | 14.770 |
| Abhydrolase_5 Alpha/beta hydrolase family | 11.707 | 11.480 | 12.419 | 13.737 | 13.342 |
| HET Heterokaryon incompatibility protein (HET) | 12.045 | 12.949 | 4.707 | 6.833 | 5.337 |
| DAO FAD dependent oxidoreductase | 11.932 | 12.860 | 13.270 | 11.317 | 12.411 |
| Abhydrolase_6 Alpha/beta hydrolase family | 11.876 | 11.703 | 11.968 | 12.598 | 12.722 |
| Pyr_redox_2 Pyridine nucleotide-disulphide oxidoreductase | 11.764 | 12.593 | 13.671 | 11.388 | 12.722 |
| Epimerase NAD dependent epimerase/dehydratase family | 10.300 | 14.417 | 14.672 | 11.744 | 12.908 |
| Pkinase Protein kinase domain | 11.257 | 9.255 | 10.566 | 9.466 | 9.495 |
| Ank Ankyrin repeat | 10.244 | 6.363 | 7.712 | 7.758 | 7.199 |
| AAA ATPase family associated with various cellular activities (AAA) | 8.724 | 7.342 | 7.611 | 10.036 | 8.750 |
| Ank_2 Ankyrin repeats (3 copies) | 10.131 | 6.630 | 7.862 | 8.114 | 7.385 |
| Pkinase_Tyr Protein tyrosine kinase | 9.794 | 8.010 | 7.962 | 8.328 | 8.316 |
| Methyltransf_18 Methyltransferase domain | 9.400 | 8.454 | 8.162 | 9.039 | 9.060 |
| NACHT NACHT domain | 9.231 | 7.743 | 7.611 | 9.324 | 7.074 |
| Pyr_redox Pyridine nucleotide-disulphide oxidoreductase | 8.949 | 8.766 | 9.815 | 8.897 | 9.619 |
| FAD_binding_3 FAD binding domain | 8.668 | 8.766 | 9.414 | 7.829 | 8.998 |
| WD40 WD domain, G-beta repeat | 7.711 | 6.319 | 7.111 | 9.324 | 7.323 |
| APH Phosphotransferase enzyme family | 8.555 | 6.541 | 7.762 | 6.904 | 5.523 |
| Methyltransf_12 Methyltransferase domain | 7.824 | 7.031 | 7.111 | 7.687 | 7.323 |
| FAD_binding_2 FAD binding domain | 7.767 | 8.276 | 9.264 | 7.900 | 8.440 |
| F-box-like F-box-like | 7.711 | 5.473 | 3.305 | 5.979 | 3.972 |
| Miro Miro-like protein | 7.542 | 6.185 | 6.360 | 9.324 | 8.192 |
| Methyltransf_11 Methyltransferase domain | 7.317 | 6.942 | 7.161 | 7.616 | 7.137 |
| Thi4 Thi4 family | 7.261 | 7.698 | 7.662 | 6.975 | 8.005 |
| HI0933_like HI0933-like protein | 7.261 | 7.476 | 8.563 | 7.473 | 8.192 |
| p450 Cytochrome P450 | 6.867 | 7.209 | 8.413 | 8.256 | 8.005 |
| Abhydrolase_1 alpha/beta hydrolase fold | 6.698 | 6.408 | 7.011 | 7.687 | 7.137 |
| Lycopene_cycl Lycopene cyclase protein | 6.642 | 7.031 | 7.712 | 5.908 | 6.640 |
| RNA_helicase RNA helicase | 6.529 | 5.206 | 5.208 | 6.619 | 5.461 |
| IncA IncA protein | 6.473 | 4.984 | 8.563 | 7.473 | 7.199 |
| NmrA NmrA-like family | 6.360 | 8.944 | 9.214 | 5.979 | 7.385 |
| AAA_10 AAA-like domain | 6.248 | 5.340 | 5.258 | 6.762 | 5.771 |
| DUF258 Protein of unknown function, DUF258 | 6.191 | 5.696 | 5.759 | 7.402 | 6.702 |
| ADH_zinc_N Zinc-binding dehydrogenase | 6.079 | 7.120 | 7.712 | 5.694 | 7.261 |
| F-box F-box domain | 5.966 | 4.672 | 8.112 | 4.484 | 4.096 |
| AAA_5 AAA domain (dynein-related subfamily) | 5.854 | 4.672 | 5.308 | 6.263 | 5.647 |
| 3Beta_HSD 3-beta hydroxysteroid dehydrogenase/isomerase family | 5.235 | 6.675 | 7.061 | 6.263 | 6.392 |
| Abhydrolase_3 alpha/beta hydrolase fold | 5.516 | 6.497 | 6.410 | 5.979 | 6.392 |
| ADH_N Alcohol dehydrogenase GroES-like domain | 4.672 | 6.319 | 6.259 | 4.769 | 5.647 |
| GIDA Glucose inhibited division protein A | 5.235 | 5.785 | 6.109 | 5.053 | 5.895 |
| 3HCDH_N 3-hydroxyacyl-CoA dehydrogenase, NAD binding domain | 4.503 | 5.162 | 5.859 | 5.338 | 5.523 |
| Polysacc_synt_2 Polysaccharide biosynthesis protein | 4.109 | 4.361 | 5.809 | 4.698 | 5.213 |
| bZIP_2 Basic region leucine zipper | 2.420 | 1.557 | 5.709 | 2.776 | 2.420 |
| Peptidase_S9 Prolyl oligopeptidase family | 4.841 | 6.096 | 5.659 | 5.552 | 6.082 |
| TrkA_N TrkA-N domain | 4.672 | 5.429 | 5.508 | 5.125 | 5.461 |
| zf-C2H2 Zinc finger, C2H2 type | 5.460 | 5.028 | 5.158 | 6.263 | 4.965 |
| TPR_2 Tetratricopeptide repeat | 5.066 | 3.738 | 5.158 | 5.623 | 4.406 |
| Shikimate_DH Shikimate / quinate 5-dehydrogenase | 4.390 | 5.651 | 5.158 | 5.409 | 5.213 |
| MTS Methyltransferase small domain | 5.291 | 5.162 | 4.256 | 4.982 | 5.027 |
| 3HCDH_N 3-hydroxyacyl-CoA dehydrogenase, NAD binding domain | 4.503 | 5.073 | 5.859 | 5.338 | 5.523 |
| zf-C2H2 Zinc finger, C2H2 type | 5.460 | 5.028 | 5.158 | 6.263 | 4.965 |
| NAD_binding_4 Male sterility protein | 3.208 | 4.761 | 4.707 | 3.843 | 4.840 |
| Ubie_methyltran ubiE/COQ5 methyltransferase family | 4.841 | 4.539 | 4.457 | 4.484 | 4.716 |

^1^The pfam hits were normalized using total genome standard deviation (SD) divided by number of hits. The SDs of *F. virguliforme*, *N. haematococca*, *F. oxysporum*, *F. graminearum*, *F. verticillioides* were 17.77, 22.47, 19.97, 14.05 and 16.11, respectively
