# Supplementary material for: The Genome Sequence of the Fungal Pathogen Fusarium virguliforme That Causes Sudden Death Syndrome in Soybean
Source: PLoS One. 2014 Jan 14;9(1):e81832. doi: 10.1371/journal.pone.0081832 (PMC3891557; doi:10.1371/journal.pone.0081832)
Supplement: Table S6 — Organisms and data sources used in genome analyses. (DOC) [file pone.0081832.s015.doc]

**Table S6.** Organisms and data sources used in genome analyses.

| **Organism** | **Data source** |
| --- | --- |
| *A. nidulans* | [http://www.broadinstitute.org](http://www.broadinstitute.org/) |
| *A. thaliana* | [ftp://ftp.arabidopsis.org](ftp://ftp.arabidopsis.org/) |
| *A. tumefaciens str. C58* | [http://agro.vbi.vt.edu](http://agro.vbi.vt.edu/) |
| *C. elegans* | [http://www.sanger.ac.uk](http://www.sanger.ac.uk/) |
| *D. discoideum* | [http://dictybase.org](http://dictybase.org/) |
| *D. rerio* | [ftp://ftp.ncbi.nih.gov](ftp://ftp.ncbi.nih.gov/) |
| *D. melanogaster* | [ftp://ftp.flybase.net](ftp://ftp.flybase.net/) |
| *E. coli_12* | [ftp://ftp.jcvi.org](ftp://ftp.jcvi.org/) |
| *F. graminearum* | [http://www.broadinstitute.org](http://www.broadinstitute.org/) |
| *F. oxysporum* | [http://www.broadinstitute.org](http://www.broadinstitute.org/) |
| *F. verticillioides* | [http://www.broadinstitute.org](http://www.broadinstitute.org/) |
| *G. max* | [ftp://ftp.jgi-psf.org](ftp://ftp.jgi-psf.org/) |
| *H. sapiens* | [ftp://ftp.ncbi.nih.gov](ftp://ftp.ncbi.nih.gov/) |
| *N. crassa* | [http://www.broadinstitute.org](http://www.broadinstitute.org/) |
| *N. haematococca* | [http://genome.jgi-psf.org](http://genome.jgi-psf.org/) |
| *O. sativa ssp. japonica* | [http://rgp.dna.affrc.go.jp](http://rgp.dna.affrc.go.jp/) |
| *P. blakesleeanus* | [http://genome.jgi-psf.org](http://genome.jgi-psf.org/) |
| *P. infestans* | [http://www.broadinstitute.org](http://www.broadinstitute.org/) |
| *P. sojae* | [http://www.broadinstitute.org](http://www.broadinstitute.org/) |
| *P. syringae* | [ftp://ftp.jcvi.org](ftp://ftp.jcvi.org/) |
| *R. leguminosarum* | [ftp://ftp.sanger.ac.uk](ftp://ftp.sanger.ac.uk/) |
| *R. oryzae* | [http://www.broadinstitute.org](http://www.broadinstitute.org/) |
| *S. cerevisiae* | [http://broad.harvard.edu](http://broad.harvard.edu/) |
| *U. maydis* | [http://www.broadinstitute.org](http://www.broadinstitute.org/) |
| *Z. mays* | [http://www.plantgdb.org](http://www.plantgdb.org/) |
