# Supplementary material for: The Genome Sequence of the Fungal Pathogen Fusarium virguliforme That Causes Sudden Death Syndrome in Soybean
Source: PLoS One. 2014 Jan 14;9(1):e81832. doi: 10.1371/journal.pone.0081832 (PMC3891557; doi:10.1371/journal.pone.0081832)
Supplement: Table S7 — F. virguliforme ( Fv ) genes thaxft showed similarity to genes of other organisms. (DOC) [file pone.0081832.s016.doc]

**Table S7.** *F. virguliforme* (*Fv*) genes that showed similarity to genes of other organisms.

| **SL. No.** | **Organisms** | **(%) similar *Fv* genes** | **Proportion of genes**  **similar to *Fv* genes** |
| --- | --- | --- | --- |
| 1 | *N. haematococca* | ^1^88.03 | ^2^0.83 |
| 2 | *F. oxysporum* | 82.41 | 0.69 |
| 3 | *F. graminearum* | 80.79 | 0.90 |
| 4 | *F. verticillioides* | 80.57 | 0.84 |
| 5 | *N. crassa* | 66.68 | 1.00 |
| 6 | *A. nidulans* | 66.11 | 0.93 |
| 7 | *U. maydis* | 42.21 | 0.96 |
| 8 | *P. blakesleeanus* | 40.71 | 0.41 |
| 9 | *R. oryzae* | 39.51 | 0.34 |
| 10 | *S cerevisiae* | 33.92 | 0.94 |
| 11 | *D. rerio* | 31.24 | 0.17 |
| 12 | *G. max* | 31.09 | 0.10 |
| 13 | *A. thaliana* | 31.01 | 0.14 |
| 14 | *H. sapiens* | 30.73 | 0.12 |
| 15 | *P. sajae* | 30.25 | 0.23 |
| 16 | *P. infestans* | 29.99 | 0.25 |
| 17 | *D. discoideum* | 29.39 | 0.33 |
| 18 | *Z. mays* | 29.29 | 0.08 |
| 19 | *O. sativa ssp. japonica* | 28.77 | 0.07 |
| 20 | *D. melanogaster* | 27.50 | 0.19 |
| 21 | *C. elegans* | 26.79 | 0.10 |
| 22 | *R. leguminosarum* | 21.85 | 0.45 |
| 23 | *P.syringae* | 15.8 | 0.42 |
| 24 | *A. tumefaciens* | 14.81 | 0.41 |
| 25 | *E. coli* | 13.98 | 0.42 |

^1^Percentage *F. virguliforme* genes that are similar (E ≤ 10^-9^) to the genes of the selected organisms.

^2^Proportion of genes in an organism that showed similarity (E ≤ 10^-9^) to *F. virguliforme* genes.
