# Supplementary material for: The Genome Sequence of the Fungal Pathogen Fusarium virguliforme That Causes Sudden Death Syndrome in Soybean
Source: PLoS One. 2014 Jan 14;9(1):e81832. doi: 10.1371/journal.pone.0081832 (PMC3891557; doi:10.1371/journal.pone.0081832)
Supplement: Table S8 — Conserved F. virguliforme genes among selected organisms. (DOC) [file pone.0081832.s017.doc]

**Table S8.** Conserved *F. virguliforme* genes among selected organisms.

| **SL. No** | **Organism** | **Number of common genes** |
| --- | --- | --- |
| 1 | *N. haematococca* | 13,068 |
| 2 | *F. oxysporum* | 11,878 |
| 3 | *F. graminearum* | 11,296 |
| 4 | *F. verticillioides* | 11,043 |
| 5 | *N. crassa* | 9,335 |
| 6 | *A. nidulans* | 8,507 |
| 7 | *U. maydis* | 5,843 |
| 8 | *P. blakesleeanus* | 5,057 |
| 9 | *R. oryzae* | 4,772 |
| 10 | *S cerevisiae* | 3,960 |
| 11 | *D. rerio* | 3,039 |
| 12 | *G. max* | 2,765 |
| 13 | *A. thaliana* | 2,737 |
| 14 | *H. sapiens* | 2,694 |
| 15 | *P. sajae* | 2,506 |
| 16 | *P. infestans* | 2,469 |
| 17 | *D. discoideum* | 2,362 |
| 18 | *Z. mays* | 2,319 |
| 19 | *O. sativa ssp. japonica* | 2,280 |
| 20 | *D. melanogaster* | 2,233 |
| 21 | *C. elegans* | 2,188 |
| 22 | *R. leguminosarum* | 935 |
| 23 | *P.syringae* | 830 |
| 24 | *A. tumefaciens* | 796 |
| 25 | *E. coli* | 762 |
