# Supplementary material for: The Genome Sequence of the Fungal Pathogen Fusarium virguliforme That Causes Sudden Death Syndrome in Soybean
Source: PLoS One. 2014 Jan 14;9(1):e81832. doi: 10.1371/journal.pone.0081832 (PMC3891557; doi:10.1371/journal.pone.0081832)
Supplement: Table S9 — The 762 F. virguliforme genes that are conserved among 25 diverse organisms. (DOC) [file pone.0081832.s018.doc]

**Table S9.** The 762 *F. virguliforme* genes that are conserved among 25 diverse organisms.

| **Fv genes** | **GO Accession** | **Function** |
| --- | --- | --- |
| Fv12 | No Annotation |  |
| Fv34 | GO:0055114 | succinate-semialdehyde dehydrogenase |
| Fv74 | GO:0030170 | acetylornithine aminotransferase |
| Fv121 | GO:0022891 | mfs quinate |
| Fv124 | GO:0009986 | enolase |
| Fv134 | GO:0009113 | amidophosphoribosyltransferase |
| Fv136 | GO:0005737 | aspartyl-trna synthetase |
| Fv150 | GO:0055114 | xylitol dehydrogenase |
| Fv153 | GO:0009851 | sodium p-type atpase |
| Fv181 | GO:0003842 | delta-1-pyrroline-5-carboxylate dehydrogenase |
| Fv196 | GO:0009027 | tartrate |
| Fv198 | GO:0022891 | mfs monosaccharide |
| Fv235 | No Annotation |  |
| Fv252 | GO:0030529 | u5 small nuclear ribonucleoprotein component |
| Fv253 | GO:0003985 | acetyl- acetyltransferase |
| Fv281 | GO:0055114 | aldehyde dehydrogenase |
| Fv292 | GO:0006457 | domain protein |
| Fv321 | GO:0008152 | amp-binding enzyme |
| Fv330 | GO:0030170 | threonine biosynthetic |
| Fv334 | GO:0055114 | peptide methionine sulfoxide reductase |
| Fv341 | GO:0016616 | d-isomer specific 2-hydroxyacid dehydrogenase family protein |
| Fv351 | GO:0005840 | 60s ribosomal protein l23 |
| Fv416 | GO:0005737 | threonyl-trna synthetase |
| Fv422 | GO:0009851 | tyrosyl-trna synthetase |
| Fv435 | GO:0005737 | adenine phosphoribosyltransferase 1 |
| Fv440 | GO:0055114 | salicylaldehyde dehydrogenase |
| Fv453 | GO:0016884 | d-lactate dehydrogenase |
| Fv455 | GO:0008152 | enoyl- hydratase isomerase family protein |
| Fv481 | GO:0004326 | folylpolyglutamate synthase |
| Fv482 | GO:0004326 | folylpolyglutamate synthase |
| Fv487 | GO:0005737 | isoleucyl-trna synthetase |
| Fv502 | GO:0009851 | abc bile acid |
| Fv516 | GO:0005975 | l-arabinitol 4-dehydrogenase |
| Fv521 | GO:0055114 | succinate-semialdehyde dehydrogenase |
| Fv531 | GO:0003938 | inosine-5 -monophosphate dehydrogenase imd2 |
| Fv532 | GO:0043581 | mitochondrial processing peptidase beta subunit |
| Fv576 | GO:0005737 | lysyl-trna synthetase |
| Fv580 | GO:0030170 | cysteine synthase 2 |
| Fv582 | GO:0005737 | alanyl-trna synthetase |
| Fv592 | GO:0003899 | dna-dependent rna polymerase ii largest |
| Fv597 | GO:0009851 | hsp70 chaperone |
| Fv649 | GO:0003755 | peptidyl-prolyl cis-trans isomerase 10 |
| Fv655 | GO:0009851 | dead deah box |
| Fv662 | GO:0055114 | aldehyde dehydrogenase |
| Fv674 | GO:0005737 | family helicase |
| Fv686 | GO:0046933 | vacuolar atp synthase subunit b |
| Fv716 | GO:0006350 | dna-directed rna polymerase iii 130 kda polypeptide |
| Fv723 | GO:0005524 | heat shock 70 kda protein |
| Fv733 | GO:0022891 | polyol transporter 5 |
| Fv745 | GO:0006807 | nitrilase |
| Fv758 | GO:0000774 | mitochondrial co-chaperone |
| Fv771 | GO:0016212 | kynurenine |
| Fv787 | GO:0008152 | acetyl-coenzyme a synthetase |
| Fv805 | GO:0030170 | cystathionine beta-lyase |
| Fv862 | GO:0006006 | phosphoglucomutase |
| Fv870 | GO:0055114 | short-chain dehydrogenase reductase sdr |
| Fv887 | GO:0055114 | sporulation protein sps19 |
| Fv908 | GO:0005737 | s-adenosylmethionine synthetase |
| Fv937 | GO:0009851 | pre-mrna splicing factor atp-dependent rna helicase prp16 |
| Fv943 | GO:0030170 | cystathionine beta-lyase |
| Fv979 | GO:0016887 | abc transporter |
| Fv985 | GO:0005840 | 60s ribosomal protein l11 |
| Fv986 | GO:0045261 | mitochondrial f1 atpase subunit |
| Fv998 | GO:0016021 | sulfate permease |
| Fv1032 | GO:0003887 | dna damage repair protein |
| Fv1044 | GO:0009851 | pre-mrna-processing atp-dependent rna helicase prp5 |
| Fv1050 | GO:0008415 | dihydrolipoamide branched chain transacylase e2 |
| Fv1103 | GO:0004643 | phosphoribosylaminoimidazolecarboxamide formyltransferase imp cyclohydrolase |
| Fv1124 | GO:0005975 | ribulose-phosphate 3-epimerase |
| Fv1155 | GO:0016491 | short-chain dehydrogenase reductase family |
| Fv1178 | GO:0005737 | 26s protease regulatory subunit 7 |
| Fv1186 | GO:0009851 | atp-dependent dna helicase q1 |
| Fv1197 | GO:0009851 | abc multidrug transporter |
| Fv1234 | GO:0005388 | calcium-transporting atpase type 2c member 1 |
| Fv1252 | GO:0006573 | methylmalonate-semialdehyde dehydrogenase |
| Fv1265 | GO:0006457 | peptidyl-prolyl cis-trans |
| Fv1268 | GO:0050661 | 6-phosphogluconate dehydrogenase |
| Fv1277 | GO:0005737 | phenylalanyl-trna synthetase alpha chain |
| Fv1311 | GO:0003842 | delta-1-pyrroline-5-carboxylate dehydrogenase |
| Fv1318 | GO:0005737 | glycine cleavage system t protein |
| Fv1350 | GO:0030170 | cystathionine gamma-lyase |
| Fv1361 | GO:0004540 | exosome complex exonuclease exoribonuclease |
| Fv1372 | GO:0005388 | p-type calcium |
| Fv1375 | GO:0005737 | 26s protease regulatory subunit s10b |
| Fv1376 | GO:0004360 | glucosamine-fructose-6-phosphate aminotransferase |
| Fv1386 | GO:0009851 | dna replication factor c subunit |
| Fv1439 | GO:0004829 | threonyl-trna synthetase |
| Fv1454 | GO:0005388 | calcium p-type atpase |
| Fv1469 | GO:0006006 | glucose-6-phosphate 1-dehydrogenase |
| Fv1484 | GO:0003735 | 50s ribosomal protein l2 |
| Fv1517 | GO:0045454 | monothiol glutaredoxin-4 |
| Fv1549 | GO:0009986 | enolase |
| Fv1561 | GO:0009851 | atp-dependent rna helicase |
| Fv1575 | GO:0005524 | dna mismatch repair protein |
| Fv1610 | GO:0008152 | phenylacetyl- |
| Fv1678 | GO:0006629 | bifunctional p-450:nadph-p450 reductase |
| Fv1687 | GO:0016491 | retinol dehydrogenase 8 |
| Fv1709 | GO:0016874 | succinyl- beta subunit |
| Fv1710 | GO:0008152 | succinyl- synthetase subunit |
| Fv1728 | GO:0016620 | trichothecene 3-o-acetyltransferase |
| Fv1754 | GO:0047956 | glycerol dehydrogenase |
| Fv1807 | GO:0016491 | alcohol dehydrogenase |
| Fv1816 | GO:0045252 | dihydrolipoamide succinyltransferase |
| Fv1824 | GO:0030170 | serine palmitoyltransferase 1 |
| Fv1832 | GO:0003994 | mitochondrial aconitate |
| Fv1859 | GO:0003676 | domain-containing protein |
| Fv1861 | GO:0008652 | cystathionine gamma-synthase |
| Fv1866 | GO:0017111 | aaa family atpase |
| Fv1870 | GO:0043581 | and tpr domain protein |
| Fv1874 | GO:0005737 | dihydrolipoamide dehydrogenase |
| Fv1930 | GO:0006413 | mitochondrial translation initiation |
| Fv1949 | GO:0032301 | dna mismatch repair protein |
| Fv1989 | GO:0005737 | serine hydroxymethyltransferase |
| Fv2012 | GO:0008270 | l-threonine 3-dehydrogenase |
| Fv2029 | GO:0005737 | glyceraldehyde-3-phosphate dehydrogenase |
| Fv2042 | GO:0005737 | heat shock protein mitochondrial precursor |
| Fv2045 | GO:0003723 | trna (cytosine-5-)-methyltransferase ncl1 |
| Fv2048 | GO:0055114 | nadph-dependent alpha-keto amide |
| Fv2088 | GO:0005971 | ribonucleoside-diphosphate reductase large chain |
| Fv2099 | GO:0005737 | glutathione-disulfide reductase |
| Fv2137 | GO:0009851 | abc fatty acid |
| Fv2153 | GO:0005488 | 3-ketoacyl-(acyl-carrier-protein) reductase |
| Fv2154 | GO:0005524 | ubiquitin-like activating enzyme |
| Fv2218 | GO:0008152 | methylglutaconyl- hydratase |
| Fv2232 | GO:0005975 | d-xylose reductase |
| Fv2234 | GO:0008270 | alcohol dehydrogenase |
| Fv2235 | GO:0022891 | maltose high-affinity maltose transporter (alpha-glucoside transporter) |
| Fv2238 | GO:0016491 | #NAME? |
| Fv2269 | GO:0008152 | 2-keto-4-pentenoate hydratase |
| Fv2298 | GO:0005737 | pyruvate carboxylase |
| Fv2337 | GO:0009851 | peroxisome biosynthesis protein (pas1 peroxin-1) |
| Fv2345 | GO:0005524 | dna mismatch repair protein msh3 |
| Fv2359 | GO:0005737 | uridylate kinase |
| Fv2371 | GO:0009851 | abc drug exporter |
| Fv2376 | GO:0035091 | succinate dehydrogenase |
| Fv2389 | GO:0005737 | protein mitochondrial targeting protein |
| Fv2392 | GO:0043581 | dna mismatch repair protein |
| Fv2405 | GO:0009851 | eukaryotic translation initiation factor |
| Fv2407 | GO:0003924 | eukaryotic translation initiation factor 2 gamma subunit |
| Fv2413 | GO:0005737 | pre-mrna-splicing atp-dependent rna helicase prp28 |
| Fv2414 | GO:0005737 | valyl-trna synthetase |
| Fv2427 | GO:0050660 | trna dihydrouridine synthase |
| Fv2429 | GO:0004222 | peptidase m3 family protein |
| Fv2442 | GO:0045261 | atp synthase gamma chain |
| Fv2449 | GO:0009851 | lipid a export atp-binding permease protein msba |
| Fv2460 | GO:0004222 | glycoprotease family protein |
| Fv2509 | GO:0006166 | adenosine kinase |
| Fv2547 | GO:0009851 | atp-dependent rna helicase mrh4 |
| Fv2581 | GO:0009851 | abc multidrug transporter mdr1 |
| Fv2604 | GO:0016620 | succinate-semialdehyde dehydrogenase |
| Fv2606 | GO:0030170 | 4-aminobutyrate transaminase |
| Fv2629 | GO:0004637 | bifunctional purine biosynthetic protein |
| Fv2651 | GO:0003723 | atp-dependent rna helicase dbp8 |
| Fv2661 | GO:0008152 | enoyl- hydratase isomerase family protein |
| Fv2662 | GO:0030170 | cysteine synthase |
| Fv2666 | GO:0008270 | actin interacting protein 2 |
| Fv2673 | GO:0008152 | amp-binding domain |
| Fv2674 | GO:0016491 | aldo-keto |
| Fv2687 | GO:0003870 | 5-aminolevulinate mitochondrial precursor |
| Fv2697 | GO:0005488 | pyrroline-5-carboxylate reductase |
| Fv2711 | GO:0005622 | gtp binding |
| Fv2726 | GO:0004315 | 3-oxoacyl- |
| Fv2731 | GO:0009851 | aaa family |
| Fv2737 | GO:0050660 | dihydrouridine synthase family |
| Fv2765 | GO:0006268 | dna topoisomerase iii |
| Fv2781 | GO:0006094 | triosephosphate isomerase |
| Fv2806 | GO:0006457 | dnaj domain containing protein |
| Fv2813 | GO:0005737 | 3-isopropylmalate dehydrogenase |
| Fv2867 | GO:0009851 | atp-dependent rna helicase drs1 |
| Fv2937 | GO:0006633 | acetyl- carboxylase |
| Fv2946 | GO:0009851 | abc bile acid |
| Fv2969 | GO:0005737 | cystathionine beta-synthase |
| Fv2995 | GO:0016491 | short chain dehydrogenase |
| Fv3008 | GO:0031177 | nonribosomal peptide synthetase 10 |
| Fv3095 | GO:0016874 | amp-binding enzyme |
| Fv3103 | GO:0017111 | vacuolar sorting atpase |
| Fv3141 | GO:0004591 | alpha-ketoglutarate dehydrogenase complex subunit |
| Fv3142 | GO:0045252 | dihydrolipoamide succinyltransferase |
| Fv3156 | GO:0046872 | hexaprenyl pyrophosphate synthetase |
| Fv3158 | GO:0009851 | abc efflux transporter |
| Fv3187 | GO:0009374 | urea amidolyase |
| Fv3191 | GO:0055085 | iron-sulfur clusters transporter atm1 |
| Fv3199 | GO:0017111 | vesicular-fusion protein sec18 |
| Fv3206 | GO:0003723 | atp-dependent rrna helicase spb4 |
| Fv3215 | GO:0030170 | cysteine synthase |
| Fv3224 | GO:0005198 | iron-sulfur cluster assembly accessory protein |
| Fv3226 | GO:0004776 | succinyl- ligase beta-chain |
| Fv3230 | GO:0017111 | ribosome biogenesis atpase rix7 |
| Fv3256 | GO:0005737 | alcohol dehydrogenase i |
| Fv3273 | GO:0016301 | ribose-phosphate pyrophosphokinase |
| Fv3294 | GO:0005524 | hsp70 family chaperone lhs1 |
| Fv3321 | GO:0005737 | asparaginyl-trna synthetase |
| Fv3327 | GO:0003723 | atp dependent rna helicase |
| Fv3328 | GO:0020037 | acyl- dehydrogenase |
| Fv3371 | GO:0004326 | folylpolyglutamate synthase |
| Fv3381 | GO:0005737 | 26s protease regulatory subunit 6b |
| Fv3398 | GO:0009851 | pre-mrna splicing factor atp-dependent rna helicase prp43 |
| Fv3411 | GO:0022891 | mfs alpha-glucoside |
| Fv3423 | GO:0005758 | adenylate kinase cytosolic |
| Fv3428 | GO:0003676 | para-hydroxybenzoate-polyprenyltransferase |
| Fv3440 | GO:0005737 | isoleucyl-trna synthetase |
| Fv3453 | GO:0003899 | dna-directed rna polymerase iii largest subunit |
| Fv3467 | GO:0005840 | translational activator |
| Fv3468 | GO:0009851 | atp dependent rna |
| Fv3475 | GO:0055114 | alcohol dehydrogenase |
| Fv3493 | GO:0016491 | l-xylulose reductase |
| Fv3527 | GO:0043581 | c-1-tetrahydrofolate synthase |
| Fv3533 | GO:0008152 | transketolase |
| Fv3550 | GO:0016810 | nitrilase family protein |
| Fv3565 | GO:0003994 | aconitate hydratase |
| Fv3567 | GO:0055114 | glutathione-dependent formaldehyde dehydrogenase |
| Fv3606 | GO:0046872 | delta-aminolevulinic acid dehydratase |
| Fv3627 | GO:0006081 | aldehyde dehydrogenase 3b1 |
| Fv3651 | GO:0016301 | ubiquinone biosynthesis |
| Fv3720 | GO:0030170 | trna splicing protein |
| Fv3819 | GO:0016874 | acetate- ligase |
| Fv3822 | GO:0055114 | quinone oxidoreductase |
| Fv3827 | GO:0008152 | pyridoxal-phosphate dependent |
| Fv3829 | GO:0006810 | transporter protein smf2 |
| Fv3859 | GO:0055085 | atp-dependent rna helicase dbp5 |
| Fv3882 | GO:0005737 | dimethyladenosine transferase dimethyltransferase |
| Fv3896 | GO:0008152 | 4-coumarate- ligase |
| Fv3923 | GO:0020037 | acyl- dehydrogenase |
| Fv3938 | No Annotation |  |
| Fv3952 | GO:0005737 | cysteinyl-trna synthetase |
| Fv3964 | GO:0004550 | nucleoside diphosphate kinase |
| Fv4001 | GO:0009851 | atp-binding cassette multidrug transport protein atrc |
| Fv4016 | GO:0005622 | gtp binding protein |
| Fv4024 | GO:0006457 | dnaj heat shock family protein |
| Fv4048 | GO:0009851 | abc multidrug |
| Fv4060 | GO:0055114 | formate dehydrogenase |
| Fv4086 | GO:0055114 | l-xylulose reductase |
| Fv4089 | GO:0009851 | deah-box rna helicase |
| Fv4090 | GO:0055114 | short-chain dehydrogenase reductase family |
| Fv4099 | GO:0006629 | acetoacetyl- synthase |
| Fv4111 | GO:0009851 | vacuolar atp synthase catalytic subunit a |
| Fv4117 | GO:0004540 | cell wall biogenesis protein phosphatase |
| Fv4128 | GO:0008152 | mitochondrial 3-hydroxyisobutyryl- |
| Fv4167 | GO:0005737 | atp-dependent rna helicase ded1 |
| Fv4179 | GO:0009851 | matrix aaa protease map-1 |
| Fv4219 | GO:0009851 | abc transporter family protein |
| Fv4228 | GO:0016787 | fumarylacetoacetate hydrolase family protein |
| Fv4238 | GO:0009851 | replication factor c subunit 4 |
| Fv4252 | GO:0004470 | nadp-dependent malic enzyme |
| Fv4259 | GO:0003746 | elongation factor tu |
| Fv4261 | GO:0005788 | disulfide isomerase |
| Fv4284 | GO:0030170 | serine family amino acid catabolism-related protein |
| Fv4303 | No Annotation |  |
| Fv4314 | GO:0009851 | dead deah box |
| Fv4329 | No Annotation |  |
| Fv4333 | GO:0009851 | leucyl-trna synthetase |
| Fv4334 | GO:0006555 | methylenetetrahydrofolate reductase |
| Fv4337 | No Annotation |  |
| Fv4356 | GO:0005737 | adenylosuccinate synthetase |
| Fv4375 | GO:0043190 | molecular chaperone |
| Fv4385 | GO:0005737 | o-sialoglycoprotein endopeptidase |
| Fv4433 | No Annotation |  |
| Fv4454 | GO:0004617 | d-3-phosphoglycerate dehydrogenase |
| Fv4459 | GO:0009116 | uracil phosphoribosyltransferase |
| Fv4504 | GO:0009851 | abc a-pheromone efflux pump |
| Fv4506 | No Annotation |  |
| Fv4513 | GO:0004743 | pyruvate kinase |
| Fv4538 | GO:0005524 | dna mismatch repair protein msh6 |
| Fv4540 | GO:0048034 | protoheme ix farnesyltransferase |
| Fv4543 | No Annotation |  |
| Fv4545 | GO:0022891 | sugar transport protein |
| Fv4554 | GO:0045454 | mitochondrial peroxiredoxin prx1 |
| Fv4570 | GO:0008270 | d-lactate dehydrogenase |
| Fv4586 | GO:0047432 | #NAME? |
| Fv4610 | No Annotation |  |
| Fv4613 | GO:0008270 | zinc-containing alcohol |
| Fv4623 | GO:0022891 | mfs monosaccharide |
| Fv4657 | GO:0005737 | alcohol dehydrogenase i |
| Fv4698 | GO:0009851 | multidrug resistance protein 3 (p glycoprotein 3) |
| Fv4699 | GO:0009851 | multidrug resistance protein 3 (p glycoprotein 3) |
| Fv4703 | GO:0009851 | abc multidrug |
| Fv4748 | GO:0005737 | translation elongation factor 1 alpha |
| Fv4770 | GO:0005524 | hsp70 chaperone |
| Fv4796 | GO:0003723 | atp-dependent rna helicase sub2 |
| Fv4810 | GO:0009374 | methylcrotonoyl- carboxylase subunit alpha |
| Fv4824 | GO:0000785 | mrna-nucleus export atpase |
| Fv4845 | GO:0016874 | amp-binding enzyme |
| Fv4850 | GO:0004370 | glycerol |
| Fv4887 | GO:0016491 | d-lactate mitochondrial precursor |
| Fv4955 | GO:0016491 | oxidoreductase |
| Fv4957 | GO:0009851 | dead deah box rna helicase |
| Fv4967 | GO:0045239 | fumarate mitochondrial precursor |
| Fv4981 | GO:0055114 | d-arabinitol dehydrogenase |
| Fv5022 | GO:0005488 | polyketide synthase |
| Fv5032 | GO:0005785 | signal sequence receptor alpha subunit |
| Fv5042 | GO:0003755 | peptidyl prolyl cis-trans isomerase |
| Fv5062 | GO:0005737 | lysyl-trna synthetase |
| Fv5068 | GO:0031072 | domain-containing protein |
| Fv5073 | GO:0005737 | fad-dependent pyridine nucleotide-disulphide oxidoreductase |
| Fv5102 | GO:0003824 | 2-oxoisovalerate dehydrogenase subunit beta |
| Fv5106 | GO:0022891 | mfs sugar |
| Fv5127 | GO:0022891 | myo-inositol transporter |
| Fv5141 | GO:0055114 | electron transfer flavoprotein-ubiquinone oxidoreductase |
| Fv5159 | GO:0030170 | alanine aminotransferase |
| Fv5192 | GO:0006415 | eukaryotic peptide chain release factor gtp-binding subunit |
| Fv5225 | GO:0009851 | copper resistance-associated p-type atpase |
| Fv5230 | GO:0055114 | sulfite reductase flavoprotein component |
| Fv5244 | GO:0005524 | nucleotide binding |
| Fv5254 | GO:0016887 | abc transporter |
| Fv5265 | GO:0009851 | h k atpase alpha |
| Fv5280 | GO:0030170 | serine hydroxymethyltransferase |
| Fv5282 | GO:0004375 | glycine dehydrogenase |
| Fv5283 | GO:0030170 | serine family amino acid catabolism-related protein |
| Fv5308 | GO:0008237 | aminopeptidase |
| Fv5332 | GO:0009851 | sodium p-type |
| Fv5334 | GO:0008152 | amp-dependent synthetase and ligase |
| Fv5341 | GO:0009851 | abc transporter cdr4 |
| Fv5347 | No Annotation |  |
| Fv5353 | No Annotation |  |
| Fv5368 | GO:0009851 | abc multidrug |
| Fv5370 | GO:0009851 | brefeldin a resistance protein |
| Fv5403 | GO:0008152 | 3-ketoacyl- thiolase |
| Fv5418 | GO:0055114 | short-chain dehydrogenase reductase sdr |
| Fv5420 | GO:0022891 | myo-inositol transporter |
| Fv5428 | GO:0020037 | cytochrome b5 |
| Fv5430 | GO:0009851 | abc multidrug |
| Fv5448 | GO:0016787 | fumarylacetoacetate hydrolase |
| Fv5518 | GO:0022891 | quinate permease |
| Fv5531 | GO:0046872 | superoxide dismutase |
| Fv5574 | GO:0004776 | succinyl- ligase alpha- mitochondrial precursor |
| Fv5578 | GO:0005737 | 26s protease regulatory subunit 4 |
| Fv5604 | GO:0016887 | atp-binding cassette sub-family f member 2 |
| Fv5653 | GO:0016620 | aldehyde dehydrogenase |
| Fv5663 | GO:0003842 | delta-1-pyrroline-5-carboxylate dehydrogenase |
| Fv5683 | GO:0022891 | mfs quinate |
| Fv5693 | GO:0055114 | rhamnolipids biosynthesis 3-oxoacyl- |
| Fv5728 | GO:0009851 | abc multidrug transporter mdr2 |
| Fv5751 | GO:0055114 | short chain dehydrogenase reductase family |
| Fv5770 | GO:0050660 | electron transfer flavoprotein subunit alpha |
| Fv5773 | GO:0005524 | dna mismatch repair protein |
| Fv5778 | GO:0030170 | cystathionine beta-lyase |
| Fv5779 | GO:0009851 | atpase family aaa domain-containing protein 1 |
| Fv5799 | GO:0005737 | heat shock protein hsp88 |
| Fv5800 | GO:0003924 | mitochondrial translation initiation factor if- |
| Fv5816 | GO:0004437 | inositol monophosphatase |
| Fv5823 | GO:0006629 | bifunctional p-450:nadph-p450 reductase |
| Fv5831 | GO:0009851 | aspartyl-trna synthetase |
| Fv5886 | No Annotation |  |
| Fv5889 | GO:0006457 | domain-containing protein |
| Fv5890 | GO:0009851 | mrna splicing factor rna helicase |
| Fv5986 | GO:0005737 | adenylate kinase 2 |
| Fv6015 | GO:0030170 | cystathionine beta-lyase |
| Fv6017 | GO:0055114 | succinate-semialdehyde dehydrogenase |
| Fv6060 | No Annotation |  |
| Fv6086 | GO:0004222 | metallopeptidase |
| Fv6088 | GO:0008270 | alcohol dehydrogenase |
| Fv6096 | GO:0055114 | short-chain dehydrogenase |
| Fv6098 | GO:0008152 | dihydroxyacetone synthase |
| Fv6105 | GO:0009851 | abc transporter |
| Fv6127 | GO:0005488 | aldehyde dehydrogenase |
| Fv6130 | GO:0022891 | sugar transport protein 4 |
| Fv6148 | GO:0045454 | thioredoxin-like protein |
| Fv6154 | GO:0009851 | abc transporter cdr4 |
| Fv6168 | GO:0005388 | p-type calcium |
| Fv6184 | GO:0016491 | aldehyde dehydrogenase |
| Fv6192 | GO:0008270 | s- glutathione dehydrogenase |
| Fv6215 | GO:0005737 | vacuolar protein sorting-associated protein |
| Fv6225 | GO:0004819 | estradiol 17-beta-dehydrogenase 1 |
| Fv6230 | GO:0046872 | xaa-pro aminopeptidase |
| Fv6232 | GO:0005840 | tryptophanyl-trna synthetase |
| Fv6253 | GO:0003924 | gtp-binding protein guf1 |
| Fv6257 | GO:0006350 | dna-directed rna polymerase ii subunit rpb2 |
| Fv6278 | GO:0003723 | atp-dependent rna helicase dbp3 |
| Fv6290 | GO:0003723 | drap deaminase |
| Fv6318 | GO:0005737 | bifunctional purine biosynthetic protein |
| Fv6352 | GO:0005840 | 40s ribosomal protein s14 |
| Fv6373 | GO:0003723 | rrna biogenesis protein |
| Fv6382 | GO:0003746 | ribosome biogenesis protein |
| Fv6440 | GO:0003723 | rna helicase |
| Fv6451 | GO:0005737 | domain protein |
| Fv6471 | GO:0006629 | acetoacetyl- synthase |
| Fv6472 | GO:0030170 | serine palmitoyltransferase |
| Fv6515 | GO:0043581 | gtp binding protein |
| Fv6570 | GO:0016491 | aldo-keto reductase |
| Fv6609 | GO:0009851 | sodium p-type |
| Fv6630 | GO:0016301 | ribose-phosphate pyrophosphokinase |
| Fv6632 | GO:0031167 | trna (uridine-2 -o-)-methyltransferase trm7 |
| Fv6654 | GO:0016887 | translation initiation regulator |
| Fv6670 | GO:0005388 | calcium p-type atpase |
| Fv6682 | GO:0003988 | 3-ketoacyl-coa thiolase peroxisomal a precursor |
| Fv6685 | GO:0032312 | methionine-r-sulfoxide reductase |
| Fv6693 | GO:0055114 | bacilysin biosynthesis oxidoreductase bacc |
| Fv6705 | GO:0051287 | d-3-phosphoglycerate dehydrogenase |
| Fv6716 | GO:0009851 | hsp70-like protein |
| Fv6720 | GO:0003746 | elongation factor tu gtp binding domain protein |
| Fv6721 | GO:0005737 | t-complex protein 1 subunit epsilon |
| Fv6739 | GO:0045454 | 37s ribosomal protein rsm22 |
| Fv6747 | GO:0022891 | mfs monosaccharide transporter |
| Fv6748 | GO:0005488 | pre-mrna splicing factor rna helicase |
| Fv6760 | GO:0016787 | pre-mrna splicing factor atp-dependent rna helicase prp43 |
| Fv6761 | GO:0009941 | atp-dependent rna |
| Fv6772 | GO:0043581 | phosphoribosylformylglycinamidine synthase |
| Fv6791 | GO:0008152 | amp-binding enzyme |
| Fv6837 | GO:0006541 | bifunctional pyrimidine biosynthesis protein |
| Fv6844 | GO:0009405 | peroxisomal multifunctional beta-oxidation protein |
| Fv6875 | GO:0009851 | abc multidrug |
| Fv6890 | GO:0055114 | sorbitol dehydrogenase |
| Fv6902 | GO:0003861 | 3-isopropylmalate dehydratase |
| Fv6912 | GO:0051287 | isocitrate nad-dependent |
| Fv6919 | GO:0005737 | elongation factor 2 |
| Fv6920 | GO:0006555 | methylenetetrahydrofolate reductase 1 |
| Fv6941 | GO:0010181 | nadph-dependent fmn fad containing |
| Fv6945 | GO:0006526 | carbamoyl-phosphate small subunit |
| Fv7007 | GO:0009851 | dna topoisomerase 2 |
| Fv7025 | GO:0030170 | ornithine aminotransferase |
| Fv7027 | GO:0055114 | succinate-semialdehyde dehydrogenase |
| Fv7029 | GO:0055114 | cytochrome p450 oxidoreductase |
| Fv7041 | GO:0046872 | protein nbp35 |
| Fv7052 | GO:0003723 | atp-dependent rna helicase dbp10 |
| Fv7054 | GO:0005737 | mitochondrial chaperone |
| Fv7089 | GO:0003723 | atp-dependent rna helicase dbp7 |
| Fv7092 | GO:0055114 | aldehyde dehydrogenase |
| Fv7108 | GO:0005524 | dna mismatch repair protein |
| Fv7112 | GO:0009851 | atp-dependent protease la |
| Fv7133 | GO:0016887 | abc |
| Fv7162 | GO:0009851 | atp-binding cassette sub-family g member 2 |
| Fv7170 | GO:0003924 | elongation factor g |
| Fv7174 | GO:0008152 | acetyl- acetyltransferase |
| Fv7188 | GO:0031072 | domain protein |
| Fv7189 | GO:0030528 | domain protein |
| Fv7196 | GO:0009851 | dead box rna helicase |
| Fv7215 | GO:0009851 | mitochondrial hsp70 chaperone |
| Fv7234 | GO:0008081 | mate efflux family protein |
| Fv7246 | GO:0009851 | atp-dependent protease la |
| Fv7270 | GO:0006457 | 10 kda heat shock mitochondrial |
| Fv7274 | GO:0017111 | aaa family atpase |
| Fv7306 | GO:0004222 | metallopeptidase |
| Fv7342 | GO:0055114 | short-chain dehydrogenase |
| Fv7374 | GO:0005777 | d-amino acid oxidase |
| Fv7405 | GO:0009851 | p-type copper atpase |
| Fv7422 | GO:0055114 | d-hydroxyacid dehydrogenase |
| Fv7440 | GO:0043581 | molybdenum cofactor biosynthetic protein |
| Fv7465 | GO:0009085 | homoaconitase |
| Fv7490 | GO:0008152 | triosephosphate isomerase |
| Fv7491 | GO:0016888 | deoxyribonuclease tat-d |
| Fv7499 | GO:0051287 | isocitrate dehydrogenase subunit mitochondrial precursor |
| Fv7530 | GO:0008270 | zinc alcohol |
| Fv7531 | GO:0055114 | polyketide |
| Fv7532 | GO:0030170 | class ii aminotransferase 8-amino-7-oxononanoate synthase |
| Fv7603 | GO:0045454 | monothiol glutaredoxin-5 |
| Fv7604 | GO:0003824 | phosphopantothenoylcysteine decarboxylase |
| Fv7612 | GO:0005388 | plasma membrane calcium-transporting atpase 2 |
| Fv7616 | GO:0005737 | translation releasing factor rf-1 |
| Fv7621 | GO:0009851 | abc transporter cdr4 |
| Fv7632 | GO:0016491 | short chain dehydrogenase reductase |
| Fv7644 | GO:0046872 | mn superoxide dismutase |
| Fv7675 | GO:0022891 | mfs glucose |
| Fv7685 | GO:0009851 | na k atpase alpha 1 |
| Fv7706 | GO:0008415 | alcohol dehydrogenase domain protein |
| Fv7712 | GO:0055114 | aldehyde dehydrogenase |
| Fv7743 | GO:0006541 | ctp synthase |
| Fv7794 | GO:0005737 | seryl-trna synthetase |
| Fv7870 | GO:0022891 | mfs myo-inositol |
| Fv7898 | GO:0003723 | atp-dependent rna helicase has1 |
| Fv7912 | GO:0009851 | abc multidrug transporter |
| Fv7915 | GO:0016874 | long-chain-fatty-acid- ligase |
| Fv7937 | GO:0009851 | atp-dependent clp protease atp-binding subunit clpx |
| Fv7939 | GO:0045261 | atp synthase beta mitochondrial |
| Fv7942 | GO:0017111 | mitochondrial aaa |
| Fv7944 | GO:0004591 | 2-oxoglutarate dehydrogenase e1 component |
| Fv7954 | GO:0006072 | glycerol-3-phosphate dehydrogenase |
| Fv7966 | GO:0003899 | dna-directed rna polymerase i subunit rpa1 |
| Fv7973 | GO:0003824 | short-chain dehydrogenase reductase sdr |
| Fv7979 | GO:0055114 | short chain |
| Fv7988 | GO:0009851 | abc transporter |
| Fv7989 | GO:0016769 | classes i and ii family protein |
| Fv7991 | GO:0003746 | translation elongation factor |
| Fv7992 | GO:0047046 | isocitrate dehydrogenase |
| Fv7996 | GO:0009987 | methionine aminopeptidase |
| Fv8005 | GO:0003887 | dna-directed polymerase |
| Fv8022 | GO:0030170 | homocysteine synthase |
| Fv8027 | GO:0044262 | citrate synthase |
| Fv8032 | GO:0009851 | plasma membrane atpase |
| Fv8041 | GO:0006537 | glutamate synthase |
| Fv8065 | GO:0055114 | short-chain dehydrogenase reductase family |
| Fv8082 | GO:0016491 | 3-oxoacyl-(acyl-carrier-protein) reductase |
| Fv8085 | GO:0009851 | intermembrane space aaa protease iap-1 |
| Fv8111 | GO:0005507 | copper-transporting atpase 2 |
| Fv8112 | GO:0016301 | ribose-phosphate pyrophosphokinase |
| Fv8131 | GO:0009851 | peroxisomal abc transporter |
| Fv8167 | GO:0005840 | 54s ribosomal protein l9 |
| Fv8172 | GO:0009851 | atp dependent rna |
| Fv8188 | GO:0016212 | aminotransferase class i and ii |
| Fv8203 | GO:0006526 | acetylornithine aminotransferase |
| Fv8231 | GO:0055114 | zinc-binding alcohol |
| Fv8251 | GO:0006508 | peptidase family m1 |
| Fv8254 | GO:0005737 | 26s protease regulatory subunit 8 |
| Fv8390 | GO:0009851 | abc multidrug transporter mdr1 |
| Fv8400 | GO:0022891 | mfs maltose |
| Fv8422 | GO:0055114 | aldehyde dehydrogenase |
| Fv8469 | No Annotation |  |
| Fv8497 | GO:0008152 | 4-coumarate- ligase |
| Fv8550 | GO:0016491 | dtdp-glucose -dehydratase |
| Fv8564 | GO:0008270 | alcohol dehydrogenase |
| Fv8593 | GO:0009851 | vacuolar abc heavy metal transporter |
| Fv8613 | GO:0005811 | enoyl- hydratase |
| Fv8614 | GO:0016874 | nonribosomal peptide synthetase 12 |
| Fv8627 | GO:0022891 | mfs sugar |
| Fv8639 | GO:0008152 | short chain |
| Fv8665 | GO:0008152 | aconitase family protein |
| Fv8714 | GO:0031177 | nonribosomal peptide |
| Fv8715 | GO:0031177 | nonribosomal peptide |
| Fv8768 | GO:0022891 | mfs sugar transporter |
| Fv8771 | GO:0009851 | abc multidrug |
| Fv8802 | GO:0055114 | polyketide |
| Fv8891 | GO:0045254 | pyruvate dehydrogenase dihydrolipoamide acetyltransferase |
| Fv8902 | GO:0016887 | elongation factor 3 |
| Fv8911 | GO:0055114 | aldehyde dehydrogenase aldh |
| Fv8922 | GO:0055114 | succinate-semialdehyde dehydrogenase |
| Fv8930 | GO:0005737 | seryl-trna synthetase |
| Fv8962 | GO:0047956 | aldehyde reductase |
| Fv8988 | GO:0003988 | 3-ketoacyl- ketothiolase |
| Fv8990 | GO:0009851 | p-type na+-atpase |
| Fv9007 | GO:0055114 | short chain oxidoreductase |
| Fv9018 | GO:0055114 | short chain dehydrogenase reductase family |
| Fv9019 | GO:0016301 | ribose-phosphate pyrophosphokinase 1 |
| Fv9046 | GO:0048037 | amp-dependent synthetase and ligase |
| Fv9049 | GO:0005737 | 26s protease regulatory subunit 6a |
| Fv9072 | GO:0017111 | mitochondrial chaperone bcs1 |
| Fv9081 | GO:0005622 | gtp-binding protein |
| Fv9091 | GO:0003723 | atp dependent rna helicase |
| Fv9097 | GO:0009851 | heavy metal tolerance protein |
| Fv9112 | GO:0043581 | acetolactate synthase |
| Fv9113 | GO:0055114 | aldehyde dehydrogenase |
| Fv9136 | GO:0008152 | dihydroxyacetone synthase |
| Fv9137 | GO:0016491 | short chain dehydrogenase reductase family |
| Fv9139 | GO:0022891 | mfs sugar |
| Fv9181 | GO:0016491 | 3-oxoacyl-(acyl-carrier-protein) reductase |
| Fv9192 | GO:0016491 | glycerol dehydrogenase |
| Fv9201 | GO:0009851 | atp-dependent rrna helicase rrp3 |
| Fv9205 | GO:0055114 | glycerate-and formate-dehydrogenase |
| Fv9231 | GO:0008270 | alcohol dehydrogenase |
| Fv9255 | GO:0006457 | peptidyl-prolyl cis-trans |
| Fv9279 | No Annotation |  |
| Fv9309 | GO:0045454 | fad-dependent pyridine nucleotide-disulfide reduction |
| Fv9327 | GO:0016491 | dtdp-d-glucose -dehydratase |
| Fv9373 | GO:0004386 | sodium hydrogen exchanger |
| Fv9392 | GO:0009851 | vacuolar abc heavy metal transporter |
| Fv9395 | GO:0009851 | cell division control protein cdc48 |
| Fv9411 | GO:0030170 | ornithine aminotransferase |
| Fv9421 | GO:0030170 | 4-aminobutyrate aminotransferase |
| Fv9438 | GO:0009851 | abc metal ion |
| Fv9458 | GO:0009851 | abc |
| Fv9468 | GO:0009851 | peroxisomal biogenesis factor 6 |
| Fv9471 | GO:0008152 | phenylacetyl- |
| Fv9489 | GO:0055114 | succinate dehydrogenase iron-sulfur subunit |
| Fv9495 | GO:0055114 | glyoxylate reductase |
| Fv9556 | GO:0005789 | estradiol 17-beta-dehydrogenase 12-b |
| Fv9617 | GO:0022891 | mfs sugar |
| Fv9647 | GO:0009851 | cation transport atpase |
| Fv9650 | GO:0008152 | enoyl- hydratase isomerase family protein |
| Fv9666 | GO:0022891 | general alpha-glucoside permease |
| Fv9667 | GO:0016491 | aldo-keto reductase |
| Fv9724 | GO:0055114 | short-chain dehydrogenase reductase sdr |
| Fv9726 | GO:0022891 | mfs maltose permease |
| Fv9742 | GO:0016491 | xylose and arabinose reductase |
| Fv9806 | GO:0055114 | short chain dehydrogenase |
| Fv9868 | GO:0005737 | cystathionine beta-synthase |
| Fv9873 | GO:0016491 | short-chain dehydrogenase reductase |
| Fv9957 | GO:0022891 | mfs monosaccharide |
| Fv9987 | GO:0055114 | short-chain |
| Fv9990 | GO:0008152 | fumarylacetoacetate hydrolase family protein |
| Fv9993 | GO:0055114 | short-chain dehydrogenase reductase sdr |
| Fv10022 | GO:0016491 | isoepoxydon dehydrogenase |
| Fv10026 | GO:0016491 | #NAME? |
| Fv10031 | GO:0030170 | aromatic aminotransferase |
| Fv10040 | GO:0008270 | alcohol dehydrogenase |
| Fv10047 | GO:0009851 | atp-dependent dna family |
| Fv10055 | GO:0046872 | short-chain dehydrogenase |
| Fv10092 | GO:0055114 | short-chain dehydrogenase |
| Fv10144 | GO:0030170 | pyridoxal-5 -phosphate-dependent protein beta subunit |
| Fv10150 | GO:0009851 | atp-dependent rna helicase mss116 |
| Fv10160 | GO:0022891 | mfs quinate |
| Fv10162 | GO:0055114 | d-arabinitol dehydrogenase |
| Fv10175 | GO:0008152 | 4-coumarate- ligase |
| Fv10286 | GO:0030170 | class iii |
| Fv10333 | GO:0008874 | short-chain dehydrogenase |
| Fv10339 | GO:0009851 | multidrug resistance protein 1 |
| Fv10340 | GO:0009851 | multidrug resistance protein 1 |
| Fv10382 | GO:0055114 | 2-deoxy-d-gluconate 3-dehydrogenase |
| Fv10384 | GO:0016491 | d-galacturonic acid reductase |
| Fv10521 | GO:0008483 | ornithine aminotransferase |
| Fv10537 | GO:0016874 | phenylacetyl- ligase |
| Fv10608 | GO:0008168 | ubiquinone biosynthesis methlytransferase |
| Fv10650 | GO:0055114 | betaine aldehyde dehydrogenase |
| Fv10654 | GO:0048037 | nonribosomal siderophore peptide |
| Fv10676 | GO:0022891 | mfs hexose |
| Fv10698 | GO:0003723 | atp-dependent rna helicase mak5 |
| Fv10709 | GO:0016021 | sulfate permease |
| Fv10725 | GO:0016491 | short chain dehydrogenase reductase |
| Fv10733 | GO:0003677 | 3-hydroxymethyl-3-methylglutaryl-coenzyme a lyase |
| Fv10735 | GO:0017111 | mitochondrial chaperone |
| Fv10745 | GO:0008152 | long-chain-fatty-acid- ligase |
| Fv10747 | GO:0055114 | aldehyde dehydrogenase |
| Fv10775 | GO:0022891 | mfs monosaccharide |
| Fv10794 | GO:0009851 | atp-binding cassette transporter |
| Fv10811 | GO:0030170 | class iii |
| Fv10876 | GO:0055114 | aldehyde dehydrogenase |
| Fv10883 | GO:0016491 | glycerol dehydrogenase |
| Fv10894 | No Annotation |  |
| Fv10895 | No Annotation |  |
| Fv10898 | GO:0016491 | nadp-dependent l-serine l-allo-threonine dehydrogenase ydfg |
| Fv10899 | GO:0008152 | d-xylose reductase |
| Fv10908 | GO:0016020 | sugar transporter |
| Fv10918 | GO:0030170 | 8-amino-7-oxononanoate synthase |
| Fv10934 | GO:0046872 | cytosolic fe-s cluster assembling factor cfd1 |
| Fv10941 | GO:0005525 | signal recognition particle 54 kda protein |
| Fv10955 | GO:0003842 | delta-1-pyrroline-5-carboxylate dehydrogenase |
| Fv10982 | GO:0016884 | d-mandelate |
| Fv10995 | GO:0009851 | sodium p-type |
| Fv11011 | GO:0004811 | trna isopentenyltransferase |
| Fv11013 | GO:0009851 | atp-dependent bile acid permease |
| Fv11028 | GO:0055114 | short-chain dehydrogenase reductase family |
| Fv11063 | GO:0009851 | atp-dependent rna helicase dhx8 |
| Fv11069 | GO:0009851 | calcium-transporting atpase 3 |
| Fv11137 | GO:0003723 | atp-dependent rna helicase dbp4 |
| Fv11139 | GO:0050662 | udp-glucose 4-epimerase |
| Fv11140 | GO:0008270 | quinone oxidoreductase |
| Fv11166 | GO:0055114 | 3-oxoacyl-(acyl-carrier-protein) reductase |
| Fv11179 | GO:0004726 | low molecular weight phosphotyrosine protein phosphatase |
| Fv11202 | GO:0016021 | hexose transporter |
| Fv11217 | GO:0016491 | short-chain oxidoreductase |
| Fv11242 | GO:0016874 | peroxisomal-coenzyme a synthetase |
| Fv11259 | GO:0004326 | dihydrofolate synthetase fol3 |
| Fv11284 | GO:0016491 | short-chain dehydrogenase |
| Fv11293 | GO:0016616 | glyoxylate reductase |
| Fv11423 | GO:0005737 | guanylate kinase |
| Fv11437 | GO:0009851 | abc multidrug |
| Fv11486 | GO:0004825 | methionyl-trna synthetase |
| Fv11517 | GO:0005737 | gtp cyclohydrolase i |
| Fv11521 | GO:0003743 | eukaryotic initiation factor 4a-12 |
| Fv11541 | GO:0008152 | enoyl- hydratase |
| Fv11587 | GO:0016491 | short chain oxidoreductase |
| Fv11594 | GO:0009851 | abc |
| Fv11610 | GO:0009374 | acetyl- propionyl-coenzyme a carboxylase alpha chain |
| Fv11697 | GO:0009851 | mitochondrial aaa |
| Fv11713 | GO:0016021 | sugar transporter (hexose transporter |
| Fv11810 | GO:0008415 | polyketide synthase |
| Fv11821 | GO:0003677 | sulfate permease |
| Fv11827 | GO:0017111 | abc multidrug |
| Fv11854 | GO:0009851 | multidrug resistance protein cdr1 |
| Fv11887 | GO:0016491 | short-chain dehydrogenase reductase sdr |
| Fv11896 | GO:0008415 | polyketide synthase |
| Fv11904 | GO:0022891 | mfs monosaccharide transporter |
| Fv11921 | GO:0009851 | abc |
| Fv11928 | GO:0008152 | phenylacetyl- ligase |
| Fv11947 | GO:0005622 | protein |
| Fv11971 | GO:0016491 | short-chain dehydrogenase |
| Fv11978 | GO:0005524 | dna mismatch repair protein |
| Fv12010 | No Annotation |  |
| Fv12040 | GO:0055114 | gluconate 5-dehydrogenase |
| Fv12051 | GO:0009374 | carbamoyl-phosphate synthase l atp-binding |
| Fv12059 | GO:0008270 | alcohol dehydrogenase 2 |
| Fv12115 | GO:0016491 | nadp-dependent l-serine l-allo-threonine dehydrogenase ydfg |
| Fv12119 | GO:0055114 | lung carbonyl |
| Fv12139 | GO:0016491 | short-chain dehydrogenase |
| Fv12168 | GO:0055114 | short chain type |
| Fv12294 | GO:0016787 | fumarylacetoacetate hydrolase family protein |
| Fv12409 | GO:0005737 | phosphoglycerate kinase |
| Fv12444 | GO:0022891 | hexose transporter protein |
| Fv12485 | GO:0005488 | pyrroline-5-carboxylate reductase |
| Fv12498 | GO:0055114 | l- -butanediol dehydrogenase acetoinreductase |
| Fv12507 | GO:0016491 | l-xylulose reductase |
| Fv12572 | GO:0008270 | alcohol dehydrogenase |
| Fv12589 | GO:0005488 | non-reducing polyketide synthase |
| Fv12594 | GO:0031177 | polyketide |
| Fv12688 | GO:0043581 | gmp synthase |
| Fv12704 | GO:0016491 | short chain dehydrogenase reductase |
| Fv12722 | GO:0022891 | msf superfamily transporter |
| Fv12758 | GO:0017111 | mitochondrial aaa |
| Fv12765 | GO:0009851 | multidrug resistance protein 3 (p glycoprotein 3) |
| Fv12816 | GO:0055085 | abc transporter |
| Fv12847 | GO:0030170 | acetylornithine aminotransferase |
| Fv12865 | GO:0009851 | abc transporter |
| Fv12872 | GO:0055114 | aldehyde dehydrogenase aldh |
| Fv12876 | GO:0006573 | methylmalonate-semialdehyde dehydrogenase |
| Fv12878 | GO:0022891 | sugar transporter stl1 |
| Fv12891 | GO:0016620 | succinate-semialdehyde dehydrogenase |
| Fv12913 | GO:0008415 | methylcitrate synthase precursor |
| Fv12946 | GO:0030170 | acetylornithine aminotransferase |
| Fv12951 | GO:0009851 | copper-exporting p-type atpase a |
| Fv12969 | GO:0010033 | acetoacetyl- synthetase |
| Fv12972 | GO:0016021 | amino acid permease |
| Fv13007 | GO:0055114 | succinate-semialdehyde dehydrogenase |
| Fv13014 | GO:0022891 | mfs sugar transporter |
| Fv13101 | GO:0016021 | mfs hexose |
| Fv13110 | GO:0003862 | tartrate dehydrogenase |
| Fv13117 | GO:0055114 | vanillin |
| Fv13140 | GO:0055114 | short-chain dehydrogenase reductase sdr |
| Fv13145 | GO:0055114 | short chain dehydrogenase |
| Fv13165 | GO:0055114 | aldehyde dehydrogenase aldh |
| Fv13189 | GO:0055114 | short-chain dehydrogenase reductase sdr |
| Fv13220 | GO:0016491 | major facilitator superfamily protein |
| Fv13245 | GO:0030170 | 4-aminobutyrate aminotransferase |
| Fv13281 | GO:0050662 | udp-glucose 4- |
| Fv13302 | GO:0022891 | hexose transporter |
| Fv13319 | GO:0008152 | short chain dehydrogenase reductase family |
| Fv13334 | GO:0009851 | multidrug resistance protein 3 (p glycoprotein 3) |
| Fv13377 | GO:0031177 | polyketide synthase |
| Fv13398 | GO:0003842 | delta-1-pyrroline-5-carboxylate dehydrogenase |
| Fv13444 | GO:0055114 | aldehyde dehydrogenase family |
| Fv13445 | GO:0044271 | short-chain dehydrogenase reductase family |
| Fv13468 | GO:0046933 | vacuolar atp synthase subunit b |
| Fv13477 | GO:0016874 | amp-binding enzyme |
| Fv13480 | GO:0045261 | mitochondrial f1 atpase subunit |
| Fv13509 | GO:0022891 | lactose permease |
| Fv13512 | GO:0016787 | fumarylacetoacetate hydrolase family protein |
| Fv13535 | No Annotation |  |
| Fv13541 | GO:0009851 | abc transporter |
| Fv13558 | GO:0006573 | methylmalonate-semialdehyde dehydrogenase |
| Fv13572 | GO:0022891 | maltose permease |
| Fv13627 | No Annotation |  |
| Fv13650 | GO:0005488 | succinate semialdehyde dehydrogenase |
| Fv13655 | GO:0022891 | mfs maltose permease |
| Fv13688 | GO:0009851 | abc |
| Fv13739 | GO:0006810 | sugar transporter |
| Fv13749 | GO:0050085 | l-xylulose reductase |
| Fv13781 | GO:0009851 | abc bile acid |
| Fv13873 | GO:0016853 | enoyl- hydratase isomerase family protein |
| Fv13884 | GO:0016853 | fumarylacetoacetate hydrolase family protein |
| Fv13985 | GO:0009851 | casein kinase i |
| Fv14002 | GO:0016491 | glycerol dehydrogenase |
| Fv14005 | GO:0006081 | aldehyde dehydrogenase |
| Fv14007 | GO:0003824 | duf1446 domain-containing protein |
| Fv14028 | GO:0042744 | peroxiredoxin 1 variant 2 |
| Fv14033 | GO:0005488 | short chain dehydrogenase family |
| Fv14046 | GO:0055114 | glycerate-and formate-dehydrogenase |
| Fv14088 | GO:0009851 | mdr efflux pump abc3 |
| Fv14092 | GO:0009851 | abc transporter |
| Fv14094 | GO:0055085 | efflux pump antibiotic resistance |
| Fv14096 | GO:0016616 | glycerate dehydrogenase |
| Fv14144 | GO:0016491 | oxidoreductase |
| Fv14154 | GO:0022891 | myo-inositol transporter |
| Fv14198 | GO:0016491 | short-chain oxidoreductase |
| Fv14257 | GO:0008152 | superoxide mitochondrial precursor |
| Fv14263 | GO:0003824 | short-chain dehydrogenase reductase family |
| Fv14314 | GO:0005737 | gtp cyclohydrolase i |
| Fv14320 | GO:0017111 | protein |
| Fv14335 | GO:0022891 | mfs quinate |
| Fv14418 | GO:0022891 | mfs glucose |
| Fv14433 | GO:0017111 | protein |
| Fv14467 | GO:0055114 | 2-deoxy-d-gluconate 3-dehydrogenase |
| Fv14477 | GO:0009851 | abc multidrug |
| Fv14494 | GO:0008152 | short-chain dehydrogenase reductase |
| Fv14506 | GO:0055114 | short-chain dehydrogenase reductase family |
| Fv14508 | GO:0016491 | short chain |
| Fv14533 | GO:0008152 | acetyl-coenzyme a synthetase |
| Fv14544 | GO:0016620 | succinate-semialdehyde dehydrogenase |
| Fv14579 | GO:0003824 | short-chain dehydrogenase reductase family |
| Fv14580 | GO:0055114 | succinate-semialdehyde dehydrogenase |
| Fv14585 | No Annotation |  |
| Fv14617 | GO:0031177 | polyketide |
| Fv14625 | GO:0005488 | polyketide |
| Fv14638 | GO:0048034 | protoheme ix farnesyltransferase |
| Fv14662 | GO:0018478 | methylmalonate-semialdehyde dehydrogenase |
| Fv14783 | GO:0055114 | 3-ketoacyl-acyl carrier protein reductase |
| Fv14784 | GO:0008152 | 4-coumarate- ligase |
