# Supplementary material for: The Genome Sequence of the Fungal Pathogen Fusarium virguliforme That Causes Sudden Death Syndrome in Soybean
Source: PLoS One. 2014 Jan 14;9(1):e81832. doi: 10.1371/journal.pone.0081832 (PMC3891557; doi:10.1371/journal.pone.0081832)
Supplement: Table S10 — GO annotation of 98 of the 1,332 unique F. virguliforme genes. (DOC) [file pone.0081832.s019.doc]

**Table S10.** GO annotation of 98 of the 1,332 unique *F. virguliforme* genes.

| **Gene Name** | **Seq. Description** | **Seq. Length** | **Min. E -Value** | **Mean Similarity** | **GOs** |
| --- | --- | --- | --- | --- | --- |
| *Fv7* | helix-turn-helix domain-containing protein | 203 | 6.85E-19 | 46.75% |  |
| *Fv11* | amp-dependent synthetase ligase | 429 | 2.24E-55 | 49.30% | F:catalytic activity |
| *Fv387* | hypothetical protein FG00775.1 [Gibberella zeae PH-1] | 112 | 3.99E-12 | 58.00% |  |
| *Fv1253* | f-box domain protein | 717 | 6.02E-41 | 38.63% |  |
| *Fv1399* | glutaredoxin domain-containing protein | 103 | 1.25E-32 | 62.55% | P:cell redox homeostasis; F:molecular_function; F:electron carrier activity; F:protein disulfide oxidoreductase activity; C:cellular_component |
| *Fv1851* | gnat | 199 | 1.28E-53 | 59.55% | F:catalytic activity |
| *Fv2709* | predicted protein [Verticillium albo-atrum VaMs.102] | 247 | 3.87E-17 | 74.50% |  |
| *Fv3011* | predicted protein [Chaetomium globosum CBS 148.51] | 296 | 3.74E-19 | 44.00% |  |
| *Fv3346* | hypothetical protein PMAA_068960 [Penicillium marneffei ATCC 18224] | 437 | 1.20E-38 | 52.00% |  |
| *Fv4595* | predicted protein [Laccaria bicolor S238N-H82] | 753 | 2.78E-16 | 44.00% | F:nucleotide binding; F:nucleoside-triphosphatase activity; P:auxin biosynthetic process |
| *Fv4600* | predicted protein [Laccaria bicolor S238N-H82] | 742 | 5.92E-17 | 43.67% | F:nucleotide binding; F:nucleoside-triphosphatase activity; P:auxin biosynthetic process |
| *Fv4715* | hypothetical protein NFIA_002470 [Neosartorya fischeri NRRL 181] | 410 | 4.56E-17 | 44.00% |  |
| *Fv4772* | retrotransposon unclassified | 223 | 1.07E-40 | 51.10% | P:DNA metabolic process; F:nucleic acid binding; C:intracellular organelle |
| *Fv4834* | atp-binding protein | 202 | 5.63E-47 | 56.15% | F:ATP binding; P:cell killing |
| *Fv4849* | hypothetical protein NCU04723 [Neurospora crassa OR74A] | 395 | 1.69E-15 | 48.60% |  |
| *Fv5955* | ankyrin repeat protein | 301 | 2.52E-51 | 55.85% | F:kinase activity; F:protein kinase activity; F:ATP binding; P:protein amino acid phosphorylation |
| *Fv6112* | predicted protein [Aspergillus terreus NIH2624] | 338 | 7.48E-43 | 49.50% | F:ATP binding; P:apoptosis; F:binding |
| *Fv6229* | hypothetical protein NCU00111 [Neurospora crassa OR74A] | 146 | 1.20E-12 | 66.00% |  |
| *Fv6750* | hypothetical protein FG01518.1 [Gibberella zeae PH-1] | 307 | 6.13E-13 | 64.00% |  |
| *Fv6980* | predicted protein [Ajellomyces dermatitidis ER-3] | 237 | 4.35E-13 | 46.00% |  |
| *Fv7056* | filamentation induced by camp protein fic | 392 | 4.88E-68 | 49.30% | P:regulation of transcription, DNA-dependent; F:nucleotide binding; F:DNA binding; F:transcription factor activity |
| *Fv7808* | prdm5 protein | 553 | 6.61E-11 | 39.00% |  |
| *Fv7971* | protein | 391 | 4.02E-116 | 67.65% | F:phosphopantetheine binding; F:zinc ion binding; F:transposase activity; P:biosynthetic process; P:oxidation reduction; F:acyl carrier activity; F:DNA binding; F:oxidoreductase activity; F:transferase activity; P:transposition, DNA-mediated; P:DNA integration; F:cofactor binding |
| *Fv8237* | hypothetical protein AN5279.2 [Aspergillus nidulans FGSC A4] | 439 | 1.26E-12 | 44.00% | P:regulation of transcription, DNA-dependent; F:zinc ion binding; F:transcription factor activity; C:nucleus |
| *Fv8247* | hypothetical protein SBI_06607 [Streptomyces bingchenggensis BCW-1] | 283 | 1.07E-17 | 50.33% |  |
| *Fv8312* | hypothetical protein SNOG_11241 [Phaeosphaeria nodorum SN15] | 74 | 7.99E-11 | 79.00% | F:catalytic activity; P:metabolic process; F:binding |
| *Fv8324* | hypothetical protein [Podospora anserina S mat+] | 706 | 2.41E-28 | 40.00% |  |
| *Fv8438* | hypothetical protein TSTA_066400 [Talaromyces stipitatus ATCC 10500] | 192 | 4.55E-43 | 61.25% |  |
| *Fv8495* | hypothetical protein [Podospora anserina S mat+] | 659 | 3.44E-13 | 40.00% |  |
| *Fv8501* | protein | 637 | 3.43E-74 | 46.25% | P:lipid metabolic process; F:hydrolase activity, acting on ester bonds; F:hydrolase activity |
| *Fv8666* | hypothetical protein SACT1DRAFT_6512 [Streptomyces sp. ACT-1] | 113 | 2.62E-13 | 64.00% | P:fruiting body development; P:hemolysis by symbiont of host erythrocytes |
| *Fv8696* | alpha beta hydrolase fold | 164 | 4.44E-17 | 56.47% | F:catalytic activity |
| *Fv8767* | unnamed protein product [Sordaria macrospora] | 183 | 6.60E-24 | 51.00% |  |
| *Fv9581* | pleurotolysin a | 170 | 4.62E-29 | 60.15% | P:fruiting body development; P:hemolysis by symbiont of host erythrocytes; P:hemolysis of cells in other organism; P:cytolysis |
| *Fv9606* | protein serine threonine kinase | 189 | 9.67E-62 | 70.36% | F:kinase activity |
| *Fv9689* | conserved hypothetical protein [Arthroderma otae CBS 113480] | 622 | 1.08E-55 | 54.20% |  |
| *Fv9691* | von willebrand factor type a domain-containing protein | 143 | 2.28E-12 | 52.00% |  |
| *Fv9863* | hypothetical protein [Podospora anserina S mat+] | 276 | 2.58E-12 | 46.00% |  |
| *Fv9988* | putative protein [Neurospora crassa] | 212 | 1.11E-19 | 63.00% |  |
| *Fv10073* | hypothetical protein [Podospora anserina S mat+] | 1370 | 4.45E-16 | 50.00% |  |
| *Fv10096* | hypothetical protein An15g02270 [Aspergillus niger] | 134 | 3.82E-35 | 72.00% |  |
| *Fv10103* | conserved hypothetical protein [Verticillium albo-atrum VaMs.102] | 233 | 2.42E-17 | 50.00% |  |
| *Fv10524* | hypothetical protein [Penicillium chrysogenum Wisconsin 54-1255] | 300 | 7.65E-86 | 66.67% |  |
| *Fv10525* | hypothetical protein SCHCODRAFT_60016 [Schizophyllum commune H4-8] | 160 | 6.72E-12 | 51.00% |  |
| *Fv10717* | hypothetical protein FG09246.1 [Gibberella zeae PH-1] | 228 | 2.72E-14 | 60.00% |  |
| *Fv10724* | hypothetical protein TRV_04951 [Trichophyton verrucosum HKI 0517] | 204 | 1.00E-23 | 53.00% |  |
| *Fv10748* | interferon-inducible gtpase- isoform cra_a | 290 | 1.30E-16 | 49.95% | F:hydrolase activity, acting on acid anhydrides; F:guanyl ribonucleotide binding; C:cell part |
| *Fv10838* | hypothetical protein [Podospora anserina S mat+] | 154 | 4.86E-46 | 62.00% |  |
| *Fv10901* | transposable element tc3 | 355 | 1.36E-56 | 53.10% | F:transposase activity; P:DNA integration; P:transposition, DNA-mediated; F:DNA binding |
| *Fv10911* | transposase [Fusarium oxysporum f. sp. lycopersici] | 208 | 1.60E-111 | 58.60% | F:protein dimerization activity |
| *Fv10944* | hypothetical protein AN4796.2 [Aspergillus nidulans FGSC A4] | 131 | 3.64E-15 | 45.33% |  |
| *Fv11614* | hypothetical protein PMAA_088990 [Penicillium marneffei ATCC 18224] | 476 | 2.62E-16 | 44.00% |  |
| *Fv11738* | hypothetical protein [Aspergillus oryzae RIB40] | 180 | 3.10E-21 | 60.50% |  |
| *Fv11800* | predicted protein [Coccidioides immitis RS] | 101 | 9.70E-15 | 65.75% |  |
| *Fv11876* | conserved hypothetical protein [Trichophyton verrucosum HKI 0517] | 369 | 1.19E-16 | 52.75% |  |
| *Fv12141* | hypothetical protein Bcav_0812 [Beutenbergia cavernae DSM 12333] | 227 | 2.67E-12 | 52.00% |  |
| *Fv12146* | family protein | 329 | 1.74E-17 | 42.60% | P:protein secretion; F:binding; P:metabolic process; C:membrane; F:catalytic activity; F:protein transporter activity |
| *Fv12291* | conserved hypothetical protein [Verticillium albo-atrum VaMs.102] | 139 | 2.06E-45 | 85.00% |  |
| *Fv12306* | conserved hypothetical protein [Ajellomyces capsulatus H143] | 114 | 1.13E-17 | 69.78% | F:molecular_function; P:biological_process; C:cellular_component |
| *Fv12316* | predicted protein [Chaetomium globosum CBS 148.51] | 597 | 1.79E-16 | 39.50% | F:metal ion binding; F:zinc ion binding |
| *Fv12317* | predicted protein [Coccidioides immitis RS] | 109 | 3.74E-13 | 61.80% |  |
| *Fv12347* | conserved hypothetical protein [Arthroderma otae CBS 113480] | 381 | 1.74E-14 | 40.00% |  |
| *Fv12527* | hypothetical protein TSTA_012410 [Talaromyces stipitatus ATCC 10500] | 354 | 5.14E-16 | 48.00% |  |
| *Fv12592* | ankyrin repeat protein | 222 | 8.98E-14 | 61.36% |  |
| *Fv12700* | predicted protein [Chaetomium globosum CBS 148.51] | 273 | 6.36E-25 | 49.50% |  |
| *Fv12963* | beta-lactamase domain protein | 630 | 0 | 75.85% | F:sterol binding; F:hydrolase activity |
| *Fv13021* | protein | 1027 | 1.10E-85 | 43.95% |  |
| *Fv13042* | predicted protein [Ajellomyces capsulatus NAm1] | 235 | 3.35E-42 | 61.50% | F:ribonuclease H activity; F:RNA-directed DNA polymerase activity; P:RNA-dependent DNA replication; F:RNA binding; F:zinc ion binding |
| *Fv13310* | predicted protein [Arthroderma otae CBS 113480] | 226 | 2.92E-14 | 51.00% |  |
| *Fv13438* | family protein | 436 | 2.14E-61 | 51.75% | P:propionate catabolic process; F:2-methylcitrate dehydratase activity |
| *Fv13556* | predicted protein [Botryotinia fuckeliana B05.10] | 690 | 7.44E-93 | 45.67% |  |
| *Fv13800* | hypothetical protein CHGG_08799 [Chaetomium globosum CBS 148.51] | 423 | 3.39E-24 | 44.00% | P:regulation of transcription, DNA-dependent; F:zinc ion binding; F:transcription factor activity; C:nucleus |
| *Fv13801* | hypothetical protein CHGG_02270 [Chaetomium globosum CBS 148.51] | 583 | 2.33E-61 | 58.25% | F:nucleic acid binding; P:DNA integration; F:protein dimerization activity; F:DNA binding |
| *Fv13816* | conserved hypothetical protein [Magnaporthe oryzae 70-15] | 332 | 1.24E-18 | 49.10% | F:nucleic acid binding; P:mycelium development; F:molecular_function; P:biological_process; C:cellular_component |
| *Fv13817* | fot5 transposase | 143 | 7.64E-14 | 71.00% | F:nucleic acid binding |
| *Fv14056* | hypothetical protein CHGG_06760 [Chaetomium globosum CBS 148.51] | 120 | 8.48E-13 | 54.00% |  |
| *Fv14070* | predicted protein [Ajellomyces capsulatus NAm1] | 260 | 1.03E-20 | 50.00% |  |
| *Fv14075* | protein | 214 | 2.20E-47 | 55.25% |  |
| *Fv14195* | hypothetical protein AN8532.2 [Aspergillus nidulans FGSC A4] | 408 | 2.93E-19 | 47.75% |  |
| *Fv14277* | conserved hypothetical protein [Verticillium albo-atrum VaMs.102] | 126 | 2.68E-16 | 59.00% |  |
| *Fv14291* | carbonic anhydrase 2 | 1330 | 0 | 45.45% | F:kinase activity; F:zinc ion binding; F:carbonate dehydratase activity; F:lyase activity; P:carbon utilization; C:intracellular; F:DNA binding |
| *Fv14293* | hypothetical protein TSTA_066400 [Talaromyces stipitatus ATCC 10500] | 244 | 2.11E-25 | 50.86% |  |
| *Fv14321* | hypothetical protein CHGG_06024 [Chaetomium globosum CBS 148.51] | 145 | 2.37E-26 | 64.25% | F:nucleic acid binding; P:DNA integration; F:DNA binding |
| *Fv14327* | predicted protein [Verticillium albo-atrum VaMs.102] | 163 | 1.25E-12 | 60.00% |  |
| *Fv14387* | atp-dependent dna helicase pif1 | 484 | 1.04E-49 | 56.67% |  |
| *Fv14426* | predicted protein [Ajellomyces capsulatus NAm1] | 239 | 4.72E-24 | 47.00% |  |
| *Fv14432* | hypothetical protein TSTA_066400 [Talaromyces stipitatus ATCC 10500] | 228 | 9.26E-19 | 45.75% |  |
| *Fv14448* | predicted protein [Verticillium albo-atrum VaMs.102] | 146 | 1.08E-23 | 72.00% |  |
| *Fv14454* | genome poly | 709 | 1.17E-177 | 55.00% | F:RNA binding; F:RNA-directed DNA polymerase activity; F:DNA binding; P:DNA integration; P:RNA-dependent DNA replication; P:DNA recombination |
| *Fv14561* | transposable element tc3 | 224 | 2.36E-38 | 53.90% | P:metabolic process; F:catalytic activity |
| *Fv14582* | metallo-beta-lactamase superfamily protein | 163 | 2.30E-78 | 69.10% | F:hydrolase activity |
| *Fv14596* | hypothetical protein CHGG_04534 [Chaetomium globosum CBS 148.51] | 156 | 2.35E-18 | 84.50% | F:microtubule motor activity; F:binding; P:lipid catabolic process; C:kinesin complex |
| *Fv14599* | hypothetical protein CHGG_00307 [Chaetomium globosum CBS 148.51] | 132 | 2.06E-12 | 72.87% | F:RNA-directed DNA polymerase activity; P:RNA-dependent DNA replication; F:RNA binding; F:hydrolase activity; F:zinc ion binding |
| *Fv14621* | conserved hypothetical protein [Ajellomyces capsulatus H143] | 254 | 6.68E-17 | 49.33% |  |
| *Fv14626* | polyketide synthase | 194 | 2.15E-24 | 53.25% | F:catalytic activity |
| *Fv14759* | gag polyprotein | 455 | 1.15E-59 | 55.05% | F:binding; P:mycelium development |
| *Fv14770* | beta-galactosidase alpha-peptide | 103 | 3.20E-46 | 94.15% |  |
| *Fv14830* | fot5 transposase | 112 | 2.96E-28 | 70.50% | F:nucleic acid binding; P:mycelium development |

*Fusarium virguliforme* (*Fv*) genes showing annotation using GO (Gene Ontology) database.
