# Supplementary material for: The Genome Sequence of the Fungal Pathogen Fusarium virguliforme That Causes Sudden Death Syndrome in Soybean
Source: PLoS One. 2014 Jan 14;9(1):e81832. doi: 10.1371/journal.pone.0081832 (PMC3891557; doi:10.1371/journal.pone.0081832)
Supplement: Table S11 — Identification of candidate 358 pathogenicity genes through interrogation of the F. virguliforme genome with the PHI database. (DOC) [file pone.0081832.s020.doc]

**Table S11.** Identification of candidate 358 pathogenicity genes through interrogation of the *F. virguliforme* genome with the PHI database.

| ***Fv* gene** | **Accession** | **Organism** | **Function/Role** | **Reference** |
| --- | --- | --- | --- | --- |
| *Fv48* | AAO25556.1 | *Candida albicans* | guanine nucleotide exchange factor Cdc24 | Eukaryotic Cell 2 (1), 9-18 (2003) |
| *Fv53* | EAK96144.1 | *Candida albicans SC5314* | potential dual specificity phosphatase | Proc. Natl. Acad. Sci. U.S.A. 101 (19), 7329-7334 (2004) |
| *Fv134* | CAA89133.1 | *Saccharomyces cerevisiae* | amidophosphoribosyltransferase | Direct Submission |
| *Fv202* | ABX79379.1 | *Magnaporthe grisea* | MADS-box MEF2 type transcription factor | Eukaryotic Cell 7 (5), 791-799 (2008) |
| *Fv216* | AAT84078.1 | *Phaeosphaeria nodorum* | mannitol 1-phosphate dehydrogenase | Direct Submission |
| *Fv232* | ABK64184.1 | *Cercospora nicotianae* | oxidoreductase | Microbiology (Reading, Engl.) 153 (PT 8), 2781-2790 (2007) |
| *Fv270* | AAA17547.1 | *Filobasidiella neoformans* | N-myristoyltransferase | J. Biol. Chem. 269 (4), 2996-3009 (1994) |
| *Fv274* | AAB09711.1 | *Filobasidiella neoformans* | phosphoribosylaminoimidazole carboxylase | J. Bacteriol. 175 (5), 1405-1411 (1993) |
| *Fv381* | AAK35180.1 | *Cochliobolus carbonum* | histone deacetylase 2" | Eukaryot Cell. 2002 Aug;1(4):538-47. |
| *Fv384* | AAQ16572.1 | *Botryotinia fuckeliana* | putative mitochondrial cyclophilin 1 | Mol. Microbiol. 50 (5), 1451-1465 (2003) |
| *Fv426* | AAC62257.1 | *Cryptococcus neoformans var. Grubii* | urease | Infect Immun. 2000 Feb;68(2):443-8. |
| *Fv449* | AAW72938.1 | *Cryptococcus neoformans* | white collar 2 | PLoS Biol. 3 (4), E95 (2005) |
| *Fv610* | EAK92427.1 | *Candida albicans SC5314* | spindle pole antigen | Proc. Natl. Acad. Sci. U.S.A. 101 (19), 7329-7334 (2004) |
| *Fv624* | BAE72680.1 | *Epichloe festucae* | NADPH oxidase | Plant Cell 18 (4), 1052-1066 (2006) |
| *Fv645* | AAY42143.1 | *Ustilago maydis* | G1 cyclin | Plant Cell 17 (12), 3544-3560 (2005) |
| *Fv730* | BAB43823.1 | *Candida albicans* | Root hair defective 3 GTP-binding protein (RHD3) | Direct Submission |
| *Fv748* | AAK81705.1 | *Cryptococcus neoformans var. grubii* | vacuolar (H+)-ATPase subunit | Direct Submission |
| *Fv790* | XP_380508.1 | *Fusarium graminearum* | hypothetical protein | Direct Submission |
| *Fv795* | ABD59787.1 | *Stagonospora nodorum* | Cm-dependent protein kinase B | Direct Submission |
| *Fv796* | AAD28749.1 | *Filobasidiella neoformans* | inositolphosphorylceramide synthase | Direct Submission |
| *Fv850* | XP_380552.1 | *Fusarium graminearum* | NUCM_NEUCR NADH-ubiquinone oxidoreductase | Direct Submission |
| *Fv890* | AAP72037.1 | *Cercospora zeae-maydis* | MAP kinase kinase kinase Czk3 | Mol. Plant Microbe Interact. 16 (9), 760-768 (2003) |
| *Fv908* | EDK03005.1 | *Magnaporthe grisea* | hypothetical protein | Nature 434 (7036), 980-986 (2005) |
| *Fv959* | AAM21494.1 | *Cryptococcus neoformans var. grubii* | protein kinase Sch9 | Curr. Genet. 46 (5), 247-255 (2004) |
| *Fv986* | AAO68935.1 | *Salmonella enterica subsp. enterica serovar Typhi str. Ty2* | unknown | J. Bacteriol. 185 (7), 2330-2337 (2003) |
| *Fv1034* | EAK92385.1 | *Candida albicans* | hypothetical protein | Proc. Natl. Acad. Sci. U.S.A. 101 (19), 7329-7334 (2004) |
| *Fv1039* | AAD51715.1 | *Candida albicans* | Arrestin (or S-antigen), N-terminal domain | Mol. Cell. Biol. 20 (3), 971-978 (2000) |
| *Fv1046* | ABJ98717.1 | *Aspergillus fumigatus* | zinc-responsiveness transcriptional activator | Mol. Microbiol. 64 (5), 1182-1197 (2007) |
| *Fv1065* | AAB63336.1 | *Ustilago maydis* | kinesin motor protein | EMBO J. 16 (12), 3464-3473 (1997) |
| *Fv1070* | ABO93363.1 | *Mycosphaerella graminicola* | eburicol 14 alpha-demethylase | Pest Manag. Sci. (2007) |
| *Fv1106* | EDK05593.1 | *Magnaporthe oryzae 70-15* | hypothetical protein | Nature 434 (7036), 980-986 (2005) |
| *Fv1118* | EAK95207.1 | *Candida albicans SC5314* | hypothetical protein" | Proc Natl Acad Sci U S A. 2004 May 11;101(19):7329-34. |
| *Fv1138* | AAK07740.1 | *Magnaporthe grisea* | P-type ATPase" | Plant Cell 13 (9), 1987-2004 (2001) |
| *Fv1141* | EDK03309.1 | *Magnaporthe oryzae 70-15* | hypothetical protein | Nature 434 (7036), 980-986 (2005) |
| *Fv1157* | EAL00626.1 | *Candida albicans* | potential secreted Cu/Zn superoxide dismutase | Direct Submission |
| *Fv1161* | AAG25917.1 | *Glomerella lindemuthiana* | putative GAL4-like transcriptional activator | Plant Cell 12 (9), 1579-1590 (2000) |
| *Fv1234* | CAB87245.1 | *Candida albicans* | calcium/mangenease P-type ATPase PMR1 | J Biol Chem. 2005 Jun 17;280(24):23408-15. |
| *Fv1328* | EDJ95977.1 | *Magnaporthe grisea* | hypothetical protein | Nature 434 (7036), 980-986 (2005) |
| *Fv1336* | EDJ95969.1 | *Magnaporthe oryzae 70-15* | hypothetical protein | Nature 434 (7036), 980-986 (2005) |
| *Fv1355* | EDK05615.1 | *Magnaporthe oryzae 70-15* | hypothetical protein | Nature 434 (7036), 980-986 (2005) |
| *Fv1365* | CAH04535.1 | *Claviceps purpurea* | putative serine/threonine kinase | Direct Submission |
| *Fv1419* | BAD04045.1 | *Colletotrichum lagenarium* | adenylate cyclase | Mol. Plant Microbe Interact. 17 (12), 1355-1365 (2004) |
| *Fv1437* | EDJ95999.1 | *Magnaporthe oryzae 70-15* | hypothetical protein | Nature 434 (7036), 980-986 (2005) |
| *Fv1462* | BAA02707.1 | *Candida albicans* | chitin synthase III | Direct Submission |
| *Fv1485* | AAP94020.2 | *Ustilago maydis* | B-type cyclin 2 | J. Cell. Sci. 117 (PT 3), 487-506 (2004) |
| *Fv1493* | AAK09366.1 | *Candida albicans* | zinc finger DNA binding protein | EMBO J. 20 (17), 4742-4752 (2001) |
| *Fv1497* | AAG36938.1 | *Cryptococcus neoformans var. neoformans* | Uncharacterized conserved protein, contains HAT | Infect Immun. 2003 Apr;71(4):1988-94. |
| *Fv1526* | AAT85969.1 | *Fusarium oxysporum f. sp. lycopersici* | F-box protein required for pathogenicity | Mol. Microbiol. 57 (4), 1051-1063 (2005) |
| *Fv1545* | AAM22475.1 | *Cryptococcus neoformans var. grubii* | alternative oxidase | Infect. Immun. 71 (10), 5794-5802 (2003) |
| *Fv1698* | ABK64183.1 | *Cercospora nicotianae* | reductase | Mol. Microbiol. 64 (3), 755-770 (2007) |
| *Fv1714* | AAL35099.1 | *Cryptococcus neoformans* | O-acetyltransferase | Mol. Microbiol. 42 (2), 453-467 (2001) |
| *Fv1793* | CAC17748.1 | *Candida albicans* | trehalose-6-phosphate phosphatase | Direct Submission |
| *Fv1796* | EDJ94656.1 | *Magnaporthe grisea* | hypothetical protein | Nature 434 (7036), 980-986 (2005) |
| *Fv1809* | EDJ94565.1 | *Magnaporthe oryzae 70-15* | hypothetical protein | Nature 434 (7036), 980-986 (2005) |
| *Fv1813* | EAL01265.1 | *Candida albicans* | potential glycoprotein glucosyltransferase | Direct Submission |
| *Fv1814* | AAW72937.1 | *Cryptococcus neoformans var. neoformans* | white collar 1 | PLoS Biol. 3 (4), E95 (2005) |
| *Fv1888* | AAG01162.1 | *Fusarium oxysporum* | mitogen-activated protein kinase | Mol. Microbiol. 39 (5), 1140-1152 (2001) |
| *Fv1917* | EAK97884.1 | *Candida albicans SC5314* | potential HSF-type DNA binding transcription | Direct Submission |
| *Fv1957* | EAK93180.1 | *Candida albicans SC5314* | potential zinc finger transcription factor | Proc. Natl. Acad. Sci. U.S.A. 101 (19), 7329-7334 (2004 |
| *Fv1968* | EDK02450.1 | *Magnaporthe oryzae 70-15* | hypothetical protein | Nature 434 (7036), 980-986 (2005) |
| *Fv1991* | AAL54912.2 | *Candida albicans* | putative transcriptional repressor | Direct Submission |
| *Fv2065* | AAK15315.1 | *Candida albicans* | regulator of filamentous growth and virulence Rfg1 | Mol. Cell. Biol. 21 (7), 2496-2505 (2001) |
| *Fv2084* | EDJ98604.1 | *Magnaporthe grisea* | hypothetical protein | Nature 434 (7036), 980-986 (2005) |
| *Fv2093* | AAA77678.1 | *Colletotrichum gloeosporioides* | unknown | Plant Cell 7 (2), 183-193 (1995) |
| *Fv2114* | EAK82380.1 | *Ustilago maydis* | hypothetical protein | Nature 444 (7115), 97-101 (2006) |
| *Fv2120* | AAK27436.1 | *Aspergillus fumigatus* | putative two-component histidine kinase Fos-1 | Direct Submission |
| *Fv2142* | ABB43267.1 | *Botrytis elliptica* | necrosis- and ethylene-inducing protein 2 | Fungal Genet. Biol. 44 (1), 52-63 (2007) |
| *Fv2157* | CAA81090.1 | *Candida albicans* | deduced Candida albicans CDC10 protein | Mol. Gen. Genet. 242 (6), 689-698 (1994) |
| *Fv2158* | EAL88984.1 | *Aspergillus fumigatus* | 1,3-beta-glucanosyltransferase Gel2 | Direct Submission |
| *Fv2174* | AAN46744.1 | *Magnaporthe grisea* | trehalose-6-phosphate synthase subunit 1 | EMBO J. 22 (2), 225-235 (2003) |
| *Fv2234* | AAO54374.1 | *Pseudomonas syringae* | alcohol dehydrogenase, zinc-containing protein | Proc. Natl. Acad. Sci. U.S.A. 100 (18), 10181-10186 (2003) |
| *Fv2353* | BAB40947.1 | *Magnaporthe grisea* | histidine kinase | Fungal Genet Biol. 2005 Mar;42(3):200-12. |
| *Fv2371* | CAA93142.1 | *Botryotinia fuckeliana* | ATP-binding multidrug cassette transporter | Direct Submission |
| *Fv2441* | AAB39507.1 | *Candida albicans* | topoisomerase I | Direct Submission |
| *Fv2446* | ABD59786.1 | *Stagonospora nodorum* | Ca/Cm-dependent protein kinase A | Direct Submission |
| *Fv2471* | EAK82767.1 | *Ustilago maydis* | hypothetical protein | Nature 444 (7115), 97-101 (2006) |
| *Fv2490* | EAL02391.1 | *Candida albicans SC5314* | hypothetical protein | Direct Submission |
| *Fv2538* | CAO82106.1 | *Claviceps purpurea* | PAK kinase | Mol. Microbiol. 68 (2), 405-423 (2008) |
| *Fv2561* | AAC05307.1 | *Candida albicans* | protein-tyrosine phosphatase | Mol. Biol. Cell 8 (12), 2539-2551 (1997) |
| *Fv2579* | EDK03302.1 | *Magnaporthe oryzae 70-15* | hypothetical protein | Nature 434 (7036), 980-986 (2005) |
| *Fv2587* | ACB38886.1 | *Cercospora zeae-maydis* | putative 6-4 photolyase | Fungal Genet Biol. 2008 Oct;45(10):1364-72. |
| *Fv2611* | EDJ95219.1 | *Magnaporthe oryzae 70-15* | hypothetical protein | Nature 434 (7036), 980-986 (2005) |
| *Fv2624* | CAA04716.1 | *Claviceps purpurea* | catalase | Direct Submission |
| *Fv2631* | AAA96019.1 | *Candida albicans* | integrin-like protein alpha Int1p | Proc Natl Acad Sci U S A. 1996 Jan 9;93(1):357-61. |
| *Fv2633* | BAC11803.1 | *Colletotrichum lagenarium* | Ste12-like transcription factor | Mol. Plant Microbe Interact. 16 (4), 315-325 (2003) |
| *Fv2658* | BAC76819.1 | *Cryptococcus neoformans* | capsule protein | Direct Submission |
| *Fv2683* | AAK31936.1 | *Cryptococcus neoformans* | pheromone receptor CPRa1p | Infect. Immun. 71 (9), 4953-4960 (2003) |
| *Fv2687* | AAZ95011.1 | *Phaeosphaeria nodorum* | delta-aminolevulinic acid synthase | Microbiology (Reading, Engl.) 152 (PT 5), 1533-1538 (2006) |
| *Fv2701* | BAD04044.1 | *Colletotrichum lagenarium* | catalytic subunit of cAMP-dependent protein | Direct Submission |
| *Fv2767* | AAM95700.1 | *Fusarium oxysporum* | pH transcription factor | Mol. Microbiol. 48 (3), 765-779 (2003) |
| *Fv2771* | EDJ95433.1 | *Magnaporthe grisea* | hypothetical protein | Nature 434 (7036), 980-986 (2005) |
| *Fv2806* | AAL84247.1 | *Pseudomonas syringae* | type III effector HopI1 | Science 295 (5560), 1722-1726 (2002) |
| *Fv2813* | CAA42366.2 | *Saccharomyces cerevisiae* | beta-isopropyl-malate dehydrogenase | Yeast 7 (5), 533-538 (1991) |
| *Fv2861* | EAL01707.1 | *Candida albicans* | hypothetical protein | Direct Submission |
| *Fv2863* | AAP12366.1 | *Gibberella zeae* | acyl CoA ligase-like protein | Proc. Natl. Acad. Sci. U.S.A. 100 (10), 5980-5985 (2003) |
| *Fv2899* | EAK81357.1 | *Ustilago maydis* | hypothetical protein | Nature 444 (7115), 97-101 (2006) |
| *Fv2928* | EDK02952.1 | *Magnaporthe grisea* | hypothetical protein | Nature 434 (7036), 980-986 (2005) |
| *Fv2929* | EDK00897.1 | *Magnaporthe oryzae 70-15* | hypothetical protein | Nature 434 (7036), 980-986 (2005) |
| *Fv2981* | AAN03477.1 | *Cryptococcus neoformans var. neoformans* | prolyl isomerase Ess1 | Microbiology (Reading, Engl.) 151 (PT 5), 1593-1605 (2005) |
| *Fv3078* | AAA34824.1 | *Saccharomyces cerevisiae* | orotidine-5'-phosphate decarboxylase monomer | Gene. 1984 Jul-Aug;29(1-2):113-24. |
| *Fv3099* | EAL92371.2 | *Aspergillus fumigatus Af293* | fatty acid oxygenase PpoC | Direct Submission |
| *Fv3222* | AAO19638.1 | *Ustilago maydis* | guanyl nucleotide exchange factor Sql2 | Eukaryotic Cell 2 (3), 609-617 (2003) |
| *Fv3288* | XP_391001.1 | *Fusarium graminearum* | hypothetical protein | Direct Submission |
| *Fv3311* | BAE72682.1 | *Epichloe festucae* | NADPH oxidase | Plant Cell. 2006 Apr;18(4):1052-66. |
| *Fv3327* | AAV41010.1 | *Cryptococcus neoformans* | virulence associated DEAD box protein 1 | J. Clin. Invest. 115 (3), 632-641 (2005) |
| *Fv3334* | AAM81358.1 | *Leptosphaeria maculans* | aspartyl proteinase | Physiol. Mol. Plant Pathol. 62, 305-313 (2003) |
| *Fv3401* | BAB40769.1 | *Fusarium oxysporum* | argininosuccinate lyase | Direct Submission |
| *Fv3547* | AAB65427.1 | *Magnaporthe grisea* | G alpha subunit | Mol. Plant Microbe Interact. 10 (9), 1075-1086 (1997) |
| *Fv3567* | AAP41027.1 | *Cryptococcus neoformans* | GSNO reductase | Curr. Biol. 13 (22), 1963-1968 (2003) |
| *Fv3582* | EDJ97928.1 | *Magnaporthe grisea* | hypothetical protein | Nature 434 (7036), 980-986 (2005) |
| *Fv3607* | AAF00024.1 | *Colletotrichum gloeosporioides* | unknown | J. Bacteriol. 182 (17), 4688-4695 (2000) |
| *Fv3641* | EDK03989.1 | *Magnaporthe oryzae 70-15* | hypothetical protein | Nature 434 (7036), 980-986 (2005) |
| *Fv3672* | EAK93902.1 | *Candida albicans* | hypothetical protein | Proc. Natl. Acad. Sci. U.S.A. 101 (19), 7329-7334 (2004) |
| *Fv3683* | AAK31209.1 | *Colletotrichum lagenarium* | cAMP-dependent protein kinase regulatory | Mol. Plant Microbe Interact. 14 (10), 1149-1157 (2001) |
| *Fv3687* | AAD55385.1 | *Glomerella cingulata* | MAP kinase kinase | Plant Cell 12 (8), 1331-1343 (2000) |
| *Fv3696* | AAN32715.1 | *Fusarium oxysporum* | protein kinase SNF1 | Curr. Genet. 44 (1), 49-57 (2003) |
| *Fv3697* | EAK93039.1 | *Candida albicans* | hypothetical protein | Proc. Natl. Acad. Sci. U.S.A. 101 (19), 7329-7334 (2004) |
| *Fv3698* | EDJ95622.1 | *Magnaporthe oryzae 70-15* | neutral trehalase | Nature 434 (7036), 980-986 (2005) |
| *Fv3713* | BAA24951.1 | *Candida albicans* | Histidine Kinase A | Microbiology (Reading, Engl.) 144 (PT 2), 425-432 (1998) |
| *Fv3800* | CAD21519.1 | *Claviceps purpurea* | putative bZip transcription factor | Direct Submission |
| *Fv3815* | EDK02656.1 | *Magnaporthe oryzae 70-15* | hypothetical protein | Nature 434 (7036), 980-986 (2005) |
| *Fv3830* | AAL23718.1 | *Glomerella graminicola* | chitin synthase B | Direct Submission |
| *Fv3832* | AAO19639.1 | *Ustilago maydis* | small G-protein Ras2 | Eukaryotic Cell 2 (3), 609-617 (2003) |
| *Fv3913* | AAM33130.1 | *Leptosphaeria maculans* | secreted protein 1 | Mol. Plant Pathol. 3 (6), 487-493 (2002) |
| *Fv3965* | CAF05793.1 | *Ustilago maydis* | alpha-glucosidase II precursor | Plant Cell 17 (12), 3532-3543 (2005) |
| *Fv3980* | AAD31929.1 | *Aspergillus fumigatus* | para aminobenzoic acid synthetase | Mol. Microbiol. 36 (6), 1371-1380 (2000) |
| *Fv4087* | AAB97372.1 | *Cryptococcus neoformans var. grubii* | calcineurin A catalytic subunit | EMBO J. 16 (10), 2576-2589 (1997) |
| *Fv4097* | AAB88888.1 | *Magnaporthe grisea* | imidazole glycerol phosphate dehydratase | Direct Submission |
| *Fv4117* | BAE66713.1 | *Colletotrichum lagenarium* | hypothetical protein | Mol. Microbiol. 64 (5), 1332-1349 (2007) |
| *Fv4220* | EDJ94907.1 | *Magnaporthe oryzae 70-15* | hypothetical protein | Nature 434 (7036), 980-986 (2005) |
| *Fv4231* | CAA80454.1 | *Magnaporthe grisea* | C-8 sterol isomerase | Curr. Genet. 25 (6), 531-537 (1994) |
| *Fv4302* | AAS48112.1 | *Cryptococcus neoformans* | lysine and spermidine biosynthesis | Eukaryotic Cell 3 (3), 752-763 (2004) |
| *Fv4316* | EDK03750.1 | *Magnaporthe grisea* | hypothetical protein | Nature 434 (7036), 980-986 (2005) |
| *Fv4383* | AAG13937.1 | *Filobasidiella neoformans* | calcineurin B regulatory subunit | Mol. Microbiol. 39 (4), 835-849 (2001) |
| *Fv4388* | AAD25159.1 | *Candida albicans* | CDC2-related protein kinase CRK1 | Mol. Cell. Biol. 20 (23), 8696-8708 (2000) |
| *Fv4457* | AAN17787.1 | *Aspergillus fumigatus* | Ras homolog enriched in brain | Fungal Genet. Biol. 36 (3), 207-214 (2002) |
| *Fv4529* | AAF82788.1 | *Passalora fulva* | alcohol oxidase | Mol Plant Microbe Interact. 1997 Dec;10(9):1106-9. |
| *Fv4703* | CAB52402.1 | *Botryotinia fuckeliana* | ABC transporter | Mol. Plant Microbe Interact. 14 (4), 562-571 (2001) |
| *Fv4755* | AAN65464.1 | *Glomerella lindemuthiana* | major nitrogen regulatory protein | Mol. Microbiol. 48 (3), 639-655 (2003) |
| *Fv4772* | EDK03349.1 | *Magnaporthe grisea* | hypothetical protein | Nature 434 (7036), 980-986 (2005) |
| *Fv4906* | CAC50073.1 | *Claviceps purpurea* | Cu/Zn-superoxide dismutase | Mol. Plant Pathol. 3 (1), 9-22 (2002) |
| *Fv4907* | AAS91580.1 | *Phaeosphaeria nodorum* | malate synthase | Direct Submission |
| *Fv4916* | CAD43407.1 | *Glomerella lindemuthiana* | tetraspanin | Biochem. Biophys. Res. Commun. 297 (5), 1197-1204 (2002) |
| *Fv4917* | AAC98913.1 | *Candida albicans* | D-arabinono-1,4-lactone oxidase | Direct Submission |
| *Fv4992* | CAA70254.1 | *Candida albicans* | phosphatidylinositol 3-kinase | Yeast 16 (10), 933-944 (2000) |
| *Fv4998* | AAW45289.1 | *Cryptococcus neoformans var. neoformans JEC21* | Rho guanyl-nucleotide exchange factor | Science 307 (5713), 1321-1324 (2005) |
| *Fv5016* | AAK98783.1 | *Magnaporthe grisea* | putative vacuolar ATPase MVP1 | FEMS Microbiol Lett. 2004 Jan 15;230(1):85-90. |
| *Fv5021* | ABK64180.1 | *Cercospora nicotianae* | O-methyltransferase | Mol. Microbiol. 64 (3), 755-770 (2007) |
| *Fv5022* | AAS57292.1 | *Gibberella zeae* | polyketide synthase | Proc. Natl. Acad. Sci. U.S.A. 100 (26), 15670-15675 (2003) |
| *Fv5063* | AAB64668.1 | *Saccharomyces cerevisiae* | Cox15p: Cytochrom oxidase assembly factor | Nature 387 (6632 Suppl), 78-81 (1997) |
| *Fv5083* | AAS64313.1 | *Cochliobolus heterostrophus* |  | Eukaryotic Cell 4 (2), 443-454 (2005) |
| *Fv5108* | BAB43813.1 | *Candida albicans* | CaNAG2 | Eur. J. Biochem. 268 (8), 2498-2505 (2001) |
| *Fv5109* | BAB43821.1 | *Candida albicans* | CaNAG1 | Direct Submission |
| *Fv5110* | EAK93243.1 | *Candida albicans SC5314* | N-acetylglucosamine kinase | Proc. Natl. Acad. Sci. U.S.A. 101 (19), 7329-7334 (2004) |
| *Fv5193* | EAL19243.1 | *Cryptococcus neoformans var. neoformans B-3501A* | hypothetical protein | Direct Submission |
| *Fv5217* | EDJ98555.1 | *Magnaporthe grisea* | hypothetical protein | Nature 434 (7036), 980-986 (2005) |
| *Fv5341* | AAB86640.1 | *Magnaporthe grisea* | ABC1 transporter | EMBO J. 18 (3), 512-521 (1999) |
| *Fv5390* | AAK00131.1 | *Magnaporthe grisea* | Deuterolysin metalloprotease (M35) family | Plant Cell 12 (11), 2019-2032 (2000) |
| *Fv5403* | ABB55459.1 | *Leptosphaeria maculans* | probable 3-ketoacyl-CoA thiolase | Mol. Plant Microbe Interact. 19 (6), 588-596 (2006) |
| *Fv5433* | AAF69680.1 | *Candida albicans* | high-affinity iron permease CaFTR1 | Science 288 (5468), 1062-1064 (2000) |
| *Fv5509* | AAC61890.1 | *Candida albicans* | phospholipase B precursor | J. Biol. Chem. 273 (40), 26078-26086 (1998) |
| *Fv5591* | ABP98949.1 | *Magnaporthe grisea* | Cyt_b561_FRRS1_like | Direct Submission |
| *Fv5597* | AAA02743.1 | *Saccharomyces cerevisiae* | hsp82 protein | J. Biol. Chem. 259 (9), 5745-5751 (1984) |
| *Fv5600* | AAX78216.1 | *Fusarium oxysporum* | beta-1,3-glucanosyltransferase" | The following term was not found in Protein: AAX7821. |
| *Fv5618* | EAK95175.1 | *Candida albicans SC5314* | mannosylation of inositolphosphorylceramid | Proc. Natl. Acad. Sci. U.S.A. 101 (19), 7329-7334 (2004) |
| *Fv5678* | XP_759763.1 | *Ustilago maydis* | hypothetical protein | Direct Submission |
| *Fv5748* | AAN10186.1 | *Ustilago maydis* | cell cycle regulatory protein | Direct Submission |
| *Fv5767* | AAD42978.1 | *Candida albicans* | adenylate cyclase-associated protein homolog | J. Bacteriol. 183 (10), 3211-3223 (2001) |
| *Fv5768* | AAB88887.2 | *Magnaporthe grisea* | carnitine acetyl transferase | Direct Submission |
| *Fv5778* | XP_382108.1 | *Fusarium graminearum* | hypothetical protein | Direct Submission |
| *Fv5779* | AAX07670.1 | *Magnaporthe grisea* | MSP1 protein-like protein | Direct Submission |
| *Fv5782* | ABR12478.1 | *Fusarium oxysporum* | Ctf1 | Mol. Plant Pathol. 9 (3), 293-304 (2008) |
| *Fv5809* | AAB84285.1 | *Ustilago maydis* | class V chitin synthase | Fungal Genet. Biol. 22 (3), 199-208 (1997) |
| *Fv5810* | AAO49384.1 | *Fusarium oxysporum* | class V chitin synthase | Direct Submission |
| *Fv5838* | ABN41464.1 | *Fusarium oxysporum* | XlnR-transcription factor | Mol Plant Microbe Interact. 2007 Aug;20(8):977-85. |
| *Fv5842* | AAC62818.1 | *Cochliobolus carbonum* | putative fatty acid synthase beta | Mol. Plant Microbe Interact. 10 (2), 207-214 (1997) |
| *Fv5843* | AAA34345.1 | *Candida albicans* | fatty acid synthase alpha subunit | Direct Submission |
| *Fv5846* | CAA37820.1 | *Candida albicans* | benomyl/methotrexate resistance | Direct Submission |
| *Fv5848* | CAM56211.1 | *Claviceps purpurea* | ku70 protein" | Fungal Genet Biol. 2008 Jan;45(1):35-44. |
| *Fv5856* | BAE98264.2 | *Fusarium oxysporum* | Zn(II)2Cys6 transcription factor | Mol. Microbiol. 63 (3), 737-753 (2007) |
| *Fv5864* | AAO73005.1 | *Cryptococcus neoformans var. grubii* | voltage-gated chloride channel | Mol. Microbiol. 50 (4), 1271-1281 (2003) |
| *Fv5904* | EDJ98842.1 | *Magnaporthe oryzae 70-15* | hypothetical protein | Nature 434 (7036), 980-986 (2005) |
| *Fv5953* | AAC13946.1 | *Filobasidiella neoformans* | unknown | Mol. Cell. Biol. 14 (7), 4912-4919 (1994) |
| *Fv5995* | AAB80929.1 | *Candida albicans* | proteinase | J. Bacteriol. 177 (18), 5215-5221 (1995) |
| *Fv6059* | ABC79591.2 | *Cercospora nicotianae* | cercosporin toxin biosynthesis protein | Fungal Genet. Biol. 44 (5), 444-454 (2007) |
| *Fv6060* | AAC39471.1 | *Aspergillus fumigatus* | polyketide synthase | J. Bacteriol. 180 (12), 3031-3038 (1998) |
| *Fv6075* | XP_389373.1 | *Fusarium graminearum* | hypothetical protein | Direct Submission |
| *Fv6088* | EDJ96020.1 | *Magnaporthe oryzae 70-15* | zinc-binding dehydrogenase | Nature 434 (7036), 980-986 (2005) |
| *Fv6253* | EDJ95941.1 | *Magnaporthe grisea* | hypothetical protein | Nature 434 (7036), 980-986 (2005) |
| *Fv6280* | EAK94586.1 | *Candida albicans SC5314* | hypothetical protein" | Proc. Natl. Acad. Sci. U.S.A. 101 (19), 7329-7334 (2004) |
| *Fv6312* | AAB63195.1 | *Candida albicans* | transcriptional repressor TUP1 | Science 277 (5322), 105-109 (1997) |
| *Fv6321* | AAL83897.1 | *Candida albicans* | putative Gpi7p | Direct Submission |
| *Fv6371* | EAK93583.1 | *Candida albicans* | hypothetical protein | Proc. Natl. Acad. Sci. U.S.A. 101 (19), 7329-7334 (2004) |
| *Fv6421* | CAA64457.2 | *Candida albicans* | ATP-dependent DNA ligase | Yeast 12 (9), 893-898 (1996) |
| *Fv6439* | AAB69694.1 | *Magnaporthe grisea* | putative transcriptional regulator | Direct Submission |
| *Fv6449* | BAF36501.1 | *Epichloe festucae* | NADPH oxidase regulator NoxR | Plant Cell. 2006 Oct;18(10):2807-21. |
| *Fv6468* | AAO91808.1 | *Fusarium oxysporum f. sp. lycopersici* | G protein beta subunit | Fungal Genet. Biol. 42 (1), 61-72 (2005) |
| *Fv6473* | ACS34772.1 | *Mycosphaerella graminicola* | scaffold protein | Direct Submission |
| *Fv6498* | AAG09789.1 | *Candida albicans* | repressed by TUP1 protein 4 | Genetics 155 (1), 57-67 (2000) |
| *Fv6528* | AAT77184.1 | *Fusarium oxysporum f. sp. lycopersici* | chitin synthase chaperone-like protein Chs7 | Microbiology (Reading, Engl.) 150 (PT 10), 3175-3187 (2004) |
| *Fv6653* | CAA56936.1 | *Rhynchosporium secalis* | beta-tubulin | Pestic. Sci. 43, 201-209 (1995) |
| *Fv6694* | AAP93639.1 | *Magnaporthe grisea* | PAK kinase | Mol. Plant Microbe Interact. 17 (5), 547-556 (2004) |
| *Fv6706* | AAT73077.1 | *Stagonospora nodorum* | glyoxylase I | Curr. Genet. 46 (2), 115-121 (2004) |
| *Fv6773* | AAO72959.1 | *Candida albicans* | agglutinin-like sequence 3 | Direct Submission |
| *Fv6794* | AAM51626.1 | *Candida albicans* | septin | Mol Biol Cell. 2002 Aug;13(8):2732-46. |
| *Fv6847* | AAK77953.1 | *Botrytis cinerea* | p-diphenol:oxygen oxidoreductase | Direct Submission |
| *Fv6871* | BAD44729.1 | *Fusarium oxysporum* | G-protein alpha subunit | FEMS Microbiol. Lett. 243 (1), 165-172 (2005) |
| *Fv6874* | XP_389788.1 | *Fusarium graminearum* | hypothetical protein | Direct Submission |
| *Fv6904* | EAL02110.1 | *Candida albicans SC5314* | hypothetical protein | Direct Submission |
| *Fv6940* | EDK04531.1 | *Magnaporthe oryzae 70-15* | hypothetical protein | Nature 434 (7036), 980-986 (2005) |
| *Fv7007* | BAC98434.1 | *Burkholderia glumae* | GyrA protein | Appl. Environ. Microbiol. 70 (9), 5613-5620 (2004) |
| *Fv7038* | AAD55937.1 | *Cryptococcus neoformans var. grubii* | GTPase SAR1 and related small G proteins | Mol. Microbiol. 36 (2), 352-365 (2000) |
| *Fv7051* | EDJ99464.1 | *Magnaporthe oryzae 70-15* | hypothetical protein | Nature 434 (7036), 980-986 (2005) |
| *Fv7124* | ABF69301.1 | *Cryptococcus neoformans* | transcriptional regulator Nrg1 | Eukaryotic Cell 5 (7), 1147-1156 (2006) |
| *Fv7177* | AAB63337.1 | *Ustilago maydis* | kinesin motor protein | EMBO J. 16 (12), 3464-3473 (1997) |
| *Fv7180* | AAD33888.1 | *Candida albicans* | transcription factor Spt3 | Genetics 161 (2), 509-519 (2002) |
| *Fv7197* | EDK04805.1 | *Magnaporthe grisea* | Direct Submission | Nature 434 (7036), 980-986 (2005) |
| *Fv7204* | AAL20306.1 | *Salmonella enterica* | putative regulatory protein, deoR family | Nature 413 (6858), 852-856 (2001) |
| *Fv7225* | AAC62627.1 | *Ajellomyces capsulatus* | orotidine-5'-monophosphate pyrophosphorylase | J. Bacteriol. 180 (19), 5135-5143 (1998) |
| *Fv7272* | CAC41973.1 | *Colletotrichum lindemuthianum* | putative Rab/GTPase | Gene 272 (1-2), 219-225 (2001) |
| *Fv7278* | AAQ18216.1 | *Trichophyton rubrum* | squalene epoxidase | Direct Submission |
| *Fv7292* | AAT92283.1 | *Cryphonectria parasitica* | regulator of G-protein signaling | Eukaryotic Cell 3 (6), 1454-1463 (2004) |
| *Fv7346* | EDK04578.1 | *Magnaporthe oryzae 70-15* | hypothetical protein |  |
| *Fv7361* | AAF74764.1 | *Magnaporthe grisea* | MAS1 protein | Direct Submission |
| *Fv7383* | CAA67930.1 | *Candida albicans* | putative mannosyl transferase | Direct Submission |
| *Fv7415* | ABA25866.1 | *Botryotinia fuckeliana* | putative FKBP12 protein | J. Mol. Biol. 358 (2), 372-386 (2006) |
| *Fv7423* | EAK93097.1 | *Candida albicans* | potential polyamine transporter | Proc. Natl. Acad. Sci. U.S.A. 101 (19), 7329-7334 (2004) |
| *Fv7465* | CAC48042.1 | *Aspergillus fumigatus* | homoaconitase | Arch. Microbiol. 181 (5), 378-383 (2004) |
| *Fv7531* | AAD43562.2 | *Gibberella moniliformis* | Fum1p | Fungal Genet. Biol. 27 (1), 100-112 (1999) |
| *Fv7592* | AAZ30050.1 | *Cryptococcus neoformans var. grubii* | carbonic anhydrase 1 | Curr. Biol. 15 (22), 2013-2020 (2005) |
| *Fv7621* | CAC40023.1 | *Gibberella pulicaris* | ABC-transporter | Mol. Plant Microbe Interact. 15 (2), 102-108 (2002) |
| *Fv7642* | AAX14039.2 | *Glomerella acutata* | key lime pathogenicity protein | Mol. Plant Pathol. 6 (5), 513-525 (2005) |
| *Fv7644* | AAS19620.1 | *Cryptococcus bacillisporus* | manganese superoxide dismutase | Mol. Microbiol. 55 (6), 1782-1800 (2005) |
| *Fv7648* | EAK91807.1 | *Candida albicans* | likely flavohemoglobin | Proc. Natl. Acad. Sci. U.S.A. 101 (19), 7329-7334 (2004) |
| *Fv7668* | AAG10203.1 | *Cryptococcus neoformans var. neoformans* | mannose-6-phosphate isomerase | Mol. Microbiol. 40 (3), 610-620 (2001) |
| *Fv7738* | AAM21640.3 | *Ustilago maydis* | cdk-related kinase 1 | Mol. Microbiol. 47 (3), 729-743 (2003) |
| *Fv7775* | BAA24262.1 | *Candida albicans* | RhoA_like | J Bacteriol. 1997 Dec;179(24):7734-41. |
| *Fv7916* | AAX09988.1 | *Cochliobolus heterostrophus* | nonribosomal peptide synthetase 6 | Eukaryotic Cell 4 (3), 545-555 (2005) |
| *Fv8026* | BAB85760.1 | *Fusarium oxysporum* | putative mitochondrial carrier protein | Direct Submission |
| *Fv8077* | EAK91146.1 | *Candida albicans* | potential type 2A-related protein phosphatase | Proc. Natl. Acad. Sci. U.S.A. 101 (19), 7329-7334 (2004) |
| *Fv8105* | AAO68047.1 | *Salmonella enterica subsp. enterica serovar Typhi str. Ty2* | putative thiosulfate sulfurtransferase | J. Bacteriol. 185 (7), 2330-2337 (2003) |
| *Fv8111* | AAN62846.1 | *Glomerella lindemuthiana* | CLAP1" | Mol Genet Genomics. 2002 Oct;268(2):139-51. |
| *Fv8160* | EAL01044.1 | *Candida albicans SC5314* | hypothetical protein | Direct Submission |
| *Fv8181* | XP_381731.1 | *Fusarium graminearum* | hypothetical protein | Direct Submission |
| *Fv8200* | CAA93255.1 | *Botryotinia fuckeliana* | cutinase | Mol Plant Microbe Interact. 1997 Jan;10(1):21-9. |
| *Fv8250* | AAU87359.1 | *Botrytis cinerea* | lipase | Direct Submission |
| *Fv8260* | EDK00201.1 | *Magnaporthe grisea* | hypothetical protein | Nature 434 (7036), 980-986 (2005) |
| *Fv8266* | AAX55652.1 | *Gibberella moniliformis* | stalk rot protein | Direct Submission |
| *Fv8325* | EDJ98620.1 | *Magnaporthe grisea* | HDEL sequence binding protein, putative | Nature 434 (7036), 980-986 (2005) |
| *Fv8328* | AAT40313.1 | *Botryotinia fuckeliana* | endo-beta-1,4-glucanase precursor" | Physiol. Mol. Plant Pathol. 66 (6), 213-221 (2005) |
| *Fv8414* | ABB90284.1 | *Gibberella zeae* | FAD/FMN-containing dehydrogenases | Mol. Microbiol. 58 (4), 1102-1113 (2005) |
| *Fv8468* | AAP93915.1 | *Candida dubliniensis* | cell surface hydrophobicity-associated protein | J. Bacteriol. 183 (12), 3582-3588 (2001) |
| *Fv8469* | AAF01762.1 | *Alternaria alternata* | AM-toxin synthetase | Mol. Plant Microbe Interact. 13 (7), 742-753 (2000) |
| *Fv8565* | AAM88291.1 | *Cochliobolus heterostrophus* | decarboxylase DEC1 | Mol. Plant Microbe Interact. 15 (9), 883-893 (2002) |
| *Fv8721* | AAO63562.1 | *Verticillium fungicola* | beta-1,6-glucanase | Direct Submission |
| *Fv8731* | EDK06580.1 | *Magnaporthe oryzae 70-15* | hypothetical protein | Nature 434 (7036), 980-986 (2005) |
| *Fv8771* | AAK15314.1 | *Mycosphaerella graminicola* | ABC transporter Atr4 | Gene 289 (1-2), 141-149 (2002) |
| *Fv8860* | BAC55015.1 | *Fusarium oxysporum* | MedA homolog | Genetics 166 (1), 113-124 (2004) |
| *Fv8865* | EDJ99440.1 | *Magnaporthe oryzae 70-15* | hypothetical protein | Nature 434 (7036), 980-986 (2005) |
| *Fv8873* | AAT77182.1 | *Fusarium oxysporum f. sp. lycopersici* | chitin synthase class II | Microbiology (Reading, Engl.) 150 (PT 10), 3175-3187 (2004) |
| *Fv8938* | EDK03444.1 | *Magnaporthe grisea* | hypothetical protein | Nature 434 (7036), 980-986 (2005) |
| *Fv8976* | AAK95561.1 | *Cryptococcus neoformans* | UDP-glucose dehydrogenase Ugd1p | Biochem. J. 381 (PT 1), 131-136 (2004) |
| *Fv9012* | EDJ99431.1 | *Magnaporthe oryzae 70-15* | hypothetical protein | Nature 434 (7036), 980-986 (2005) |
| *Fv9112* | AAR29084.1 | *Cryptococcus neoformans var. grubii* | acetolactate synthase | Microbiology (Reading, Engl.) 150 (PT 5), 1547-1558 (2004) |
| *Fv9210* | AAK19621.1 | *Cochliobolus carbonum* | cellulase | Direct Submission |
| *Fv9290* | EAK98810.1 | *Candida albicans SC5314* | potential forkhead-like transcriptional | GenBank: EAK98810.1 |
| *Fv9305* | CAI48090.1 | *Claviceps purpurea* | putative Cdc42-like GTP-binding protein | Direct Submission |
| *Fv9330* | AAM21050.1 | *Candida albicans* | secreted aspartic proteinase 2 | Mol. Microbiol. 44 (5), 1351-1366 (2002) |
| *Fv9343* | ACS91346.1 | *Mycosphaerella graminicola* | mitogen-activated protein kinase kinase kinase | Direct Submission |
| *Fv9378* | AAA17543.1 | *Saccharomyces cerevisiae* | mitochondrial DNA polymerase" | J Biol Chem. 1989 Dec 5;264(34):20552-60. |
| *Fv9399* | BAB69488.1 | *Fusarium oxysporum* | guanine nucleotide-binding protein alpha | Curr. Genet. 41 (6), 407-413 (2002) |
| *Fv9412* | AAB61403.1 | *Colletotrichum lindemuthianum* | putative serine/threonine kinase | Direct Submission |
| *Fv9438* | AAD51594.2 | *Candida albicans* | MRP-like transporter | Mol. Microbiol. 43 (3), 571-584 (2002) |
| *Fv9469* | AAK16738.1 | *Colletotrichum lagenarium* | unknown | Plant Cell 13 (8), 1945-1957 (2001) |
| *Fv9489* | AAB97419.1 | *Mycosphaerella graminicola* | succinate dehydrogenase iron-sulphur protein | Curr. Genet. 34 (5), 393-398 (1998) |
| *Fv9507* | AAD47837.1 | *Cochliobolus carbonum* | alanine racemase | J. Biol. Chem. 275 (7), 4906-4911 (2000) |
| *Fv9526* | EDJ99418.1 | *Magnaporthe grisea* | hypothetical protein | Nature 434 (7036), 980-986 (2005) |
| *Fv9543* | XP_385834.1 | *Fusarium graminearum* | hypothetical protein | Direct Submission |
| *Fv9625* | BAA36589.1 | *Alternaria alternata* | Akt2 | Direct Submission |
| *Fv9766* | AAO31597.1 | *Phaeosphaeria nodorum* | di/tri peptide transporter 2 | Physiol. Mol. Plant Pathol. 63 (4), 191-199 (2004) |
| *Fv9875* | ABK64182.1 | *Cercospora nicotianae* | oxidoreductase | Mol. Microbiol. 64 (3), 755-770 (2007) |
| *Fv9999* | AAL23530.1 | *Salmonella enterica subsp. enterica serovar Typhimurium str. LT2* | Salmonella plasmid virulence | Nature 413 (6858), 852-856 (2001) |
| *Fv10257* | XP_759762.1 | *Ustilago maydis* | hypothetical protein | Direct Submission |
| *Fv10359* | AAD00894.3 | *Glomerella cingulata* | CHIP6 | Plant J. 30 (2), 177-187 (2002) |
| *Fv10378* | AAD55813.1 | *Candida albicans* | ssk1p | Yeast 15 (12), 1243-1254 (1999) |
| *Fv10386* | BAA36496.1 | *Candida albicans* | acetyltransferase | J Biol Chem. 1999 Jan 1;274(1):424-9. |
| *Fv10400* | AAC31119.1 | *Candida albicans* | protein mannosyltransferase 1 | Direct Submission |
| *Fv10453* | EDK01543.1 | *Magnaporthe oryzae 70-15* | hypothetical protein | Nature 434 (7036), 980-986 (2005) |
| *Fv10479* | AAL92174.1 | *Cryptococcus neoformans var. grubii* | sulfate adenyltransferase MET3 | Microbiology (Reading, Engl.) 148 (PT 8), 2617-2625 (2002) |
| *Fv10508* | CAO82105.1 | *Claviceps purpurea* | Rho-GTPase | Mol. Microbiol. 68 (2), 405-423 (2008) |
| *Fv10549* | AAD09857.1 | *Glomerella cingulata* | pectate lyase B | Mol. Plant Microbe Interact. 13 (8), 887-891 (2000) |
| *Fv10554* | AAC16255.1 | *Cryphonectria parasitica* | cross-pathway control protein 1 | Direct Submission |
| *Fv10569* | AAW46354.1 | *Cryptococcus neoformans* | protein kinase kin1, putative | Science 307 (5713), 1321-1324 (2005) |
| *Fv10614* | AAF66693.2 | *Candida albicans* | NOT4p | Med. Mycol. 39 (1), 81-86 (2001) |
| *Fv10704* | AAA33024.1 | *Cochliobolus carbonum* | beta-1,4-D-xylanase | Appl Environ Microbiol. 1996 Nov;62(11):4129-35. |
| *Fv11180* | AAM13670.1 | *Gibberella zeae* | MAP kinase | Mol. Plant Microbe Interact. 15 (11), 1119-1127 (2002) |
| *Fv11197* | AAL23717.1 | *Glomerella graminicola* | chitin synthase A | Direct Submission |
| *Fv11229* | EAK98681.1 | *Candida albicans SC5314* | potential mRNA deadenylase and CCR4-NOT complex | GenBank: EAK98681.1 |
| *Fv11424* | AAX55972.1 | *Cryptococcus neoformans var. grubii* | glucosylceramide synthase | J. Clin. Invest. 116 (6), 1651-1659 (2006) |
| *Fv11426* | EDJ97946.1 | *Magnaporthe grisea* | hypothetical protein | Nature 434 (7036), 980-986 (2005) |
| *Fv11430* | AAB09777.1 | *Gaeumannomyces graminis* | avenacinase | Mol. Plant Microbe Interact. 8 (6), 971-978 (1995) |
| *Fv11471* | AAB39564.1 | *Botryotinia fuckeliana* | phosphoinositide-specific phospholipase C | Mol. Cells 7 (2), 192-199 (1997) |
| *Fv11474* | EDJ98595.1 | *Magnaporthe grisea* | hypothetical protein | Nature 434 (7036), 980-986 (2005) |
| *Fv11476* | EDJ98115.1 | *Magnaporthe oryzae 70-15* | hypothetical protein | Nature 434 (7036), 980-986 (2005) |
| *Fv11479* | CAB56523.1 | *Stagonospora nodorum* | ornithine decarboxylase | Direct Submission |
| *Fv11481* | EDJ98043.1 | *Magnaporthe grisea* | hypothetical protein | Nature 434 (7036), 980-986 (2005) |
| *Fv11482* | AAQ23181.1 | *Fusarium graminearum* | extracellular lipase | Plant J. 42 (3), 364-375 (2005) |
| *Fv11511* | EAK83313.1 | *Ustilago maydis* | hypothetical protein | Direct Submission |
| *Fv11518* | AAG02418.1 | *Ustilago maydis* | regulator Ustilago maydis 1 protein | Mol. Microbiol. 38 (1), 154-166 (2000) |
| *Fv11556* | EAK97011.1 | *Candida albicans* | ferrichrome-type siderophore transporter | Direct Submission |
| *Fv11590* | AAD30436.1 | *Magnaporthe grisea* | integral membrane protein | Plant Cell 11 (10), 2013-2030 (1999) |
| *Fv11592* | AAK52794.1 | *Magnaporthe grisea* | expressed during appressorium formation | Direct Submission |
| *Fv11605* | EAK82769.1 | *Ustilago maydis* | hypothetical protein | Nature 444 (7115), 97-101 (2006) |
| *Fv11607* | AAA33353.1 | *Gloeocercospora sorghi* | cyanide hydratase | Biochem. Biophys. Res. Commun. 187 (2), 1048-1054 (1992) |
| *Fv11624* | AAW46720.1 | *Cryptococcus neoformans* | Thioredoxin_like | Science 307 (5713), 1321-1324 (2005) |
| *Fv11691* | AAM22494.1 | *Cryptococcus neoformans var. neoformans* | UDP-xylose synthase | Mol. Microbiol. 45 (3), 837-849 (2002) |
| *Fv11732* | AAC06239.1 | *Fusarium oxysporum* | Fungal cellulose binding domain; cl02521 | Mol Gen Genet. 1999 Apr;261(3):530-6. |
| *Fv11810* | AAB08104.3 | *Cochliobolus heterostrophus* | polyketide synthase | Plant Cell 8 (11), 2139-2150 (1996) |
| *Fv11956* | AAF64435.2 | *Botryotinia fuckeliana* | DHA14-like major facilitator | Pest Manag. Sci. 57 (5), 393-402 (2001) |
| *Fv11983* | AAD45321.1 | *Cochliobolus carbonum* | product="putative branched-chain amino acid | Microbiology 145 (Pt 12), 3539-3546 (1999) |
| *Fv11985* | AAC39448.1 | *Candida albicans* | catalase | Infect. Immun. 66 (5), 1953-1961 (1998) |
| *Fv12026* | XP_759761.1 | *Ustilago maydis* | hypothetical protein | Direct Submission |
| *Fv12151* | ABB43265.1 | *Botrytis elliptica* | necrosis- and ethylene-inducing protein 1 | Fungal Genet Biol. 2007 Jan;44(1):52-63. |
| *Fv12187* | AAV64894.1 | *Cryptococcus neoformans var. grubii* | LAC2 isoform 1 | Eukaryotic Cell 4 (1), 190-201 (2005) |
| *Fv12222* | AAK81847.1 | *Fusarium oxysporum f. sp. radicis-lycopersici* | exopolygalacturonase PGX1 | Direct Submission |
| *Fv12257* | CAA61538.1 | *Candida albicans* | putative mannosyl transferase | J. Biol. Chem. 280 (2), 1051-1060 (2005) |
| *Fv12335* | CAC29255.1 | *Botryotinia fuckeliana* | pectin methyl esterase | Direct Submission |
| *Fv12558* | AAC05829.1 | *Trichoderma virens* | chitinase | Curr. Genet. 35 (1), 41-50 (1999) |
| *Fv12589* | ABB90282.1 | *Gibberella zeae* | polyketide synthase | Mol. Microbiol. 58 (4), 1102-1113 (2005) |
| *Fv12594* | ABB90283.1 | *Gibberella zeae* | polyketide synthase | Mol. Microbiol. 58 (4), 1102-1113 (2005) |
| *Fv12663* | CAD79488.1 | *Ustilago maydis* | Glyoxaloxidase 1 | Mol. Genet. Genomics 272 (6), 639-650 (2005) |
| *Fv12681* | AAP41066.1 | *Cryptococcus neoformans var. grubii* | flavohemoglobin | Curr. Biol. 13 (22), 1963-1968 (2003) |
| *Fv12834* | AAD53262.1 | *Glomerella cingulata* | hard-surface inducible protein | J. Bacteriol. 182 (17), 4688-4695 (2000) |
| *Fv12841* | AAA21151.1 | *Candida albicans* | beta-1,3 glucan transferase | Direct Submission |
| *Fv12904* | ABK60346.1 | *Magnaporthe grisea* | GTP-binding protein | J Bacteriol. 1997 Dec;179(24):7734-41. |
| *Fv12913* | CAI61947.1 | *Aspergillus fumigatus* | methylcitrate synthase | FEBS J. 272 (14), 3615-3630 (2005) |
| *Fv13103* | AAU87358.1 | *Botryotinia fuckeliana* | fructose transporter 1 | Fungal Genet. Biol. 42 (7), 601-610 (2005) |
| *Fv13123* | BAA18962.1 | *Colletotrichum lagenarium* | reductase | Mol. Plant Microbe Interact. 9 (5), 323-329 (1996) |
| *Fv13129* | AAK11167.1 | *Nectria haematococca (related:Fusarium solani)* | PEP2 | Plant J. 25 (3), 305-314 (2001) |
| *Fv13130* | AAK16922.1 | *Nectria haematococca (related:Fusarium solani)* | PEP5 | Plant J. 25 (3), 305-314 (2001) |
| *Fv13189* | EDK03390.1 | *Magnaporthe grisea* | hypothetical protein | Nature 434 (7036), 980-986 (2005) |
| *Fv13252* | AAC49420.1 | *Nectria haematococca (related:Fusarium solani)* | pectate lyase D | Arch. Biochem. Biophys. 332 (2), 305-312 (1996) |
| *Fv13467* | AAK11166.1 | *Nectria haematococca mpVI* | PEP1 | Plant J. 25 (3), 305-314 (2001) |
| *Fv13527* | AAR06609.1 | *Magnaporthe grisea* | extracellular matrix protein | Direct Submission |
| *Fv13749* | AAX14688.1 | *Stagonospora nodorum* | mannitol dehydrogenase | Direct Submission |
| *Fv13798* | EDJ94108.1 | *Magnaporthe oryzae 70-15* | hypothetical protein | Nature 434 (7036), 980-986 (2005) |
| *Fv13901* | AAQ07436.1 | *Coccidioides posadasii* | metalloprotease 1 precursor | Infect. Immun. 73 (10), 6689-6703 (2005) |
| *Fv13985* | AAD13811.1 | *Cochliobolus carbonum* | TOXE protein | Direct Submission |
| *Fv14012* | AAQ16576.1 | *Botryotinia fuckeliana* | CND5p | Mol. Microbiol. 50 (5), 1451-1465 (2003) |
| *Fv14016* | AAM90953.1 | *Gibberella zeae* | trichodiene synthetase | Mycol. Res. 107 (PT 2), 190-197 (2003) |
| *Fv14028* | AAP68994.1 | *Cryptococcus neoformans var. grubii* | thiol-specific antioxidant protein 1 | Mol. Microbiol. 51 (5), 1447-1458 (2004) |
| *Fv14088* | AAZ81480.1 | *Magnaporthe grisea* | MDR efflux pump ABC3 | Plant Cell 18 (12), 3686-3705 (2006) |
| *Fv14106* | AAA33338.1 | *Nectria haematococca (related:Fusarium solani)* | pectate lyase A | Arch. Biochem. Biophys. 258 (1), 196-205 (1987) |
| *Fv14158* | AAA79885.1 | *Cochliobolus carbonum* | endopolygalacturonase | Plant Cell 2 (12), 1191-1200 (1990) |
| *Fv14468* | AAR01218.1 | *Aspergillus fumigatus* | methyltransferase | Eukaryotic Cell 3 (2), 527-535 (2004) |
| *Fv14693* | AAC49410.1 | *Nectria haematococca* | maackiain detoxification | Mol. Gen. Genet. 251 (4), 397-406 (1996) |
| *Fv14725* | BAB69078.1 | *Alternaria alternata* | Aft3-1 | Genetics 161 (1), 59-70 (2002) |
| *Fv14784* | BAB69076.1 | *Alternaria alternata* | involved in AF-toxin biosynthesis | Genetics 161 (1), 59-70 (2002) |
