# Supplementary material for: The Genome Sequence of the Fungal Pathogen Fusarium virguliforme That Causes Sudden Death Syndrome in Soybean
Source: PLoS One. 2014 Jan 14;9(1):e81832. doi: 10.1371/journal.pone.0081832 (PMC3891557; doi:10.1371/journal.pone.0081832)
Supplement: Table S12 — Candidate F. virguliforme secretory proteins. (DOC) [file pone.0081832.s021.doc]

| **Table S12.** Candidate *F. virguliforme* secretory proteins. | | | |
| --- | --- | --- | --- |
| ***Fv* protein** | **Probablity** | **Signal anchor probability** | **Max cleavage site probability** |
| Fv20 | 0.993 | 0 | 0.666 between pos.22 and 23 |
| Fv21 | 0.997 | 0.003 | 0.582 between pos.38 and 39 |
| Fv24 | 0.967 | 0 | 0.773 between pos.20 and 21 |
| Fv30 | 0.984 | 0 | 0.753 between pos.20 and 21 |
| Fv35 | 1 | 0 | 0.951 between pos.18 and 19 |
| Fv64 | 0.998 | 0 | 0.812 between pos.16 and 17 |
| Fv73 | 0.937 | 0 | 0.584 between pos.23 and 24 |
| Fv76 | 0.997 | 0 | 0.442 between pos.19 and 20 |
| Fv80 | 1 | 0 | 0.982 between pos.19 and 20 |
| Fv162 | 0.923 | 0 | 0.566 between pos.24 and 25 |
| Fv168 | 0.906 | 0.004 | 0.212 between pos.33 and 34 |
| Fv172 | 0.999 | 0 | 0.823 between pos.17 and 18 |
| Fv201 | 0.998 | 0 | 0.415 between pos.18 and 19 |
| Fv204 | 0.986 | 0.003 | 0.753 between pos.18 and 19 |
| Fv218 | 0.978 | 0 | 0.886 between pos.20 and 21 |
| Fv228 | 0.94 | 0.018 | 0.828 between pos.34 and 35 |
| Fv263 | 0.995 | 0.001 | 0.992 between pos.21 and 22 |
| Fv266 | 0.999 | 0 | 0.900 between pos.44 and 45 |
| Fv268 | 0.999 | 0 | 0.347 between pos.23 and 24 |
| Fv273 | 0.993 | 0 | 0.594 between pos.18 and 19 |
| Fv285 | 0.971 | 0 | 0.437 between pos.28 and 29 |
| Fv290 | 0.999 | 0 | 0.949 between pos.20 and 21 |
| Fv305 | 0.909 | 0.068 | 0.666 between pos.26 and 27 |
| Fv318 | 1 | 0 | 0.970 between pos.20 and 21 |
| Fv328 | 0.986 | 0 | 0.963 between pos.21 and 22 |
| Fv335 | 0.981 | 0 | 0.932 between pos.18 and 19 |
| Fv336 | 0.999 | 0 | 0.937 between pos.22 and 23 |
| Fv365 | 0.971 | 0 | 0.845 between pos.18 and 19 |
| Fv372 | 0.947 | 0 | 0.335 between pos.16 and 17 |
| Fv378 | 0.999 | 0 | 0.427 between pos.22 and 23 |
| Fv393 | 0.917 | 0.044 | 0.665 between pos.20 and 21 |
| Fv411 | 0.983 | 0 | 0.194 between pos.20 and 21 |
| Fv428 | 0.954 | 0 | 0.684 between pos.25 and 26 |
| Fv518 | 0.98 | 0.013 | 0.719 between pos.29 and 30 |
| Fv556 | 1 | 0 | 0.303 between pos.60 and 61 |
| Fv561 | 0.915 | 0 | 0.752 between pos.19 and 20 |
| Fv575 | 0.927 | 0 | 0.586 between pos.19 and 20 |
| Fv616 | 0.969 | 0 | 0.913 between pos.18 and 19 |
| Fv665 | 0.991 | 0.002 | 0.856 between pos.21 and 22 |
| Fv668 | 0.978 | 0 | 0.607 between pos.19 and 20 |
| Fv670 | 0.955 | 0 | 0.879 between pos.19 and 20 |
| Fv690 | 0.999 | 0 | 0.620 between pos.27 and 28 |
| Fv767 | 0.992 | 0 | 0.991 between pos.21 and 22 |
| Fv770 | 1 | 0 | 0.904 between pos.20 and 21 |
| Fv791 | 0.937 | 0.001 | 0.372 between pos.25 and 26 |
| Fv814 | 0.998 | 0 | 0.951 between pos.17 and 18 |
| Fv865 | 0.98 | 0 | 0.688 between pos.18 and 19 |
| Fv886 | 0.943 | 0 | 0.615 between pos.18 and 19 |
| Fv945 | 0.936 | 0 | 0.407 between pos.16 and 17 |
| Fv1033 | 0.999 | 0.001 | 0.641 between pos.21 and 22 |
| Fv1056 | 0.998 | 0 | 0.885 between pos.21 and 22 |
| Fv1077 | 0.998 | 0 | 0.775 between pos.21 and 22 |
| Fv1084 | 0.996 | 0.004 | 0.947 between pos.30 and 31 |
| Fv1085 | 0.924 | 0 | 0.859 between pos.20 and 21 |
| Fv1119 | 0.901 | 0.043 | 0.893 between pos.34 and 35 |
| Fv1131 | 0.987 | 0 | 0.877 between pos.19 and 20 |
| Fv1137 | 0.998 | 0 | 0.728 between pos.69 and 70 |
| Fv1144 | 1 | 0 | 0.797 between pos.17 and 18 |
| Fv1157 | 1 | 0 | 0.981 between pos.20 and 21 |
| Fv1163 | 0.988 | 0 | 0.964 between pos.17 and 18 |
| Fv1165 | 1 | 0 | 0.984 between pos.21 and 22 |
| Fv1219 | 0.978 | 0 | 0.875 between pos.17 and 18 |
| Fv1244 | 0.995 | 0 | 0.485 between pos.21 and 22 |
| Fv1250 | 1 | 0 | 0.726 between pos.18 and 19 |
| Fv1256 | 0.918 | 0 | 0.838 between pos.30 and 31 |
| Fv1283 | 0.98 | 0 | 0.932 between pos.18 and 19 |
| Fv1320 | 0.983 | 0 | 0.881 between pos.18 and 19 |
| Fv1339 | 1 | 0 | 0.649 between pos.25 and 26 |
| Fv1340 | 1 | 0 | 0.621 between pos.19 and 20 |
| Fv1353 | 1 | 0 | 0.778 between pos.17 and 18 |
| Fv1395 | 0.997 | 0 | 0.975 between pos.17 and 18 |
| Fv1401 | 0.987 | 0 | 0.684 between pos.16 and 17 |
| Fv1417 | 0.992 | 0.007 | 0.781 between pos.22 and 23 |
| Fv1437 | 0.95 | 0 | 0.929 between pos.19 and 20 |
| Fv1441 | 0.95 | 0.049 | 0.719 between pos.60 and 61 |
| Fv1442 | 0.973 | 0 | 0.742 between pos.20 and 21 |
| Fv1449 | 0.988 | 0 | 0.559 between pos.24 and 25 |
| Fv1450 | 0.997 | 0 | 0.768 between pos.16 and 17 |
| Fv1473 | 0.993 | 0 | 0.894 between pos.19 and 20 |
| Fv1567 | 0.994 | 0.005 | 0.533 between pos.44 and 45 |
| Fv1574 | 0.995 | 0.002 | 0.877 between pos.22 and 23 |
| Fv1589 | 0.948 | 0 | 0.788 between pos.19 and 20 |
| Fv1594 | 0.989 | 0 | 0.852 between pos.20 and 21 |
| Fv1595 | 0.991 | 0 | 0.925 between pos.18 and 19 |
| Fv1598 | 1 | 0 | 0.903 between pos.16 and 17 |
| Fv1614 | 0.986 | 0 | 0.950 between pos.23 and 24 |
| Fv1615 | 1 | 0 | 0.950 between pos.19 and 20 |
| Fv1621 | 1 | 0 | 0.783 between pos.19 and 20 |
| Fv1629 | 1 | 0 | 0.906 between pos.21 and 22 |
| Fv1632 | 0.943 | 0 | 0.691 between pos.21 and 22 |
| Fv1634 | 1 | 0 | 0.738 between pos.19 and 20 |
| Fv1636 | 0.99 | 0 | 0.700 between pos.27 and 28 |
| Fv1655 | 0.96 | 0 | 0.907 between pos.19 and 20 |
| Fv1661 | 0.944 | 0 | 0.291 between pos.21 and 22 |
| Fv1662 | 0.964 | 0 | 0.436 between pos.39 and 40 |
| Fv1666 | 1 | 0 | 0.590 between pos.19 and 20 |
| Fv1667 | 0.924 | 0 | 0.694 between pos.20 and 21 |
| Fv1683 | 0.993 | 0 | 0.550 between pos.21 and 22 |
| Fv1713 | 0.925 | 0.002 | 0.809 between pos.23 and 24 |
| Fv1719 | 0.926 | 0.006 | 0.504 between pos.21 and 22 |
| Fv1739 | 1 | 0 | 0.990 between pos.17 and 18 |
| Fv1745 | 0.991 | 0.002 | 0.642 between pos.21 and 22 |
| Fv1747 | 0.988 | 0 | 0.793 between pos.20 and 21 |
| Fv1752 | 0.997 | 0 | 0.602 between pos.20 and 21 |
| Fv1768 | 1 | 0 | 0.863 between pos.20 and 21 |
| Fv1787 | 0.998 | 0 | 0.774 between pos.19 and 20 |
| Fv1800 | 0.946 | 0 | 0.853 between pos.20 and 21 |
| Fv1801 | 0.999 | 0 | 0.821 between pos.19 and 20 |
| Fv1819 | 0.992 | 0 | 0.931 between pos.19 and 20 |
| Fv1838 | 0.981 | 0 | 0.718 between pos.24 and 25 |
| Fv1870 | 0.994 | 0 | 0.867 between pos.21 and 22 |
| Fv1877 | 0.999 | 0 | 0.800 between pos.16 and 17 |
| Fv1891 | 0.943 | 0.053 | 0.347 between pos.28 and 29 |
| Fv1904 | 0.922 | 0.011 | 0.299 between pos.21 and 22 |
| Fv1948 | 0.945 | 0 | 0.753 between pos.31 and 32 |
| Fv1970 | 0.948 | 0.025 | 0.282 between pos.77 and 78 |
| Fv1986 | 0.988 | 0.012 | 0.576 between pos.25 and 26 |
| Fv2022 | 0.999 | 0 | 0.642 between pos.19 and 20 |
| Fv2059 | 0.97 | 0.013 | 0.929 between pos.23 and 24 |
| Fv2090 | 0.99 | 0 | 0.404 between pos.21 and 22 |
| Fv2091 | 0.915 | 0.027 | 0.625 between pos.20 and 21 |
| Fv2135 | 0.999 | 0 | 0.295 between pos.15 and 16 |
| Fv2142 | 0.992 | 0 | 0.918 between pos.20 and 21 |
| Fv2147 | 0.956 | 0 | 0.947 between pos.17 and 18 |
| Fv2166 | 0.996 | 0 | 0.886 between pos.18 and 19 |
| Fv2176 | 0.956 | 0 | 0.667 between pos.21 and 22 |
| Fv2209 | 0.999 | 0 | 0.970 between pos.25 and 26 |
| Fv2228 | 0.998 | 0 | 0.692 between pos.14 and 15 |
| Fv2258 | 0.929 | 0.008 | 0.600 between pos.58 and 59 |
| Fv2278 | 0.998 | 0 | 0.833 between pos.25 and 26 |
| Fv2294 | 0.98 | 0 | 0.808 between pos.21 and 22 |
| Fv2319 | 0.994 | 0 | 0.800 between pos.20 and 21 |
| Fv2369 | 0.991 | 0 | 0.422 between pos.22 and 23 |
| Fv2428 | 0.999 | 0 | 0.954 between pos.32 and 33 |
| Fv2440 | 0.999 | 0 | 0.951 between pos.23 and 24 |
| Fv2463 | 1 | 0 | 0.996 between pos.19 and 20 |
| Fv2465 | 0.995 | 0 | 0.728 between pos.22 and 23 |
| Fv2484 | 0.998 | 0.002 | 0.804 between pos.36 and 37 |
| Fv2500 | 0.935 | 0 | 0.655 between pos.23 and 24 |
| Fv2578 | 0.939 | 0 | 0.794 between pos.17 and 18 |
| Fv2580 | 1 | 0 | 0.896 between pos.43 and 44 |
| Fv2582 | 0.998 | 0 | 0.911 between pos.19 and 20 |
| Fv2586 | 0.97 | 0.016 | 0.452 between pos.28 and 29 |
| Fv2602 | 0.993 | 0 | 0.684 between pos.22 and 23 |
| Fv2624 | 0.923 | 0 | 0.450 between pos.19 and 20 |
| Fv2650 | 0.938 | 0.002 | 0.456 between pos.20 and 21 |
| Fv2658 | 0.987 | 0.001 | 0.680 between pos.32 and 33 |
| Fv2681 | 1 | 0 | 0.994 between pos.23 and 24 |
| Fv2702 | 1 | 0 | 0.515 between pos.20 and 21 |
| Fv2715 | 0.928 | 0 | 0.871 between pos.21 and 22 |
| Fv2740 | 0.963 | 0.028 | 0.490 between pos.21 and 22 |
| Fv2753 | 0.98 | 0 | 0.295 between pos.24 and 25 |
| Fv2768 | 0.998 | 0 | 0.760 between pos.17 and 18 |
| Fv2798 | 0.972 | 0 | 0.369 between pos.22 and 23 |
| Fv2802 | 0.999 | 0 | 0.942 between pos.19 and 20 |
| Fv2811 | 0.993 | 0 | 0.985 between pos.16 and 17 |
| Fv2825 | 0.988 | 0 | 0.784 between pos.20 and 21 |
| Fv2838 | 0.999 | 0.001 | 0.559 between pos.27 and 28 |
| Fv2866 | 0.999 | 0 | 0.504 between pos.20 and 21 |
| Fv2868 | 0.967 | 0 | 0.362 between pos.17 and 18 |
| Fv2897 | 0.999 | 0 | 0.501 between pos.18 and 19 |
| Fv2906 | 0.996 | 0 | 0.964 between pos.16 and 17 |
| Fv2909 | 0.949 | 0 | 0.915 between pos.19 and 20 |
| Fv2945 | 0.999 | 0 | 0.936 between pos.18 and 19 |
| Fv2960 | 0.993 | 0 | 0.680 between pos.21 and 22 |
| Fv2970 | 0.968 | 0 | 0.964 between pos.19 and 20 |
| Fv2986 | 0.97 | 0 | 0.947 between pos.18 and 19 |
| Fv2987 | 0.932 | 0 | 0.742 between pos.24 and 25 |
| Fv3010 | 0.998 | 0 | 0.972 between pos.23 and 24 |
| Fv3013 | 0.996 | 0 | 0.928 between pos.17 and 18 |
| Fv3017 | 0.996 | 0 | 0.886 between pos.19 and 20 |
| Fv3023 | 0.995 | 0 | 0.926 between pos.16 and 17 |
| Fv3038 | 0.973 | 0.016 | 0.307 between pos.35 and 36 |
| Fv3057 | 0.981 | 0 | 0.956 between pos.18 and 19 |
| Fv3068 | 1 | 0 | 0.641 between pos.16 and 17 |
| Fv3069 | 0.937 | 0.001 | 0.552 between pos.18 and 19 |
| Fv3079 | 0.93 | 0 | 0.573 between pos.24 and 25 |
| Fv3084 | 0.983 | 0 | 0.592 between pos.19 and 20 |
| Fv3098 | 0.999 | 0 | 0.836 between pos.20 and 21 |
| Fv3105 | 0.983 | 0 | 0.618 between pos.35 and 36 |
| Fv3121 | 0.999 | 0 | 0.991 between pos.19 and 20 |
| Fv3123 | 0.994 | 0 | 0.974 between pos.26 and 27 |
| Fv3125 | 1 | 0 | 0.734 between pos.17 and 18 |
| Fv3127 | 1 | 0 | 0.666 between pos.16 and 17 |
| Fv3134 | 0.988 | 0 | 0.900 between pos.18 and 19 |
| Fv3136 | 1 | 0 | 0.989 between pos.18 and 19 |
| Fv3174 | 0.988 | 0 | 0.787 between pos.16 and 17 |
| Fv3179 | 0.912 | 0.016 | 0.539 between pos.46 and 47 |
| Fv3245 | 0.975 | 0.019 | 0.603 between pos.38 and 39 |
| Fv3271 | 0.965 | 0.001 | 0.840 between pos.43 and 44 |
| Fv3277 | 0.902 | 0.094 | 0.797 between pos.60 and 61 |
| Fv3295 | 0.999 | 0 | 0.551 between pos.18 and 19 |
| Fv3302 | 0.9 | 0 | 0.712 between pos.22 and 23 |
| Fv3308 | 0.999 | 0 | 0.774 between pos.19 and 20 |
| Fv3310 | 0.96 | 0 | 0.872 between pos.17 and 18 |
| Fv3331 | 0.987 | 0 | 0.920 between pos.21 and 22 |
| Fv3332 | 0.996 | 0 | 0.470 between pos.24 and 25 |
| Fv3334 | 0.998 | 0 | 0.964 between pos.17 and 18 |
| Fv3347 | 0.997 | 0 | 0.978 between pos.22 and 23 |
| Fv3372 | 0.996 | 0 | 0.669 between pos.19 and 20 |
| Fv3376 | 0.994 | 0 | 0.755 between pos.18 and 19 |
| Fv3396 | 0.922 | 0 | 0.267 between pos.21 and 22 |
| Fv3402 | 0.977 | 0 | 0.626 between pos.20 and 21 |
| Fv3412 | 0.92 | 0.001 | 0.909 between pos.20 and 21 |
| Fv3425 | 1 | 0 | 0.986 between pos.19 and 20 |
| Fv3431 | 0.997 | 0.001 | 0.663 between pos.22 and 23 |
| Fv3451 | 0.95 | 0 | 0.614 between pos.25 and 26 |
| Fv3463 | 1 | 0 | 0.730 between pos.19 and 20 |
| Fv3470 | 0.994 | 0 | 0.851 between pos.21 and 22 |
| Fv3480 | 0.963 | 0 | 0.891 between pos.21 and 22 |
| Fv3517 | 0.915 | 0 | 0.658 between pos.17 and 18 |
| Fv3539 | 0.997 | 0 | 0.785 between pos.22 and 23 |
| Fv3574 | 0.999 | 0 | 0.760 between pos.16 and 17 |
| Fv3592 | 0.996 | 0 | 0.914 between pos.16 and 17 |
| Fv3616 | 0.943 | 0.053 | 0.620 between pos.30 and 31 |
| Fv3632 | 1 | 0 | 0.618 between pos.17 and 18 |
| Fv3638 | 0.979 | 0 | 0.736 between pos.38 and 39 |
| Fv3691 | 0.98 | 0.002 | 0.759 between pos.31 and 32 |
| Fv3692 | 0.976 | 0.016 | 0.928 between pos.30 and 31 |
| Fv3708 | 0.987 | 0.013 | 0.964 between pos.26 and 27 |
| Fv3716 | 0.954 | 0.002 | 0.539 between pos.31 and 32 |
| Fv3749 | 0.987 | 0 | 0.793 between pos.19 and 20 |
| Fv3752 | 0.994 | 0 | 0.986 between pos.22 and 23 |
| Fv3763 | 1 | 0 | 0.840 between pos.23 and 24 |
| Fv3768 | 0.959 | 0 | 0.938 between pos.22 and 23 |
| Fv3770 | 0.961 | 0 | 0.668 between pos.17 and 18 |
| Fv3772 | 0.924 | 0 | 0.865 between pos.24 and 25 |
| Fv3791 | 0.914 | 0.079 | 0.307 between pos.30 and 31 |
| Fv3796 | 0.987 | 0 | 0.387 between pos.25 and 26 |
| Fv3804 | 0.998 | 0.001 | 0.909 between pos.23 and 24 |
| Fv3809 | 0.905 | 0 | 0.822 between pos.17 and 18 |
| Fv3816 | 0.99 | 0.006 | 0.335 between pos.31 and 32 |
| Fv3820 | 0.998 | 0 | 0.571 between pos.17 and 18 |
| Fv3857 | 0.998 | 0 | 0.391 between pos.21 and 22 |
| Fv3867 | 0.998 | 0 | 0.959 between pos.18 and 19 |
| Fv3873 | 1 | 0 | 0.707 between pos.23 and 24 |
| Fv3890 | 0.998 | 0 | 0.888 between pos.17 and 18 |
| Fv3901 | 1 | 0 | 0.990 between pos.23 and 24 |
| Fv3913 | 1 | 0 | 0.685 between pos.18 and 19 |
| Fv3916 | 0.956 | 0 | 0.302 between pos.24 and 25 |
| Fv3917 | 0.999 | 0 | 0.766 between pos.15 and 16 |
| Fv3932 | 0.986 | 0.001 | 0.789 between pos.30 and 31 |
| Fv3939 | 0.993 | 0 | 0.957 between pos.18 and 19 |
| Fv3962 | 0.999 | 0 | 0.996 between pos.17 and 18 |
| Fv3965 | 0.998 | 0 | 0.983 between pos.26 and 27 |
| Fv4032 | 0.992 | 0 | 0.890 between pos.17 and 18 |
| Fv4057 | 0.998 | 0 | 0.477 between pos.16 and 17 |
| Fv4071 | 0.945 | 0.017 | 0.368 between pos.28 and 29 |
| Fv4072 | 0.927 | 0 | 0.825 between pos.21 and 22 |
| Fv4074 | 0.999 | 0 | 0.600 between pos.20 and 21 |
| Fv4079 | 0.994 | 0 | 0.549 between pos.27 and 28 |
| Fv4084 | 0.996 | 0.002 | 0.682 between pos.30 and 31 |
| Fv4109 | 0.953 | 0 | 0.851 between pos.20 and 21 |
| Fv4118 | 0.991 | 0 | 0.819 between pos.18 and 19 |
| Fv4133 | 0.925 | 0 | 0.772 between pos.18 and 19 |
| Fv4140 | 0.942 | 0 | 0.938 between pos.18 and 19 |
| Fv4142 | 0.998 | 0 | 0.930 between pos.19 and 20 |
| Fv4158 | 0.998 | 0.001 | 0.969 between pos.23 and 24 |
| Fv4241 | 0.957 | 0 | 0.789 between pos.19 and 20 |
| Fv4243 | 0.991 | 0 | 0.295 between pos.25 and 26 |
| Fv4245 | 0.997 | 0 | 0.876 between pos.26 and 27 |
| Fv4246 | 0.999 | 0 | 0.475 between pos.21 and 22 |
| Fv4261 | 0.94 | 0 | 0.653 between pos.18 and 19 |
| Fv4285 | 1 | 0 | 0.921 between pos.18 and 19 |
| Fv4306 | 0.999 | 0 | 0.406 between pos.26 and 27 |
| Fv4330 | 0.951 | 0.002 | 0.711 between pos.29 and 30 |
| Fv4342 | 0.98 | 0.01 | 0.808 between pos.31 and 32 |
| Fv4369 | 0.999 | 0 | 0.548 between pos.21 and 22 |
| Fv4397 | 0.992 | 0 | 0.681 between pos.35 and 36 |
| Fv4414 | 0.993 | 0 | 0.920 between pos.21 and 22 |
| Fv4418 | 0.999 | 0.001 | 0.941 between pos.27 and 28 |
| Fv4420 | 0.989 | 0 | 0.857 between pos.18 and 19 |
| Fv4441 | 0.981 | 0 | 0.597 between pos.17 and 18 |
| Fv4474 | 0.972 | 0.024 | 0.639 between pos.18 and 19 |
| Fv4479 | 0.925 | 0.028 | 0.704 between pos.24 and 25 |
| Fv4482 | 0.998 | 0 | 0.955 between pos.19 and 20 |
| Fv4510 | 0.991 | 0 | 0.955 between pos.18 and 19 |
| Fv4519 | 0.999 | 0 | 0.947 between pos.21 and 22 |
| Fv4533 | 0.993 | 0 | 0.831 between pos.18 and 19 |
| Fv4535 | 0.999 | 0 | 0.431 between pos.17 and 18 |
| Fv4536 | 0.998 | 0 | 0.635 between pos.18 and 19 |
| Fv4542 | 0.998 | 0 | 0.809 between pos.20 and 21 |
| Fv4551 | 0.945 | 0 | 0.913 between pos.24 and 25 |
| Fv4553 | 0.96 | 0 | 0.702 between pos.19 and 20 |
| Fv4575 | 0.959 | 0 | 0.528 between pos.20 and 21 |
| Fv4576 | 0.999 | 0 | 0.965 between pos.21 and 22 |
| Fv4581 | 1 | 0 | 0.822 between pos.21 and 22 |
| Fv4584 | 0.97 | 0 | 0.898 between pos.26 and 27 |
| Fv4587 | 0.994 | 0 | 0.376 between pos.20 and 21 |
| Fv4588 | 0.926 | 0 | 0.467 between pos.20 and 21 |
| Fv4604 | 1 | 0 | 0.624 between pos.27 and 28 |
| Fv4614 | 0.915 | 0 | 0.525 between pos.21 and 22 |
| Fv4648 | 0.997 | 0 | 0.753 between pos.19 and 20 |
| Fv4665 | 0.972 | 0 | 0.733 between pos.18 and 19 |
| Fv4708 | 0.994 | 0.003 | 0.534 between pos.20 and 21 |
| Fv4711 | 0.993 | 0 | 0.937 between pos.20 and 21 |
| Fv4714 | 0.999 | 0 | 0.519 between pos.19 and 20 |
| Fv4758 | 1 | 0 | 0.981 between pos.22 and 23 |
| Fv4759 | 0.948 | 0.027 | 0.389 between pos.41 and 42 |
| Fv4805 | 0.988 | 0.012 | 0.355 between pos.33 and 34 |
| Fv4827 | 0.97 | 0 | 0.955 between pos.19 and 20 |
| Fv4858 | 0.998 | 0 | 0.905 between pos.15 and 16 |
| Fv4893 | 1 | 0 | 0.583 between pos.25 and 26 |
| Fv4939 | 0.996 | 0 | 0.494 between pos.16 and 17 |
| Fv4954 | 0.98 | 0.007 | 0.633 between pos.30 and 31 |
| Fv4964 | 0.965 | 0 | 0.600 between pos.19 and 20 |
| Fv4965 | 0.976 | 0 | 0.562 between pos.21 and 22 |
| Fv4966 | 0.945 | 0.002 | 0.576 between pos.15 and 16 |
| Fv4969 | 0.996 | 0 | 0.540 between pos.23 and 24 |
| Fv4976 | 0.985 | 0 | 0.935 between pos.24 and 25 |
| Fv5030 | 0.999 | 0 | 0.896 between pos.22 and 23 |
| Fv5058 | 1 | 0 | 0.478 between pos.25 and 26 |
| Fv5115 | 0.973 | 0 | 0.782 between pos.24 and 25 |
| Fv5139 | 1 | 0 | 0.669 between pos.17 and 18 |
| Fv5174 | 0.995 | 0 | 0.932 between pos.28 and 29 |
| Fv5228 | 0.98 | 0 | 0.581 between pos.20 and 21 |
| Fv5239 | 0.995 | 0 | 0.936 between pos.15 and 16 |
| Fv5290 | 1 | 0 | 0.766 between pos.26 and 27 |
| Fv5310 | 1 | 0 | 0.329 between pos.20 and 21 |
| Fv5318 | 0.999 | 0 | 0.291 between pos.21 and 22 |
| Fv5333 | 0.97 | 0 | 0.908 between pos.29 and 30 |
| Fv5351 | 0.998 | 0.001 | 0.727 between pos.83 and 84 |
| Fv5361 | 0.995 | 0 | 0.803 between pos.15 and 16 |
| Fv5363 | 0.999 | 0 | 0.972 between pos.21 and 22 |
| Fv5364 | 0.938 | 0 | 0.924 between pos.19 and 20 |
| Fv5389 | 0.912 | 0.087 | 0.498 between pos.33 and 34 |
| Fv5390 | 0.996 | 0 | 0.952 between pos.15 and 16 |
| Fv5411 | 1 | 0 | 0.724 between pos.22 and 23 |
| Fv5437 | 0.998 | 0 | 0.907 between pos.17 and 18 |
| Fv5450 | 0.999 | 0 | 0.920 between pos.15 and 16 |
| Fv5452 | 0.924 | 0 | 0.752 between pos.17 and 18 |
| Fv5454 | 0.996 | 0 | 0.905 between pos.17 and 18 |
| Fv5461 | 1 | 0 | 0.807 between pos.18 and 19 |
| Fv5462 | 0.998 | 0 | 0.880 between pos.23 and 24 |
| Fv5464 | 0.995 | 0 | 0.798 between pos.20 and 21 |
| Fv5490 | 0.963 | 0 | 0.734 between pos.20 and 21 |
| Fv5504 | 1 | 0 | 0.491 between pos.20 and 21 |
| Fv5506 | 1 | 0 | 0.303 between pos.17 and 18 |
| Fv5509 | 0.99 | 0 | 0.910 between pos.23 and 24 |
| Fv5513 | 0.966 | 0 | 0.932 between pos.17 and 18 |
| Fv5530 | 0.942 | 0 | 0.794 between pos.19 and 20 |
| Fv5553 | 0.998 | 0 | 0.988 between pos.17 and 18 |
| Fv5580 | 0.912 | 0 | 0.463 between pos.18 and 19 |
| Fv5591 | 0.994 | 0 | 0.922 between pos.22 and 23 |
| Fv5602 | 0.941 | 0.052 | 0.933 between pos.47 and 48 |
| Fv5625 | 0.999 | 0 | 0.943 between pos.18 and 19 |
| Fv5637 | 0.996 | 0 | 0.621 between pos.20 and 21 |
| Fv5639 | 0.968 | 0.008 | 0.166 between pos.42 and 43 |
| Fv5645 | 0.966 | 0 | 0.945 between pos.16 and 17 |
| Fv5647 | 0.989 | 0 | 0.632 between pos.17 and 18 |
| Fv5648 | 0.998 | 0 | 0.972 between pos.16 and 17 |
| Fv5717 | 0.999 | 0 | 0.917 between pos.17 and 18 |
| Fv5735 | 0.984 | 0.004 | 0.533 between pos.28 and 29 |
| Fv5742 | 0.995 | 0 | 0.834 between pos.18 and 19 |
| Fv5776 | 0.979 | 0 | 0.299 between pos.28 and 29 |
| Fv5815 | 0.948 | 0.051 | 0.688 between pos.22 and 23 |
| Fv5822 | 1 | 0 | 0.991 between pos.21 and 22 |
| Fv5840 | 0.969 | 0.023 | 0.455 between pos.42 and 43 |
| Fv5855 | 0.969 | 0 | 0.581 between pos.19 and 20 |
| Fv5874 | 1 | 0 | 0.987 between pos.24 and 25 |
| Fv5875 | 0.998 | 0 | 0.836 between pos.19 and 20 |
| Fv5883 | 0.955 | 0 | 0.540 between pos.22 and 23 |
| Fv5889 | 1 | 0 | 0.947 between pos.20 and 21 |
| Fv5892 | 1 | 0 | 0.449 between pos.18 and 19 |
| Fv5915 | 0.942 | 0 | 0.713 between pos.17 and 18 |
| Fv5916 | 0.998 | 0 | 0.674 between pos.21 and 22 |
| Fv5946 | 0.962 | 0 | 0.755 between pos.18 and 19 |
| Fv5949 | 0.966 | 0.026 | 0.631 between pos.34 and 35 |
| Fv5965 | 0.997 | 0 | 0.855 between pos.18 and 19 |
| Fv5975 | 0.938 | 0 | 0.484 between pos.19 and 20 |
| Fv5978 | 0.989 | 0 | 0.383 between pos.19 and 20 |
| Fv5984 | 0.999 | 0 | 0.896 between pos.18 and 19 |
| Fv5992 | 0.998 | 0 | 0.567 between pos.23 and 24 |
| Fv5993 | 0.996 | 0 | 0.912 between pos.20 and 21 |
| Fv5997 | 0.969 | 0 | 0.513 between pos.22 and 23 |
| Fv6002 | 0.979 | 0 | 0.942 between pos.17 and 18 |
| Fv6003 | 0.977 | 0 | 0.972 between pos.20 and 21 |
| Fv6013 | 0.94 | 0 | 0.937 between pos.20 and 21 |
| Fv6027 | 0.999 | 0 | 0.942 between pos.22 and 23 |
| Fv6028 | 0.999 | 0 | 0.978 between pos.27 and 28 |
| Fv6040 | 0.995 | 0 | 0.824 between pos.18 and 19 |
| Fv6055 | 0.998 | 0 | 0.526 between pos.17 and 18 |
| Fv6063 | 0.974 | 0 | 0.967 between pos.22 and 23 |
| Fv6066 | 1 | 0 | 0.701 between pos.16 and 17 |
| Fv6090 | 0.997 | 0 | 0.975 between pos.23 and 24 |
| Fv6094 | 0.999 | 0 | 0.928 between pos.21 and 22 |
| Fv6104 | 0.999 | 0 | 0.995 between pos.20 and 21 |
| Fv6107 | 0.995 | 0 | 0.526 between pos.18 and 19 |
| Fv6137 | 0.999 | 0 | 0.948 between pos.17 and 18 |
| Fv6140 | 0.963 | 0.007 | 0.788 between pos.29 and 30 |
| Fv6179 | 1 | 0 | 0.968 between pos.20 and 21 |
| Fv6188 | 1 | 0 | 0.990 between pos.26 and 27 |
| Fv6198 | 0.998 | 0.001 | 0.697 between pos.26 and 27 |
| Fv6202 | 1 | 0 | 0.946 between pos.26 and 27 |
| Fv6244 | 1 | 0 | 0.941 between pos.20 and 21 |
| Fv6259 | 0.968 | 0 | 0.374 between pos.20 and 21 |
| Fv6265 | 0.964 | 0 | 0.836 between pos.15 and 16 |
| Fv6303 | 0.986 | 0 | 0.282 between pos.21 and 22 |
| Fv6305 | 0.999 | 0 | 0.722 between pos.25 and 26 |
| Fv6314 | 0.914 | 0.078 | 0.842 between pos.24 and 25 |
| Fv6330 | 0.99 | 0 | 0.662 between pos.38 and 39 |
| Fv6341 | 0.998 | 0 | 0.942 between pos.18 and 19 |
| Fv6365 | 0.956 | 0 | 0.933 between pos.21 and 22 |
| Fv6372 | 1 | 0 | 0.960 between pos.18 and 19 |
| Fv6394 | 0.948 | 0.038 | 0.557 between pos.28 and 29 |
| Fv6400 | 0.999 | 0 | 0.563 between pos.18 and 19 |
| Fv6413 | 0.999 | 0.001 | 0.931 between pos.20 and 21 |
| Fv6480 | 0.905 | 0 | 0.376 between pos.19 and 20 |
| Fv6481 | 0.979 | 0.001 | 0.528 between pos.15 and 16 |
| Fv6485 | 0.992 | 0 | 0.641 between pos.23 and 24 |
| Fv6491 | 0.996 | 0 | 0.831 between pos.18 and 19 |
| Fv6498 | 1 | 0 | 0.902 between pos.16 and 17 |
| Fv6537 | 0.996 | 0 | 0.767 between pos.17 and 18 |
| Fv6539 | 0.982 | 0 | 0.349 between pos.14 and 15 |
| Fv6543 | 0.994 | 0 | 0.920 between pos.18 and 19 |
| Fv6556 | 0.999 | 0 | 0.918 between pos.21 and 22 |
| Fv6576 | 0.985 | 0.014 | 0.446 between pos.25 and 26 |
| Fv6577 | 0.936 | 0.054 | 0.795 between pos.35 and 36 |
| Fv6583 | 0.994 | 0 | 0.894 between pos.26 and 27 |
| Fv6585 | 0.999 | 0 | 0.865 between pos.18 and 19 |
| Fv6599 | 0.998 | 0 | 0.577 between pos.19 and 20 |
| Fv6604 | 0.999 | 0 | 0.930 between pos.20 and 21 |
| Fv6608 | 0.918 | 0.002 | 0.455 between pos.28 and 29 |
| Fv6615 | 1 | 0 | 0.667 between pos.16 and 17 |
| Fv6634 | 1 | 0 | 0.843 between pos.25 and 26 |
| Fv6644 | 0.977 | 0 | 0.856 between pos.20 and 21 |
| Fv6663 | 0.976 | 0 | 0.712 between pos.22 and 23 |
| Fv6716 | 1 | 0 | 0.902 between pos.33 and 34 |
| Fv6743 | 0.999 | 0 | 0.522 between pos.17 and 18 |
| Fv6774 | 0.913 | 0.004 | 0.356 between pos.25 and 26 |
| Fv6789 | 0.981 | 0.008 | 0.497 between pos.26 and 27 |
| Fv6821 | 0.987 | 0 | 0.588 between pos.22 and 23 |
| Fv6825 | 0.925 | 0 | 0.526 between pos.17 and 18 |
| Fv6833 | 0.996 | 0.001 | 0.266 between pos.28 and 29 |
| Fv6835 | 0.942 | 0 | 0.414 between pos.17 and 18 |
| Fv6853 | 0.983 | 0 | 0.672 between pos.27 and 28 |
| Fv6857 | 0.995 | 0 | 0.845 between pos.23 and 24 |
| Fv6863 | 0.999 | 0 | 0.591 between pos.19 and 20 |
| Fv6905 | 0.999 | 0 | 0.624 between pos.19 and 20 |
| Fv6924 | 0.999 | 0 | 0.943 between pos.19 and 20 |
| Fv6984 | 0.998 | 0 | 0.834 between pos.18 and 19 |
| Fv7010 | 0.904 | 0.084 | 0.641 between pos.35 and 36 |
| Fv7049 | 0.903 | 0.011 | 0.652 between pos.27 and 28 |
| Fv7075 | 0.996 | 0 | 0.915 between pos.21 and 22 |
| Fv7076 | 0.999 | 0 | 0.798 between pos.17 and 18 |
| Fv7084 | 0.991 | 0 | 0.926 between pos.23 and 24 |
| Fv7119 | 0.998 | 0 | 0.541 between pos.20 and 21 |
| Fv7137 | 0.963 | 0.001 | 0.646 between pos.36 and 37 |
| Fv7141 | 0.998 | 0 | 0.415 between pos.21 and 22 |
| Fv7151 | 0.98 | 0.013 | 0.951 between pos.37 and 38 |
| Fv7171 | 0.999 | 0 | 0.885 between pos.20 and 21 |
| Fv7253 | 0.95 | 0 | 0.403 between pos.22 and 23 |
| Fv7283 | 0.979 | 0.002 | 0.531 between pos.39 and 40 |
| Fv7293 | 0.997 | 0 | 0.284 between pos.18 and 19 |
| Fv7318 | 0.938 | 0 | 0.477 between pos.20 and 21 |
| Fv7320 | 0.989 | 0 | 0.484 between pos.18 and 19 |
| Fv7324 | 0.959 | 0 | 0.689 between pos.18 and 19 |
| Fv7334 | 0.955 | 0 | 0.574 between pos.27 and 28 |
| Fv7361 | 0.993 | 0 | 0.617 between pos.15 and 16 |
| Fv7363 | 0.978 | 0 | 0.395 between pos.17 and 18 |
| Fv7387 | 0.982 | 0 | 0.718 between pos.21 and 22 |
| Fv7470 | 1 | 0 | 0.545 between pos.18 and 19 |
| Fv7473 | 0.968 | 0.031 | 0.601 between pos.47 and 48 |
| Fv7477 | 0.992 | 0 | 0.673 between pos.19 and 20 |
| Fv7482 | 1 | 0 | 0.619 between pos.20 and 21 |
| Fv7485 | 0.909 | 0.02 | 0.539 between pos.34 and 35 |
| Fv7506 | 0.969 | 0.027 | 0.602 between pos.26 and 27 |
| Fv7508 | 0.975 | 0 | 0.375 between pos.22 and 23 |
| Fv7518 | 0.997 | 0 | 0.941 between pos.18 and 19 |
| Fv7528 | 0.947 | 0 | 0.684 between pos.20 and 21 |
| Fv7529 | 0.992 | 0 | 0.368 between pos.14 and 15 |
| Fv7538 | 0.959 | 0 | 0.654 between pos.18 and 19 |
| Fv7542 | 0.999 | 0 | 0.986 between pos.20 and 21 |
| Fv7545 | 0.983 | 0 | 0.728 between pos.26 and 27 |
| Fv7569 | 1 | 0 | 0.667 between pos.20 and 21 |
| Fv7572 | 0.906 | 0 | 0.890 between pos.27 and 28 |
| Fv7574 | 1 | 0 | 0.789 between pos.18 and 19 |
| Fv7576 | 0.99 | 0 | 0.835 between pos.16 and 17 |
| Fv7586 | 0.999 | 0 | 0.947 between pos.22 and 23 |
| Fv7587 | 0.977 | 0 | 0.949 between pos.22 and 23 |
| Fv7592 | 0.926 | 0 | 0.291 between pos.47 and 48 |
| Fv7597 | 0.953 | 0 | 0.893 between pos.16 and 17 |
| Fv7598 | 0.991 | 0.001 | 0.757 between pos.20 and 21 |
| Fv7608 | 0.995 | 0 | 0.881 between pos.18 and 19 |
| Fv7623 | 0.947 | 0.035 | 0.315 between pos.22 and 23 |
| Fv7635 | 0.999 | 0 | 0.516 between pos.17 and 18 |
| Fv7637 | 0.999 | 0 | 0.758 between pos.20 and 21 |
| Fv7638 | 1 | 0 | 0.500 between pos.19 and 20 |
| Fv7655 | 0.97 | 0 | 0.922 between pos.25 and 26 |
| Fv7658 | 0.996 | 0 | 0.882 between pos.19 and 20 |
| Fv7661 | 0.998 | 0.002 | 0.916 between pos.25 and 26 |
| Fv7676 | 0.996 | 0 | 0.656 between pos.16 and 17 |
| Fv7684 | 1 | 0 | 0.439 between pos.17 and 18 |
| Fv7692 | 0.969 | 0 | 0.939 between pos.21 and 22 |
| Fv7694 | 0.998 | 0 | 0.811 between pos.18 and 19 |
| Fv7700 | 0.999 | 0 | 0.858 between pos.24 and 25 |
| Fv7707 | 0.931 | 0 | 0.740 between pos.20 and 21 |
| Fv7716 | 0.996 | 0 | 0.661 between pos.22 and 23 |
| Fv7723 | 0.903 | 0 | 0.355 between pos.17 and 18 |
| Fv7772 | 1 | 0 | 0.879 between pos.24 and 25 |
| Fv7777 | 0.992 | 0 | 0.919 between pos.19 and 20 |
| Fv7779 | 0.98 | 0 | 0.621 between pos.21 and 22 |
| Fv7815 | 0.998 | 0 | 0.963 between pos.19 and 20 |
| Fv7823 | 0.999 | 0 | 0.904 between pos.20 and 21 |
| Fv7838 | 0.999 | 0 | 0.843 between pos.22 and 23 |
| Fv7855 | 0.992 | 0 | 0.377 between pos.20 and 21 |
| Fv7867 | 0.999 | 0 | 0.934 between pos.21 and 22 |
| Fv7891 | 0.953 | 0.03 | 0.476 between pos.35 and 36 |
| Fv7923 | 0.943 | 0.004 | 0.218 between pos.27 and 28 |
| Fv7990 | 0.998 | 0 | 0.986 between pos.19 and 20 |
| Fv8007 | 1 | 0 | 0.913 between pos.19 and 20 |
| Fv8017 | 0.999 | 0 | 0.874 between pos.23 and 24 |
| Fv8021 | 0.999 | 0 | 0.844 between pos.21 and 22 |
| Fv8023 | 0.988 | 0.005 | 0.624 between pos.24 and 25 |
| Fv8048 | 0.999 | 0 | 0.730 between pos.18 and 19 |
| Fv8067 | 1 | 0 | 0.483 between pos.20 and 21 |
| Fv8079 | 0.999 | 0 | 0.660 between pos.23 and 24 |
| Fv8081 | 0.998 | 0 | 0.940 between pos.19 and 20 |
| Fv8122 | 0.994 | 0 | 0.944 between pos.18 and 19 |
| Fv8142 | 0.992 | 0.003 | 0.392 between pos.38 and 39 |
| Fv8155 | 0.976 | 0 | 0.925 between pos.17 and 18 |
| Fv8200 | 0.996 | 0 | 0.371 between pos.18 and 19 |
| Fv8204 | 0.982 | 0 | 0.857 between pos.20 and 21 |
| Fv8206 | 0.938 | 0.013 | 0.407 between pos.34 and 35 |
| Fv8219 | 0.999 | 0 | 0.921 between pos.18 and 19 |
| Fv8221 | 0.999 | 0 | 0.742 between pos.18 and 19 |
| Fv8250 | 0.987 | 0 | 0.968 between pos.18 and 19 |
| Fv8252 | 0.997 | 0 | 0.768 between pos.21 and 22 |
| Fv8259 | 0.998 | 0 | 0.916 between pos.22 and 23 |
| Fv8275 | 1 | 0 | 0.562 between pos.17 and 18 |
| Fv8276 | 0.993 | 0 | 0.773 between pos.19 and 20 |
| Fv8296 | 0.984 | 0.001 | 0.915 between pos.24 and 25 |
| Fv8302 | 0.992 | 0 | 0.974 between pos.24 and 25 |
| Fv8310 | 0.996 | 0 | 0.936 between pos.21 and 22 |
| Fv8328 | 0.996 | 0 | 0.969 between pos.18 and 19 |
| Fv8351 | 1 | 0 | 0.654 between pos.17 and 18 |
| Fv8386 | 1 | 0 | 0.983 between pos.20 and 21 |
| Fv8392 | 0.998 | 0 | 0.950 between pos.18 and 19 |
| Fv8397 | 0.988 | 0 | 0.709 between pos.21 and 22 |
| Fv8414 | 0.914 | 0 | 0.297 between pos.21 and 22 |
| Fv8421 | 0.903 | 0 | 0.711 between pos.18 and 19 |
| Fv8424 | 0.994 | 0 | 0.783 between pos.17 and 18 |
| Fv8436 | 0.994 | 0 | 0.404 between pos.20 and 21 |
| Fv8441 | 0.916 | 0 | 0.681 between pos.18 and 19 |
| Fv8444 | 0.997 | 0 | 0.805 between pos.21 and 22 |
| Fv8462 | 0.999 | 0 | 0.809 between pos.20 and 21 |
| Fv8463 | 0.985 | 0 | 0.980 between pos.20 and 21 |
| Fv8464 | 0.998 | 0 | 0.888 between pos.22 and 23 |
| Fv8465 | 0.994 | 0 | 0.396 between pos.20 and 21 |
| Fv8474 | 0.948 | 0 | 0.839 between pos.16 and 17 |
| Fv8479 | 0.994 | 0 | 0.853 between pos.19 and 20 |
| Fv8489 | 0.997 | 0 | 0.655 between pos.18 and 19 |
| Fv8494 | 0.911 | 0 | 0.460 between pos.22 and 23 |
| Fv8500 | 0.998 | 0 | 0.906 between pos.19 and 20 |
| Fv8523 | 0.973 | 0 | 0.729 between pos.20 and 21 |
| Fv8524 | 0.996 | 0 | 0.398 between pos.25 and 26 |
| Fv8530 | 0.988 | 0 | 0.882 between pos.21 and 22 |
| Fv8559 | 1 | 0 | 0.539 between pos.17 and 18 |
| Fv8562 | 0.98 | 0 | 0.832 between pos.17 and 18 |
| Fv8574 | 1 | 0 | 0.847 between pos.18 and 19 |
| Fv8576 | 0.984 | 0 | 0.438 between pos.18 and 19 |
| Fv8577 | 0.91 | 0 | 0.474 between pos.23 and 24 |
| Fv8583 | 0.979 | 0 | 0.859 between pos.19 and 20 |
| Fv8584 | 0.97 | 0 | 0.894 between pos.30 and 31 |
| Fv8587 | 0.984 | 0.001 | 0.344 between pos.26 and 27 |
| Fv8588 | 0.975 | 0.001 | 0.932 between pos.20 and 21 |
| Fv8591 | 0.961 | 0 | 0.923 between pos.20 and 21 |
| Fv8592 | 0.994 | 0.001 | 0.942 between pos.22 and 23 |
| Fv8597 | 0.997 | 0 | 0.459 between pos.18 and 19 |
| Fv8598 | 0.961 | 0 | 0.745 between pos.22 and 23 |
| Fv8611 | 0.997 | 0 | 0.985 between pos.21 and 22 |
| Fv8621 | 0.973 | 0 | 0.744 between pos.21 and 22 |
| Fv8630 | 0.998 | 0.001 | 0.684 between pos.20 and 21 |
| Fv8651 | 1 | 0 | 0.929 between pos.23 and 24 |
| Fv8652 | 0.985 | 0 | 0.594 between pos.19 and 20 |
| Fv8654 | 0.985 | 0 | 0.689 between pos.17 and 18 |
| Fv8656 | 0.998 | 0 | 0.682 between pos.20 and 21 |
| Fv8672 | 0.979 | 0 | 0.494 between pos.23 and 24 |
| Fv8673 | 0.988 | 0 | 0.592 between pos.17 and 18 |
| Fv8675 | 0.998 | 0 | 0.925 between pos.16 and 17 |
| Fv8676 | 1 | 0 | 0.924 between pos.18 and 19 |
| Fv8684 | 0.986 | 0 | 0.754 between pos.15 and 16 |
| Fv8688 | 0.995 | 0 | 0.710 between pos.22 and 23 |
| Fv8691 | 0.999 | 0 | 0.860 between pos.17 and 18 |
| Fv8704 | 0.99 | 0 | 0.861 between pos.19 and 20 |
| Fv8719 | 1 | 0 | 0.942 between pos.16 and 17 |
| Fv8721 | 0.963 | 0 | 0.950 between pos.17 and 18 |
| Fv8731 | 0.996 | 0 | 0.581 between pos.18 and 19 |
| Fv8733 | 0.992 | 0 | 0.829 between pos.19 and 20 |
| Fv8742 | 0.997 | 0 | 0.333 between pos.17 and 18 |
| Fv8744 | 0.997 | 0 | 0.919 between pos.15 and 16 |
| Fv8745 | 0.954 | 0 | 0.765 between pos.20 and 21 |
| Fv8750 | 0.969 | 0 | 0.852 between pos.22 and 23 |
| Fv8752 | 0.999 | 0 | 0.619 between pos.20 and 21 |
| Fv8758 | 0.946 | 0 | 0.455 between pos.15 and 16 |
| Fv8760 | 0.995 | 0 | 0.488 between pos.18 and 19 |
| Fv8762 | 0.996 | 0 | 0.458 between pos.17 and 18 |
| Fv8765 | 0.994 | 0 | 0.858 between pos.19 and 20 |
| Fv8767 | 0.995 | 0 | 0.570 between pos.18 and 19 |
| Fv8778 | 0.956 | 0 | 0.851 between pos.18 and 19 |
| Fv8779 | 0.991 | 0 | 0.854 between pos.16 and 17 |
| Fv8784 | 0.998 | 0 | 0.374 between pos.20 and 21 |
| Fv8790 | 0.999 | 0 | 0.774 between pos.16 and 17 |
| Fv8791 | 0.992 | 0.001 | 0.903 between pos.18 and 19 |
| Fv8795 | 1 | 0 | 0.901 between pos.19 and 20 |
| Fv8805 | 0.946 | 0 | 0.350 between pos.17 and 18 |
| Fv8810 | 0.998 | 0 | 0.480 between pos.18 and 19 |
| Fv8812 | 0.983 | 0 | 0.963 between pos.17 and 18 |
| Fv8816 | 0.978 | 0 | 0.560 between pos.22 and 23 |
| Fv8828 | 1 | 0 | 0.974 between pos.21 and 22 |
| Fv8896 | 0.996 | 0 | 0.925 between pos.18 and 19 |
| Fv8948 | 0.999 | 0 | 0.759 between pos.16 and 17 |
| Fv8955 | 0.995 | 0 | 0.883 between pos.17 and 18 |
| Fv8971 | 0.996 | 0 | 0.987 between pos.19 and 20 |
| Fv8974 | 1 | 0 | 0.436 between pos.27 and 28 |
| Fv8977 | 0.988 | 0 | 0.942 between pos.20 and 21 |
| Fv8980 | 0.96 | 0 | 0.523 between pos.16 and 17 |
| Fv8989 | 1 | 0 | 0.957 between pos.18 and 19 |
| Fv8993 | 0.972 | 0 | 0.917 between pos.18 and 19 |
| Fv8996 | 1 | 0 | 0.900 between pos.17 and 18 |
| Fv9048 | 0.969 | 0 | 0.831 between pos.18 and 19 |
| Fv9080 | 1 | 0 | 0.869 between pos.20 and 21 |
| Fv9103 | 0.91 | 0.042 | 0.506 between pos.30 and 31 |
| Fv9120 | 0.954 | 0 | 0.913 between pos.17 and 18 |
| Fv9127 | 0.999 | 0 | 0.967 between pos.19 and 20 |
| Fv9143 | 0.998 | 0 | 0.847 between pos.16 and 17 |
| Fv9145 | 0.99 | 0 | 0.703 between pos.19 and 20 |
| Fv9152 | 0.999 | 0 | 0.925 between pos.21 and 22 |
| Fv9153 | 0.995 | 0 | 0.769 between pos.21 and 22 |
| Fv9163 | 0.999 | 0 | 0.849 between pos.21 and 22 |
| Fv9173 | 0.999 | 0 | 0.982 between pos.17 and 18 |
| Fv9200 | 0.917 | 0 | 0.782 between pos.16 and 17 |
| Fv9209 | 1 | 0 | 0.953 between pos.21 and 22 |
| Fv9210 | 0.997 | 0 | 0.915 between pos.18 and 19 |
| Fv9235 | 0.963 | 0 | 0.725 between pos.22 and 23 |
| Fv9253 | 0.988 | 0 | 0.695 between pos.17 and 18 |
| Fv9256 | 0.995 | 0 | 0.983 between pos.19 and 20 |
| Fv9259 | 0.999 | 0 | 0.614 between pos.23 and 24 |
| Fv9267 | 0.907 | 0 | 0.552 between pos.21 and 22 |
| Fv9281 | 0.984 | 0.001 | 0.487 between pos.30 and 31 |
| Fv9304 | 0.993 | 0 | 0.843 between pos.20 and 21 |
| Fv9314 | 1 | 0 | 0.215 between pos.23 and 24 |
| Fv9317 | 0.998 | 0 | 0.853 between pos.20 and 21 |
| Fv9454 | 0.997 | 0 | 0.968 between pos.20 and 21 |
| Fv9459 | 0.997 | 0 | 0.934 between pos.19 and 20 |
| Fv9464 | 0.994 | 0 | 0.794 between pos.18 and 19 |
| Fv9466 | 0.996 | 0 | 0.867 between pos.18 and 19 |
| Fv9480 | 0.997 | 0 | 0.744 between pos.18 and 19 |
| Fv9487 | 0.999 | 0.001 | 0.882 between pos.18 and 19 |
| Fv9488 | 0.992 | 0.001 | 0.847 between pos.18 and 19 |
| Fv9508 | 0.997 | 0.001 | 0.764 between pos.26 and 27 |
| Fv9515 | 0.91 | 0.089 | 0.324 between pos.31 and 32 |
| Fv9536 | 0.998 | 0 | 0.971 between pos.21 and 22 |
| Fv9550 | 0.998 | 0 | 0.961 between pos.18 and 19 |
| Fv9564 | 1 | 0 | 0.654 between pos.20 and 21 |
| Fv9597 | 0.91 | 0 | 0.803 between pos.18 and 19 |
| Fv9602 | 0.997 | 0 | 0.555 between pos.19 and 20 |
| Fv9604 | 0.93 | 0 | 0.362 between pos.20 and 21 |
| Fv9606 | 0.997 | 0 | 0.858 between pos.19 and 20 |
| Fv9608 | 0.987 | 0 | 0.323 between pos.22 and 23 |
| Fv9615 | 0.989 | 0 | 0.801 between pos.22 and 23 |
| Fv9623 | 1 | 0 | 0.975 between pos.22 and 23 |
| Fv9641 | 0.997 | 0 | 0.950 between pos.18 and 19 |
| Fv9652 | 0.981 | 0 | 0.448 between pos.20 and 21 |
| Fv9680 | 0.931 | 0 | 0.452 between pos.16 and 17 |
| Fv9711 | 0.96 | 0.011 | 0.941 between pos.29 and 30 |
| Fv9736 | 0.918 | 0 | 0.873 between pos.18 and 19 |
| Fv9762 | 0.999 | 0 | 0.627 between pos.18 and 19 |
| Fv9768 | 0.995 | 0 | 0.693 between pos.18 and 19 |
| Fv9782 | 1 | 0 | 0.417 between pos.17 and 18 |
| Fv9790 | 1 | 0 | 0.771 between pos.15 and 16 |
| Fv9792 | 0.998 | 0 | 0.948 between pos.19 and 20 |
| Fv9802 | 0.996 | 0 | 0.906 between pos.24 and 25 |
| Fv9805 | 1 | 0 | 0.751 between pos.19 and 20 |
| Fv9808 | 0.944 | 0 | 0.506 between pos.36 and 37 |
| Fv9816 | 0.999 | 0.001 | 0.462 between pos.20 and 21 |
| Fv9823 | 0.984 | 0 | 0.921 between pos.18 and 19 |
| Fv9825 | 0.975 | 0 | 0.392 between pos.21 and 22 |
| Fv9851 | 0.986 | 0 | 0.889 between pos.19 and 20 |
| Fv9855 | 0.944 | 0 | 0.393 between pos.16 and 17 |
| Fv9875 | 0.983 | 0 | 0.576 between pos.20 and 21 |
| Fv9879 | 0.918 | 0 | 0.843 between pos.19 and 20 |
| Fv9881 | 0.999 | 0 | 0.762 between pos.17 and 18 |
| Fv9912 | 1 | 0 | 0.827 between pos.20 and 21 |
| Fv9913 | 1 | 0 | 0.725 between pos.18 and 19 |
| Fv9917 | 0.976 | 0 | 0.406 between pos.30 and 31 |
| Fv9922 | 0.997 | 0 | 0.448 between pos.17 and 18 |
| Fv9925 | 0.997 | 0 | 0.832 between pos.18 and 19 |
| Fv9955 | 0.998 | 0 | 0.616 between pos.26 and 27 |
| Fv9977 | 0.993 | 0 | 0.405 between pos.19 and 20 |
| Fv9979 | 1 | 0 | 0.511 between pos.16 and 17 |
| Fv9988 | 0.987 | 0 | 0.786 between pos.20 and 21 |
| Fv9995 | 1 | 0 | 0.733 between pos.26 and 27 |
| Fv10001 | 0.989 | 0 | 0.808 between pos.21 and 22 |
| Fv10002 | 0.991 | 0 | 0.378 between pos.15 and 16 |
| Fv10008 | 0.985 | 0 | 0.979 between pos.20 and 21 |
| Fv10024 | 0.979 | 0 | 0.899 between pos.24 and 25 |
| Fv10025 | 0.997 | 0 | 0.892 between pos.20 and 21 |
| Fv10034 | 1 | 0 | 0.516 between pos.25 and 26 |
| Fv10049 | 0.999 | 0 | 0.780 between pos.24 and 25 |
| Fv10052 | 0.966 | 0 | 0.938 between pos.20 and 21 |
| Fv10056 | 0.958 | 0 | 0.815 between pos.23 and 24 |
| Fv10058 | 0.997 | 0 | 0.765 between pos.19 and 20 |
| Fv10061 | 0.999 | 0 | 0.980 between pos.18 and 19 |
| Fv10063 | 0.999 | 0 | 0.973 between pos.20 and 21 |
| Fv10082 | 0.989 | 0.003 | 0.269 between pos.22 and 23 |
| Fv10085 | 0.992 | 0 | 0.693 between pos.23 and 24 |
| Fv10100 | 0.983 | 0 | 0.870 between pos.19 and 20 |
| Fv10104 | 0.991 | 0 | 0.396 between pos.19 and 20 |
| Fv10121 | 1 | 0 | 0.760 between pos.20 and 21 |
| Fv10124 | 0.992 | 0 | 0.953 between pos.18 and 19 |
| Fv10128 | 0.982 | 0.006 | 0.501 between pos.19 and 20 |
| Fv10131 | 0.999 | 0 | 0.960 between pos.20 and 21 |
| Fv10138 | 0.989 | 0 | 0.326 between pos.19 and 20 |
| Fv10142 | 0.978 | 0 | 0.859 between pos.20 and 21 |
| Fv10164 | 1 | 0 | 0.909 between pos.17 and 18 |
| Fv10170 | 0.986 | 0 | 0.754 between pos.18 and 19 |
| Fv10193 | 0.981 | 0 | 0.424 between pos.18 and 19 |
| Fv10199 | 0.969 | 0 | 0.796 between pos.35 and 36 |
| Fv10201 | 0.997 | 0 | 0.606 between pos.19 and 20 |
| Fv10203 | 0.999 | 0 | 0.953 between pos.18 and 19 |
| Fv10206 | 0.986 | 0 | 0.953 between pos.17 and 18 |
| Fv10207 | 0.99 | 0 | 0.863 between pos.19 and 20 |
| Fv10216 | 0.908 | 0 | 0.904 between pos.21 and 22 |
| Fv10227 | 0.999 | 0 | 0.684 between pos.19 and 20 |
| Fv10242 | 0.998 | 0 | 0.862 between pos.20 and 21 |
| Fv10264 | 0.997 | 0 | 0.577 between pos.15 and 16 |
| Fv10268 | 0.992 | 0 | 0.944 between pos.21 and 22 |
| Fv10310 | 1 | 0 | 0.620 between pos.17 and 18 |
| Fv10356 | 1 | 0 | 0.994 between pos.18 and 19 |
| Fv10360 | 0.999 | 0 | 0.858 between pos.20 and 21 |
| Fv10373 | 0.979 | 0.019 | 0.435 between pos.26 and 27 |
| Fv10398 | 0.991 | 0 | 0.636 between pos.23 and 24 |
| Fv10414 | 0.981 | 0.001 | 0.460 between pos.41 and 42 |
| Fv10416 | 0.97 | 0.026 | 0.954 between pos.33 and 34 |
| Fv10444 | 0.995 | 0 | 0.919 between pos.16 and 17 |
| Fv10477 | 0.98 | 0 | 0.888 between pos.26 and 27 |
| Fv10481 | 0.93 | 0 | 0.455 between pos.21 and 22 |
| Fv10500 | 0.961 | 0 | 0.919 between pos.34 and 35 |
| Fv10507 | 0.976 | 0.014 | 0.784 between pos.37 and 38 |
| Fv10523 | 0.993 | 0 | 0.736 between pos.25 and 26 |
| Fv10525 | 1 | 0 | 0.917 between pos.21 and 22 |
| Fv10545 | 0.954 | 0 | 0.402 between pos.33 and 34 |
| Fv10546 | 0.962 | 0 | 0.552 between pos.16 and 17 |
| Fv10549 | 0.99 | 0 | 0.907 between pos.15 and 16 |
| Fv10551 | 0.996 | 0 | 0.963 between pos.21 and 22 |
| Fv10552 | 0.996 | 0 | 0.711 between pos.21 and 22 |
| Fv10564 | 0.983 | 0 | 0.563 between pos.23 and 24 |
| Fv10574 | 0.995 | 0 | 0.815 between pos.21 and 22 |
| Fv10586 | 0.997 | 0 | 0.868 between pos.23 and 24 |
| Fv10599 | 0.994 | 0.002 | 0.950 between pos.22 and 23 |
| Fv10632 | 0.989 | 0 | 0.981 between pos.20 and 21 |
| Fv10688 | 0.999 | 0 | 0.942 between pos.23 and 24 |
| Fv10692 | 1 | 0 | 0.542 between pos.16 and 17 |
| Fv10704 | 0.999 | 0 | 0.934 between pos.19 and 20 |
| Fv10715 | 0.996 | 0 | 0.927 between pos.20 and 21 |
| Fv10727 | 1 | 0 | 0.916 between pos.20 and 21 |
| Fv10748 | 0.931 | 0.005 | 0.346 between pos.19 and 20 |
| Fv10753 | 1 | 0 | 0.987 between pos.16 and 17 |
| Fv10766 | 0.996 | 0 | 0.767 between pos.23 and 24 |
| Fv10769 | 0.999 | 0 | 0.782 between pos.20 and 21 |
| Fv10779 | 0.998 | 0 | 0.454 between pos.23 and 24 |
| Fv10785 | 1 | 0 | 0.786 between pos.18 and 19 |
| Fv10786 | 0.975 | 0 | 0.466 between pos.21 and 22 |
| Fv10795 | 0.96 | 0.002 | 0.497 between pos.36 and 37 |
| Fv10799 | 0.989 | 0 | 0.398 between pos.16 and 17 |
| Fv10821 | 0.968 | 0 | 0.966 between pos.19 and 20 |
| Fv10822 | 0.999 | 0 | 0.942 between pos.20 and 21 |
| Fv10823 | 0.998 | 0 | 0.900 between pos.26 and 27 |
| Fv10826 | 0.999 | 0 | 0.965 between pos.20 and 21 |
| Fv10829 | 1 | 0 | 0.434 between pos.22 and 23 |
| Fv10845 | 0.998 | 0 | 0.525 between pos.20 and 21 |
| Fv10848 | 0.998 | 0 | 0.888 between pos.15 and 16 |
| Fv10878 | 0.918 | 0 | 0.813 between pos.27 and 28 |
| Fv10917 | 0.977 | 0 | 0.465 between pos.21 and 22 |
| Fv10937 | 0.998 | 0 | 0.676 between pos.24 and 25 |
| Fv10944 | 0.936 | 0.001 | 0.646 between pos.25 and 26 |
| Fv10953 | 0.931 | 0.002 | 0.496 between pos.22 and 23 |
| Fv10994 | 0.999 | 0.001 | 0.944 between pos.25 and 26 |
| Fv10998 | 0.999 | 0 | 0.979 between pos.16 and 17 |
| Fv11043 | 0.984 | 0 | 0.894 between pos.21 and 22 |
| Fv11049 | 0.999 | 0 | 0.934 between pos.24 and 25 |
| Fv11065 | 0.996 | 0.001 | 0.830 between pos.20 and 21 |
| Fv11072 | 0.997 | 0.002 | 0.505 between pos.44 and 45 |
| Fv11082 | 0.994 | 0 | 0.918 between pos.20 and 21 |
| Fv11087 | 0.923 | 0 | 0.523 between pos.17 and 18 |
| Fv11088 | 0.981 | 0 | 0.792 between pos.22 and 23 |
| Fv11093 | 0.999 | 0 | 0.907 between pos.22 and 23 |
| Fv11097 | 0.995 | 0 | 0.847 between pos.19 and 20 |
| Fv11100 | 0.993 | 0.002 | 0.790 between pos.20 and 21 |
| Fv11101 | 0.999 | 0 | 0.827 between pos.18 and 19 |
| Fv11107 | 0.998 | 0 | 0.988 between pos.20 and 21 |
| Fv11109 | 0.999 | 0.001 | 0.697 between pos.20 and 21 |
| Fv11112 | 0.999 | 0 | 0.968 between pos.18 and 19 |
| Fv11113 | 0.999 | 0 | 0.813 between pos.17 and 18 |
| Fv11125 | 0.999 | 0 | 0.938 between pos.18 and 19 |
| Fv11127 | 0.98 | 0 | 0.678 between pos.19 and 20 |
| Fv11152 | 0.979 | 0.005 | 0.234 between pos.25 and 26 |
| Fv11153 | 0.925 | 0.034 | 0.332 between pos.30 and 31 |
| Fv11184 | 0.922 | 0 | 0.738 between pos.24 and 25 |
| Fv11185 | 0.998 | 0 | 0.860 between pos.17 and 18 |
| Fv11196 | 1 | 0 | 0.944 between pos.20 and 21 |
| Fv11208 | 0.968 | 0 | 0.930 between pos.18 and 19 |
| Fv11217 | 0.963 | 0 | 0.909 between pos.20 and 21 |
| Fv11219 | 0.964 | 0 | 0.662 between pos.20 and 21 |
| Fv11256 | 0.991 | 0.001 | 0.745 between pos.20 and 21 |
| Fv11258 | 0.901 | 0 | 0.801 between pos.22 and 23 |
| Fv11290 | 0.999 | 0 | 0.974 between pos.19 and 20 |
| Fv11298 | 0.999 | 0 | 0.877 between pos.18 and 19 |
| Fv11319 | 0.999 | 0 | 0.578 between pos.17 and 18 |
| Fv11330 | 0.994 | 0 | 0.991 between pos.19 and 20 |
| Fv11340 | 0.974 | 0 | 0.775 between pos.23 and 24 |
| Fv11343 | 0.999 | 0 | 0.496 between pos.26 and 27 |
| Fv11344 | 0.999 | 0 | 0.956 between pos.19 and 20 |
| Fv11345 | 0.994 | 0 | 0.343 between pos.22 and 23 |
| Fv11353 | 0.996 | 0 | 0.917 between pos.15 and 16 |
| Fv11354 | 0.997 | 0 | 0.596 between pos.14 and 15 |
| Fv11365 | 0.965 | 0 | 0.616 between pos.23 and 24 |
| Fv11372 | 1 | 0 | 0.844 between pos.20 and 21 |
| Fv11377 | 0.947 | 0 | 0.468 between pos.21 and 22 |
| Fv11378 | 0.998 | 0 | 0.704 between pos.20 and 21 |
| Fv11390 | 1 | 0 | 0.900 between pos.17 and 18 |
| Fv11394 | 0.999 | 0 | 0.687 between pos.19 and 20 |
| Fv11402 | 0.958 | 0 | 0.807 between pos.18 and 19 |
| Fv11404 | 0.978 | 0 | 0.410 between pos.16 and 17 |
| Fv11411 | 1 | 0 | 0.561 between pos.20 and 21 |
| Fv11415 | 1 | 0 | 0.990 between pos.21 and 22 |
| Fv11416 | 0.99 | 0 | 0.846 between pos.19 and 20 |
| Fv11430 | 0.991 | 0 | 0.969 between pos.19 and 20 |
| Fv11456 | 0.999 | 0 | 0.573 between pos.16 and 17 |
| Fv11482 | 0.981 | 0 | 0.508 between pos.15 and 16 |
| Fv11500 | 0.971 | 0.022 | 0.348 between pos.34 and 35 |
| Fv11546 | 0.991 | 0 | 0.741 between pos.19 and 20 |
| Fv11552 | 0.997 | 0 | 0.928 between pos.17 and 18 |
| Fv11559 | 0.992 | 0 | 0.503 between pos.17 and 18 |
| Fv11566 | 0.993 | 0 | 0.289 between pos.17 and 18 |
| Fv11567 | 1 | 0 | 0.962 between pos.18 and 19 |
| Fv11574 | 0.908 | 0 | 0.394 between pos.15 and 16 |
| Fv11580 | 0.991 | 0 | 0.773 between pos.17 and 18 |
| Fv11586 | 0.998 | 0 | 0.372 between pos.26 and 27 |
| Fv11590 | 0.983 | 0 | 0.770 between pos.21 and 22 |
| Fv11591 | 0.972 | 0 | 0.923 between pos.21 and 22 |
| Fv11592 | 0.97 | 0 | 0.773 between pos.21 and 22 |
| Fv11597 | 0.994 | 0 | 0.409 between pos.19 and 20 |
| Fv11600 | 0.99 | 0 | 0.705 between pos.18 and 19 |
| Fv11602 | 0.991 | 0.001 | 0.332 between pos.34 and 35 |
| Fv11603 | 0.967 | 0 | 0.876 between pos.17 and 18 |
| Fv11621 | 0.929 | 0 | 0.919 between pos.17 and 18 |
| Fv11622 | 0.996 | 0 | 0.849 between pos.17 and 18 |
| Fv11623 | 0.986 | 0 | 0.772 between pos.16 and 17 |
| Fv11626 | 0.988 | 0 | 0.933 between pos.17 and 18 |
| Fv11632 | 0.997 | 0 | 0.790 between pos.18 and 19 |
| Fv11634 | 1 | 0 | 0.828 between pos.24 and 25 |
| Fv11635 | 0.945 | 0 | 0.858 between pos.23 and 24 |
| Fv11636 | 0.996 | 0 | 0.959 between pos.20 and 21 |
| Fv11640 | 0.988 | 0 | 0.748 between pos.20 and 21 |
| Fv11655 | 0.937 | 0 | 0.777 between pos.21 and 22 |
| Fv11665 | 0.998 | 0 | 0.827 between pos.16 and 17 |
| Fv11671 | 1 | 0 | 0.828 between pos.20 and 21 |
| Fv11672 | 0.947 | 0 | 0.570 between pos.21 and 22 |
| Fv11677 | 0.999 | 0 | 0.755 between pos.20 and 21 |
| Fv11692 | 0.998 | 0 | 0.964 between pos.21 and 22 |
| Fv11699 | 0.999 | 0 | 0.997 between pos.19 and 20 |
| Fv11700 | 1 | 0 | 0.866 between pos.22 and 23 |
| Fv11701 | 0.996 | 0 | 0.630 between pos.21 and 22 |
| Fv11705 | 0.99 | 0 | 0.976 between pos.25 and 26 |
| Fv11724 | 0.998 | 0 | 0.876 between pos.19 and 20 |
| Fv11732 | 0.999 | 0 | 0.783 between pos.15 and 16 |
| Fv11733 | 1 | 0 | 0.994 between pos.19 and 20 |
| Fv11742 | 0.991 | 0 | 0.519 between pos.21 and 22 |
| Fv11743 | 0.999 | 0 | 0.568 between pos.18 and 19 |
| Fv11754 | 0.998 | 0 | 0.920 between pos.17 and 18 |
| Fv11757 | 0.993 | 0 | 0.954 between pos.16 and 17 |
| Fv11758 | 0.993 | 0 | 0.355 between pos.32 and 33 |
| Fv11778 | 0.997 | 0 | 0.936 between pos.19 and 20 |
| Fv11780 | 0.978 | 0 | 0.695 between pos.17 and 18 |
| Fv11796 | 0.992 | 0 | 0.986 between pos.23 and 24 |
| Fv11797 | 0.987 | 0 | 0.523 between pos.26 and 27 |
| Fv11812 | 0.982 | 0 | 0.824 between pos.18 and 19 |
| Fv11815 | 0.999 | 0 | 0.973 between pos.19 and 20 |
| Fv11819 | 0.994 | 0 | 0.912 between pos.18 and 19 |
| Fv11822 | 0.967 | 0 | 0.712 between pos.19 and 20 |
| Fv11836 | 0.992 | 0 | 0.512 between pos.17 and 18 |
| Fv11856 | 1 | 0 | 0.884 between pos.19 and 20 |
| Fv11861 | 1 | 0 | 0.493 between pos.26 and 27 |
| Fv11867 | 0.989 | 0.001 | 0.481 between pos.25 and 26 |
| Fv11872 | 0.997 | 0 | 0.975 between pos.18 and 19 |
| Fv11873 | 0.986 | 0.01 | 0.824 between pos.34 and 35 |
| Fv11885 | 0.954 | 0 | 0.825 between pos.15 and 16 |
| Fv11893 | 0.917 | 0 | 0.792 between pos.22 and 23 |
| Fv11898 | 0.999 | 0 | 0.905 between pos.19 and 20 |
| Fv11919 | 0.962 | 0 | 0.913 between pos.22 and 23 |
| Fv11934 | 1 | 0 | 0.919 between pos.20 and 21 |
| Fv11958 | 0.959 | 0 | 0.849 between pos.22 and 23 |
| Fv11961 | 0.965 | 0 | 0.594 between pos.20 and 21 |
| Fv11973 | 1 | 0 | 0.948 between pos.17 and 18 |
| Fv11991 | 1 | 0 | 0.926 between pos.22 and 23 |
| Fv12006 | 0.926 | 0.001 | 0.647 between pos.45 and 46 |
| Fv12044 | 0.977 | 0.008 | 0.395 between pos.28 and 29 |
| Fv12056 | 0.968 | 0 | 0.821 between pos.19 and 20 |
| Fv12058 | 0.996 | 0 | 0.985 between pos.19 and 20 |
| Fv12068 | 0.994 | 0 | 0.670 between pos.27 and 28 |
| Fv12069 | 0.999 | 0 | 0.734 between pos.19 and 20 |
| Fv12070 | 0.992 | 0.001 | 0.915 between pos.28 and 29 |
| Fv12073 | 1 | 0 | 0.885 between pos.17 and 18 |
| Fv12081 | 0.995 | 0 | 0.854 between pos.19 and 20 |
| Fv12083 | 0.93 | 0 | 0.759 between pos.20 and 21 |
| Fv12092 | 0.996 | 0 | 0.580 between pos.22 and 23 |
| Fv12100 | 0.998 | 0 | 0.967 between pos.19 and 20 |
| Fv12105 | 0.998 | 0 | 0.617 between pos.17 and 18 |
| Fv12110 | 0.997 | 0 | 0.732 between pos.18 and 19 |
| Fv12130 | 0.997 | 0 | 0.665 between pos.18 and 19 |
| Fv12131 | 0.965 | 0.001 | 0.337 between pos.21 and 22 |
| Fv12135 | 0.99 | 0 | 0.759 between pos.17 and 18 |
| Fv12136 | 0.987 | 0 | 0.304 between pos.24 and 25 |
| Fv12145 | 0.963 | 0 | 0.649 between pos.19 and 20 |
| Fv12147 | 1 | 0 | 0.923 between pos.18 and 19 |
| Fv12151 | 0.999 | 0 | 0.969 between pos.18 and 19 |
| Fv12159 | 0.932 | 0 | 0.829 between pos.20 and 21 |
| Fv12171 | 0.999 | 0 | 0.478 between pos.24 and 25 |
| Fv12174 | 0.977 | 0 | 0.527 between pos.19 and 20 |
| Fv12201 | 0.998 | 0 | 0.491 between pos.22 and 23 |
| Fv12211 | 0.998 | 0 | 0.877 between pos.16 and 17 |
| Fv12214 | 0.993 | 0 | 0.399 between pos.26 and 27 |
| Fv12227 | 0.997 | 0 | 0.898 between pos.18 and 19 |
| Fv12230 | 0.997 | 0 | 0.541 between pos.18 and 19 |
| Fv12231 | 0.99 | 0 | 0.828 between pos.17 and 18 |
| Fv12236 | 0.999 | 0 | 0.872 between pos.18 and 19 |
| Fv12251 | 0.998 | 0 | 0.960 between pos.18 and 19 |
| Fv12252 | 0.999 | 0 | 0.848 between pos.17 and 18 |
| Fv12255 | 1 | 0 | 0.980 between pos.21 and 22 |
| Fv12257 | 0.968 | 0.003 | 0.838 between pos.31 and 32 |
| Fv12259 | 0.943 | 0 | 0.864 between pos.21 and 22 |
| Fv12283 | 0.985 | 0 | 0.588 between pos.24 and 25 |
| Fv12285 | 0.995 | 0 | 0.925 between pos.17 and 18 |
| Fv12286 | 0.934 | 0.05 | 0.714 between pos.34 and 35 |
| Fv12287 | 1 | 0 | 0.886 between pos.19 and 20 |
| Fv12291 | 0.995 | 0.004 | 0.986 between pos.22 and 23 |
| Fv12304 | 0.999 | 0 | 0.775 between pos.22 and 23 |
| Fv12317 | 1 | 0 | 0.975 between pos.20 and 21 |
| Fv12323 | 0.98 | 0.006 | 0.531 between pos.25 and 26 |
| Fv12334 | 0.999 | 0 | 0.984 between pos.18 and 19 |
| Fv12335 | 0.906 | 0 | 0.541 between pos.18 and 19 |
| Fv12352 | 0.995 | 0.001 | 0.657 between pos.20 and 21 |
| Fv12367 | 0.951 | 0 | 0.939 between pos.20 and 21 |
| Fv12371 | 1 | 0 | 0.979 between pos.18 and 19 |
| Fv12372 | 0.998 | 0 | 0.819 between pos.23 and 24 |
| Fv12373 | 1 | 0 | 0.878 between pos.16 and 17 |
| Fv12383 | 0.998 | 0 | 0.774 between pos.21 and 22 |
| Fv12388 | 0.976 | 0 | 0.872 between pos.16 and 17 |
| Fv12423 | 0.974 | 0.023 | 0.573 between pos.47 and 48 |
| Fv12431 | 0.998 | 0 | 0.946 between pos.24 and 25 |
| Fv12436 | 0.975 | 0.003 | 0.630 between pos.19 and 20 |
| Fv12452 | 1 | 0 | 0.740 between pos.20 and 21 |
| Fv12459 | 0.998 | 0 | 0.920 between pos.19 and 20 |
| Fv12461 | 0.999 | 0 | 0.991 between pos.20 and 21 |
| Fv12464 | 0.999 | 0 | 0.496 between pos.23 and 24 |
| Fv12470 | 0.964 | 0 | 0.870 between pos.18 and 19 |
| Fv12499 | 0.969 | 0 | 0.913 between pos.26 and 27 |
| Fv12501 | 0.981 | 0 | 0.573 between pos.21 and 22 |
| Fv12504 | 0.969 | 0 | 0.463 between pos.19 and 20 |
| Fv12508 | 0.988 | 0 | 0.621 between pos.21 and 22 |
| Fv12509 | 0.998 | 0 | 0.952 between pos.16 and 17 |
| Fv12515 | 0.981 | 0.005 | 0.775 between pos.30 and 31 |
| Fv12523 | 0.997 | 0 | 0.977 between pos.17 and 18 |
| Fv12555 | 0.995 | 0 | 0.428 between pos.15 and 16 |
| Fv12558 | 0.976 | 0 | 0.395 between pos.20 and 21 |
| Fv12581 | 0.996 | 0 | 0.732 between pos.20 and 21 |
| Fv12608 | 1 | 0 | 0.856 between pos.21 and 22 |
| Fv12632 | 0.992 | 0 | 0.857 between pos.19 and 20 |
| Fv12666 | 1 | 0 | 0.802 between pos.25 and 26 |
| Fv12670 | 0.962 | 0 | 0.776 between pos.19 and 20 |
| Fv12674 | 0.995 | 0.001 | 0.577 between pos.18 and 19 |
| Fv12686 | 0.951 | 0 | 0.761 between pos.20 and 21 |
| Fv12694 | 0.999 | 0 | 0.967 between pos.18 and 19 |
| Fv12709 | 0.999 | 0 | 0.591 between pos.17 and 18 |
| Fv12725 | 0.997 | 0 | 0.525 between pos.23 and 24 |
| Fv12729 | 1 | 0 | 0.882 between pos.18 and 19 |
| Fv12736 | 0.921 | 0 | 0.718 between pos.19 and 20 |
| Fv12750 | 1 | 0 | 0.951 between pos.19 and 20 |
| Fv12752 | 0.986 | 0 | 0.458 between pos.19 and 20 |
| Fv12756 | 0.999 | 0 | 0.955 between pos.20 and 21 |
| Fv12761 | 0.929 | 0 | 0.457 between pos.18 and 19 |
| Fv12769 | 1 | 0 | 0.943 between pos.21 and 22 |
| Fv12789 | 0.997 | 0 | 0.518 between pos.16 and 17 |
| Fv12795 | 0.997 | 0 | 0.988 between pos.18 and 19 |
| Fv12801 | 1 | 0 | 0.944 between pos.18 and 19 |
| Fv12808 | 1 | 0 | 0.972 between pos.20 and 21 |
| Fv12809 | 0.997 | 0 | 0.992 between pos.23 and 24 |
| Fv12815 | 0.98 | 0 | 0.821 between pos.19 and 20 |
| Fv12818 | 0.99 | 0 | 0.893 between pos.19 and 20 |
| Fv12821 | 0.999 | 0 | 0.894 between pos.25 and 26 |
| Fv12827 | 0.993 | 0 | 0.386 between pos.24 and 25 |
| Fv12838 | 0.994 | 0 | 0.957 between pos.22 and 23 |
| Fv12844 | 1 | 0 | 0.914 between pos.19 and 20 |
| Fv12846 | 0.972 | 0 | 0.749 between pos.32 and 33 |
| Fv12856 | 0.932 | 0 | 0.477 between pos.28 and 29 |
| Fv12861 | 0.956 | 0.004 | 0.590 between pos.24 and 25 |
| Fv12867 | 0.959 | 0 | 0.417 between pos.15 and 16 |
| Fv12893 | 0.998 | 0 | 0.467 between pos.16 and 17 |
| Fv12894 | 0.994 | 0 | 0.845 between pos.15 and 16 |
| Fv12911 | 0.999 | 0 | 0.377 between pos.20 and 21 |
| Fv12921 | 1 | 0 | 0.632 between pos.26 and 27 |
| Fv12926 | 0.998 | 0 | 0.811 between pos.19 and 20 |
| Fv12929 | 1 | 0 | 0.496 between pos.18 and 19 |
| Fv12932 | 0.926 | 0 | 0.484 between pos.15 and 16 |
| Fv12962 | 0.989 | 0 | 0.560 between pos.23 and 24 |
| Fv12970 | 0.997 | 0 | 0.831 between pos.15 and 16 |
| Fv12986 | 0.99 | 0 | 0.502 between pos.17 and 18 |
| Fv12993 | 0.967 | 0 | 0.802 between pos.25 and 26 |
| Fv13035 | 0.997 | 0.001 | 0.958 between pos.26 and 27 |
| Fv13062 | 0.997 | 0 | 0.766 between pos.17 and 18 |
| Fv13066 | 1 | 0 | 0.948 between pos.20 and 21 |
| Fv13072 | 0.998 | 0 | 0.939 between pos.16 and 17 |
| Fv13081 | 0.996 | 0 | 0.759 between pos.19 and 20 |
| Fv13090 | 0.999 | 0 | 0.663 between pos.21 and 22 |
| Fv13092 | 0.998 | 0.001 | 0.870 between pos.18 and 19 |
| Fv13096 | 0.981 | 0 | 0.959 between pos.24 and 25 |
| Fv13097 | 1 | 0 | 0.952 between pos.21 and 22 |
| Fv13099 | 0.999 | 0 | 0.991 between pos.24 and 25 |
| Fv13104 | 0.989 | 0 | 0.761 between pos.19 and 20 |
| Fv13105 | 0.99 | 0 | 0.367 between pos.14 and 15 |
| Fv13113 | 0.987 | 0 | 0.909 between pos.23 and 24 |
| Fv13146 | 0.996 | 0 | 0.820 between pos.18 and 19 |
| Fv13150 | 0.94 | 0.004 | 0.404 between pos.23 and 24 |
| Fv13174 | 1 | 0 | 0.848 between pos.23 and 24 |
| Fv13175 | 1 | 0 | 0.991 between pos.22 and 23 |
| Fv13177 | 1 | 0 | 0.889 between pos.19 and 20 |
| Fv13208 | 0.991 | 0 | 0.983 between pos.16 and 17 |
| Fv13210 | 0.97 | 0 | 0.903 between pos.23 and 24 |
| Fv13214 | 0.994 | 0 | 0.872 between pos.19 and 20 |
| Fv13223 | 0.999 | 0 | 0.646 between pos.15 and 16 |
| Fv13234 | 0.961 | 0 | 0.895 between pos.22 and 23 |
| Fv13238 | 0.996 | 0 | 0.655 between pos.17 and 18 |
| Fv13251 | 0.957 | 0 | 0.847 between pos.19 and 20 |
| Fv13252 | 0.993 | 0 | 0.439 between pos.18 and 19 |
| Fv13259 | 0.995 | 0 | 0.912 between pos.19 and 20 |
| Fv13264 | 0.999 | 0 | 0.903 between pos.19 and 20 |
| Fv13270 | 0.971 | 0 | 0.610 between pos.19 and 20 |
| Fv13271 | 0.999 | 0 | 0.606 between pos.20 and 21 |
| Fv13285 | 0.999 | 0 | 0.889 between pos.18 and 19 |
| Fv13294 | 0.997 | 0.002 | 0.642 between pos.19 and 20 |
| Fv13306 | 0.991 | 0 | 0.651 between pos.19 and 20 |
| Fv13316 | 1 | 0 | 0.898 between pos.22 and 23 |
| Fv13325 | 0.999 | 0 | 0.864 between pos.25 and 26 |
| Fv13345 | 0.998 | 0 | 0.876 between pos.19 and 20 |
| Fv13346 | 0.991 | 0 | 0.530 between pos.17 and 18 |
| Fv13351 | 1 | 0 | 0.948 between pos.25 and 26 |
| Fv13353 | 0.991 | 0 | 0.685 between pos.17 and 18 |
| Fv13370 | 0.983 | 0 | 0.961 between pos.30 and 31 |
| Fv13392 | 0.993 | 0 | 0.901 between pos.18 and 19 |
| Fv13425 | 0.987 | 0 | 0.665 between pos.16 and 17 |
| Fv13437 | 0.999 | 0 | 0.919 between pos.19 and 20 |
| Fv13484 | 0.989 | 0 | 0.281 between pos.19 and 20 |
| Fv13489 | 0.974 | 0 | 0.572 between pos.39 and 40 |
| Fv13521 | 0.997 | 0 | 0.750 between pos.22 and 23 |
| Fv13522 | 0.938 | 0 | 0.857 between pos.23 and 24 |
| Fv13524 | 0.986 | 0 | 0.696 between pos.16 and 17 |
| Fv13526 | 1 | 0 | 0.895 between pos.20 and 21 |
| Fv13527 | 0.991 | 0 | 0.816 between pos.18 and 19 |
| Fv13537 | 0.967 | 0 | 0.936 between pos.19 and 20 |
| Fv13539 | 0.996 | 0 | 0.903 between pos.19 and 20 |
| Fv13540 | 0.996 | 0 | 0.925 between pos.36 and 37 |
| Fv13548 | 0.983 | 0 | 0.911 between pos.19 and 20 |
| Fv13550 | 1 | 0 | 0.782 between pos.22 and 23 |
| Fv13553 | 0.989 | 0 | 0.604 between pos.18 and 19 |
| Fv13562 | 1 | 0 | 0.261 between pos.18 and 19 |
| Fv13563 | 0.994 | 0 | 0.813 between pos.19 and 20 |
| Fv13564 | 0.997 | 0 | 0.404 between pos.23 and 24 |
| Fv13573 | 0.981 | 0 | 0.839 between pos.18 and 19 |
| Fv13577 | 0.993 | 0 | 0.447 between pos.18 and 19 |
| Fv13593 | 0.999 | 0.001 | 0.539 between pos.20 and 21 |
| Fv13617 | 0.985 | 0 | 0.881 between pos.17 and 18 |
| Fv13618 | 0.988 | 0.009 | 0.329 between pos.33 and 34 |
| Fv13620 | 0.986 | 0 | 0.640 between pos.18 and 19 |
| Fv13657 | 0.912 | 0 | 0.508 between pos.21 and 22 |
| Fv13663 | 1 | 0 | 0.787 between pos.18 and 19 |
| Fv13723 | 0.995 | 0 | 0.948 between pos.17 and 18 |
| Fv13732 | 0.932 | 0.011 | 0.404 between pos.20 and 21 |
| Fv13746 | 0.997 | 0 | 0.593 between pos.21 and 22 |
| Fv13748 | 0.999 | 0 | 0.811 between pos.22 and 23 |
| Fv13757 | 0.985 | 0 | 0.863 between pos.17 and 18 |
| Fv13762 | 0.991 | 0 | 0.468 between pos.22 and 23 |
| Fv13775 | 0.959 | 0.007 | 0.516 between pos.19 and 20 |
| Fv13777 | 0.999 | 0 | 0.698 between pos.20 and 21 |
| Fv13804 | 1 | 0 | 0.978 between pos.19 and 20 |
| Fv13825 | 0.998 | 0 | 0.953 between pos.20 and 21 |
| Fv13851 | 0.995 | 0 | 0.618 between pos.18 and 19 |
| Fv13878 | 0.987 | 0 | 0.675 between pos.21 and 22 |
| Fv13901 | 0.999 | 0 | 0.874 between pos.18 and 19 |
| Fv13913 | 0.932 | 0 | 0.642 between pos.18 and 19 |
| Fv13936 | 0.928 | 0.001 | 0.898 between pos.21 and 22 |
| Fv13957 | 0.975 | 0.001 | 0.856 between pos.26 and 27 |
| Fv13959 | 0.998 | 0 | 0.506 between pos.21 and 22 |
| Fv13978 | 0.999 | 0 | 0.833 between pos.18 and 19 |
| Fv13989 | 0.997 | 0 | 0.922 between pos.18 and 19 |
| Fv13992 | 0.999 | 0 | 0.493 between pos.20 and 21 |
| Fv13998 | 0.998 | 0 | 0.735 between pos.19 and 20 |
| Fv14010 | 0.993 | 0 | 0.574 between pos.25 and 26 |
| Fv14022 | 0.935 | 0 | 0.759 between pos.25 and 26 |
| Fv14024 | 0.985 | 0 | 0.625 between pos.19 and 20 |
| Fv14025 | 0.978 | 0 | 0.890 between pos.23 and 24 |
| Fv14026 | 0.99 | 0 | 0.979 between pos.20 and 21 |
| Fv14034 | 0.947 | 0 | 0.538 between pos.15 and 16 |
| Fv14105 | 0.999 | 0 | 0.548 between pos.21 and 22 |
| Fv14106 | 0.997 | 0 | 0.738 between pos.17 and 18 |
| Fv14113 | 0.997 | 0.001 | 0.651 between pos.20 and 21 |
| Fv14114 | 0.995 | 0 | 0.752 between pos.16 and 17 |
| Fv14115 | 0.999 | 0 | 0.726 between pos.25 and 26 |
| Fv14123 | 0.999 | 0 | 0.870 between pos.17 and 18 |
| Fv14125 | 0.993 | 0 | 0.932 between pos.18 and 19 |
| Fv14140 | 0.999 | 0 | 0.678 between pos.18 and 19 |
| Fv14155 | 0.997 | 0 | 0.800 between pos.18 and 19 |
| Fv14158 | 0.988 | 0 | 0.756 between pos.17 and 18 |
| Fv14166 | 1 | 0 | 0.559 between pos.20 and 21 |
| Fv14185 | 0.988 | 0 | 0.925 between pos.19 and 20 |
| Fv14206 | 0.915 | 0 | 0.306 between pos.20 and 21 |
| Fv14213 | 0.999 | 0 | 0.698 between pos.19 and 20 |
| Fv14217 | 0.999 | 0 | 0.816 between pos.17 and 18 |
| Fv14218 | 0.999 | 0 | 0.975 between pos.17 and 18 |
| Fv14221 | 0.999 | 0 | 0.889 between pos.23 and 24 |
| Fv14273 | 0.995 | 0 | 0.960 between pos.21 and 22 |
| Fv14275 | 0.981 | 0 | 0.505 between pos.18 and 19 |
| Fv14276 | 0.992 | 0 | 0.571 between pos.19 and 20 |
| Fv14278 | 0.997 | 0 | 0.534 between pos.21 and 22 |
| Fv14346 | 0.989 | 0 | 0.780 between pos.18 and 19 |
| Fv14347 | 0.974 | 0 | 0.635 between pos.25 and 26 |
| Fv14384 | 0.998 | 0 | 0.988 between pos.20 and 21 |
| Fv14396 | 0.997 | 0 | 0.421 between pos.18 and 19 |
| Fv14488 | 0.935 | 0.061 | 0.328 between pos.24 and 25 |
| Fv14494 | 0.978 | 0.001 | 0.805 between pos.40 and 41 |
| Fv14500 | 0.995 | 0 | 0.850 between pos.15 and 16 |
| Fv14504 | 0.99 | 0 | 0.940 between pos.17 and 18 |
| Fv14510 | 0.992 | 0 | 0.906 between pos.17 and 18 |
| Fv14515 | 0.999 | 0 | 0.988 between pos.18 and 19 |
| Fv14578 | 1 | 0 | 0.901 between pos.18 and 19 |
| Fv14620 | 0.999 | 0 | 0.496 between pos.18 and 19 |
| Fv14643 | 0.994 | 0 | 0.669 between pos.25 and 26 |
| Fv14646 | 0.945 | 0.016 | 0.374 between pos.51 and 52 |
| Fv14663 | 0.97 | 0.001 | 0.817 between pos.19 and 20 |
| Fv14667 | 1 | 0 | 0.933 between pos.20 and 21 |
| Fv14671 | 0.956 | 0 | 0.744 between pos.21 and 22 |
| Fv14703 | 0.931 | 0 | 0.295 between pos.16 and 17 |
| Fv14704 | 0.998 | 0 | 0.947 between pos.19 and 20 |
| Fv14713 | 0.933 | 0 | 0.337 between pos.30 and 31 |
| Fv14719 | 0.967 | 0 | 0.739 between pos.20 and 21 |
| Fv14720 | 0.985 | 0.003 | 0.817 between pos.25 and 26 |
| Fv14721 | 0.999 | 0 | 0.969 between pos.18 and 19 |
| Fv14722 | 1 | 0 | 0.898 between pos.19 and 20 |
| Fv14726 | 0.992 | 0 | 0.932 between pos.19 and 20 |
| Fv14747 | 0.982 | 0 | 0.747 between pos.23 and 24 |
| Fv14748 | 0.991 | 0 | 0.927 between pos.23 and 24 |
| Fv14751 | 0.998 | 0 | 0.814 between pos.18 and 19 |
| Fv14802 | 0.977 | 0 | 0.517 between pos.22 and 23 |
| Fv14818 | 0.998 | 0 | 0.930 between pos.19 and 20 |
